# Upcycling of waste sodium sulfate to sodium carbonate and sulfur

**DOI:** 10.1038/s41467-026-72286-y

**Published:** 2026-04-24

**Authors:** Hongya Wang, Shiyu Wang, Fengyin Zhou, Muya Cai, Jingjing Zhao, Danfeng Wang, Zhan Shi, Xiang Chen, Dihua Wang, Huayi Yin

**Affiliations:** 1 School of Resource and Environmental Sciences, Wuhan University, Wuhan, PR China; 2 Department of Land, Environment, Agriculture and Forestry, University of Padova, Padova, Italy; 3 Hubei International Scientific and Technological Cooperation Base of Sustainable Resources and Energy, Wuhan University, Wuhan, PR China; 4 Joint Center of Green Manufacturing of Energy Storage Materials of Wuhan University and Chilwee, Wuhan, PR China

**Keywords:** Pollution remediation, Inorganic chemistry, Chemical engineering

## Abstract

Waste sodium sulfate (Na_2_SO_4_) is a common industrial byproduct that poses environmental risks and resource loss if improperly managed. Here, we report a thermochemical upcycling method to convert waste Na_2_SO_4_ into value-added sodium carbonate (Na_2_CO_3_) and sulfur (S_x_). In this process, Na_2_SO_4_ is first reduced to sodium sulfide (Na_2_S) at 750 °C using charcoal. Subsequently, the generated Na_2_S is oxidized by CO_2_ via carbonation at 300 °C to produce Na_2_CO_3_ and S_x_. Temperature modulation shifts thermodynamic equilibrium to drive the conversion of SO_4_^2−^ to S_x_, achieving a carbonate yield of 95.35% with purity exceeding 99.53%. Life cycle assessment (LCA) indicates that this anhydrous route reduces the global warming potential by > 0.43 kg CO_2_-eq per kg Na_2_CO_3_ compared with the conventional sodium sulfate-based ammonia-soda process (SSA-Process). By eliminating water-intensive steps and ammonia (NH_3_) usage, our method lowers the end-point environmental impact to 34.69 mPt per kg Na_2_CO_3_ (vs. 48.81 mPt for conventional routes). Overall, this work provides a sustainable strategy for reclaiming waste salts and closing the sodium and sulfur cycles.

## Abstract (web summary)

Researchers developed a two-step thermochemical method to upcycle waste sodium sulfate and charcoal into valuable sodium carbonate and sulfur. This ammonia-free, low-carbon approach provides a greener pathway for large-scale industrial manufacturing.

## Introduction

Sulfuric acid (H_2_SO_4_) and sodium-based compounds (e.g., NaOH, Na_2_CO_3_, and NaHCO_3_) are widely used across diverse industrial sectors, including chemical manufacturing, metallurgy, pharmaceuticals, and desulfurization (Supplementary Fig. 1)^1–4^. Such extensive utilization generates > 20 million tons of waste Na_2_SO_4_ annually worldwide (Supplementary Table 1)^5^. Unfortunately, only ~5% of this output is currently recycled into pure Na_2_SO_4_ or value-added products^6,7^. Consequently, at least 19 million tons of waste accumulate each year, posing substantial threats to environmental safety, resource sustainability, and public health^8–10^. Beyond its classification as waste, Na_2_SO_4_ represents an underutilized reservoir of sodium and sulfur. Despite various efforts to manage this byproduct, scalable solutions commensurate with such immense quantities remain elusive^11–13^. This highlights a critical imperative for sustainable and resource-efficient approaches to upcycle this waste stream.

Several strategies have been adopted to valorize waste Na_2_SO_4_ into industrial feedstocks, aiming to establish a sustainable “valuable product-to-Na_2_SO_4_-to-valuable product” resource loop. Existing methods primarily focus on converting SO_4_^2−^ into H_2_SO_4_ or reusable sulfates^5,14–16^. For instance, bipolar membrane electrodialysis (BMED) offers a pathway to regenerate NaOH and H_2_SO_4_ (or sulfate) solutions^5,17–19^. However, to fully realize its techno-economic potential for large-scale applications, optimization is still required regarding membrane costs (~50% of capital expenditure)^20,21^, fouling induced by impurities in waste Na_2_SO_4_^22,23^, and product concentrations (<1 M)^24,25^. Alternatively, the SSA-process produces Na_2_CO_3_, a critical raw material with world production projected to exceed 80 million tons by 2030 (Supplementary Fig. 2a, b, and Table 2)^26,27^, simultaneously yielding ammonium sulfate [(NH_4_)_2_SO_4_]^6,16,28,29^. While successfully demonstrated at the pilot scale^30^, this process currently relies on NH_3_ to drive carbonation (Supplementary Fig. 2c, d)^29^. Although emerging electrochemical NH_3_ synthesis offers a potential sustainable pathway (Supplementary Fig. 3), further enhancements in efficiency are anticipated to facilitate its large-scale deployment (Supplementary Table 3)^31^. Recent advances in anti-solvent crystallization have notably improved Na_2_SO_4_ utilization and byproduct purity, underscoring the evolving potential of the SSA-process^16^. Nevertheless, the development of a complementary NH_3_-free paradigm is essential for ensuring comprehensive industrial viability and enabling Na_2_CO_3_ production in regions lacking NH_3_ infrastructure^32,33^. Consequently, an NH_3_-free strategy that reconverts waste Na_2_SO_4_ into Na_2_CO_3_ and an easy-to-digest byproduct is urgently needed, offering a transformative solution to close both the sodium and sulfur resource loops.

Here, we report a two-step thermochemical strategy for upcycling waste Na_2_SO_4_ into Na_2_CO_3_ under NH_3_-free conditions, distinctively fixing sulfur as S_x_ rather than sulfates or sulfur oxides (SO_x_). The resulting S_x_ serves as a critical and easily storable feedstock for emerging applications in battery production and disinfectants^34,35^. In the first step, Na_2_SO_4_ is reduced by charcoal (C) to decompose sulfate ions into Na_2_S and CO_2_ (>750 °C). Subsequently, the intermediate Na_2_S reacts with self-generated CO_2_ to yield Na_2_CO_3_ and S_x_ powder by reducing the temperature (300 °C). Notably, CO_2_ production and consumption are balanced throughout the process. The products are separated via evaporation and water leaching, achieving a Na_2_CO_3_ yield of 95.35%. The reaction mechanism was elucidated through thermodynamic calculations, chemical equilibrium analysis, and experimental validation. Furthermore, comprehensive LCA and life cycle cost (LCC) analyses were conducted to evaluate the environmental footprint, energy consumption, and economic viability of this approach.

## Results

### Thermochemical deoxygenation of Na_2_SO_4_

**Fig. 1:**
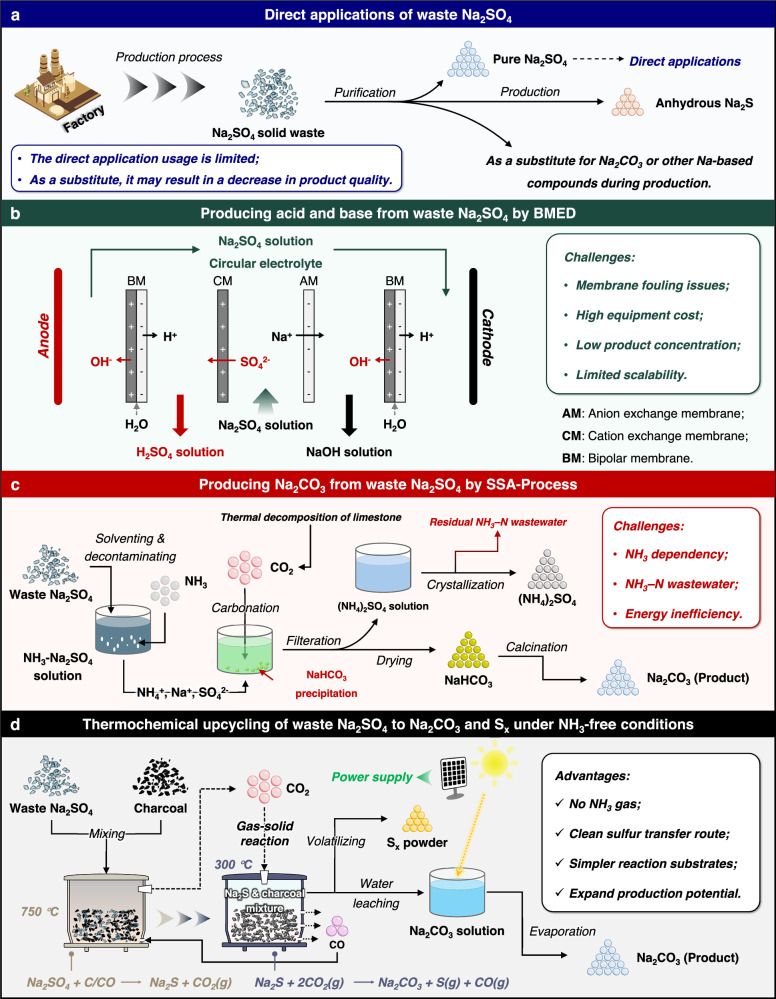
Comparison of strategies for disposing and recycling industrial waste Na_2_SO_4_. **a** Direct utilization pathways via purification or conversion to Na_2_S, which are limited by narrow application scope and product quality concerns. **b** Recovery of H_2_SO_4_ and NaOH via bipolar membrane electrodialysis (BMED). Industrial implementation is currently hindered by high membrane costs, fouling issues, and limited scalability. **c** The sodium sulfate-based ammonia-soda process (SSA-process) for producing Na_2_CO_3_ and (NH_4_)_2_SO_4_ utilizing NH_3_. This route challenges related to NH_3_ dependency, wastewater generation, and energy inefficiency. **d** The proposed NH_3_-free thermochemical upcycling strategy. This approach converts waste Na_2_SO_4_ into Na_2_CO_3_ and S_x_ through a two-step C-mediated reduction and carbonation process, offering a clean S transfer route without hazardous intermediates.

As previously discussed, current methods for disposing and recycling waste Na_2_SO_4_ involve the direct production of other low-usage products (Eq. 1), or the utilization of bipolar membranes or NH_3_ to produce acids, bases, and Na_2_CO_3_ (Fig. 1a–c). However, these approaches often result in substantial environmental burdens or rely heavily on energy-intensive NH_3_ production. BMED generates NaOH and H_2_SO_4_ by dissociating water and coupling Na^+^ and SO_4_^2−^ (Eq. 2). While the SSA-process produces the more desirable Na_2_CO_3_, implementation is constrained by its dependence on NH_3_ (Eq. 3). In contrast, the proposed two-step thermochemical upcycling process converts Na_2_SO_4_ into Na_2_CO_3_ and S_x_ under NH_3_-free conditions (Fig. 1d). This method first involves the deoxygenation of Na_2_SO_4_ followed by CO_2_ carbonation, thereby achieving NH_3_-free Na_2_CO_3_ production and providing a cleaner and more controllable route for sulfur transformation.

1 $${{{\rm{Na}}}}_{2}{{{\rm{SO}}}}_{4}+{{{\rm{C}}}/{{\rm{H}}}}_{2}{({{\rm{g}}})\to {{\rm{Na}}}}_{2}{{\rm{S}}}{+{{\rm{CO}}}}_{2}{({{\rm{g}}})/{{\rm{H}}}}_{2}{{\rm{O}}}({{\rm{g}}})\,$$

2 $${{{\rm{Na}}}}_{2}{{{\rm{SO}}}}_{4}{({{\rm{aq}}})+2{{\rm{H}}}}_{2}{{\rm{O}}}{\longrightarrow }^{{{\rm{Electricity}}}}{2{{\rm{NaOH}}}({{\rm{aq}}})+{{\rm{H}}}}_{2}{{{\rm{SO}}}}_{4}({{\rm{aq}}})$$

3 $${{{\rm{Na}}}}_{2}{{{\rm{SO}}}}_{4}+{{{\rm{CO}}}}_{2}({{\rm{g}}})+{{{\rm{H}}}}_{2}{{\rm{O}}}+{2{{\rm{NH}}}}_{3}({{\rm{g}}})\to {{{\rm{Na}}}}_{2}{{{\rm{CO}}}}_{3}+({{{{\rm{NH}}}}_{4}})_{2}{{{\rm{SO}}}}_{4}$$

**Fig. 2:**
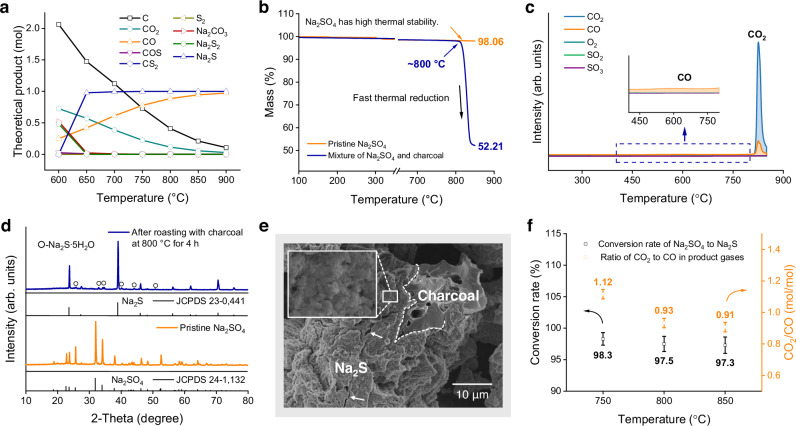
Thermochemical deoxygenation of Na_2_SO_4_. **a** Thermodynamic equilibrium analysis of the Na_2_SO_4_-C system (molar ratio: 1:4, reaction pressure: 1 atm, concentration cut-off: >10^−5^ mol, and activity: 1) derived from FactSage 8.3 calculations, predicting product distribution and phase stability across the temperature range. **b** Thermogravimetric (TG) profiles comparing the thermal decomposition behaviors of pristine Na_2_SO_4_ and the Na_2_SO_4_/C mixture (molar ratio: 1:4, heating rate: 10 °C min^−1^, atmosphere: He). **c** Mass spe**c**trometry (MS) profiles monitoring the evolution of gaseous species from the Na_2_SO_4_/C mixture. **d** XRD patterns showing the phase transformation from pristine Na_2_SO_4_ to Na_2_S after roasting. **e** Representative SEM image of the solid product reduced at 800 °C. **f** Temperature-dependent conversion rates of Na_2_SO_4_ to Na_2_S (black) and the corresponding molar ratio of evolved CO_2_/CO (orange). Error bars represent the standard deviation (SD) of *n* = 3 independent experiments.

Owing to the high thermodynamic stability and kinetic inertness of the SO_4_^2−^ oxyanion (Supplementary Fig. 4), Na_2_SO_4_ is inherently resistant to decomposition and difficult to disrupt^36^. Consequently, current methods primarily focus on converting SO_4_^2−^ into H_2_SO_4_ or other utilizable sulfate products^5^. In contrast, thermochemical deoxygenation (reduction) represents a critical strategy for processing Na_2_SO_4_, as it effectively breaks the tetrahedral structure of the SO_4_^2−^ group, leading to its collapse. Thermodynamic equilibrium calculations and Gibbs free energy (∆*G*^θ^) analyses (FactSage 8.3 and HSC Chemistry 9.0) predict the reaction products and trends for the Na_2_SO_4_-C system, as detailed in Fig. 2a and Supplementary Fig. 5. The direct carbothermal deoxygenation of Na_2_SO_4_ to Na_2_S, the thermochemical reaction of CO, and the direct formation of Na_2_CO_3_ are described by Eqs. 4–7. Thermodynamically, the reduction of hexavalent S in Na_2_SO_4_ to Na_2_S is strongly favorable, whereas the direct formation of Na_2_CO_3_ and SO_2_ is unfavorable (Supplementary Fig. 5). This selectivity is attributed to the higher thermodynamic stability of Na_2_S and the significantly greater bond dissociation energy of the C=O bond compared to the S=O bond^37,38^. Crucially, the preferential formation of Na_2_S sequesters S in a solid phase, thereby circumventing SO_x_ emissions and enabling a clean S transfer route.

4 $${{{\rm{Na}}}}_{2}{{{\rm{SO}}}}_{4}+2{{\rm{C}}}={{{\rm{Na}}}}_{2}{{\rm{S}}}+2{{\rm{C}}}{{{\rm{O}}}}_{2}({{\rm{g}}})$$

5 $${{{\rm{Na}}}}_{2}{{{\rm{SO}}}}_{4}+4{{\rm{C}}}={{{\rm{Na}}}}_{2}{{\rm{S}}}+4{{\rm{CO}}}({{\rm{g}}})$$

6 $${{{\rm{Na}}}}_{2}{{{\rm{SO}}}}_{4}+4{{\rm{CO}}}({{\rm{g}}})={{{\rm{Na}}}}_{2}{{\rm{S}}}+4{{\rm{C}}}{{{\rm{O}}}}_{2}({{\rm{g}}})$$

7 $${{{\rm{Na}}}}_{2}{{{\rm{SO}}}}_{4}+{\mbox{CO}}({{\rm{g}}})={{{\rm{Na}}}}_{2}{{{\rm{CO}}}}_{3}+{{\rm{S}}}{{{\rm{O}}}}_{2}({{\rm{g}}})$$

Experimental validation via thermogravimetric (TG) analysis of the Na_2_SO_4_/C mixture revealed a distinct mass loss onset at approximately 800 °C, culminating in a residual mass of 52.21% (Fig. 2b), which aligns closely with the theoretical value of 53.69% for reduction to Na_2_S (Supplementary Fig. 6). Mass spectrometry (MS) monitoring confirmed CO_2_ and CO as the exclusive gaseous species; the signals of SO_2_ and SO_3_ were undetected, ruling out the competitive comproportionation pathway involving SO_x_ formation (Fig. 2c and Supplementary Fig. 7). These results corroborate that the reaction proceeds via the direct reduction of Na_2_SO_4_ to Na_2_S, rather than a one-step conversion to Na_2_CO_3_. Consequently, this deoxygenation step is vital for immobilizing S as Na_2_S, thereby preventing SO_x_ emissions.

The atmospheric continuous validation involved roasting 10 g of Na_2_SO_4_ with C (molar ratio 1:4) in a tube furnace (Supplementary Fig. 8). X-ray diffraction (XRD) patterns of the product roasted at 800 °C for 4 h confirmed the dominant presence of Na_2_S, with trace Na_2_S·5H_2_O attributed to unavoidable hygroscopic hydration during ex-situ analysis (an issue easily mitigated in continuous sealed reactors) (Fig. 2d). SEM analysis revealed that the Na_2_S product exists as amorphous, cracked particles intimately adhered to the C surface, rather than forming large agglomerates (Fig. 2e). This morphology suggests a reaction mechanism mediated by a transient molten salt phase (molten Na_2_S-Na_2_SO_4_) interacting with solid C (Supplementary Fig. 9). The excess C not only ensured the complete reduction of Na_2_SO_4_ but also provided a surface for Na_2_S to deposit, which is beneficial for the subsequent Na_2_S-CO_2_ reaction by enhancing the interface. Furthermore, kinetic studies of the Na_2_SO_4_-to-Na_2_S transformation revealed that the reaction initiates as low as 750 °C (Supplementary Fig. 10), despite the negligible mass loss observed in TG at this temperature. This discrepancy is attributed to sluggish reaction kinetics at lower temperatures, which may not be fully captured under the dynamic heating conditions of TG. Quantitative analysis of sulfide ion (S^2−^) yield indicated conversion efficiencies exceeding 97% across the 750–850 °C range (Fig. 2f and Supplementary Fig. 11). However, a slight decline in yield at elevated temperatures suggests minor volatilization of the Na_2_S product. C effectively reduced Na_2_SO_4_ to Na_2_S without releasing SO_x_, and the resulting Na_2_S was porous, which is advantageous for the second step of the thermochemical reaction (i.e., the formation of Na_2_CO_3_). The CO_2_/CO ratio in the effluent gas decreased with increasing temperature, indicating that the Boudouard reaction (C + CO_2_ → 2CO) becomes more prominent at higher temperatures and thus a lower carbon-utilization efficiency. Consistently, C consumption exceeded theoretical stoichiometry (142.35–146.87%) as the temperature rose (Supplementary Fig. 12). Additionally, the feasibility of using hydrogen (H_2_) as a reducing agent was confirmed above 750 °C (Supplementary Fig. 13). As green H_2_ costs decline, this presents a viable, ultra-clean alternative for the valorization of waste Na_2_SO_4_.

### Carbonation of Na_2_S in a CO_2_ atmosphere

**Fig. 3:**
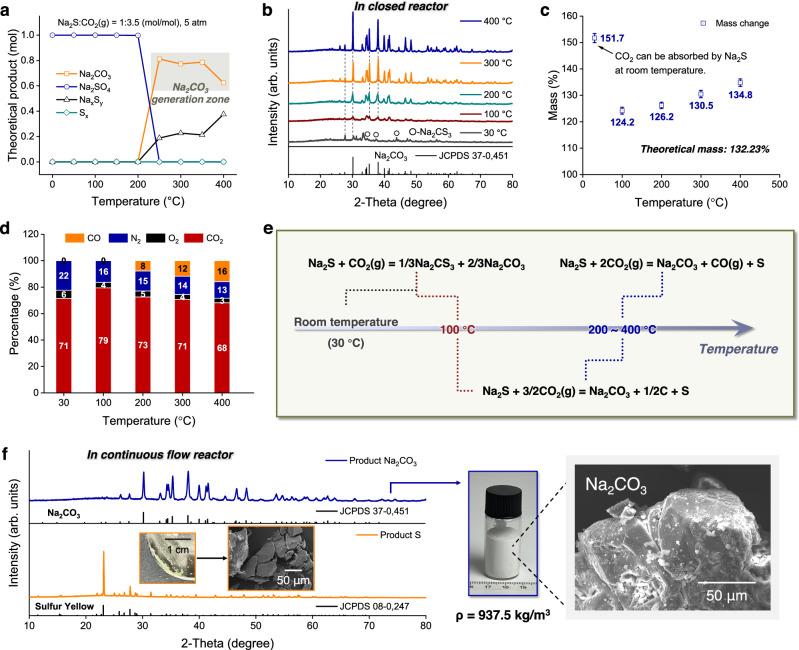
Thermochemical carbonation of Na_2_S in a CO_2_ atmosphere. **a** Thermodynamic equilibrium analysis of the Na_2_S-CO_2_ system (molar ratio: 1:3.5, reaction pressure: 5 atm, concentration cut-off: >10^−5^ mol, activity: 1), predicting the temperature window for Na_2_CO_3_ generation. XRD patterns (**b**) and mass changes (**c**) of solid products at different temperatures in the closed steel reactor. Error bars represent the SD of *n* = 3 independent experiments. **d** Compositional analysis of the gaseous byproducts at different temperatures, highlighting the generation of CO. **e** Schematic illustration of the proposed reaction pathways of Na_2_S-CO_2_ across different temperature zones. **f** Characterization of products from a continuous flow reactor, including XRD patterns of the purified Na_2_CO_3_ and recovered S_x_, along with SEM images.

Thermodynamic equilibrium calculations predict that the conversion of Na_2_S to Na_2_CO_3_ is energetically favorable within a specific temperature window of 250–350 °C, a region characterized by multiple competing reactions pathways (Fig. 3a and Supplementary Fig. 14). To validate these theoretical predictions and elucidate the underlying mechanism, the interaction between Na_2_S and CO_2_ was systematically investigated using a closed steel reactor (Supplementary Fig. 15). XRD results revealed that with increasing temperature, the product transitioned from a Na_2_CO_3_-Na_2_CS_3_ mixture towards pure Na_2_CO_3_, with the Na_2_CO_3_ crystalline phase becoming more pronounced (Fig. 3b). Above 200 °C, a small amount of S_x_ powder was collected from the reactor walls. Mass changes in the solid phase and pressure variations in the gas phase indicated that the Na_2_S-CO_2_ reaction occurred between 30 and 400 °C, exhibiting distinct mechanisms at different temperatures (Fig. 3c and Supplementary Fig. 16). Notably, at room temperature (RT), the product mass increase reached 151.7%, significantly higher than the theoretical 132.23% for Na_2_CO_3_ formation from Na_2_S. This suggests that the process initiates with the deep chemisorption of CO_2_ to form heavier sulfur-carbon intermediates (e.g., Na_2_CS_3_). Conversely, elevated temperatures overcome kinetic barriers and drive the decomposition of these intermediates into thermodynamically stable Na_2_CO_3_ and volatile S_x_.

Gas-phase compositional analysis revealed that the proportion of CO increased with temperature (Fig. 3d). Given that the Boudouard reaction is thermodynamically suppressed below 700 °C^39^, the observed CO generation is attributed exclusively to the redox reaction between Na_2_S and CO_2_. In this pathway, S^2−^ function as electron donors, reducing CO_2_ while undergoing oxidation to S_x_. This interpretation is fully consistent with thermodynamic models of the Na_2_S-CO_2_ system (Supplementary Fig. 14).

Further experiments revealed that roasting solid reaction products in air at 600 °C resulted in a mixture of Na_2_SO_4_ and Na_2_CO_3_ (Na_4_CO_3_SO_4_) (Supplementary Fig. 17). No significant changes were observed in the products above 200 °C, which aligned with equilibrium calculations, indicating that Na_2_CO_3_ is thermodynamically favored at higher temperatures. The TG of Na_2_S in CO_2_ further indicated that CO_2_ absorption occurred at low temperatures (Supplementary Fig. 18). The mass change did not reach 151.7%, which was likely attributed to the mismatch between the heating rate and the absorption kinetics. In summary, the Na_2_S-CO_2_ reaction proceeds follow (Fig. 3e): at RT, CO_2_ was fully absorbed and converted into Na_2_CO_3_ and Na_2_CS_3_, while at higher temperatures, the reaction between CO_2_ and Na_2_S to form Na_2_CO_3_ became kinetically favorable. The volatilization of S_x_ also enhanced the reaction forward progression.

In a continuous flow reactor, complete conversion of Na_2_SO_4_ to Na_2_CO_3_ was achieved (Supplementary Fig. 19). After S_x_ and C removal, the resulting Na_2_CO_3_ appeared white, with a bulk density of 937.5 kg m^−3^ (classified as dense soda ash) (Fig. 3f), which satisfies the industrial specifications for desulfurization applications. CO was detected within 20 min after switching to CO_2_ atmosphere, confirming the electron transfer from S^2−^ to CO_2_ (Supplementary Fig. 20). S_x_ products were collected and identified as yellow S_x_ powder (Fig. 3f). The efficient product separation is attributed to the synergistic effects of S_x_ volatility during roasting and the stark solubility difference between hydrophobic S_x_ and hydrophilic Na_2_CO_3_ in aqueous media (Supplementary Figs. 21–23). Consequently, thermochemical deoxygenation and carbonation offer an effective strategy for disposing of industrial Na_2_SO_4_ waste.

### Transformation mechanism from Na_2_SO_4_ to Na_2_CO_3_ and S_x_

**Fig. 4:**
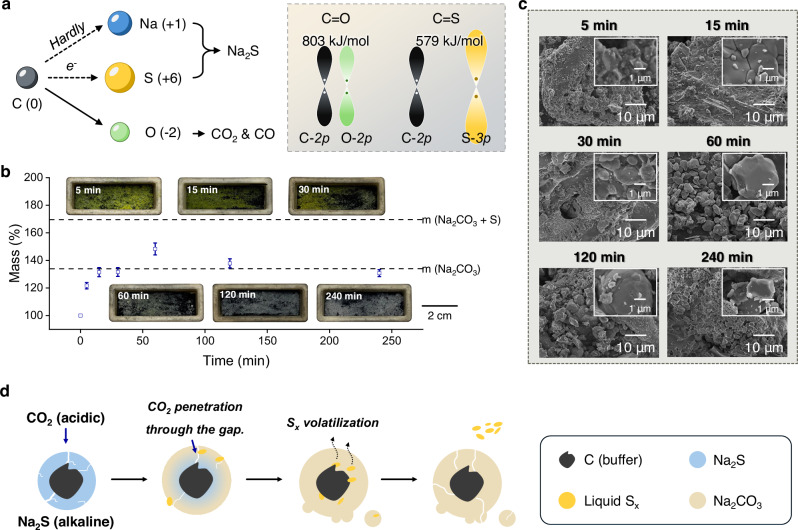
Transformation mechanism from Na_2_SO_4_ to Na_2_CO_3_ and S_x_. **a** Schematic of the selective reduction mechanism of Na_2_SO_4_ to Na_2_S, driven by the bond dissociation energy difference between C=O and C=S bonds. **b** Time-resolved mass variation profile during the carbonation of the Na_2_S intermediate, with optical insets tracking the macroscopic color transition from yellow to white. Error bars represent the SD of *n* = 3 independent experiments. **c** SEM images revealing the morphological evolution of the solid products at different reaction stages (5–240 min). **d** Proposed reaction model illustrating the CO_2_ diffusion-controlled carbonation process and simultaneous S_x_ volatilization on the C buffer.

The carbothermal reduction of Na_2_SO_4_ to Na_2_S was experimentally validated, confirming a highly selective deoxygenation pathway with zero SO_x_ emissions. This selectivity is fundamentally governed by atomic-level electronic properties and bond dissociation energies. In the Na_2_SO_4_-C system, the Na (+1) in Na_2_SO_4_ is difficult to reduce and typically requires very high temperatures (above 1200 °C) and low vapor pressure to induce Na volatilization (Fig. 4a)^40^. Conversely, the S (+6) in the sulfate tetrahedron serves as the primary electron acceptor. As carbon donates electrons, the oxygen atoms are stripped away to form stable C=O bonds. The thermodynamic driving force for this specific pathway stems from the high bond dissociation energy of C=O (803 kJ mol^−1^ in CO_2_), which is significantly more stable than the C=S bond (~579 kJ mol^−1^ in CS_2_) (Supplementary Fig. 24)^41,42^. Consequently, the formation of volatile SO_x_ or CS_2_ is thermodynamically suppressed, directing the reaction exclusively toward the formation of solid Na_2_S and gaseous CO_x_.

The temporal evolution of the subsequent carbonation step (Na_2_S-to-Na_2_CO_3_) was elucidated through time-resolved mass and microstructural analyses (Fig. 4b). A rapid mass gain of 121.66% was recorded within the first 5 min, visually accompanied by the emergence of yellow S_x_ powder on the sample surface. XRD patterns identified a biphasic mixture of Na_2_CO_3_ and residual Na_2_S, with no other crystalline intermediates detected (Supplementary Fig. 25a). The mass profile reflects CO_2_ absorption by Na_2_S and its conversion to Na_2_CO_3_, accompanied by the formation and volatilization of liquid S_x_. The mass peaked at 148.34% after 60 min, signaling the near-complete transformation of the sulfide phase into carbonate. However, trace amounts of S_x_ powder remained on the surface. Subsequent mass loss was attributed to the volatilization of S_x_. Microstructural evolution provided further insights into this solid-gas reaction mechanism. SEM imaging at 5 min revealed a molten-like surface morphology, suggesting the fast formation of liquid S_x_ upon the oxidation of S (−2) (Fig. 4c). As the reaction progressed, significant particulate growth and crystallization were observed, driven by the volumetric expansion associated with CO_2_ absorption. By 60 min, the distinct Na_2_CO_3_ crystals (3–5 μm) had formed (Supplementary Fig. 25b). Crucially, the subsequent appearance of surface cracks and fragmentation is attributed to the internal pressure exerted by volatilizing S_x_ species escaping from the interior. As the particles started to show cracks, this was attributed to the pressure from gaseous S_x_, causing fragmentation and release. Between 120 and 240 min, the continued release of trapped S_x_ caused the mass to stabilize near the theoretical value for pure Na_2_CO_3_, leaving behind particles with irregular, porous morphologies. Mechanistically, this transformation is driven by a strong acid-base neutralization reaction, where acidic CO_2_ reacts readily with alkaline Na_2_S within the thermodynamically favorable window of 200–400 °C. The C buffer plays a pivotal role here, preventing sintering and providing a high surface area for efficient gas-solid contact (Fig. 4d). To rigorously assess potential pollutant formation, the gas composition at 350 °C was analyzed by gas chromatography-mass spectrometry (GC-MS) (Supplementary Fig. 26). Crucially, the analysis confirmed the absence of detectable SO_x_, validating the kinetic and thermodynamic inhibition of SO_x_ generation in this system. The product gas consisted almost exclusively of CO_2_ (carrier gas) and CO (reduction byproduct), with only negligible traces of carbonyl sulfide (COS) observed. In summary, this NH_3_-free thermochemical strategy achieves the clean, site-independent upcycling of waste Na_2_SO_4_ into high-value Na_2_CO_3_, successfully overcoming the scalability and pollution constraints inherent in conventional precipitation-based routes.

### Scale-up production, LCA, and economic analysis

**Fig. 5:**
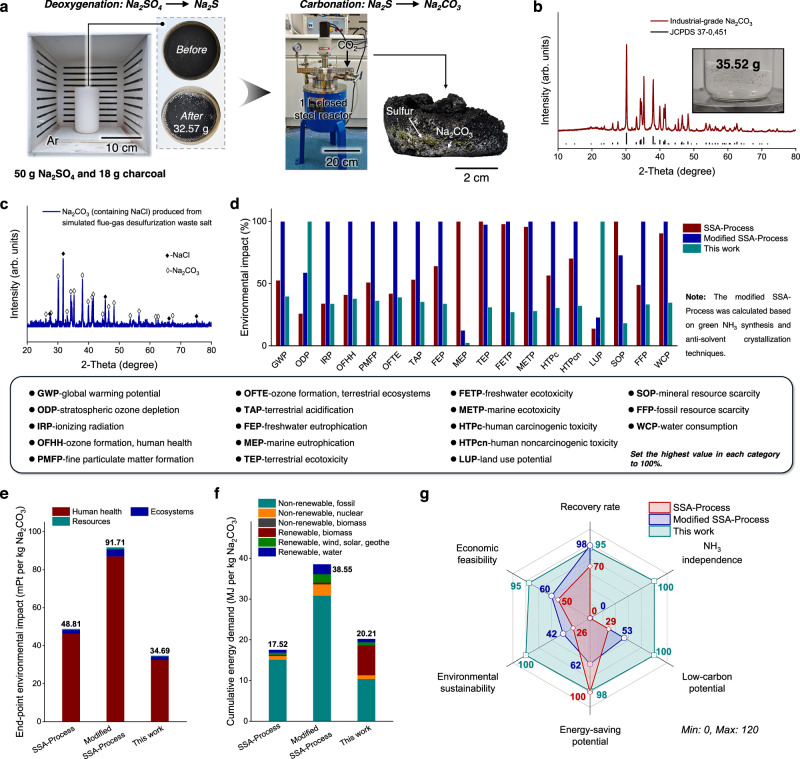
Scale-up production, LCA, and economic analysis. **a** Workflow of the upscaled production using 50 g of waste Na_2_SO_4_ and 18 g of C, illustrating the transition from deoxygenation to carbonation. **b** XRD pattern of the purified Na_2_CO_3_ obtained from the scale-up experiment. The inset photograph displays the final collected white powder (35.52 g). **c** XRD pattern of the product synthesized from simulated flue-gas desulfurization waste salt, demonstrating the tolerance of this work to NaCl impurities. Comparative LCA results among the SSA-process, modified SSA-process, and the method developed in this work: **d** mid-point environmental impact categories; **e** end-point environmental impact aggregated into human health, ecosystems, and resources; **f** cumulative energy demand (CED). **g** Radar chart comparing multidimensional performance metrics, including economic feasibility, recovery rate, NH_3_ independence, low-carbon potential, energy-saving potential, and environmental sustainability. Raw data for each criterion were normalized to a 0–100 scale, where 100 represents the optimal theoretical or best-observed performance. Detailed raw values and normalization formulas are provided in Supplementary Note 2.

The primary objective of this technological development is the clean and low-carbon scale-up of converting waste Na_2_SO_4_ into value-added Na_2_CO_3_. Accordingly, an upscaled production process was carried out (Fig. 5a). EDS analysis characterized the intermediate powder as a mixture of Na_2_CO_3_ and S_x_ (Supplementary Fig. 27). After purification yielded 35.52 g of white powder (Supplementary Fig. 28), XRD analysis confirmed the isolation of pure Na_2_CO_3_ with no detectable impurity peaks (Fig. 5b). The process achieved a yield of 95.35% relative to theoretical value (with further gains expected upon scale-up) and demonstrated a product purity exceeding 99.53%, containing only trace S and Si impurities originating from the glassware (Table 1). These minor impurities are likely mitigable by upgrading to industrial-scale equipment with superior corrosion resistance. Significantly, the presence of NaCl in common mixed waste did not interfere with the thermochemical conversion of Na_2_SO_4_ (Fig. 5c and Supplementary Table 4). The resulting NaCl-Na_2_CO_3_ mixture enables diverse downstream applications, including CO_2_ capture-conversion, electrolytic extraction of Na alloys, or desulfurization processes^43,44^.

**Table 1:** X-ray fluorescence (XRF) analysis of the purified Na_2_CO_3_ product from scale-up production

Element	Na	O	C	S	Si	K	Ca	Total
Weight (%)	43.6152	42.8332	13.0906	0.2532	0.1656	0.0310	0.0112	100.0000

To benchmark sustainability against the traditional SSA-process and the modified SSA-process (integrated with green NH_3_ synthesis and anti-solvent crystallization)^16,45^, we conducted a cradle-to-gate LCA based on a functional unit of 1 kg of dense Na_2_CO_3_ (Supplementary Tables 5–7 and Fig. 29). Environmental impact assessments reveal that our route offers distinctive advantages in global warming potential (GWP), water consumption, and human health. Notably, water consumption decreased by at least 55.7% (Fig. 5d and Supplementary Table 8), a benefit attributed to the anhydrous thermochemical nature of this work, which circumvents the water-intensive liquid-phase carbonation required by the SSA processes. Specifically, the GWP is reduced by > 0.43 kg CO_2_-eq per kg Na_2_CO_3_ produced. Conversely, the reliance on NH_3_ in competing processes introduces significant risks of aquatic eutrophication and ecotoxicity via potential nitrogen leakage. More critically, the carcinogenic and non-carcinogenic toxicity associated with NH_3_ presents a substantial barrier to the high-value upcycling of Na_2_SO_4_ via SSA routes. Normalized results highlight the superior human health performance of our approach, confirming its environmental cleanliness (Supplementary Fig. 30). Consequently, the total end-point environmental impact of this work (34.69 mPt per kg Na_2_CO_3_) is significantly lower than that of competitive routes (Fig. 5e and Supplementary Table 9). Although the cumulative energy demand (CED) appears marginally higher than the SSA-process (Fig. 5f and Supplementary Table 10), contribution analysis clarifies a critical distinction: a substantial fraction of this demand is derived from renewable biomass (charcoal) rather than fossil fuels (Supplementary Figs. 31–33). Furthermore, energy-balance analysis further indicates that 52.7% of the total energy is associated with dehydration and evaporation, processes that are highly adaptable to intermittent clean thermal sources (e.g., solar thermal energy or industrial waste heat) rather than grid electricity (Supplementary Fig. 34). Under a projected PV-driven scenario, our technology maintains the lowest environmental footprint while achieving a competitive CED (Supplementary Fig. 36). We should point out that the energy-consumption calculation is based on lab-scale experiments, which may have some deviation from large-scale applications (Supplementary Data 1). Overall, our technology demonstrates exceptional potential across multiple sustainability metrics (Fig. 5g and Supplementary Note 2), with the observed gap in energy-saving potential likely reflecting the current maturity of industrial NH_3_ synthesis infrastructure compared to emerging thermochemical routes.

The economic evaluation was broadened to include BMED as a comparative benchmark via a detailed LCC analysis (Supplementary Methods and Tables 11, 12). The proposed route demonstrates superior profitability, yielding a net profit of +22.94 USD per ton of waste Na_2_SO_4_·10H_2_O (rising to +59.50 USD under PV-driven scenarios), representing a profit margin of 19.89% (51.59%) (Table 2). Crucially, sensitivity analysis reveals high economic robustness. Our route becomes the most cost-effective option when electricity costs exceed $0.048 per kWh and shows minimal sensitivity to electricity price increases (Supplementary Fig. 37). Additionally, our process exhibits higher market stability because soda ash contributes 84% of total product revenue (Supplementary Fig. 38), whereas the modified SSA-process remains vulnerable to supply-demand fluctuations in the (NH_4_)_2_SO_4_ market.

**Table 2:** Summary of economic analysis (fundamental unit: 1 ton of waste Na_2_SO_4_·10H_2_O)

Parameter	BMED	Modified SSA-process	This work
**1. Costs (USD)**			
Electricity	439.90 (189.16)	219.47 (94.37)	54.51 (23.44)
Other materials (reagents and water)	13.53	22.78	24.01
Fixed costs (Sum of maintenance, labor, etc.)	80.02 (35.77)	42.75 (20.67)	13.86 (8.37)
Total LCC	533.45 (238.46)	285.00 (137.82)	92.38 (55.82)
**2. Revenue (USD)**			
Total revenue	147.42	206.83	115.32
(Main products)	NaOH, H_2_SO_4_	Na_2_CO_3_	Na_2_CO_3_
(Byproducts)	–	(NH_4_)_2_SO_4_	S_x_
**3. Profitability**			
Net profit (USD)	−386.03 (−91.04)	−78.17 (+69.01)	+22.94 (+59.50)
Profit margin (%)	−261.86% (−61.76%)	−37.79% (+33.36%)	+19.89% (+51.59%)

Values in parentheses denote the scenario using renewable solar (PV) electricity (0.043 USD per kWh^45^) rather than standard grid electricity (0.10 USD per kWh). The total LCC encompasses variable operating expenditures (OPEX, including electricity and other materials) and fixed costs (estimated at 15% of the total LCC). For detailed calculation procedures and comprehensive data breakdowns, please refer to Supplementary Methods and Supplementary Table 12.

Beyond process-level metrics, this work has broader implications for waste management and circular resource use. We estimate that unmanaged stockpiling of global waste Na_2_SO_4_ would squander ~1864 acres of land annually (Supplementary Fig. 39). To demonstrate circularity, we tested a closed-loop application in Li_2_CO_3_ extraction by coupling a solar concentrator for dehydration with carbonate reagent regeneration (Supplementary Fig. 40). Furthermore, we developed a general “alkaline sodiation” strategy to extend this protocol to other complex sulfate wastes, effectively preventing hazardous SO_x_ emissions (Supplementary Fig. 41). Looking forward, we envision three application scenarios: (i) decentralized Na_2_SO_4_ upcycling in regions lacking NH_3_ infrastructure, (ii) integrated on-site production-consumption loops within industrial parks, and (iii) a carbon-negative pathway coupling green H_2_ deoxygenation with direct air CO_2_ capture (Supplementary Fig. 42).

## Discussion

In this work, we demonstrate a two-step thermochemical route to upcycle waste Na_2_SO_4_ to Na_2_CO_3_ and S_x_. The process leverages temperature modulation, in which carbon disrupts sulfate structures at 750 °C, followed by a shift to sulfide carbonation at 300 °C to achieve a Na_2_CO_3_ yield of 95.35%. Environmentally, this anhydrous route circumvents the water-intensive liquid-phase carbonation and eliminates the NH_3_ use required in the conventional SSA process. Consequently, it reduces GWP by > 0.43 kg CO_2_-eq per kg Na_2_CO_3_ while exhibiting superior economic stability against electricity price fluctuations (>$0.048 per kWh). Moreover, this technology demonstrates the lowest end-point environmental impact (34.69 mPt per kg Na_2_CO_3_) among the evaluated methods. On a broader scale, this approach enables decentralized deployment in resource-limited regions lacking NH_3_ synthesis infrastructure and facilitates the integration of diverse sulfate waste recycling streams. Looking forward, the future integration of low-carbon deoxygenation and CO_2_ capture could potentially transform this technology into a carbon-negative solution. This positions waste Na_2_SO_4_ as a critical vector for closing sodium and sulfur cycles and achieving deep decarbonization.

## Methods

### Materials

Na_2_SO_4_ (analytical reagent (AR) grade, ≥99.8%), anhydrous Na_2_S (AR, ≥99.9%), and NaCl (AR, ≥99.8%) were procured from Sinopharm Chemical Reagent Co., Ltd. (Shanghai, China). Before use, Na_2_SO_4_ and other hygroscopic salts were vacuum-dried at 300 °C for 12 h to ensure moisture removal. Charcoal (C), derived from the carbonization of cherry wood, was obtained from a local supplier in Wuhan (Supplementary Fig. 43). High-purity Ar (99.99%) and CO_2_ (99.99%) were employed, while H_2_ was supplied as a gas mixture of 5 mol% H_2_ in Ar. Alumina crucibles were purchased from Changsha Miqi Instrument Co., Ltd. (Hunan, China).

### Thermochemical deoxygenation of Na_2_SO_4_

Caution: highly toxic CO gas is evolved during this reduction process. All exhaust gases must be properly vented into a designated fume hood. Pre-dried Na_2_SO_4_ (10 g, 70.4 mmol) and C (3.39 g, 281.6 mmol) were mixed in a 1:4 molar ratio and ball-milled at 400 rpm for 30 min. The mixture was subsequently loaded into an alumina crucible and heat-treated in a tube furnace under an Ar atmosphere at temperatures ranging from 700 to 850 °C (heating rate: 10 °C min^−1^). During this process, evolved gases were collected and analyzed to monitor the CO_2_/CO ratio for optimization of reducing conditions. Upon cooling to RT, the resulting solid intermediate was collected. The Na_2_S yield in the solid product was quantified via UV-Vis spectroscopy, based on the calibration curve of S^2−^ absorbance at 235 nm (Supplementary Fig. 11). The conversion efficiency was calculated using Eq. 8. For comparison, the H_2_-thermal deoxygenation of Na_2_SO_4_ (4 g, 140.8 mmol) was conducted under a flowing H_2_-Ar atmosphere (40 mL min^−1^, 8 h) using a similar setup.

8 $${{\rm{Conversion}}}\; {{\rm{rate}}}=\frac{{{\rm{moles}}}\; {{\rm{of}}}\,{{{\rm{S}}}}^{2-}{{\rm{in}}}\; {{\rm{the}}}\; {{\rm{product}}}}{{{\rm{moles}}}\; {{\rm{of}}}{{{\rm{Na}}}}_{2}{{{\rm{SO}}}}_{4}{{\rm{of}}}\; {{\rm{the}}}\; {{\rm{mixture}}}}\times 100\%$$

### Carbonation of Na_2_S in a CO_2_ atmosphere

Caution: anhydrous Na_2_S is highly reactive with moisture and generates toxic hydrogen sulfide (H_2_S) gas. All handling of Na_2_S must be strictly performed in an Ar-filled glovebox. To evaluate reaction feasibility and process continuity, the reaction between Na_2_S and CO_2_ was investigated using both a closed steel reactor and a continuous flow reactor.

### Carbonation of Na_2_S in a closed reactor

The deoxygenated C-containing Na_2_S intermediate (0.5 g, ~6.4 mmol) was loaded into a 100 mL closed steel reactor (Supplementary Fig. 15). This operation was performed inside an Ar-filled glovebox (O_2_ < 0.1 ppm, H_2_O < 0.1 ppm) to strictly exclude moisture. The reactor, equipped with a real-time pressure sensor, was purged three times with CO_2_ before being pressurized to 0.5 MPa. Subsequently, the system was heated to target temperatures ranging from 30 to 400 °C (heating rate: 10 °C min^−1^) and maintained for 8 h. Upon completion, the reactor was naturally cooled to RT. Both the solid components and gaseous products were collected for compositional analysis to elucidate the reaction pathway.

### Continuous carbonation in a flow reactor

A two-step thermochemical process was conducted in a tube furnace to simulate continuous production. Initially, the thermal deoxygenation of Na_2_SO_4_/C mixture (Na_2_SO_4_: 10 g, 70.4 mmol; C: 3.39 g, 281.6 mmol) was performed under Ar flow at 800 °C for 4 h. The temperature was then lowered to 300 °C at a rate of 10 °C min^−1^, at which point the gas stream was switched to CO_2_ (100 mL min^−1^). The system was maintained at 300 °C for an additional 4 h to facilitate carbonation. Finally, the reactor was naturally cooled to RT, and the resulting solid products and effluent gases were collected for characterization.

### Mechanism and scale-up

Following the reduction protocol outlined in the preceding section, the Na_2_SO_4_/C mixture was deoxygenated at 800 °C. Upon cooling to 350 °C, the gas flow was switched to CO_2_, and the carbonation duration was varied (5, 15, 30, 60, 120, and 240 min) to investigate the reaction mechanism. The resulting solid products were characterized via XRD, SEM, and mass change analysis.

Caution: the scale-up process involves high-pressure equipment (1.5 MPa). Appropriate safety shields and pressure relief protocols must be employed. To evaluate the scalability of the proposed strategy, a scaled-up experiment was conducted. The precursor mixture comprising Na_2_SO_4_ (50.00 g, 352.0 mmol) and C (16.95 g, 1408.0 mmol) (1:4 molar ratio) was ball-milled (400 rpm, 30 min) and subjected to thermochemical reduction in a muffle furnace under an Ar atmosphere at 800 °C for 4 h. The resulting intermediate was rapidly transferred into a 1 L high-pressure reactor to minimize air exposure. Carbonation was subsequently initiated by introducing CO_2_ at a pressure of 1.5 MPa to convert Na_2_S into Na_2_CO_3_ and S_x_. Finally, the products were separated via aqueous leaching in DI water at 40 °C, allowing for the isolation of Na_2_CO_3_, S_x_, and residual C.

### Product prediction and thermodynamic analysis

Thermodynamic equilibrium compositions for both the Na_2_SO_4_-C deoxygenation and Na_2_S-CO_2_ carbonation systems were simulated using FactSage 8.3 across relevant temperature ranges. Complementary standard Gibbs free energy (Δ*G*^θ^) calculations for specific reaction pathways were performed using HSC Chemistry 9.0. For all thermodynamic models, solid and liquid species were assumed to be pure phases with unit activity (activity = 1), and gaseous species were evaluated at standard atmospheric pressure.

### LCA and LCC analysis

LCA followed the ISO 14040 and 14044 standards^46^. The functional unit was defined as the production of 1 kg of dense Na_2_CO_3_, to compare the environmental impacts of this process with those of the current and green NH_3_-driven soda ash production methods^47^. The system boundaries included the inputs (energy and materials) and outputs (wastewater, waste gases, and waste) during the Na_2_CO_3_ production process, excluding the production and maintenance of equipment. Supplementary Table 5 presents a detailed life cycle inventory (LCI) for Na_2_CO_3_ production in this process. The LCI for the compared Na_2_CO_3_ production method was based on the global production model of the current SSA-process from the Ecoinvent database^48^. The “modified SSA-process” was modeled by modifying the baseline SSA-process to integrate green NH_3_ synthesis and anti-solvent crystallization technologies. Geographical assumptions for electricity and fuel supply were based on the Chinese scenario. Considering the byproduct generation in this process, the economic allocation method was used for distributing environmental burdens and credits. The environmental impacts of the Na_2_CO_3_ production process were assessed using the ReCiPe 2016 method, covering both mid-point and end-point environmental impact categories^49^. Additionally, the CED of the Na_2_CO_3_ production process was evaluated. A Monte Carlo simulation was performed to assess data uncertainty^50^. The detailed procedures for both LCA and LCC can be found in the Supplementary Methods.

### Characterization

The phase composition of the samples was determined by XRD using a Shimadzu X-ray 6000 diffractometer with Cu Kα radiation (*λ* = 1.5405 Å) operating at 40 kV and 40 mA, with a scan rate of 10° min^−1^ over a 2θ range of 10°–80°. Sample morphology and elemental distribution were examined using field emission scanning electron microscopy (FE-SEM, FEI Quanta FEG 250). Prior to imaging, the nonconductive powder samples were dispersed on conductive carbon tape and sputter-coated with a thin layer of gold for 120 s to prevent charging artifacts. Images were acquired using a secondary electron (SE) detector at an accelerating voltage of 5.0 kV. The elemental mapping was conducted utilizing an energy-dispersive X-ray spectroscopy (EDS) detector (Aztec Energy) at an accelerating voltage of 15.0 kV. To ensure high signal-to-noise ratios, the mapping acquisition for each selected region was performed for approximately 50 s with an average count rate of ~5000 cps (counts per second), yielding over 30,000 total accumulated counts.

Thermal behavior and evolved gas analysis were performed simultaneously using a TG-GC-MS system (PerkinElmer TGA 8000 coupled with SQ8T). Approximately 10 mg of sample was loaded into an alumina pan and heated from 30 to 800 °C at a rate of 10 °C min^−1^ under a He atmosphere with a flow rate of 40 mL min^−1^. The mass selective detector’s electron impact (EI) ion source temperature was set at 200 °C, and the mass spectra were analyzed in the range of 0–300 atomic mass units (amu).

S^2−^ concentration was quantified via UV-Vis spectrophotometry (TU-1901). For sampling, 0.5 g of the solid product was dissolved in 50 mL of 0.1 M NaOH solution. The solution was then filtered and diluted. A standard calibration curve was established using standard S^2−^ solutions with known concentrations ranging from 0 to 4.0 mg L^−1^. The absorbance of the test solutions was measured at 235 nm and interpolated against the linear calibration plot (*R*^2^ = 0.998, Supplementary Fig. 11). The composition of gaseous products (e.g., CO_2_, CO) was analyzed using GC (Fuli Instruments GC9790II) equipped with a GDX-501 packed column and a thermal conductivity detector (TCD). The carrier gas was Ar at a flow rate of 30 mL min^−1^. The temperatures of the injector, oven, and detector were maintained at 120, 80, and 130 °C, respectively. The GC system was calibrated prior to measurement using certified standard gas mixtures containing known concentrations of CO, CO_2_, and H_2_. Additionally, elemental purity was assessed by XRF (Rigaku ZSX Primus) operated at 30 kV, and optical images were captured using a digital camera (Canon, 600D).

### Reporting summary

Further information on research design is available in the Nature Portfolio Reporting Summary linked to this article.

## Supplementary information

Supplementary Information

Description of Additional Supplementary File

Supplementary Data 1

Reporting Summary

Transparent Peer Review File

## Source data

Source data

## Data Availability

The data supporting the findings of this study are available in the article and Supplementary Information files or available from the corresponding authors upon request. Source data are provided with this paper.
