# Supplementary material for: Upcycling of waste sodium sulfate to sodium carbonate and sulfur
Source: Nat Commun. 2026 Apr 24;17:5503. doi: 10.1038/s41467-026-72286-y (PMC13287641; doi:10.1038/s41467-026-72286-y)
Supplement: Supplementary file 1 — Supplementary Information [file 41467_2026_72286_MOESM1_ESM.pdf]

Supplementary Information

Upcycling of waste sodium sulfate to sodium carbonate and sulfur

*Hongya Wang[1], Shiyu Wang[1], Fengyin Zhou[1], Muya Cai[1], Jingjing Zhao[1], Danfeng Wang[1], Zhan Shi[2], Xiang Chen[1], Dihua Wang[1,3,4], Huayi Yin[1,3,4]**

1. School of Resource and Environmental Sciences, Wuhan University, 299 Bayi Road, Wuchang District, Wuhan 430072, P. R. China.
2. Department of Land, Environment, Agriculture and Forestry, University of Padova, Legnaro (PD) 35020, Italy.
3. Hubei International Scientific and Technological Cooperation Base of Sustainable Resources and Energy, Wuhan University, Wuhan 430072, P. R. China.
4. Joint Center of Green Manufacturing of Energy Storage Materials of Wuhan University and Chilwee, Wuhan 430072, P. R. China.

*-Corresponding author. Email address: yinhuayi@whu.edu.cn (Huayi Yin)

Contents

- **Supplementary Table 1.** Inventory of Na_2SO_4 waste generation across key industrial sectors.
- **Supplementary Table 2.** Inventory of Na_2CO_3 utilization across various industrial sectors.
- **Supplementary Table 3.** Summary of state-of-the-art developments in green NH_3 synthesis.
- **Supplementary Table 4.** Chemical composition of desulfurization waste determined by XRF.
- **Supplementary Table 5.** Life cycle inventory (LCI) for Na_2CO_3 production via thermochemical upcycling of waste Na_2SO_4 designed with a continuous recycling loop.
- **Supplementary Table 6.** Design parameters of enlarged furnace in LCA calculation of Na_2CO_3 production.
- **Supplementary Table 7.** LCI data for Na_2CO_3 production via the modified SSA-Process integrating electrochemical NH_3 synthesis and anti-solvent crystallization.
- **Supplementary Table 8.** Mid-point environmental impact assessment results of the Na_2CO_3 production process.
- **Supplementary Table 9.** End-point environmental impact assessment results of the Na_2CO_3 production process.
- **Supplementary Table 10.** Cumulative energy demand (CED) of the Na_2CO_3 production process.
- **Supplementary Table 11.** LCI for the production of 68% H_2SO_4 and 50% NaOH solutions via bipolar membrane electrodialysis (BMED).
- **Supplementary Table 12.** Summary of life cycle costs (LCC) for BMED (yielding H_2SO_4 and NaOH), the modified SSA-Process, and this work.
- **Supplementary Note 1.** Detailed calculation procedures for energy consumption.
- **Supplementary Note 2.** Definition and calculation of multidimensional performance metrics for Manuscript Fig. 5g.
- **Supplementary Methods.** Life cycle assessment (LCA) and LCC assessment methodology.
- **Supplementary Fig. 1.** Generation of Na_2SO_4 waste in major industrial processes.
- **Supplementary Fig. 2.** Market dynamics and conventional production pathways of Na_2CO_3.
- **Supplementary Fig. 3.** Schematic representation of emerging alternative NH_3 synthesis pathways.
- **Supplementary Fig. 4.** Molecular geometry and structural configuration of the SO_4^{2-}.
- **Supplementary Fig. 5.** Thermodynamic feasibility of carbothermal deoxygenation pathways for Na_2SO_4.
- **Supplementary Fig. 6.** Theoretical solid mass retention calculation for the carbothermal deoxygenation of Na_2SO_4.
- **Supplementary Fig. 7.** Thermodynamic unfavourability of volatile sulfur species evolution during carbothermal reduction.

- **Supplementary Fig. 8.** Experimental setup and reaction scheme for the carbothermal deoxygenation of Na_2SO_4.
- **Supplementary Fig. 9.** Phase behavior and schematic diagram of the Na_2S-Na_2SO_4 system during reduction.
- **Supplementary Fig. 10.** XRD patterns of the thermochemical deoxygenation products.
- **Supplementary Fig. 11.** Calibration curve correlating sulfide ion (S^{2-}) concentration with absorbance at λ = 235 nm.
- **Supplementary Fig. 12**. Temperature-dependent consumption of the reducing agent (C) during the carbothermal deoxygenation of Na_2SO_4.
- **Supplementary Fig. 13.** XRD patterns of products obtained from the H_2-thermochemical deoxygenation of Na_2SO_4 at varying temperatures.
- **Supplementary Fig. 14.** Standard Gibbs free energy (ΔG^{θ}) profiles for the carbonation.
- **Supplementary Fig. 15.** Experimental apparatus for the carbonation of Na_2S with CO_2.
- **Supplementary Fig. 16.** Temporal evolution of temperature and pressure within the closed steel reactor.
- **Supplementary Fig. 17.** XRD patterns of the final products after oxidative roasting at 600 °C.
- **Supplementary Fig. 18.** TGA-DTG profiles of the mixture of Na_2S and charcoal under a CO_2 atmosphere.
- **Supplementary Fig. 19.** Conversion of Na_2SO_4 to Na_2CO_3 via a sequential gas-flow strategy.
- **Supplementary Fig. 20.** Gas chromatogram (GC) of the reactor effluent.
- **Supplementary Fig. 21.** Morphology and elemental distribution of the synthesized Na_2CO_3 product.
- **Supplementary Fig. 22.** Morphology and EDS mapping of the S_x product.
- **Supplementary Fig. 23.** Physicochemical basis for the separation and recovery of Na_2CO_3 and S_x.
- **Supplementary Fig. 24.** Comparison of thermodynamic stability and overlapping efficiency between CO_2 (O=C=O) and CS_2 (S=C=S).
- **Supplementary Fig. 25.** Structural and morphological evolution of during carbonation.
- **Supplementary Fig. 26.** Investigation of potential side reactions between Na_2S and CO_2
- **Supplementary Fig. 27.** SEM morphology and EDS elemental analysis (S, C, O, and Na) of the final product obtained from the scale-up production.
- **Supplementary Fig. 28.** Optical photograph of the purified Na_2CO_3 powder recovered from 50 g of waste Na_2SO_4.
- **Supplementary Fig. 29.** System boundary and process flow for the thermochemical conversion of waste Na_2SO_4 to Na_2CO_3 (this study).

- **Supplementary Fig. 30.** Normalized environmental impact profiles of the SSA-Process, modified SSA-Process, and the proposed method (this work).
- **Supplementary Fig. 31.** Contribution analysis of mid-point environmental impact categories.
- **Supplementary Fig. 32.** Contribution analysis of end-point environmental impact categories.
- **Supplementary Fig. 33.** Contribution analysis of CED.
- **Supplementary Fig. 34.** Energy- and material-balance analysis for thermochemical upcycling of 1000 kg waste $Na_2SO_4 \cdot 10H_2O$.
- **Supplementary Fig. 35.** The global price trend of the relevant products in the past five years.
- **Supplementary Fig. 36.** Comparative assessment of environmental impacts and CED under the PV-driven scenario.
- **Supplementary Fig. 37.** Sensitivity analysis of economic performance to electricity price fluctuations.
- **Supplementary Fig. 38.** Impact of Na_2CO_3 price volatility on end-point environmental performance.
- **Supplementary Fig. 39.** Environmental implications and upcycling potential of waste Na_2SO_4.
- **Supplementary Fig. 40.** Strategies for closing the "Na_2SO_4-Na_2CO_3" loop via multi-energy integration.
- **Supplementary Fig. 41.** Overview of typical waste sulfates (e.g., Na_2SO_4, $CaSO_4$, $ZnSO_4$) identified as potential renewable resources.
- **Supplementary Fig. 42.** Future perspectives on industrial deployment and low-carbon evolution of the proposed technology.
- **Supplementary Fig. 43.** XRD pattern of charcoal derived from the carbonization of cherry wood.

Supplementary Tables

Supplementary Table 1 | Inventory of Na_2SO_4 waste generation across key industrial sectors. Data are sourced from industrial production records.

Industry Sector	Product & Process	Na_2SO_4 Waste Generation (kg/t-product)
**Chemical Industry**	Lithium carbonate	> 2,700
	Sodium hydroxide	5 – 10
	Ammonium sulfate	1 – 2
	Oxalic acid	500 – 800
	Penicillin	300 – 500
	Formic acid	400 – 600
**Coal Chemical Industry**	Coal combustion (Desulfurization)	30 – 120
**Paper Industry**	Pulping process	200 – 400
**Glass Industry**	Glass	~30

Industrial processes are significant sources of Na_2SO_4 waste, as detailed in **Supplementary Table 1**. Notably, lithium carbonate (Li_2CO_3) production is highly waste-intensive; the extraction process, which relies heavily on Na_2CO_3, generates > 2.7 tons of Na_2SO_4 byproduct for every ton of Li_2CO_3 produced[1]. Similarly, in the coal chemical industry, stringent environmental regulations mandate the removal of SO_2 generated during combustion. While Ca-based absorbents are widely used, Na-based compounds remain essential for applications requiring superior desulfurization efficiency, thereby contributing further to the Na_2SO_4 waste stream[2].

Supplementary Table 2 | Inventory of Na_2CO_3 utilization across various industrial sectors. The specific consumption data represents the amount of Na_2CO_3 required per ton of product.

Industry Sector	Product / Commodity	Specific Consumption (kg/t)
**Metallurgical Industry**	Steel	15 – 20
	Al	~410
	Hard metal	~1,350
	Au	27,000
	Electrolytic Bi	~150
	Electrolytic Ni	~80
	Refined Sb	42
	Cryolite	~620
	$CoCO_3$	3
**Chemical Industry**	Synthetic ammonia	11.3
	Sulfur blue	767
	Epoxy resin	~400
	Polyvinyl chloride	4
	Naphthol	~230
	Chrome yellow	~700
**Pharmaceutical Industry**	Sulfathiazole	1,120
	Sulphaguanidine	550
	Aminopyrine	1,260
	Aureomycin	11,400
	Tetracycline	25,000
	Streptomycin	21,000
**Food Industry**	Soy sauce	~80
	Monosodium glutamate	120
**Glass Industry**	Glass	~200
**Textile Industry**	Cloth (Dyeing & Finishing)	30 – 50

Supplementary Table 3 | Summary of state-of-the-art developments in green NH_3 synthesis.

		Catalyst	Electrolyte	Production rate	Current efficiency	Ref.
**Electro-catalysis**	N_2 direct electro-reduction	Fe HF	0.1 M Na_2SO_4	27.1 $\mu g\cdot h^{-1}\cdot cm^{-2}$	3.5%	Zhang et al. (2025)[3]
		Ru SAs/N-C	0.05 M H_2SO_4	120.9 $\mu g\cdot h^{-1}\cdot mg_{cat}^{-1}$	29.6%	Geng et al. (2018)[4]
		Trinuclear nickel thiolate complex	PhOH-0.1 M $^{n}Bu_4NClO_4$	/	83%	Das et al. (2025)[5]
	Li-mediated N_2 reduction	Lithium-deposited stainless-steel cloth	Lithium-doped polyethylene oxide	< 2.55 $mmol\cdot h^{-1}\cdot cm_{geo}^{-2}$	< 10.6%	Cai et al. (2023)[6]
		Molten salt-based NH_3 synthesis system (Ni rod cathode)	Molten LiCl-KCl	3.24 $\mu mol\cdot h^{-1}\cdot g_{molten\ salt}^{-1}$	/	Xi et al. (2025)[7]
		Cu-Li/Li_3N	$LiBF_4$ THF EfOH	< 34.2 $\mu mol\cdot h^{-1}\cdot cm^{-2}$	< 20.4%	Lazouski et al. (2019)[8]
	NO_3^- electro-reduction	Cu-Pd	1 M KNO_3 + 1M KOH	19.9 $mmol\cdot h^{-1}\cdot cm^{-2}$	86.8%	Fu et al. (2025)[9]
		$RuIn_3$/C	0.1 M KOH (10 mM NO_3^-) + 0.1 M KOH	2.7 $mmol\cdot h^{-1}\cdot mg_{Ru}^{-1}$	97.6%	Huang et al. (2023)[10]
		Ru-CuNW	0.258 M NaOH (2,000 ppm NO_3^-) + Nafion-117 + 0.258 M NaOH	/	> 90%	Chen et al. (2024)[11]
		Ni@Cu_{2-x}Se	1 M KOH (0.1 M NO_3^-) + 1 M KOH	17.2 $mg\cdot h^{-1}\cdot cm^{-2}$	91.6%	Dang et al. (2025)[12]
		$Ni(OH)_x$/Cu	1 M KOH (0.1 M NO_3^-)	3 $mmol\cdot h^{-1}\cdot cm^{-2}$	92%	Liu et al. (2024)[13]
		Cu SAGs	0.1 M PBS, pH = 7.0, and 20 mM NO_3^-	/	97%	Li et al. (2023)[14]

**Photo-catalysis**	N_2 direct reduction	Fe-SA/$WO_{2.72-x}$	Ultrapure water	186.5 $\mu mol\cdot h^{-1}\cdot cm^{-2}$	/	Hu et al. (2022)[15]
		Fe doped MoO_{3-x} nanowires	N_2-saturated solution	742.92 $\mu mol\cdot g^{-1}\cdot h^{-1}$	/	Yu et al. (2025)[16]
		Carbon shell coated defective WO_3	Ultrapure water (2 %methanol)	52.6 $\mu mol\cdot g^{-1}\cdot h^{-1}$	/	Ren et al. (2025)[17]
	NO_3^- photo-reduction	MoS_{2-x}/$ZnIn_2S_{4-x}$ heterojunction	KNO_3 solution (30 $mg\cdot L^{-1}$)	185.59 $\mu mol\cdot g^{-1}\cdot h^{-1}$	/	Zhang et al. (2024)[18]
		Cu-TiO_2	0.01 M KNO_3	~0.49 $\mu g\cdot g^{-1}\cdot h^{-1}$	/	Hiramatsu et al. (2024)[19]
**Liquid metal-catalysis**	Liquid metal-catalysis synthesis	Cu-Ga	/	3,400 $\mu mol\cdot g^{-1}\cdot h^{-1}$	/	Guan et al. (2024)[20]
		$CuGa_2$	/	10,000 $\mu mol\cdot g^{-1}\cdot h^{-1}$	/	Zeng et al. (2025)[21]

Supplementary Table 4 | Chemical composition of desulfurization waste determined by XRF. The data are adapted from **Supplementary Ref. 2**[2].

**Components**	**Weight (%)**		
C	1.29	Fe_2O_3	1.04
F	0.443	CuO	0.0108
**Na_2O**	**46.9**	ZnO	0.0236
MgO	0.051	SeO_2	0.0147
Al_2O_3	0.033	Br	0.0447
SiO_2	0.090	Rb_2O	0.0094
P_2O_5	0.0100	I	0.0696
**SO_3**	**39.3**	PbO	0.0818
**Cl**	**7.15**	Others	0.0384
K_2O	1.26	**Total**	**100**
CaO	2.14		

Supplementary Table 5 | Life cycle inventory (LCI) for Na_2CO_3 production via thermochemical upcycling of waste Na_2SO_4 designed with a continuous recycling loop. Functional unit: production of 1 kg of dense Na_2CO_3. The detailed process flow is shown in **Supplementary Fig. 29**.

Inputs/outputs	Unit	Quantity	Comments
**Step 1: Dehydration and deoxygenation of Na_2SO_4**			
Inputs			
Waste $Na_2SO_4 \cdot 10H_2O$	kg	3.040	Calculated based on stoichiometry.
Transportation	tkm	0.304	Assumed transport distance of 100 km.
Electricity 1	kWh	0.526	Dehydration energy consumption, see **Supplementary Note 1** for details.
Charcoal	kg	0.453	Excess carbon sources are based on experimental data.
CO	kg	0.264	Recycled flow from **Step 2**.
Electricity 2	kWh	0.542	Heating energy consumption (750 °C, 4h), see **Supplementary Note 1** for details.
Outputs			
Na_2S	kg	0.736	Intermediate product for **Step 2**.
CO_2 1, biogenic	kg	0.830	Recycled flow to **Step 2**.
CO_2 2, biogenic	kg	0.208	Direct emission to air.
Charcoal (unreacted)	kg	0.283	Excess charcoal passed to **Step 2**.
Water	kg	1.700	Recovered via condensation.
**Step 2: Carbonation**			
Inputs			
Na_2S	kg	0.736	From **Step 1**.
CO_2	kg	0.830	From **Step 1**.
Charcoal	kg	0.283	Unreacted residue from Step 1 (inert in **Step 2**).
Electricity 3	kWh	0.242	Heating energy consumption (300 °C, 4h), see **Supplementary Note 1** for details.
Outputs			
Mixture (Na_2CO_3 + charcoal)	kg	1.283	Solid product mixture.
S	kg	0.302	Byproduct.
CO	kg	0.264	Recycled flow to **Step 1**.
**Step 3: Purification and separation**			
Inputs			
Mixture (Na_2CO_3 +	kg	1.283	From **Step 2**.

charcoal)			
Water (process)	kg	3.333	Solvent (based on solubility 0.45 kg/L with 1.5× safety factor).
Electricity 4	kWh	0.345	Energy for densification (0.9 GJ/t-Na_2CO_3)[22].
Outputs			
Na_2CO_3 (dense)	kg	1.000	Final product.
Charcoal	kg	0.283	Recycled flow to **Step 1**.
Water	kg	3.333	Recovered via evaporation/condensation.
**Overall**			
Inputs			
Waste $Na_2SO_4 \cdot 10H_2O$	kg	3.040	Zero burden assumption.
Transportation	tkm	0.304	Uncertainty distribution: 1.50, 1.10, 1.00, 1.00, 2.00, 2.00; GSD: 2.90.
Charcoal	kg	0.170	Uncertainty distribution: 1.20, 1.10, 1.00, 1.00, 2.00, 1.05; GSD: 2.06.
Electricity (1-3)	kWh	1.311	Uncertainty distribution: 1.20, 1.10, 1.00, 1.00, 2.00, 1.05; GSD: 2.06.
Electricity 4	kWh	0.346	Uncertainty distribution: 1.20, 1.05, 1.50, 1.05, 1.05, 1.05; GSD: 1.58.
Outputs			
Na_2CO_3 (dense)	kg	1.000	Product (economic allocation: 84%).
S	kg	0.302	Byproduct (economic allocation: 16%).
CO_2 2, biogenic	kg	0.208	Uncertainty distribution: 1.20, 1.10, 1.00, 1.00, 2.00, 1.05; GSD: 2.06.
Water (Wastewater treatment)	kg	1.700	Net emissions; Uncertainty distribution: 1.20, 1.10, 1.00, 1.00, 2.00, 1.05; GSD: 2.06.

Note: Within the uncertainty distribution parameters, U_1, U_2, U_3, U_4, U_5, and U_b represent reliability, completeness, temporal correlation, geographical correlation, technological correlation, and the basic uncertainty factor, respectively.

Supplementary Table 6 | Design parameters of enlarged furnace in LCA calculation of Na_2CO_3 production[23].

Physical entity	Symbol	Unit	3000 L
Reaction mixture volume	$V_{(mix)}$	m^3	3
Height of reactor	L	m	1.614
Diameter of reactor	D	m	1.614
Surface area	A	m^2	12.275
Reactor volume	$V_{(reactor)}$	m^3	3.3
Insulation material	/	/	Glass fiber
Thermal conductivity of insulation	k_a	W/m·°C	0.042
Insulation thickness	s	m	0.075
Heat transfer coefficient of insulation	k_a/s	$W/m^2 \cdot °C$	0.56
Efficiency of heating element	η_{heat}	%	75%
Rate of heat loss	Ak_a/s	W/°C	6.874
Starting and outside temperature	$T_0 = T_{out}$	°C	25

Supplementary Table 7 | LCI data for Na_2CO_3 production via the modified SSA-Process integrating electrochemical NH_3 synthesis and anti-solvent crystallization[24].

Functional unit: production of 1 kg of dense Na_2CO_3. System boundary: Cradle-to-gate.

Inputs/outputs	Unit	Quantity	Comments
**Step 1: Electrochemical NH_3 synthesis** (data sourced from **Supplementary Ref. 25**[25])			
Inputs			
Air	kg	0.333	Calculated based on stoichiometry.
Water	kg	0.528	Calculated based on stoichiometry.
Electricity 1	kWh	0.043	Energy for N_2 production (0.584 MJ/kg-N_2).
Electricity 2	kWh	3.229	Energy for H_2 production (55 kWh/kg-H_2).
Electricity 3	kWh	0.104	Energy for NH_3 synthesis (1.165 MJ/kg-NH_3).
Outputs			
NH_3	kg	0.320	Intermediate product for **Step 2**.
H_2	kg	0.002	Loss (3.32%), emission to air.
O_2	kg	0.540	Byproduct emission to air.
**Step 2: Carbonation, calcination, and densification**			
Inputs			
Waste $Na_2SO_4 \cdot 10H_2O$	kg	3.040	Stoichiometric requirement.
Waste $Na_2SO_4 \cdot 10H_2O$ (excess)	kg	1.173	Excess feed based on equilibrium data[24].
Transportation	tkm	0.304	Assumed transport distance of 100 km.
NH_3	kg	0.320	From **Step 1**.
CO_2	kg	0.830	Stoichiometric requirement.
Electricity 4	kWh	0.578	Used for pumps, compressors, and agitation (based on data from similar chemical processes; increased due to the addition of solvent systems)
Water	kg	3.589	Solvent (1.5× safety factor based on solubility of Na_2SO_4).
Electricity 5	kWh	1.064	Energy for $NaHCO_3$ decomposition[26].
Electricity 6	kWh	0.250	Energy for densification (0.9 GJ/t-Na_2CO_3)[22].
Outputs			
Na_2CO_3 (dense)	kg	1.000	Final product.
CO_2 (recycled)	kg	0.415	Recycled flow to **Step 2**.
Water	kg	0.170	Recovered via condensation.
Mother liquor (Na^+-NH_4^+-SO_4^{2-})	kg	7.367	Intermediate stream to **Step 3**.
**Step 3: Mother liquor concentration and separation**			
Inputs			

Mother liquor (Na^+-NH_4^+-SO_4^{2-})	kg	7.367	From **Step 2**.
Electricity 7	kWh	1.036	Energy for initial concentration, see **Supplementary Note 1** for details.
Solvent (MEA:MEG = 1:1)	kg	1.456	Anti-solvent for crystallization.
Electricity 8	kWh	0.369	Energy for post-separation solvent recovery, see **Supplementary Note 1** for details.
Outputs			
$(NH_4)_2SO_4$	kg	1.250	Byproduct.
Solvent	kg	1.383	Recycled flow to **Step 3**.
Solvent (hazardous waste treatment)	kg	0.073	Loss (5%), treated as hazardous waste[27].
Water	kg	4.944	Recovered via condensation.
Waste $Na_2SO_4{\cdot}10H_2O$	kg	1.173	Recycled flow to **Step 2**.
**Overall**			
Inputs			
Air	kg	0.333	Assumed burden-free.
Electricity (1-3)	kWh	3.375	Uncertainty distribution: 1.20, 1.10, 1.00, 1.05, 1.05, 1.05; GSD: 1.25.
Waste $Na_2SO_4{\cdot}10H_2O$	kg	3.040	Assumed burden-free.
Transportation	tkm	0.304	Uncertainty distribution: 1.50, 1.10, 1.00, 1.00, 2.00, 2.00; GSD: 2.90.
CO_2	kg	0.415	Uncertainty distribution: 1.20, 1.10, 1.00, 1.00, 2.00, 1.05; GSD: 2.06.
Electricity (4, 7, 8)	kWh	1.983	Uncertainty distribution: 1.20, 1.10, 1.00, 1.00, 2.00, 1.05; GSD: 2.06.
Electricity (5, 6)	kWh	1.314	Uncertainty distribution: 1.20, 1.05, 1.50, 1.05, 1.05, 1.05; GSD: 1.58.
Solvent	kg	0.073	Uncertainty distribution: 1.20, 1.10, 1.50, 1.05, 2.00, 1.05; GSD: 2.30.
Outputs			
Na_2CO_3 (dense)	kg	1.000	Product (economic allocation: 57%).
$(NH_4)_2SO_4$	kg	1.250	Byproduct (economic allocation: 43%).
Water (wastewater treatment)	kg	0.997	Net emissions; Uncertainty distribution: 1.20, 1.10, 1.00, 1.00, 2.00, 1.05; GSD: 2.06.
Solvent (hazardous waste treatment)	kg	0.073	Uncertainty distribution: 1.20, 1.10, 1.50, 1.05, 2.00, 1.05; GSD: 2.30.
H_2		0.002	Uncertainty distribution: 1.20, 1.10, 1.00, 1.05,

			1.05, 1.05; GSD: 1.25.
O_2	kg	0.540	Uncertainty distribution: 1.20, 1.10, 1.00, 1.05, 1.05, 1.05; GSD: 1.25.

Supplementary Table 8 | Mid-point environmental impact assessment results of the Na_2CO_3 production process. Values in parentheses represent the photovoltaic (PV)-driven scenario.

Impact category	Unit	SSA-Process	Modified SSA-Process	This work
Global warming	kg CO_2 eq	1.758042084 (1.189936703)	3.335624607 (0.649737929)	1.329170016 (0.346158546)
Stratospheric ozone depletion	kg CFC11 eq	2.32902E-07 (1.56829E-07)	5.28168E-07 (1.68509E-07)	8.96357E-07 (7.64725E-07)
Ionizing radiation	kBq Co-60 eq	0.049750078 (0.024942653)	0.146108834 (0.028824375)	0.04947613 (0.006551024)
Ozone formation, Human health	kg NO_x eq	0.003507984 (0.001951449)	0.008535849 (0.001176866)	0.00324818 (0.000554856)
Fine particulate matter formation	kg PM2.5 eq	0.00248644 (0.001644234)	0.004874339 (0.000892563)	0.00177733 (0.000320034)
Ozone formation, Terrestrial ecosystems	kg NO_x eq	0.003624663 (0.002067919)	0.008598301 (0.001238335)	0.003365817 (0.000672132)
Terrestrial acidification	kg SO_2 eq	0.005798459 (0.003908322)	0.010859342 (0.001923159)	0.003849499 (0.000578933)
Freshwater eutrophication	kg P eq	0.000403585 (0.000322918)	0.000627947 (0.000246571)	0.000213107 (7.3527E-05)
Marine eutrophication	kg N eq	0.000885644 (0.000880121)	0.000110359 (8.4246E-05)	2.24994E-05 (1.29422E-05)
Terrestrial ecotoxicity	kg 1,4-DCB	4.469192213 (5.469660885)	4.360302483 (9.090314903)	1.38992887 (3.121072801)
Freshwater ecotoxicity	kg 1,4-DCB	0.045499119 (0.057739294)	0.046421938 (0.104290998)	0.012664453 (0.033844032)
Marine ecotoxicity	kg 1,4-DCB	0.060142768 (0.075031623)	0.06276473 (0.133156209)	0.017671811 (0.043434487)
Human carcinogenic toxicity	kg 1,4-DCB	0.065114516 (0.052332345)	0.114810628 (0.054379127)	0.035127132 (0.013009721)
Human non-carcinogenic toxicity	kg 1,4-DCB	1.093827993 (0.999403096)	1.555439581 (1.109017864)	0.502556473 (0.339169956)
Land use	m^2a crop eq	0.024402616 (0.018582822)	0.040037012 (0.012522209)	0.174129293 (0.164059112)
Mineral resource scarcity	kg Cu eq	0.003280938 (0.00367873)	0.002393923 (0.004274601)	0.000603257 (0.001291569)
Fossil resource scarcity	kg oil eq	0.326838693 (0.219024176)	0.665764832 (0.156039725)	0.222939757 (0.036384744)

Water consumption	m^3	0.024067092 (0.020252414)	0.026575589 (0.008540566)	0.009263805 (0.002663141)

Supplementary Table 9 | End-point environmental impact assessment results of the Na_2CO_3 production process. Values in parentheses represent the PV-driven scenario.

Damage category	Unit	SSA-Process	Modified SSA-Process	This work
Total	mPt	48.809989 (33.94760969)	91.7130932 (21.44678697)	34.69035565 (8.973490672)
Human health	mPt	46.27969794 (32.14969882)	86.94970921 (20.14594754)	32.55384782 (8.104244424)
Ecosystems	mPt	2.00674717 (1.378865911)	3.760929346 (0.792434469)	1.835624023 (0.749180378)
Resources	mPt	0.52354389 (0.419044963)	1.002454636 (0.508404968)	0.300883806 (0.120065869)

Supplementary Table 10 | Cumulative energy demand (CED) of the Na_2CO_3 production process. Values in parentheses represent the PV-driven scenario.

Impact category	Unit	SSA-Process	Modified SSA-Process	This work
Total	MJ	17.52159994 (14.25165275)	38.55206788 (23.09242279)	20.20675048 (14.54865315)
Non-renewable, fossil	MJ	15.12263745 (10.10711832)	30.86446641 (7.152112206)	10.3478856 (1.669367544)
Non-renewable, nuclear	MJ	0.933481246 (0.467572482)	2.787091448 (0.584369552)	0.930673764 (0.124496453)
Non-renewable, biomass	MJ	0.000481799 (0.000476726)	0.000292306 (0.000268321)	0.0013802 (0.001371422)
Renewable, biomass	MJ	0.263506612 (0.211227762)	0.396379877 (0.149216108)	7.330232144 (7.239772352)
Renewable, wind, solar, geothemal	MJ	0.500505891 (3.181676414)	2.069160494 (14.74518944)	0.743277341 (5.382595141)
Renewable, water	MJ	0.700986948 (0.283581052)	2.434677346 (0.461267161)	0.853301429 (0.131050236)

Supplementary Table 11 | LCI for the production of 68% H_2SO_4 and 50% NaOH solutions via bipolar membrane electrodialysis (BMED).

Inputs/outputs	Unit	Quantity	Comments
**Step 1: Electrodialysis**			
Inputs			
Waste $Na_2SO_4 \cdot 10H_2O$	kg	1.000	Calculated via stoichiometry.
Transportation	tkm	0.100	Assumed transport distance of 100 km.
Water	kg	9.023	To maintain Na_2SO_4 concentration at 0.8 mol/L.
Electricity 1	kWh	1.168	Power for electrodialysis[28].
Outputs			
5.9% H_2SO_4	kg	5.309	Intermediate for **Step 2**.
5.3% NaOH	kg	4.714	Intermediate for **Step 3**.
**Step 2: H_2SO_4 concentration**			
Inputs			
5.9% H_2SO_4	kg	5.309	From Step 1.
Electricity 2	kWh	2.411	Energy for H_2SO_4 concentration[22].
Outputs			
68% H_2SO_4	kg	0.461	Final Product.
Water	kg	4.848	Recovered via evaporation.
**Step 3: NaOH concentration**			
Inputs			
5.3% NaOH	kg	4.714	From **Step 1**.
Electricity 3	kWh	0.820	Energy for NaOH concentration[22].
Outputs			
50% NaOH	kg	0.500	Final Product.
Water	kg	4.214	Recovered via evaporation.
**Overall**			
Inputs			
Waste $Na_2SO_4 \cdot 10H_2O$	kg	1.000	Assumed burden-free.
Transportation	tkm	0.100	Assumed transport distance of 100 km.
Water	kg	9.023	To maintain Na_2SO_4 concentration at 0.8 mol/L.
Electricity (1-3)	kWh	4.399	Total energy consumption.
Outputs			
68% H_2SO_4	kg	0.461	Product.
50% NaOH	kg	0.500	Product.
Water (wastewater treatment)	kg	9.063	Net discharge (process water + crystal water − products).

Note: The electrodialysis membranes are considered to operate under stable conditions

without the need for maintenance, and the effects of impurities are not taken into account.

Data reference: **Supplementary Ref. 28**[28].

Supplementary Table 12 | Summary of life cycle costs (LCC) for BMED (yielding H_2SO_4 and NaOH), the modified SSA-Process, and this work. The assessment is based on a functional unit of processing 1,000 kg of waste $Na_2SO_4 \cdot 10H_2O$. Calculations utilize average market prices over the past five years (see **Supplementary Fig. 35** for historical price fluctuations of all products). Primary values are calculated based on coal-fired electricity generation, while values in parentheses represent a future PV-driven scenario.

Parameter	BMED	Modified SSA-Process	This work
**1. Variable Operating Expenditures (OPEX) (USD)**			
Feedstock			
Waste $Na_2SO_4 \cdot 10H_2O$	0.00[a]	0.00[a]	0.00[a]
Energy			
Electricity	439.90[b] (189.16)[b]	219.47[b] (94.37)[b]	54.51[b] (23.44)[b]
Chemical Reagents			
Charcoal (Reactant)	—	—	22.37[c]
NH_3	—	—	—
CO_2	—	4.78[d]	—
Process water/solvent	13.53[e]	18.00[f]	1.64[e]
**Total Variable OPEX (USD)**	**453.43 (202.69)**	**242.25 (117.15)**	**78.52 (47.45)**
**2. Fixed Costs (USD)**			
**Fixed Costs (Depreciation, Labor, etc.)**[g] *(Calculated as 15% of Total LCC)*	**80.02 (35.77)**	**42.75 (20.67)**	**13.86 (8.37)**
**3. Total LCC (USD)**			
(Sum of Variable OPEX + Fixed Costs)	**533.45 (238.46)**	**285.00 (137.82)**	**92.38 (55.82)**
**4. Revenue Streams (USD)**			
Main Product			
H_2SO_4	34.17[h]	—	—
NaOH	113.25[i]	—	—
Na_2CO_3	—	97.04	97.04
Byproducts			
$(NH_4)_2SO_4$	—	109.79[j]	—
S_x	—	—	18.28
**Total Revenue (USD)**	**147.42**	**206.83**	**115.32**
**5. Economic Performance (USD)**			

**Net Profit** *(Total Revenue − Total LCC)*	**−386.03** (−91.04)	**−78.17** **(+69.01)**	**+22.94** **(+59.50)**
**Profit Margin (%)**	**−261.86%** **(−61.76%)**	**−37.79%** **(+33.36%)**	**+19.89%** **(+51.59%)**

a: Waste $Na_2SO_4 \cdot 10H_2O$ is assumed to be burden-free (0.00 USD) as it is a waste stream.

b: Baseline electricity cost is set at 0.10 $USD \cdot kWh^{-1}$. A sensitivity scenario considers a reduced rate of 0.043 $USD \cdot kWh^{-1}$, reflecting the global weighted average cost of utility-scale solar PV (Source: International Renewable Energy Agency (IRENA), https://www.irena.org/).

c: Charcoal (reductant) is priced at 400 $USD \cdot ton^{-1}$ based on prevailing market rates in China; future adoption of low-cost alternative carbon sources could further reduce expenses.

d: Industrial CO_2 is priced at 35 $USD \cdot ton^{-1}$, reflecting capture costs in the Chinese region (Source: CCUS and Global Power Generation Analysis)[29].

e: Process water is valued at 1.50 $USD \cdot ton^{-1}$.

f: Solvent costs are based on Chinese market prices: MEA at 1,000 $USD \cdot ton^{-1}$ and MEG at 500 $USD \cdot ton^{-1}$. A makeup rate of 5% per cycle is assumed to account for operational losses.

g: Fixed costs account for components that cannot be directly priced at the laboratory scale, such as annualized capital expenditures (CAPEX) and labor. Given the absence of a detailed engineering design at this preliminary stage, fixed costs were estimated using standard heuristic factors widely applied in the chemical process industry. Specifically, fixed costs are assumed to constitute a typical 15% of the total LCC, with variable OPEX accounting for the remaining 85%. The integration of this estimation effectively bridges the gap between laboratory-scale data and industrial realities, providing a more realistic economic evaluation. The mathematical formulation utilized is: Fixed costs = Variable OPEX × (0.15/0.85).

h: Product revenue for NaOH and H_2SO_4 is calculated on a 100% pure substance basis (e.g., the output of NaOH is equivalent to 250 kg of pure NaOH).

i: Same as note **h**; H_2SO_4 production is also calculated on a 100% basis.

j: The market value of $(NH_4)_2SO_4$ is estimated at 150% of the standard fertilizer-grade price. This premium reflects the higher purity and economic value achieved through the MEA-MEG purification process compared to conventional fertilizer products.

Supplementary Notes

Supplementary Note 1 | Detailed calculation procedures for energy consumption.

Electricity 1 (Supplementary Table 5):

The dehydration reaction of $Na_2SO_4 \cdot 10H_2O$ is expressed as:

$Na_2SO_4 \cdot 10H_2O(s) = Na_2SO_4(s) + 10H_2O(g)$ $\Delta H = 522\ kJ \cdot mol^{-1}$ (Supplementary Ref. 30[30])

Processing 3.040 kg of the feedstock ($Na_2SO_4 \cdot 10H_2O$) corresponds to 9.44 mol, resulting in a theoretical energy demand of 4,925 kJ. By employing a triple-effect evaporator with an energy efficiency ratio of 2.6[31], the actual energy consumption is estimated to be **0.526 kWh**.

Electricity 2 (Supplementary Table 5):

The energy balance analysis of thermochemical upcycling of waste Na_2SO_4 is as follows (based on 1,000 kg of Na_2SO_4):

- *Heating Energy Consumption*

Specific heat capacity of Na_2SO_4: 898.3 $J \cdot (kg \cdot °C)^{-1}$

Specific heat capacity of industrial charcoal: 0.8-1.0 $kJ \cdot (kg \cdot °C)^{-1}$, with an average value of 900.0 $J \cdot (kg \cdot °C)^{-1}$.

Specific heat capacity of CO: 1,040.4 $J \cdot (kg \cdot °C)^{-1}$

Thus, the total energy required to heat the materials to 750 °C is 1,020,689 kJ.

Considering an energy efficiency of 0.75, the calculated total energy required is:

1,360,918.7 kJ or **378.0 kWh**.

- *Constant Temperature Energy Consumption*

The density of Na_2SO_4 is 2.67 $t \cdot m^{-3}$, and the density of charcoal is approximately 0.4 $t \cdot m^{-3}$. The recycled CO is uniformly input as a gas at a rate of 50 kg per h.

The volume of 1,000 kg of Na_2SO_4 and 338.2 kg of charcoal is calculated as follows:

1,000.0 kg Na_2SO_4 gives a volume of 374.4 L, 338.2 kg charcoal gives a volume of 845.5 L.

Thus, the total volume is approximately: 1,219.9 L.

Using a 3,000 L furnace for the calculation (Supplementary Table 6), with a porosity of 59.3%.

Q_{loss} = 6.874 W·°C^{-1} × (750-25) °C × 4h·(0.75)$^{-1}$ = **26.6 kWh**.

Therefore, the total energy consumption for **Electricity 2** is **404.6 kWh·(t-Na_2SO_4)$^{-1}$ (0.542 kWh per 3.040 kg Na_2SO_4·10H_2O)**.

Electricity 3 (Supplementary Table 5):

Cooling of solid products from 750 °C and CO_2 stored at ambient temperature with subsequent heating.

- *Specific Heat Capacities*

Specific heat capacity of CO: 843.7 J·(kg·°C)$^{-1}$.

Na_2S is cooled from 750 °C to 300 °C for the reaction, with no additional heating energy required.

With an energy efficiency of 0.75, the total energy consumption for this process is: 191,708.3 kJ or **53.3 kWh**.

- *Sublimation Heat of Sulfur*

Heat of vaporization for sulfur: 45 kJ·kg^{-1}.

225.7 kg S × 1,403.6 kJ/kg = 316,792.5 kJ.

With an energy efficiency of 0.75, the total energy consumption for sulfur sublimation is: 422,390.0 kJ or **117.3 kWh**.

- *Constant Temperature Energy Consumption*

Q_{loss} = 6.874 W·°C^{-1} × (300-25) °C × 4h·(0.75)$^{-1}$ = **10.1 kWh**.

Therefore, the total energy consumption for **Electricity 3** is **180.7 kWh·(t-Na_2SO_4)$^{-1}$ (0.242 kWh per 3.040 kg Na_2SO_4·10H_2O)**.

Based on the above calculations, we established a 1,000 kg Na_2SO_4 recovery pathway and plotted analysis diagrams of energy-balance and material-balance (**Supplementary Fig. 34**).

Electricity 7 (Supplementary Table 7):

During the concentration process, 4.144 kg of water is removed. The liquid phase exits the carbonization reactor at 80 °C, and the boiling point is set to 100 °C. Based on the specific heat capacity of water (C_p = 4.184 kJ·(kg·°C)$^{-1}$) and its latent heat of vaporization (ΔH_{vap} ≈ 2,257 kJ·kg^{-1}), the theoretical energy demand is calculated to be 9,699.8 kJ. By

employing a triple-effect evaporator with an energy efficiency ratio of 2.6[31], the actual energy consumption is estimated to be **1.036 kWh**.

Electricity 8 (Supplementary Table 7):

This stage involves the evaporation of a mixture containing 0.800 kg of water, 0.728 kg of monoethanolamine (MEA), and 0.728 kg of monoethylene glycol (MEG). Physical properties were obtained from the CAMEO Chemicals database (https://cameochemicals.noaa.gov/). For MEA, the boiling point is 170.3 °C, with a specific heat capacity (C_p) of ~2.75 kJ·(kg·°C)$^{-1}$) and a latent heat of vaporization (ΔH_{vap}) of 837 kJ·kg^{-1}. For MEG, the boiling point is 197.3 °C, with a C_p of ~2.41 kJ·(kg·°C)$^{-1}$ and a ΔH_{vap} of 800 kJ·kg^{-1}. The total theoretical energy required is calculated to be 3,449.7 kJ. By employing a triple-effect evaporator with an energy efficiency ratio of 2.6[31], the actual energy consumption is estimated to be **0.369 kWh**.

Supplementary Note 2 | Definition and calculation of multidimensional performance metrics for Manuscript Fig. 5g.

This note details the evaluation criteria and calculation methodologies used to construct the radar chart in Fig. 5g. The chart benchmarks the proposed technology ("This work") against the conventional SSA-Process and the modified SSA-Process (coupled with green NH_3 and anti-solvent crystallization) across six key dimensions: Economic feasibility, Recovery rate, NH_3 independence, Low-carbon potential, Energy-saving potential, and Environmental sustainability.

(**i**) Economic feasibility was evaluated using a semi-quantitative scoring system (0-100) that comprehensively considers profitability, robustness, and deployability. The scoring rationale focuses on "risk-adjusted returns" across both current grid and future PV scenarios.

This Work (Score: 95): In the current grid scenario, the net profitability of this work (+22.94 USD·ton^{-1}) remains robust and significantly higher than that of the traditional SSA-Process, indicating immediate deployability without the need to wait for renewable energy infrastructure. Although the theoretical maximum profit under the PV scenario (+59.50 USD·ton^{-1}) is slightly lower than that of the modified SSA-Process, it avoids the critical financial deficits associated with the latter under current conditions. This stable investment strategy ensures commercial viability throughout the global energy transition.

Modified SSA-Process (Score: 60): While exhibiting the highest theoretical profitability (+69.01 USD·ton^{-1}) under a fully decarbonized PV scenario, its feasibility is severely compromised by a significant economic deficit (−78.17 USD·ton^{-1}) under current grid conditions, especially when comprehensive fixed costs are accounted for. This deficit creates a substantial barrier to entry. The economic viability of this process is entirely contingent on the availability of cheap green H_2/NH_3 and low-cost solar energy. High sensitivity to input capital and operating costs makes it a high-risk option for immediate industrial deployment.

SSA-Process (Score: 50): This process operates on extremely thin margins (Note: previously estimated at +9.78 USD·ton^{-1} based solely on OPEX, but highly vulnerable when fixed LCC components are included) and lacks significant growth potential. The profitability

assessment was derived from the modified SSA-Process model by excluding electrolytic NH_3 electricity costs and anti-solvent crystallization costs (using substituting industrial NH_3 costs at 0.45 USD·kg^{-1}), with $(NH_4)_2SO_4$ revenue adjusted to industrial grades. Its economic resilience is exceptionally low; profitability is highly vulnerable to fluctuations in natural gas and NH_3 market prices. Even under PV scenarios, profit growth is fundamentally restricted by stoichiometric constraints and raw material costs.

(**ii**) The recovery rate represents the percentage of sulfate waste effectively converted or utilized.

This Work (Score: 95): Based on experimental data, demonstrating a recovery rate of 95.35% relative to the theoretical yield.

Modified SSA-Process (Score: 98): Assumes a closed-loop system with high-efficiency crystallization, with an estimated material loss of 2%.

SSA-Process (Score: 70): Based on literature values, the open-loop nature of this process typically results in incomplete utilization (~70%)[2].

(**iii**) The NH_3 independence metric evaluates the reliance on external NH_3 resources, reflecting supply chain security and toxicity risks.

This Work (Score: 100): Completely NH_3-free processes.

Modified SSA-Process (Score: 0): Processes dependent on NH_3 input.

SSA-Process (Score: 0): Processes dependent on NH_3 input.

(**iv**) For low-carbon Potential (GWP), energy-saving Potential (CED), and environmental Sustainability (End-point Impact), the raw values were normalized. Since lower values represent better performance for these metrics, scores were calculated using the inverse normalization method relative to the best-performing technology (which receives a score of 100, **Equation S1**).

$$\text{Score}_i = \left(\frac{1/\text{Value}_i}{1/\text{Value}_{min}}\right) \times 100 \qquad \text{S1}$$

Where Value_{min} represents the lowest (best) impact or demand among the three technologies.

A. Low-carbon potential (based on GWP in PV scenario) metric: global warming

potential (kg CO_2-eq·(kg-Na_2CO_3)$^{-1}$).

Technology	Raw Value (kg CO_2-eq)	Inverse Value (1/x)	Normalized Score
**This work**	0.3462	2.8888	100.00
**Modified SSA-Process**	0.6497	1.5391	53.28
**SSA-Process**	1.1899	0.8404	29.09

B. Energy-saving potential (based on CED in PV scenario) metric: cumulative energy demand (MJ·(kg-Na_2CO_3)$^{-1}$).

Technology	Raw Value (MJ)	Inverse Value (1/x)	Normalized Score
**This work**	14.55	0.0687	97.96
**Modified SSA-Process**	23.09	0.0433	61.72
**SSA-Process**	14.25	0.0702	100.00

Note: Although "This Work" exhibits a marginally higher CED than the SSA-Process due to biomass energy inputs, it remains highly competitive (Score 97.96). In contrast, the modified SSA-Process is significantly more energy-intensive, primarily driven by the substantial electricity demand of electrochemical NH_3 synthesis. The superior performance of the SSA-Process in this metric largely reflects the high industrial maturity and thermodynamic optimization of the established Haber-Bosch pathway compared to emerging green synthesis routes.

C. Environmental sustainability (based on end-point impact in PV scenario) metric: total end-point Impact (mPt·(kg-Na_2CO_3)$^{-1}$).

Technology	Raw Value (mPt)	Inverse Value (1/x)	Normalized Score
**This work**	34.69	0.0288	100.00
**Modified SSA-Process**	91.71	0.0109	41.84
**SSA-Process**	48.81	0.0205	26.43

Supplementary Methods | Life cycle assessment (LCA) and LCC assessment methodology.

LCA was employed to evaluate the environmental impacts and cumulative energy demand of current mainstream waste Na_2SO_4 recycling processes compared to the method proposed in this study. The analysis followed the ISO 14040 and 14044 standards and was conducted using SimaPro software[32]. The functional unit was defined as the production of dense soda ash (Na_2CO_3). The system boundary includes inputs (energy and materials) and outputs (wastewater, waste gases, and solid waste) associated with the Na_2CO_3 production process, while the manufacturing and maintenance of capital equipment were excluded.

The LCI for the Na_2CO_3 production process developed in this study is detailed in **Supplementary Table 5**. To evaluate the potential of coupling advanced technologies, we integrated green NH_3 synthesis and an MEA-MEG-H_2O anti-solvent crystallization system into the standard Solvay process (SSA-Process)[24, 25]; this integrated route was evaluated as the "modified SSA-Process" (**Supplementary Table 7**). For comparison, the LCI for the compared soda ash dense production process is based on the global production modeling of the improved Solvay process (Soda Ash process, i.e., SSA-Process in this study) in the Ecoinvent database[33].

The geographic location for electricity and fuel was set to China. Upstream background data for materials were sourced from the Ecoinvent 3.5 database, specifically data representing the Chinese (China, CN) market. When data for the CN region was unavailable, global average (GLO) or other world (ROW) production data were used as proxies. The electricity data for China within the Ecoinvent database was adjusted to reflect recent grid composition: coal-fired power (63.1%), hydropower (15.4%), wind power (8.7%), solar power (4.9%), nuclear power (4.8%), and natural gas power (3.1%). Additionally, the transportation distance for the raw material ($Na_2SO_4 \cdot 10H_2O$) was assumed to be 100 km, modeled using a truck meeting EURO 6 emission standards (>32 t).

Given the generation of byproducts (sulfur and distilled water) in the proposed process, economic allocation was applied to distribute environmental burdens and credits. This

method assigns impacts in a multi-product system proportional to the economic value of the outputs. The five-year average market prices for dense Na_2CO_3, S_x powder, and industrial water were utilized: $295/t, $184/t, and $1.5/t, respectively. Based on the production quantities presented in **Supplementary Table 5**, Na_2CO_3 accounts for 84% of the total environmental burden of the production system.

The ReCiPe 2016 method was utilized to assess environmental impacts, covering both midpoint and endpoint categories. Mid-point categories included global warming potential (GWP), stratospheric ozone depletion (ODP), ionizing radiation (IRP), ozone formation, human health (OFHH), fine particulate matter formation (PMFP), terrestrial ecosystems (OFTE), terrestrial acidification potential (TAP), freshwater eutrophication (FEP), marine eutrophication (MEP), terrestrial ecotoxicity (TEP), freshwater ecotoxicity (FETP), marine ecotoxicity (METP), human carcinogenic toxicity (HTPc), human non-carcinogenic toxicity (HTPcn), land use potential (LUP), mineral resource scarcity (SOP), fossil resource scarcity (FFP), and water consumption (WCP). End-point categories include human health, ecosystems, and resources. Mid-point characterization results and normalized environmental impacts for the Na_2CO_3 production process are provided in **Supplementary Table 8** and **Supplementary Fig. 32**, respectively (values in parentheses denote the scenario utilizing a PV-driven electricity background). End-point environmental impact categories and cumulative energy demand are presented in **Supplementary Tables 9-10**. **Supplementary Figs. 33-35** provide a contribution analysis of environmental impacts under different allocation methods.

To rigorously evaluate the propagation of uncertainty from both foreground inventories and background databases, a Monte Carlo simulation was performed with 1,000 iterations. Uncertainty distributions for inventory inputs and outputs were quantified using the Pedigree Matrix approach. The reliability (U_1), completeness (U_2), temporal correlation (U_3), geographical correlation (U_4), and technological correlation (U_5) were determined for each parameter along with a basic uncertainty factor (U_b). These were synthesized into a geometric standard deviation (GSD) representing the comprehensive uncertainty coefficient, as shown in **Equation S2** (data detailed in **Supplementary Tables 5-7**):

$$GSD = \exp\sqrt{\sum_{i=1}^{5}[\ln(U_i)]^2 + [\ln(U_b)]^2} \quad S2$$

Where:

U_1: Uncertainty factor for reliability;

U_2: Uncertainty factor for completeness;

U_3: Uncertainty factor for temporal correlation;

U_4: Uncertainty factor for geographical correlation;

U_5: Uncertainty factor for technological correlation;

U_b: Basic uncertainty factor.

Supplementary Data 1 summarizes the uncertainty analysis results for all environmental impact indicators, reporting the probabilistic mean, standard deviation, coefficient of variation, and the 95% confidence interval range. Finally, sensitivity analyses were performed. First, the environmental impact of the three processes under a PV-driven scenario was evaluated to quantify emission reduction potentials (**Supplementary Fig. 36**). Second, the robustness of the economic allocation method was verified by assessing the impact of varying soda ash prices (high, average, and low scenarios) (**Supplementary Fig. 38**). The results indicate that while price fluctuations influence the magnitude of environmental impacts, the relative ranking of the technological routes and the study's key conclusions remain unchanged, confirming the reliability of the economic allocation approach used.

The economic feasibility of the upcycling processes was evaluated using an LCC assessment framework. The system boundary was defined as "gate-to-gate," encompassing all core unit operations from the intake of waste feedstocks to the generation of final marketable products. To ensure a standardized comparison across all evaluated methods (BMED, modified SSA-Process, and this work), the functional unit was established as the processing of 1,000 kg of waste $Na_2SO_4 \cdot 10H_2O$. The variable OPEX were directly derived from the mass and energy balances established in the LCI. This includes the costs of electricity, chemical reagents, and process water/solvent. Market prices were determined

using a five-year average to mitigate short-term market volatility (**Supplementary Fig. 35**). The variable OPEX is calculated as:

$$\textbf{Variable OPEX} = \sum (m_i \times P_{m,\,i}) + \sum (E_i \times P_{e,\,i})$$

where m_i and E_j represent the mass of material inputs and energy consumption, respectively, and $P_{m,\,i}$ and $P_{e,\,i}$ denote their corresponding unit market prices.

To satisfy the comprehensive scope of an LCC assessment, the inclusion of fixed costs is imperative. Given the preliminary development stage of the proposed technology, a bottom-up engineering cost estimation is subject to significant uncertainty. Therefore, we adopted a standard factorial approach widely utilized in the chemical process industry. Fixed costs, which encompass annualized CAPEX, plant maintenance, and labor, were designated to constitute 15% of the total LCC, with variable OPEX accounting for the remaining 85%. Accordingly, the fixed costs are calculated as follows:

$$\textbf{Fixed costs} = \text{Variable OPEX} \times (0.15/0.85)$$

Consequently, the total LCC integrates both the variable OPEX and the fixed costs to represent the overall production cost per functional unit:

$$\textbf{Total LCC} = \text{Variable OPEX} + \text{Fixed costs} = \text{Variable OPEX}/0.85$$

Finally, the economic viability was quantified using net profit and profit margin. Total revenue was calculated by multiplying the yield of each product and byproduct by its respective industrial market grade price. The economic metrics were determined using the following equations:

$$\textbf{Net Profit} = \text{Total Revenue} - \text{Total LCC}$$

$$\textbf{Profit Margin (\%)} = \text{Net Profit/Total Revenue} \times 100\%$$

A positive net profit and profit margin indicate economic feasibility, whereas negative values signify an economic deficit under the modeled scenarios.

Supplementary Figures

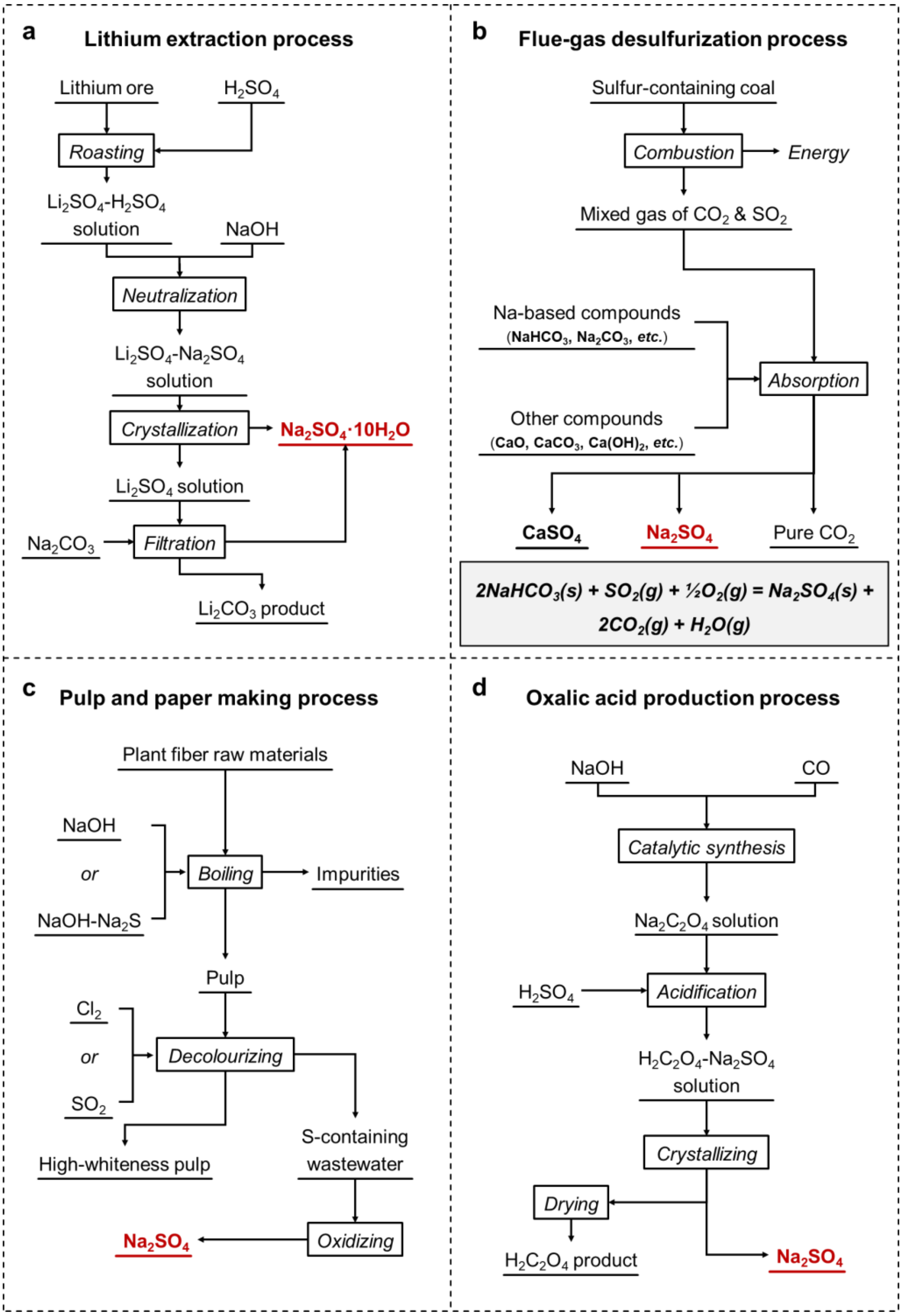

Supplementary Fig. 1 | Generation of Na_2SO_4 waste in major industrial processes. a, Lithium extraction process. **b**, Flue-gas desulfurization process. **c**, Pulp and paper making process. **d**, Oxalic acid production process.

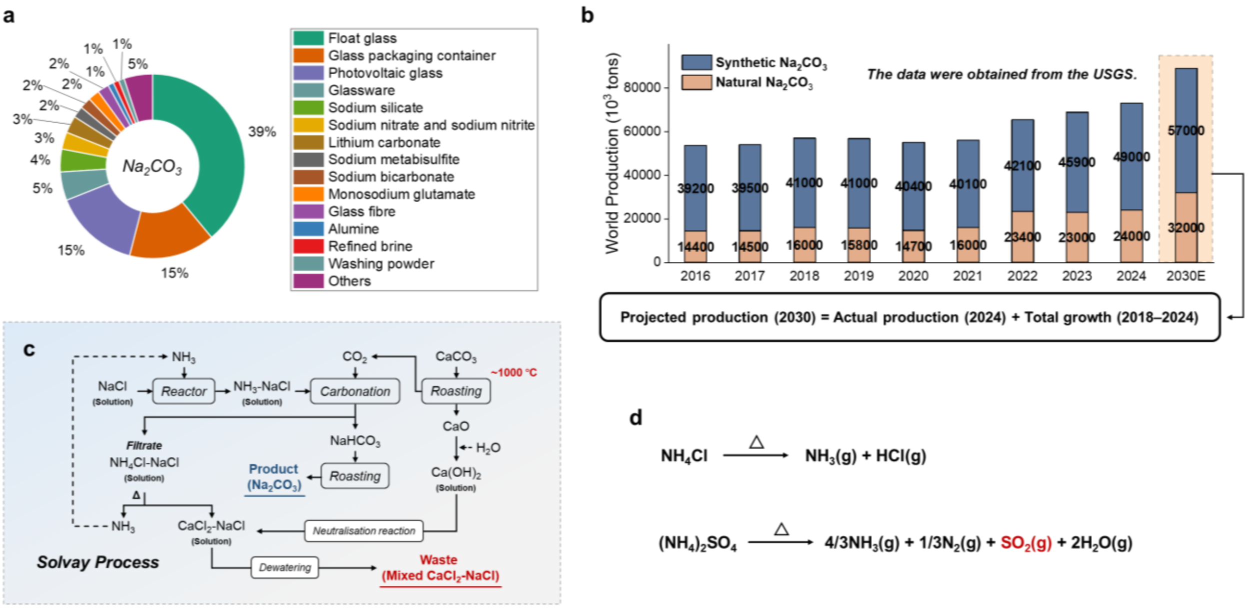

Supplementary Fig. 2 | Market dynamics and conventional production pathways of Na_2CO_3. **a**, Downstream consumption structure of Na_2CO_3 in China (2023). **b**, World production trends for Na_2CO_3 from 2016 to 2030. Data are sourced from the U.S. Geological Survey (USGS). **c**, Schematic flowchart of the conventional Solvay process for Na_2CO_3 production. **d**, Reaction schemes comparing the thermal decomposition of NH_4Cl and $(NH_4)_2SO_4$.

Supplementary Fig. 2a illustrates that the float glass sector dominated consumption in China in 2023, accounting for 39% of the market, followed by glass packaging containers and photovoltaic glass. Propelled by the global transition toward renewable photovoltaic energy and the rapid expansion of the electric vehicle market, the demand for exhibits a sustained upward trajectory (Supplementary Fig. 2b). Currently, industrial production relies predominantly on the Solvay process, a method constrained by high energy intensity (2.8 GJ per ton of Na_2CO_3) and substantial waste generation (Supplementary Fig. 2c)[26].

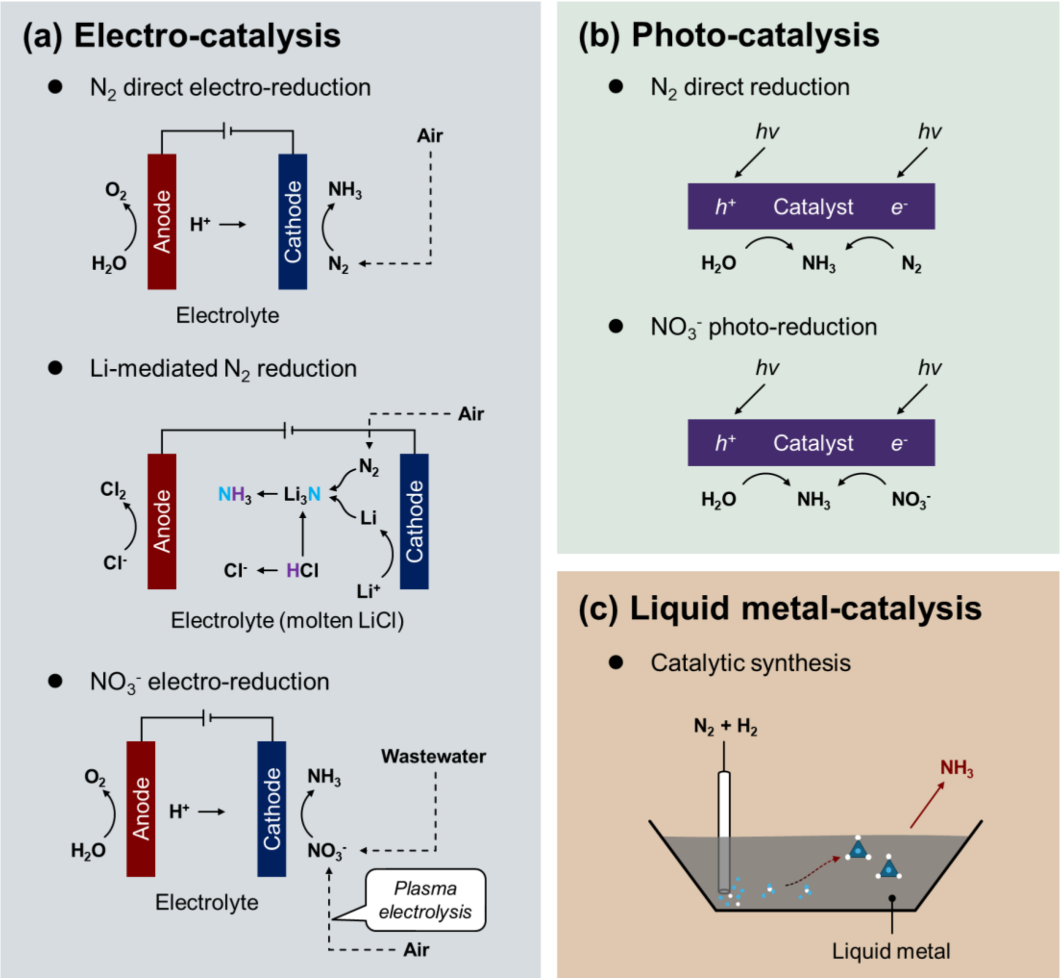

Supplementary Fig. 3 | Schematic representation of emerging alternative NH_3 synthesis pathways. a, Electro-catalytic strategies, encompassing direct nitrogen reduction (N_2 direct electro-reduction), lithium-mediated nitrogen reduction (Li-mediated N_2 reduction), and nitrate electro-reduction (NO_3^- electro-reduction). **b**, Photo-catalytic approaches utilizing light energy to drive direct N_2 reduction and NO_3^- photo-reduction over semiconductor catalysts. **c**, Liquid metal-catalyzed synthesis, where liquid metals facilitate the reaction between gaseous N_2 and H_2.

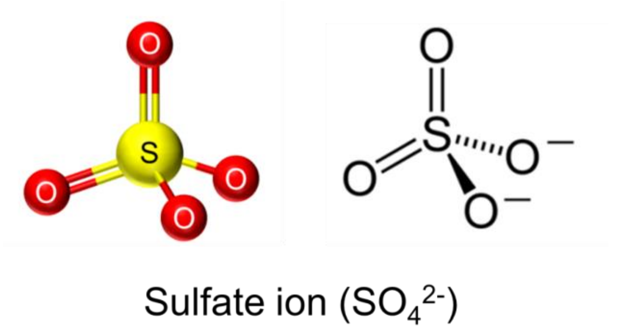

Supplementary Fig. 4 | Molecular geometry and structural configuration of the SO_4^{2-}. Three-dimensional ball-and-stick representation illustrating the tetrahedral coordination geometry (T_d symmetry). The central S atom (yellow) is bonded to four equivalent O atoms (red). Skeletal Lewis structure depicting the bonding connectivity and charge distribution.

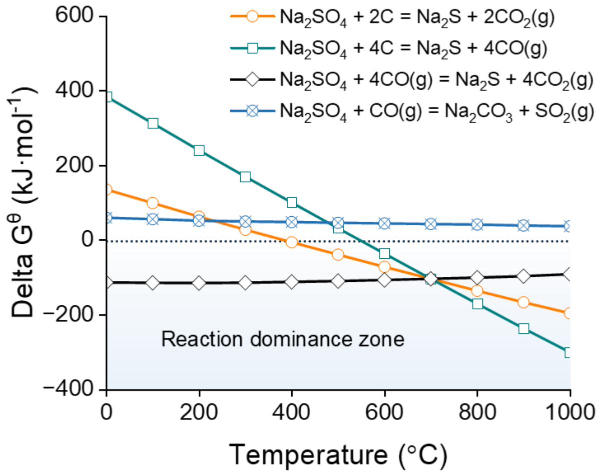

Supplementary Fig. 5 | Thermodynamic feasibility of carbothermal deoxygenation pathways for Na_2SO_4. The plot displays the standard Gibbs free energy ($ΔG^θ$) as a function of temperature for various reaction scenarios. Calculations were performed using HSC Chemistry 9.0 software under standard pressure (1 atm) with unit activity (activity: 1, settings: Show Transitions and Criss-Cobble).

Supplementary Fig. 5 elucidates the thermodynamic driving forces governing the deoxygenation of Na_2SO_4. As the temperature rises, the reduction of Na_2SO_4 to Na_2S via

reaction with solid C or CO exhibits an increasingly negative ΔG^{θ}, indicating spontaneous reaction feasibility—particularly within the 600 – 800 °C window. In contrast, the hypothetical conversion of Na_2SO_4 to Na_2CO_3 (accompanied by SO_2 release) remains thermodynamically unfavorable ($\Delta G^{\theta} > 0$) across the entire investigated temperature range. Consequently, the carbothermal treatment of Na_2SO_4 selectively favors the formation of Na_2S over Na_2CO_3.

$$\boldsymbol{Na_2SO_4 + 2C = Na_2S + CO_2}$$

1.0000 g Na_2SO_4 → **0.5494 g Na_2S**

0.3383 g C → **0.1692 g C**

$$\frac{0.5494 + 0.1692}{1.3383} \times 100\% = 53.69\%$$

Supplementary Fig. 6 | Theoretical solid mass retention calculation for the carbothermal deoxygenation of Na_2SO_4. The calculation assumes an initial precursor mixture with a molar ratio of Na_2SO_4:C = 1:4 (corresponding to 1.0000 g of Na_2SO_4 and 0.3383 g of C). Based on the stoichiometric reaction mechanism ($Na_2SO_4 + 2C \rightarrow Na_2S + 2CO_2$), where carbon is consumed at a 1:2 molar ratio, the theoretical residual solid mass (comprising the product Na_2S and unreacted excess C) is determined to be 53.69% of the total initial mass.

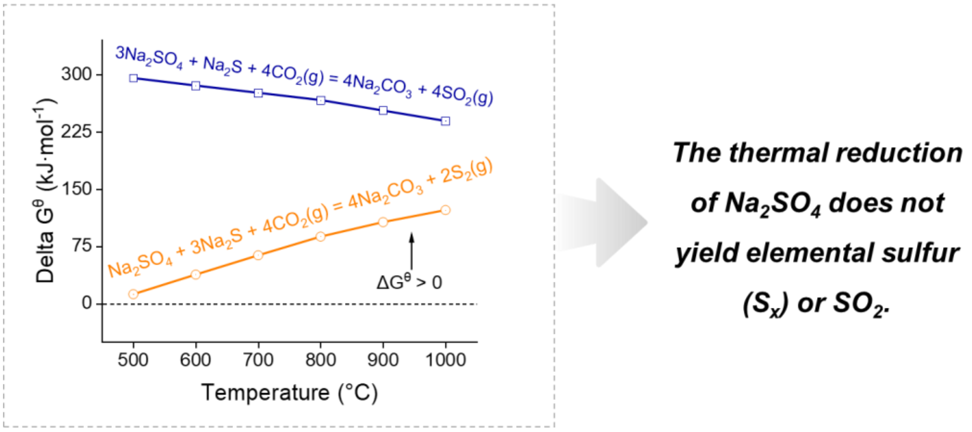

Supplementary Fig. 7 | Thermodynamic unfavourability of volatile sulfur species evolution during carbothermal reduction. The plot illustrates the standard Gibbs free energy (ΔG^{θ}) profiles for potential side reactions generating S_2(g) or SO_2(g) from the Na_2SO_4-Na_2S intermediate system. Calculations were performed using HSC Chemistry 9.0 software under standard pressure (1 atm) (activity: 1, settings: Show Transitions and Criss-Cobble).

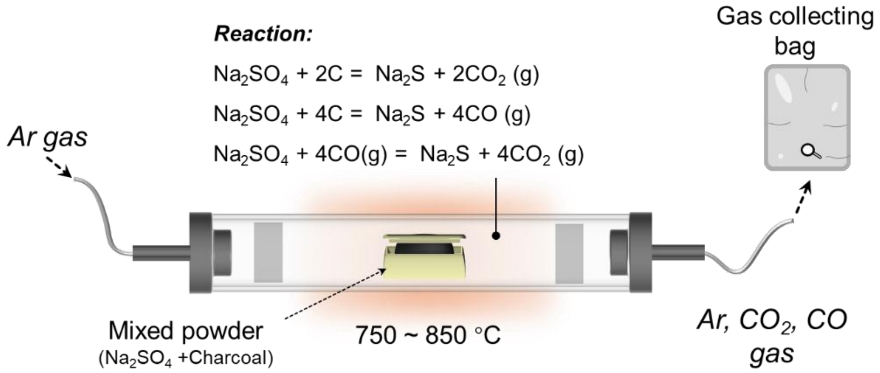

Supplementary Fig. 8 | Experimental setup and reaction scheme for the carbothermal deoxygenation of Na_2SO_4. The schematic illustrates the tube furnace apparatus used for the reduction experiments. A mixture of Na_2SO_4 and C powder is heated under a continuous Ar flow at temperatures between 750 and 800 °C. The evolved gases (CO_2, CO) are collected in a gas collecting bag.

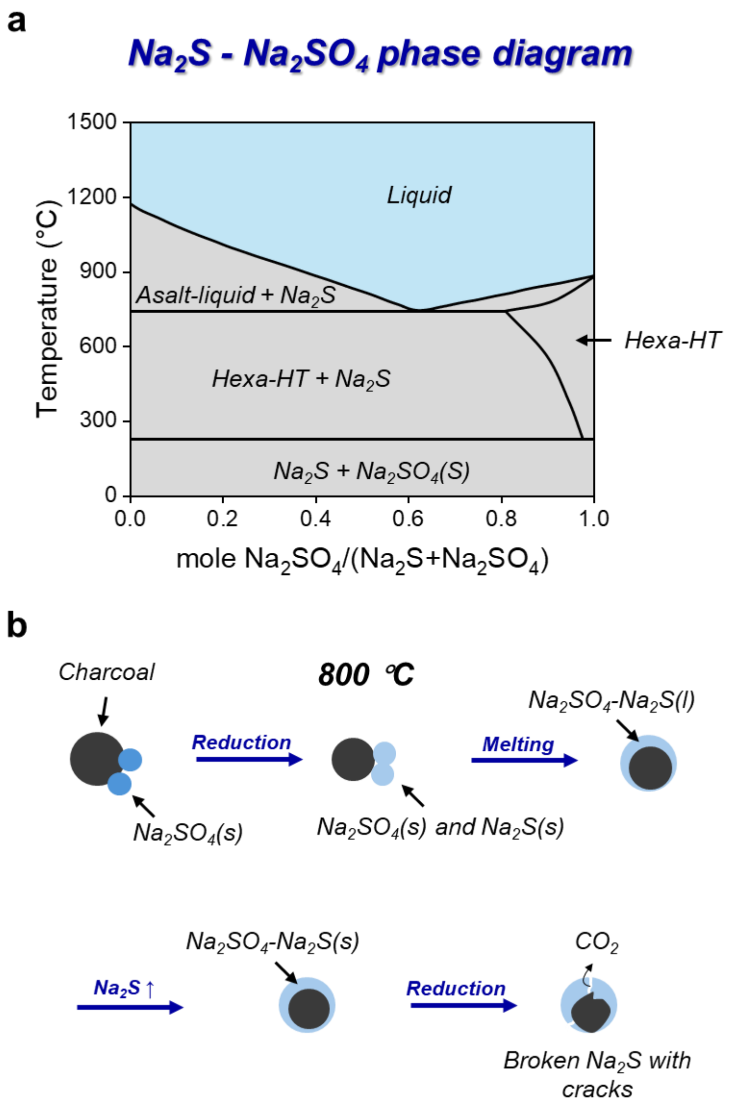

Supplementary Fig. 9 | Phase behavior and schematic diagram of the Na_2S-Na_2SO_4 system during reduction. **a**, Binary phase diagram of the Na_2S-Na_2SO_4 mixture, illustrating the liquidus region and phase transitions at elevated temperatures. **b**, Schematic representation of the carbothermal deoxygenation mechanism at 800 °C. The process depicts the evolution from an initial solid-solid contact to the formation of a molten Na_2S-Na_2SO_4 intermediate phase that wets the C surface, facilitating the deep reduction and ultimate formation of porous solid Na_2S.

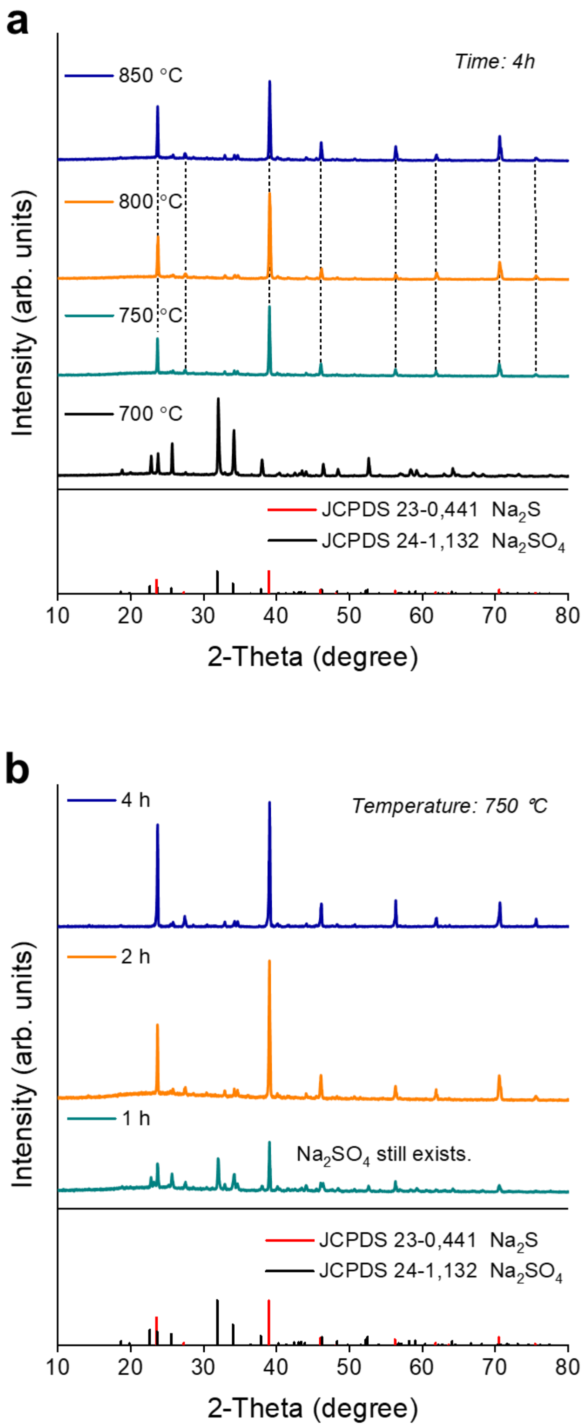

Supplementary Fig. 10 | XRD patterns of the thermochemical deoxygenation products. a, Phase composition of samples treated at different temperatures (holding time: 4 h). **b**, Phase evolution at 750 °C with varying reaction durations.

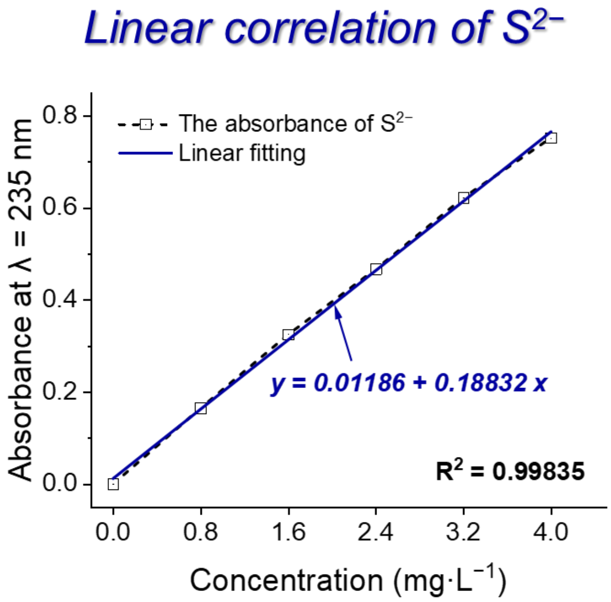

Supplementary Fig. 11 | Calibration curve correlating sulfide ion (S^{2-}) concentration with absorbance at λ = 235 nm.

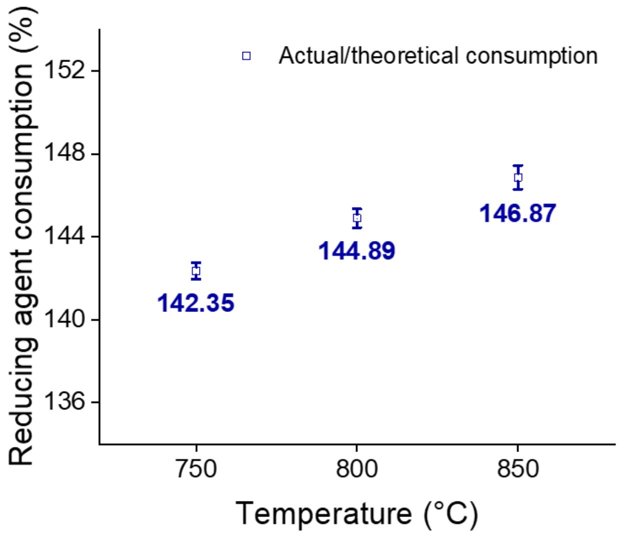

Supplementary Fig. 12 | Temperature-dependent consumption of the reducing agent (C) during the carbothermal deoxygenation of Na_2SO_4. Error bars represent the standard deviation (SD) of n=3 independent experiments.

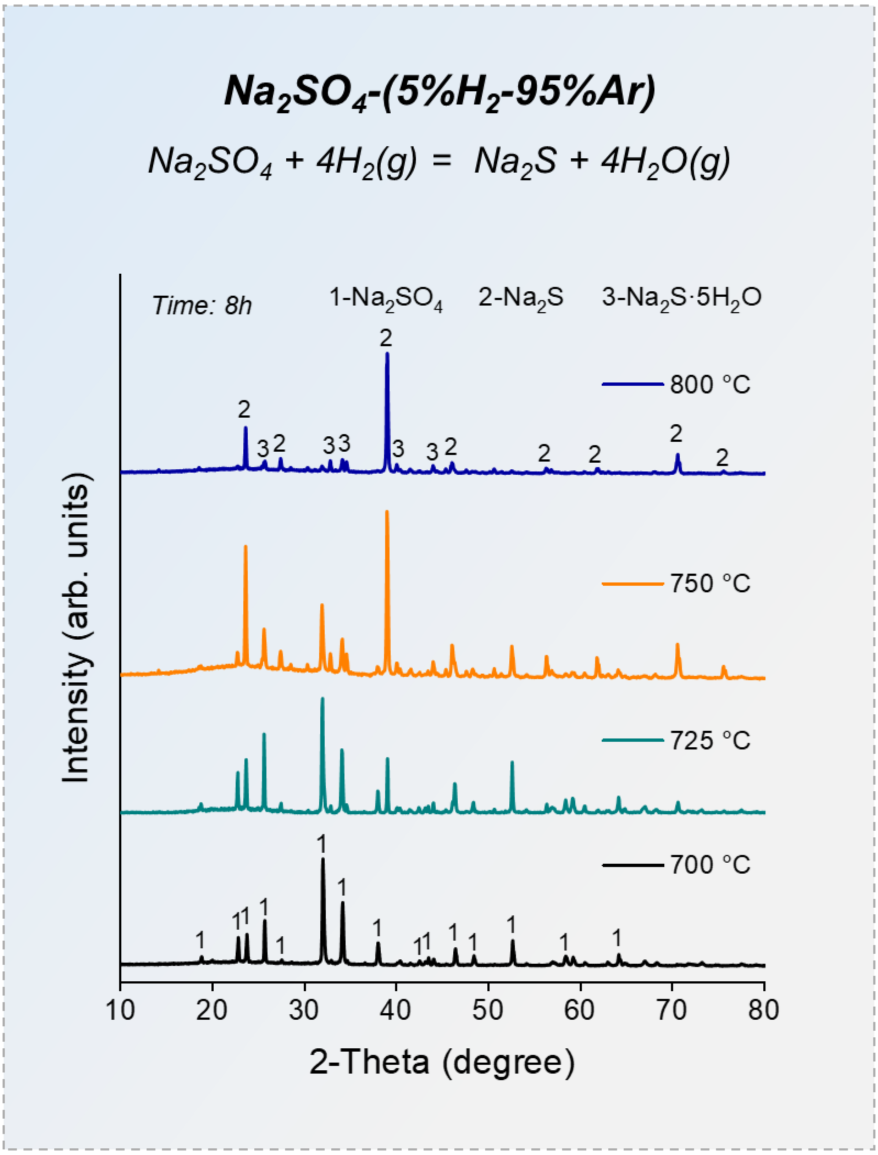

Supplementary Fig. 13 | XRD patterns of products obtained from the H_2-thermochemical deoxygenation of Na_2SO_4 at varying temperatures. Samples were treated for an isothermal duration of 8 h.

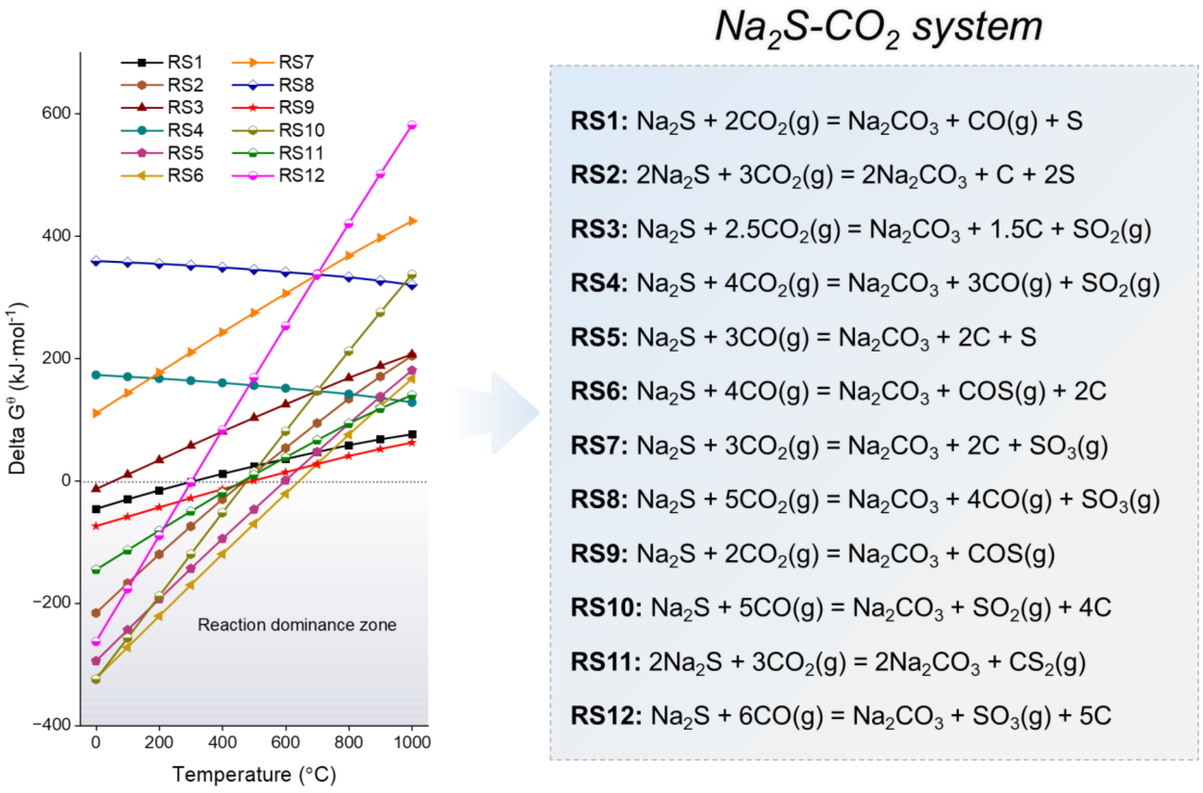

Supplementary Fig. 14 | Standard Gibbs free energy (ΔG^{θ}) profiles for the carbonation. The plot illustrates the temperature-dependent thermodynamic feasibility of the reaction in a pure CO_2 (1 atm). Thermodynamic calculations were performed using HSC Chemistry 9.0 software (activity: 1, settings: Show Transitions and Criss-Cobble).

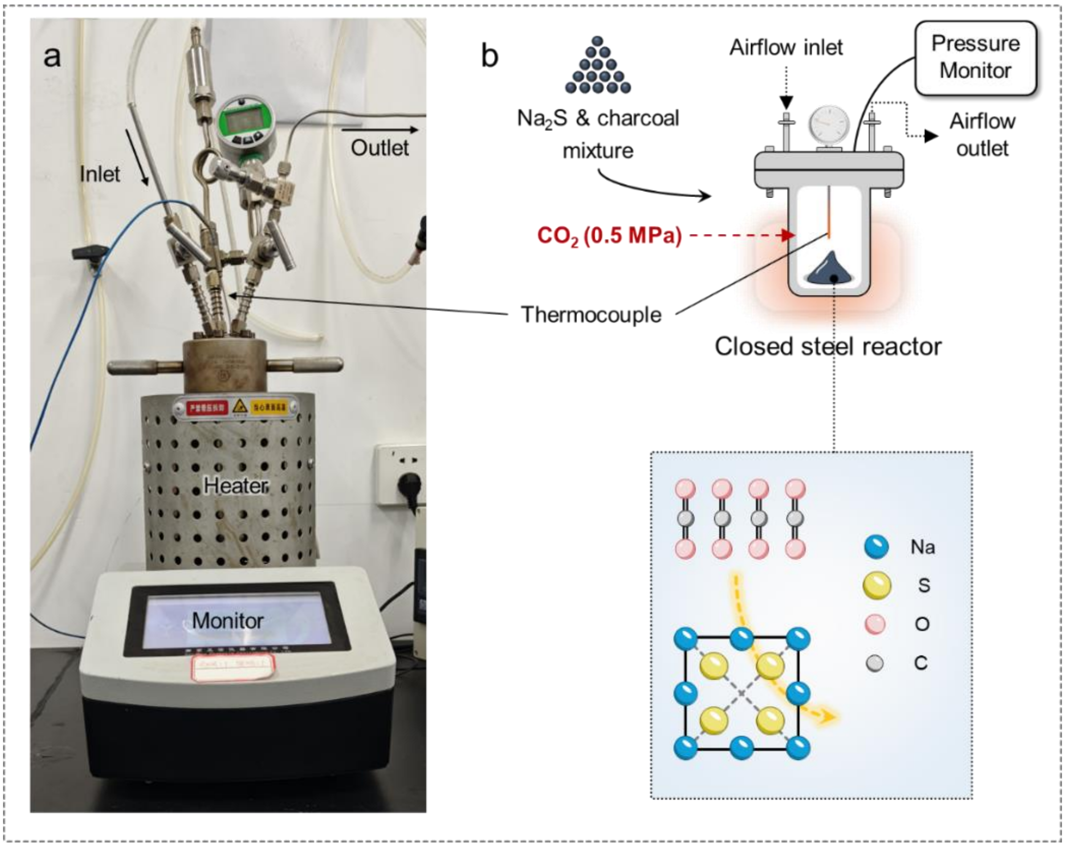

Supplementary Fig. 15 | Experimental apparatus for the carbonation of Na_2S with CO_2. **a**, Photograph of the closed steel reactor assembly used for the high-pressure carbonation experiments. **b**, Schematic illustration of the reaction system and process flow. The diagram depicts the interaction between the Na_2S-charcoal mixture and pressurized CO_2 (0.5 MPa) within the heated vessel, including a conceptual view of the gas-solid reaction interface.

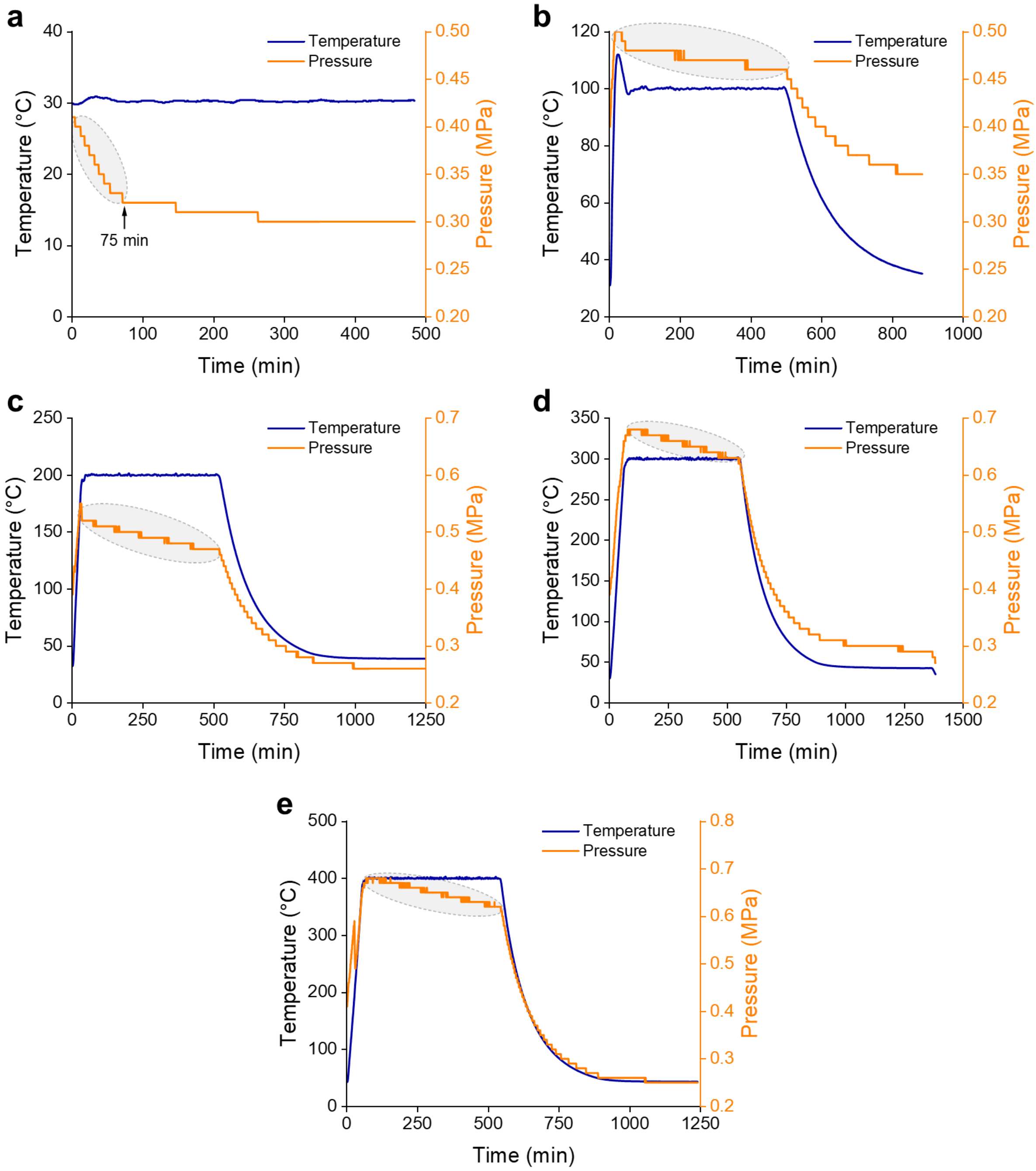

Supplementary Fig. 16 | Temporal evolution of temperature and pressure within the closed steel reactor. a-e, Monitoring profiles recorded at varying target temperatures: room temperature (**a**), 100 °C (**b**), 200 °C (**c**), 300 °C (**d**), and 400 °C (**e**).

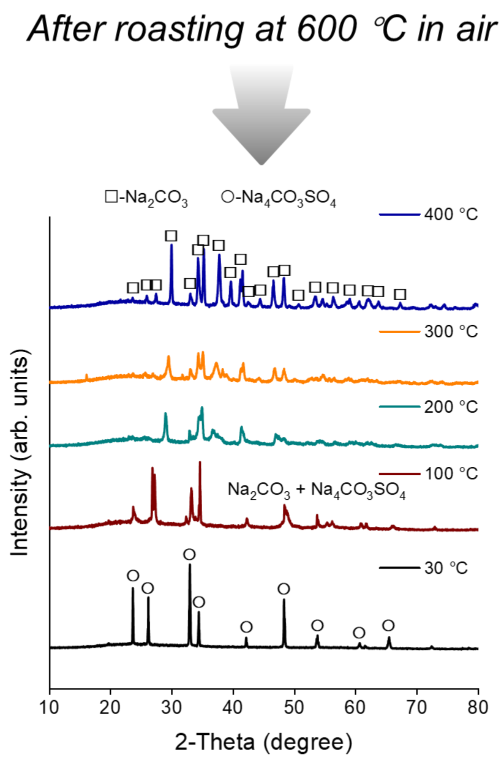

Supplementary Fig. 17 | XRD patterns of the final products after oxidative roasting at 600 °C. The samples were initially carbonated in the closed steel reactor at various temperatures.

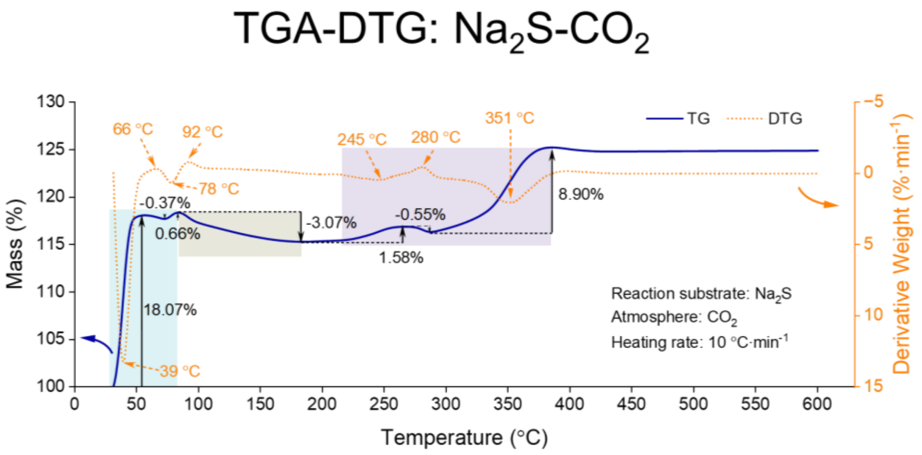

Supplementary Fig. 18 | TGA-DTG profiles of the mixture of Na_2S and charcoal under a CO_2 atmosphere. The sample corresponds to the solid product obtained from the carbothermal reduction of waste Na_2SO_4.

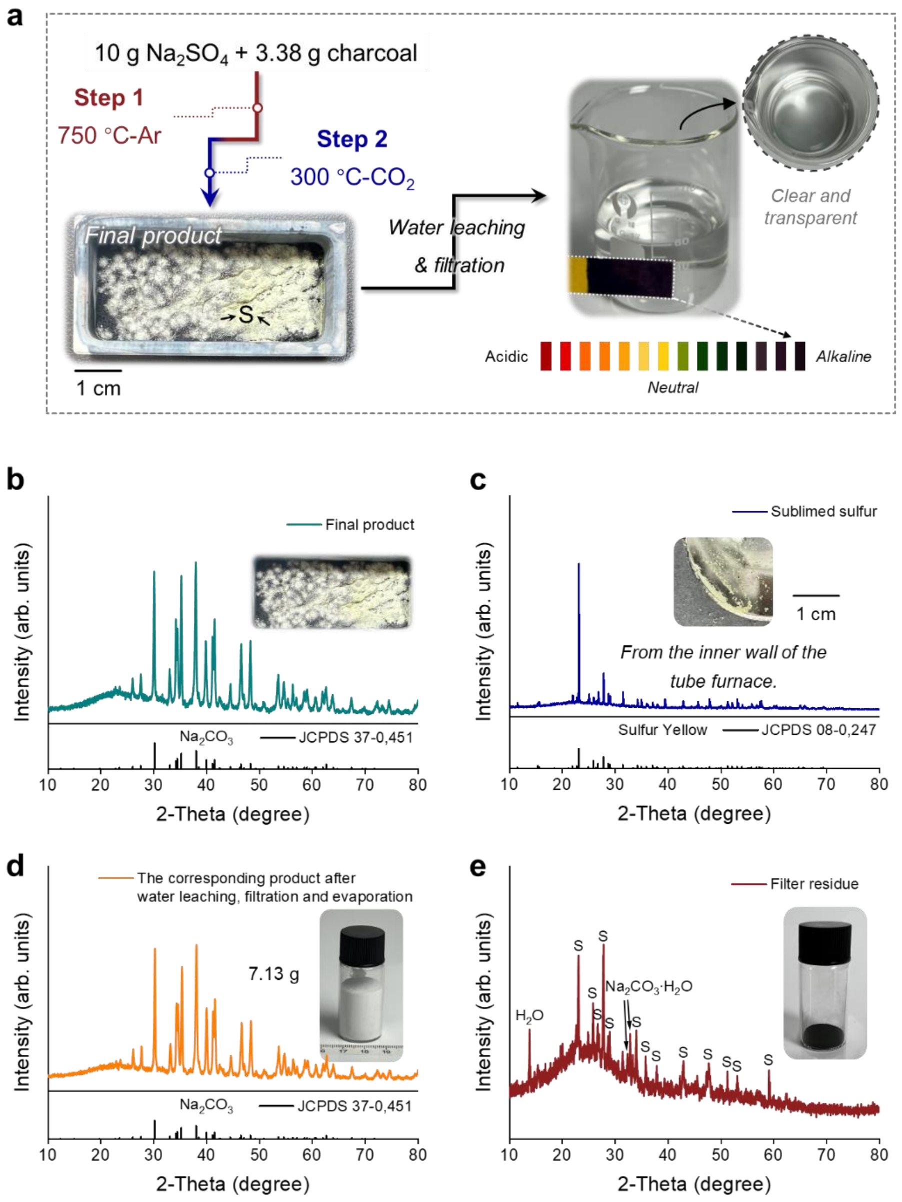

Supplementary Fig. 19 | Conversion of Na_2SO_4 to Na_2CO_3 via a sequential gas-flow strategy. a, Schematic workflow and digital photographs illustrating the two-step process: carbothermal reduction under Ar (750 °C) followed by carbonation under CO_2 (300 °C). **b-e**, XRD patterns of the distinct product fractions: the crude solid product after reaction (**b**), sublimed S_x collected from the reactor walls (**c**), purified Na_2CO_3 obtained after water leaching and evaporation (**d**), and the insoluble filter residue (**e**).

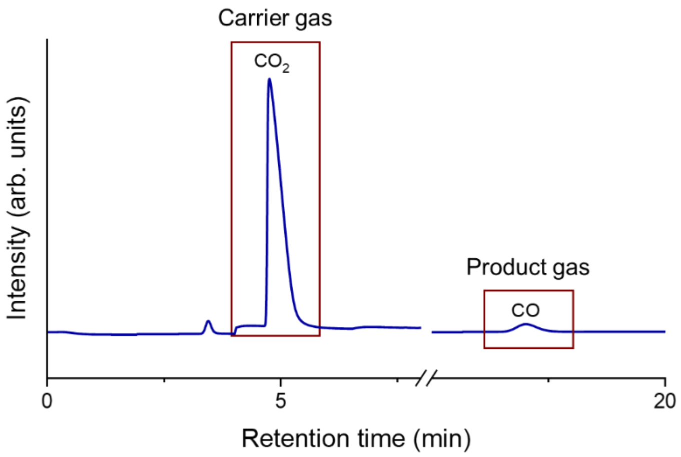

Supplementary Fig. 20 | Gas chromatogram (GC) of the reactor effluent. The sample was analyzed 20 min after switching the gas feed to CO_2 in the continuous flow system.

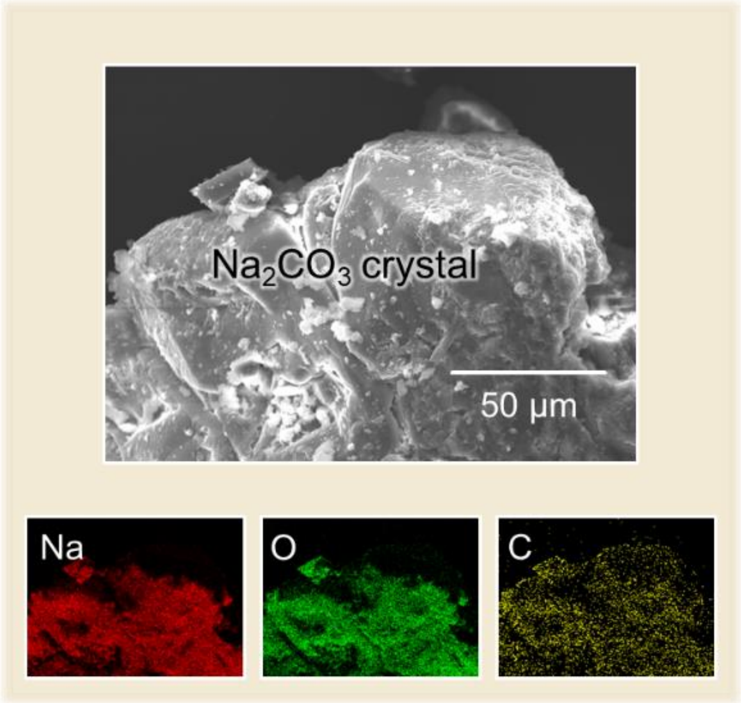

Supplementary Fig. 21 | Morphology and elemental distribution of the synthesized Na_2CO_3 product. SEM) image and corresponding EDS mapping images verify the uniform distribution of Na, O, and C.

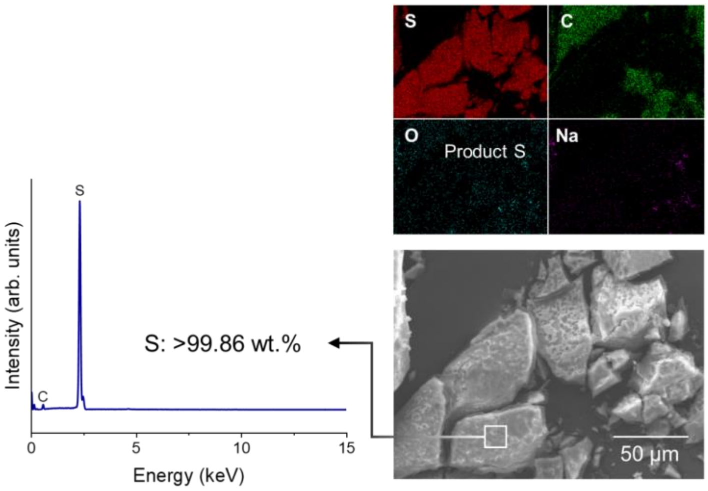

Supplementary Fig. 22 | Morphology and EDS mapping of the S_x product. The analysis confirms a high elemental purity (> 99.86 wt.%). The C signal in the elemental maps originates from the conductive carbon paper substrate rather than impurity contamination.

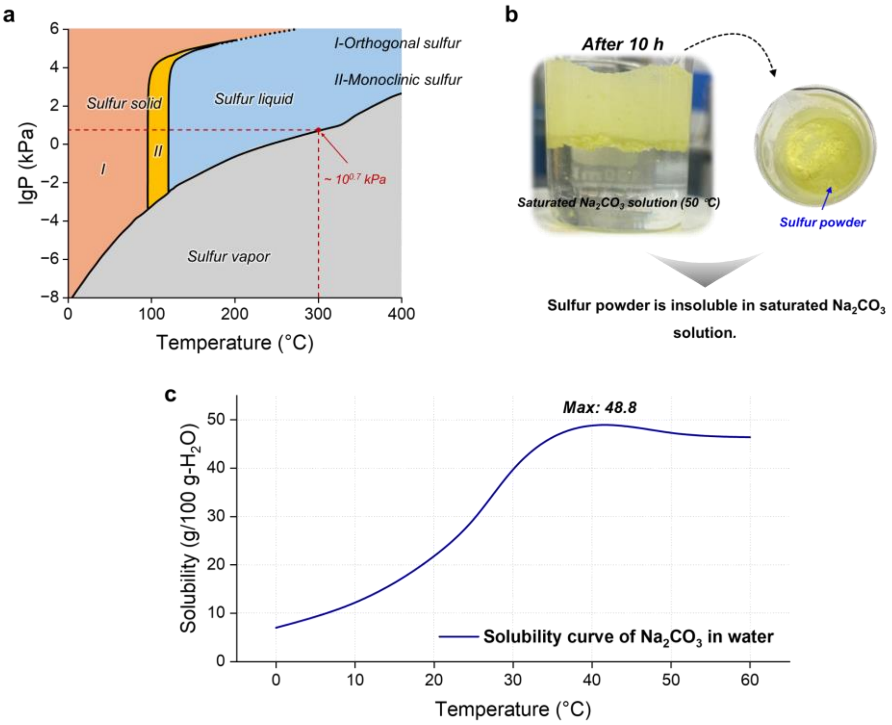

Supplementary Fig. 23 | Physicochemical basis for the separation and recovery of Na_2CO_3 and S_x. a, Phase diagram showing the temperature-dependent saturation vapor pressure of sulfur[34]. The rapid increase in vapor pressure at elevated temperatures facilitates sulfur recovery via sublimation/vaporization **b**, Solubility test demonstrating the

chemical inertness and insolubility of S_x powder in a saturated solution (50 °C) after a 10 h immersion. **c**, Temperature-dependent aqueous solubility profile of Na_2CO_3, indicating high solubility favorable for water leaching.

The effective separation of the reaction products relies on the distinct physicochemical properties of the components. As illustrated in **Supplementary Fig. 23a**, sulfur exhibits a significant rise in saturation vapor pressure with temperature (e.g., transitioning to the vapor phase above 300 °C at reduced pressures), which provides the thermodynamic driving force for its removal via sublimation or evaporation during the heating process. Conversely, the solid residue (Na_2CO_3) is recovered through water leaching. **Supplementary Fig. 23b** confirms that S_x remains insoluble and chemically stable in saturated Na_2CO_3 solutions, preventing cross-contamination during the leaching step. Furthermore, the high aqueous solubility of Na_2CO_3 (**Supplementary Fig. 23c**, reaching 48.8 g/100g water) ensures efficient recovery and crystallization of the final carbonate product.

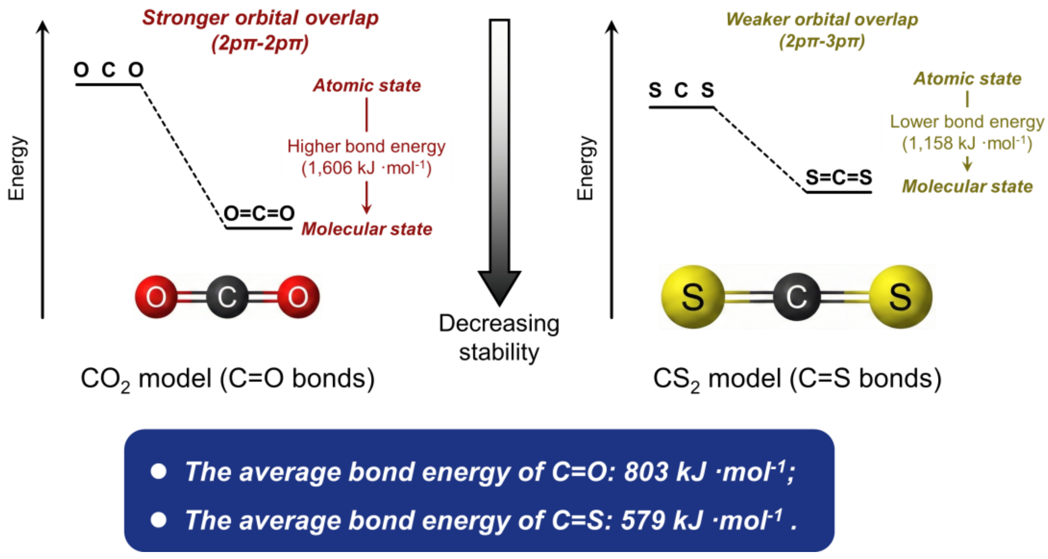

Supplementary Fig. 24 | Comparison of thermodynamic stability and overlapping efficiency between CO_2 (O=C=O) and CS_2 (S=C=S).

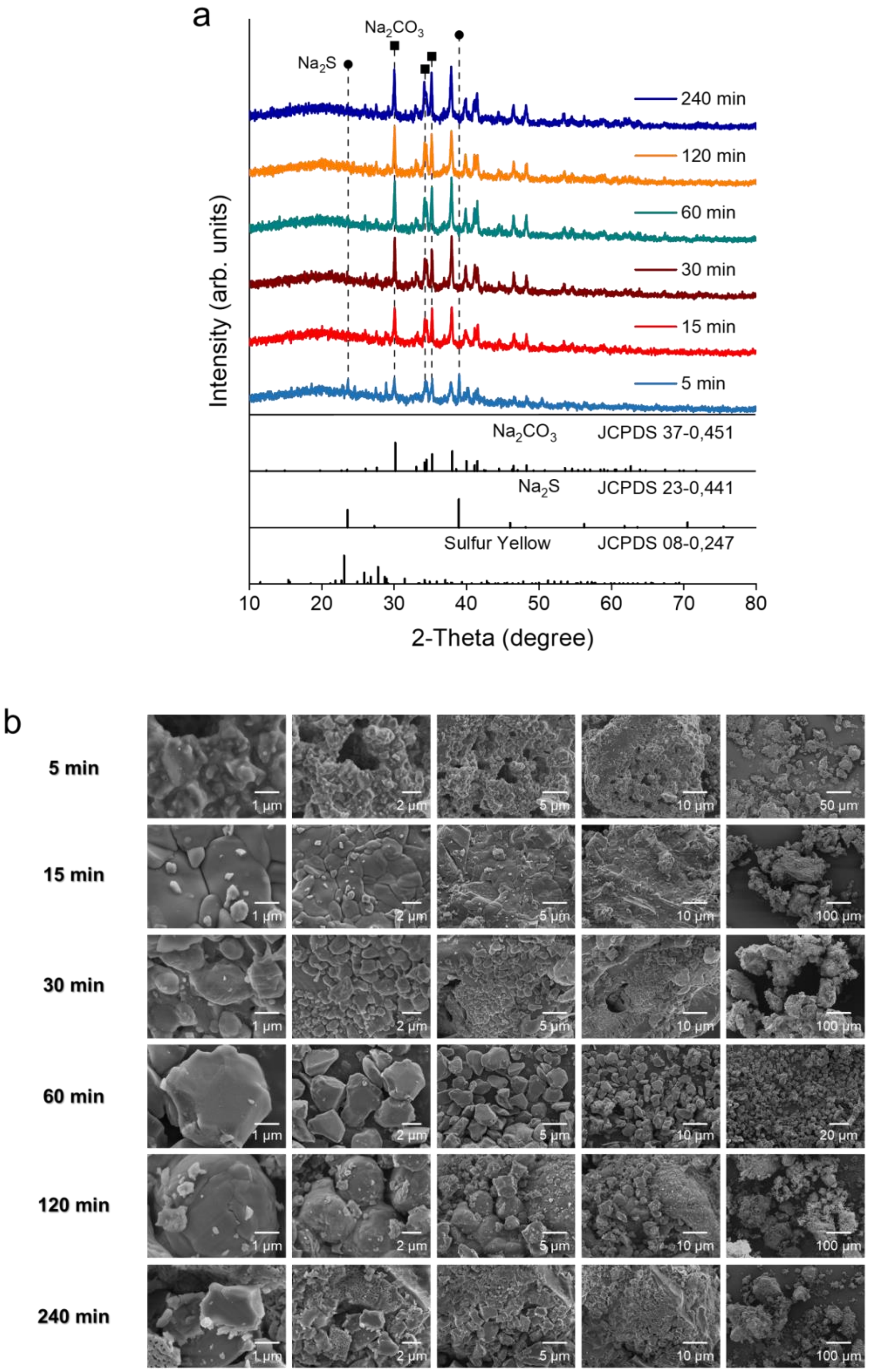

Supplementary Fig. 25 | Structural and morphological evolution of during carbonation. XRD patterns and SEM images of samples treated at 350 °C under a continuous CO_2 flow (100 mL·min^{-1}) for varying durations.

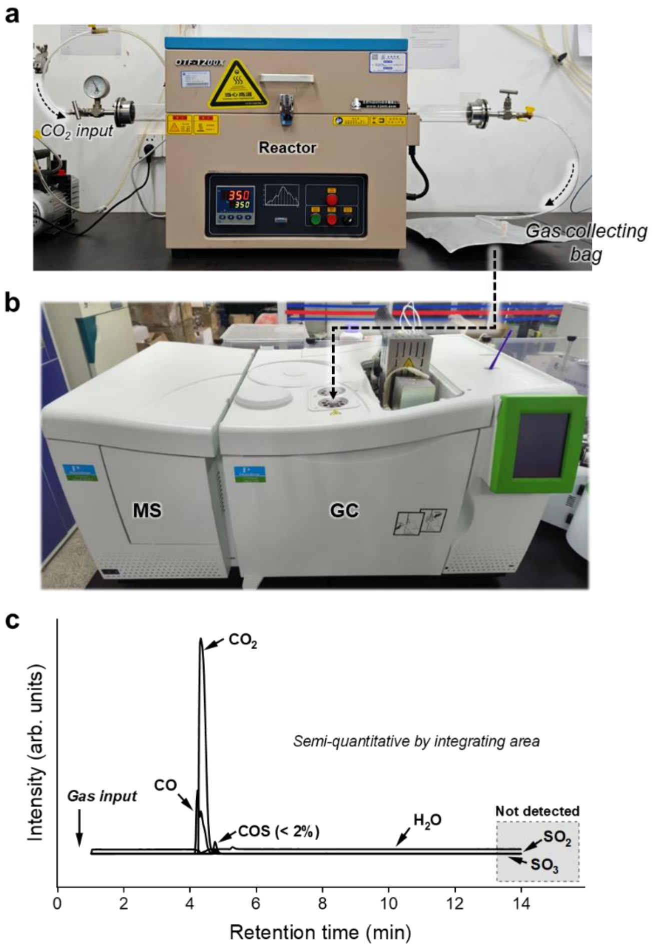

Supplementary Fig. 26 | Investigation of potential side reactions between Na_2S and CO_2. **a**, Digital photograph of the experimental setup used for reaction gas collection. **b**, The GC-MS instrument. **c**, Total ion chromatogram (TIC) identifying the gaseous byproducts. Test conditions: The system was operated in full scan mode to capture the complete spectrum of each data point during the chromatographic analysis. A 1 μL gas sample was injected into the chromatographic column with He (99.999%) as the carrier gas at a flow rate of 1.0 mL·min^{-1}. The temperature program involved holding at 40 °C for 2 min, followed by a ramp to 300 °C at a rate of 8 °C·min^{-1}. The mass selective detector's electron impact (EI) ion source temperature was set at 200 °C, and the mass spectra were analyzed in the range of 0 – 300 atomic mass units (amu).

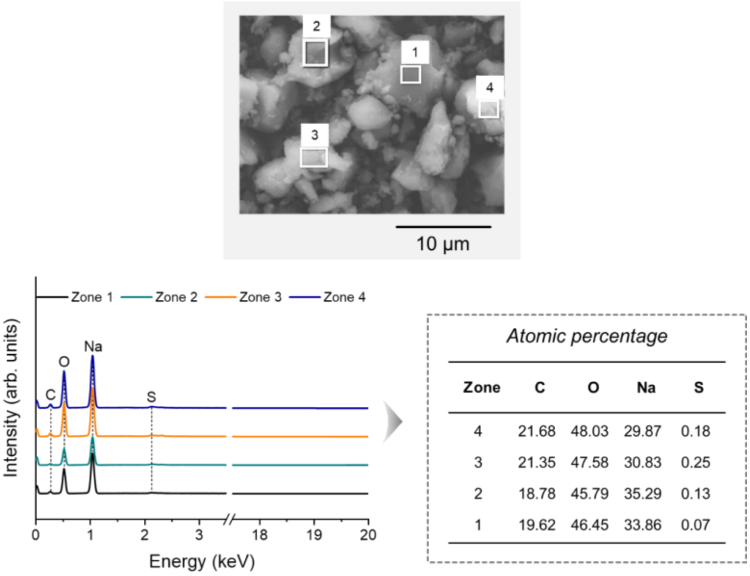

Atomic percentage

Zone	C	O	Na	S
4	21.68	48.03	29.87	0.18
3	21.35	47.58	30.83	0.25
2	18.78	45.79	35.29	0.13
1	19.62	46.45	33.86	0.07

Supplementary Fig. 27 | SEM morphology and EDS elemental analysis (S, C, O, and Na) of the final product obtained from the scale-up production.

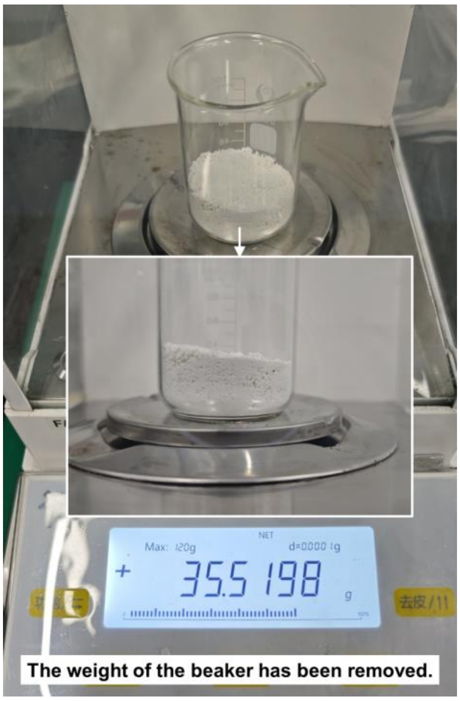

Supplementary Fig. 28 | Optical photograph of the purified Na_2CO_3 powder recovered from 50 g of waste Na_2SO_4. The digital balance indicates a final mass of approximately 35.52 g from the scale-up production.

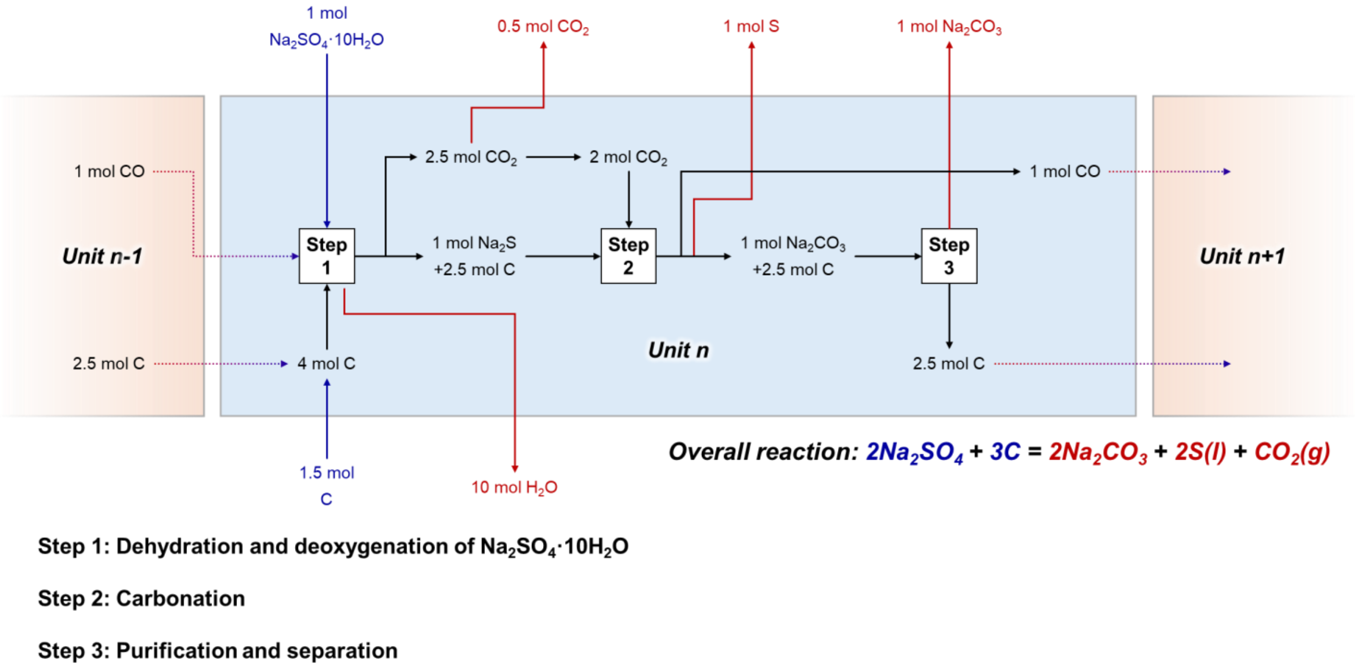

Supplementary Fig. 29 | System boundary and process flow for the thermochemical conversion of waste Na_2SO_4 to Na_2CO_3 (this study). In the *n*-th cycle, both CO and C are assumed to undergo complete oxidation to CO_2 to maximize reductant utilization efficiency. Consequently, the carbon consumption in Unit *n* is calculated based on the stoichiometry of the overall reaction depicted in the figure.

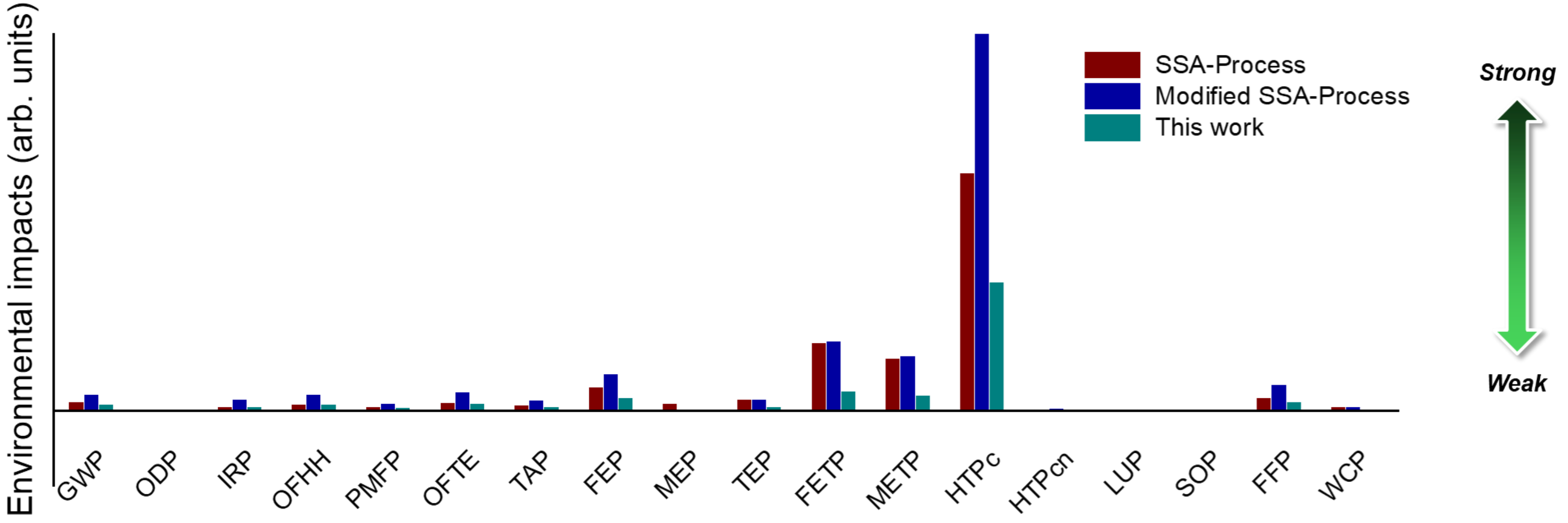

GWP-global warming potential
ODP-stratospheric ozone depletion
IRP-ionizing radiation
OFHH-ozone formation, human health
PMFP-fine particulate matter formation
OFTE-ozone formation, terrestrial ecosystems
TAP-terrestrial acidification
FEP-freshwater eutrophication
MEP-marine eutrophication
TEP-terrestrial ecotoxicity
FETP-freshwater ecotoxicity
METP-marine ecotoxicity
HTPc-human carcinogenic toxicity
HTPcn-human noncarcinogenic toxicity
LUP-land use potential
SOP-mineral resource scarcity
FFP-fossil resource scarcity
WCP-water consumption

Supplementary Fig. 30 | Normalized environmental impact profiles of the SSA-Process, modified SSA-Process, and the proposed method (this work).

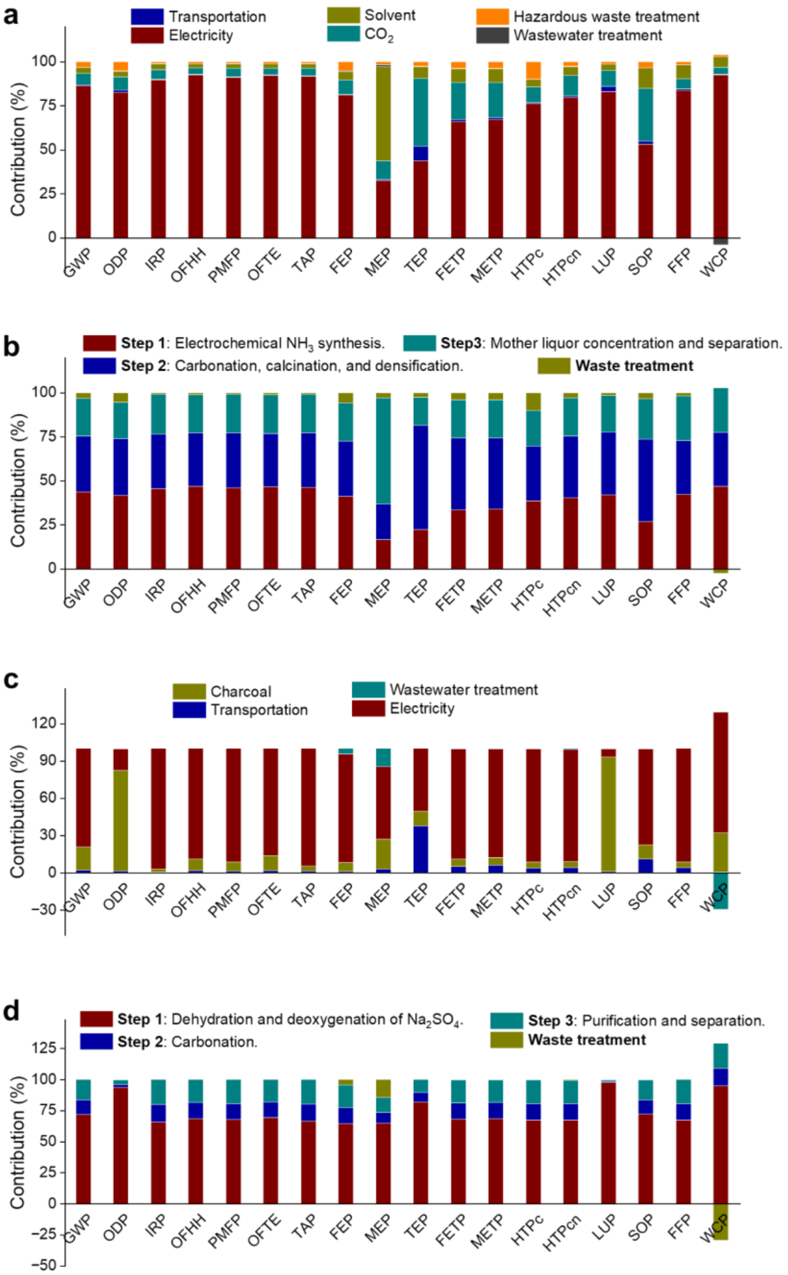

Supplementary Fig. 31 | Contribution analysis of mid-point environmental impact categories. a-b, Contribution analysis for the modified SSA-Process, broken down by specific inventory inputs (**a**) and unit processes/production steps (**b**). **c-d**, Contribution analysis for the proposed thermochemical route (this work), broken down by specific inventory inputs (**c**) and unit processes/production steps (**d**).

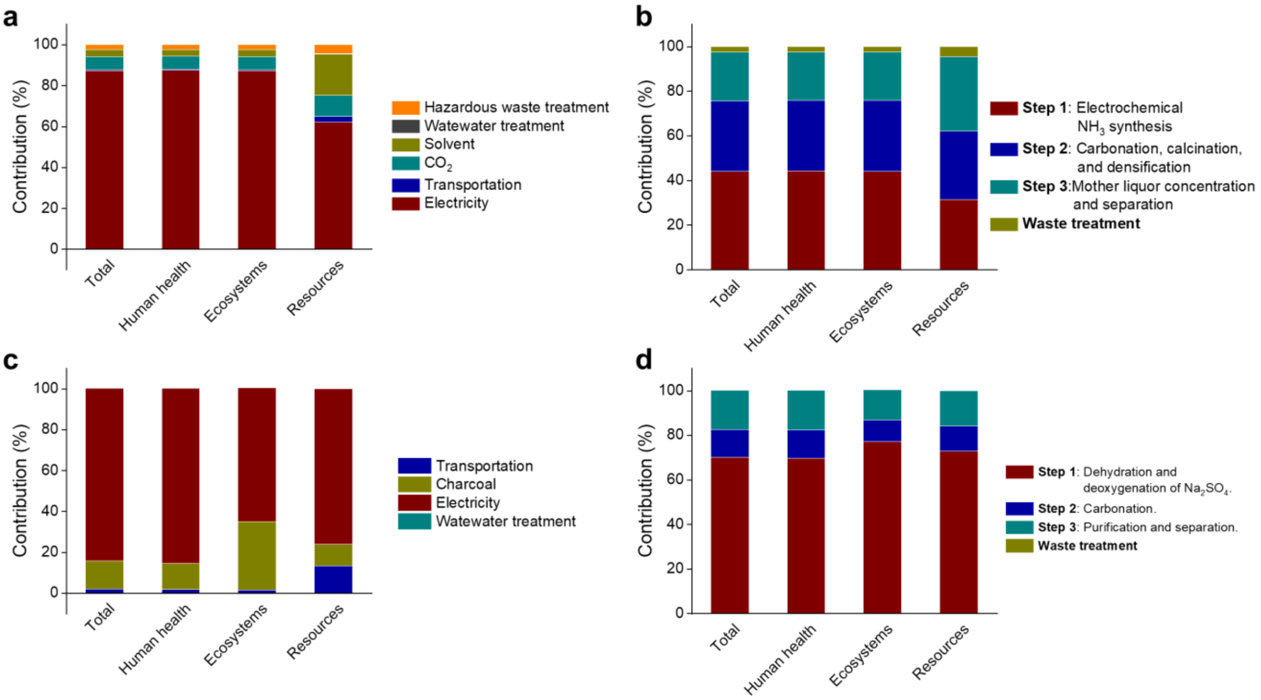

Supplementary Fig. 32 | Contribution analysis of end-point environmental impact categories. a-b, Contribution analysis for the modified SSA-Process, broken down by specific inventory inputs (**a**) and unit processes/production steps (**b**). **c-d**, Contribution analysis for the proposed thermochemical route (this work), broken down by specific inventory inputs (**c**) and unit processes/production steps (**d**).

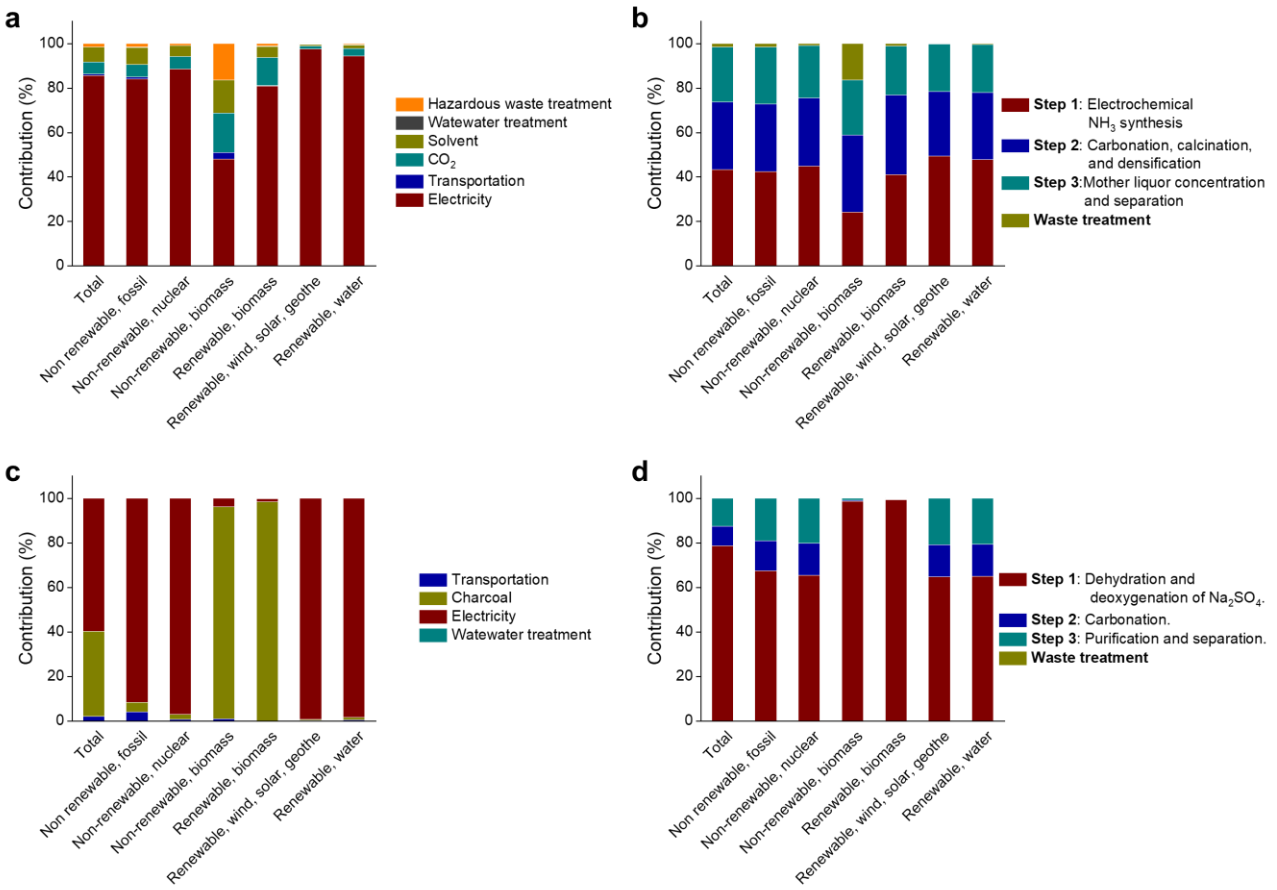

Supplementary Fig. 33 | Contribution analysis of CED. a-b, Contribution analysis for the modified SSA-Process, broken down by specific inventory inputs (**a**) and unit processes/production steps (**b**). **c-d**, Contribution analysis for the proposed thermochemical route (this work), broken down by specific inventory inputs (**c**) and unit processes/production steps (**d**).

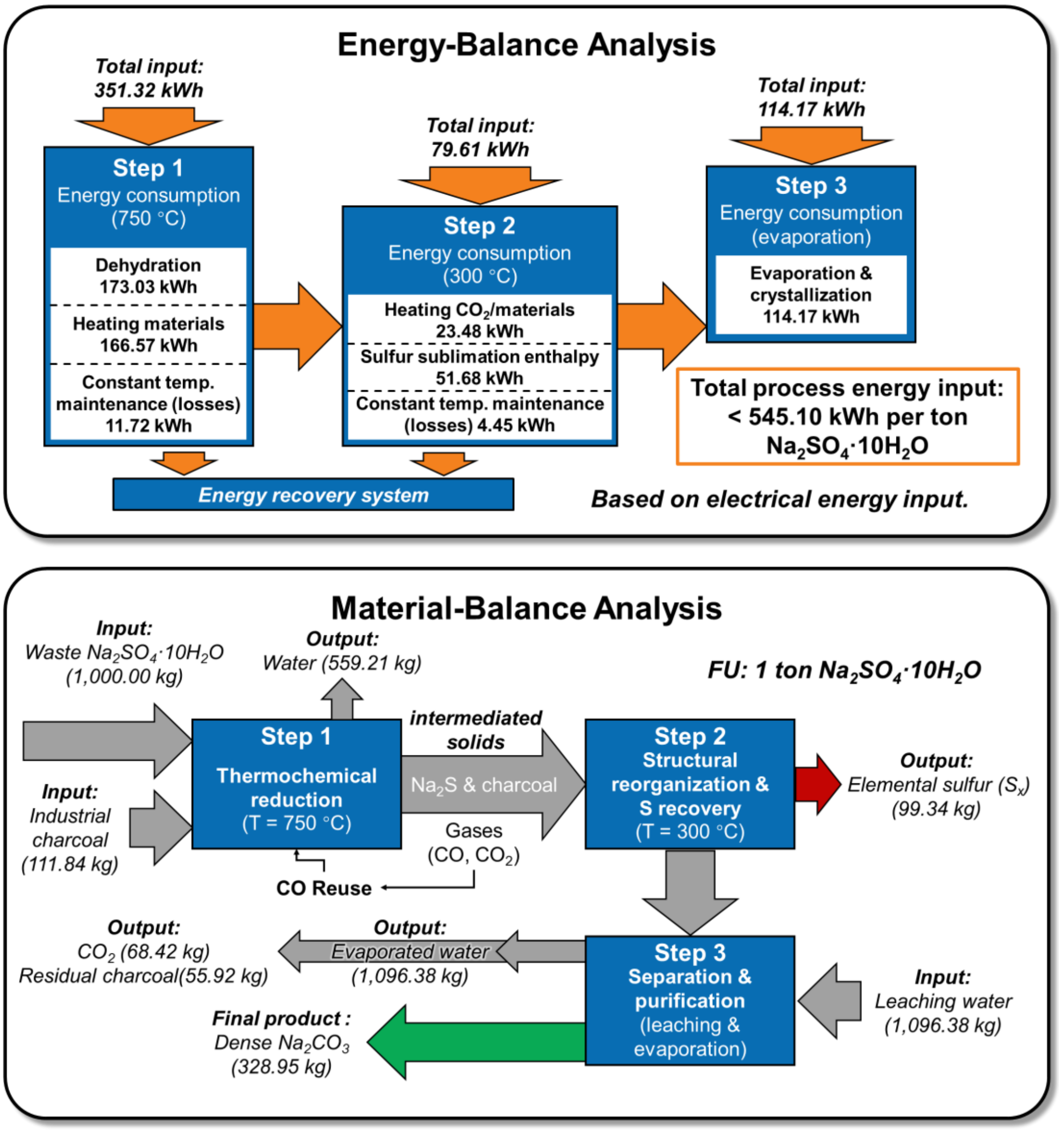

Supplementary Fig. 34 | Energy- and material-balance analysis for thermochemical upcycling of 1,000 kg waste $Na_2SO_4·10H_2O$. The energy-balance analysis reveals that the total process energy input is < 545.10 kWh per ton of $Na_2SO_4·10H_2O$. Notably, the dehydration (**Step 1**, 173.03 kWh) and evaporation (**Step 3**, 114.17 kWh) processes collectively account for approximately 52.7% of the total energy consumption. As these steps are driven primarily by thermal demand, they offer a significant avenue for decarbonization by coupling with intermittent renewable sources, such as solar thermal energy or industrial waste heat, instead of relying solely on grid electricity.

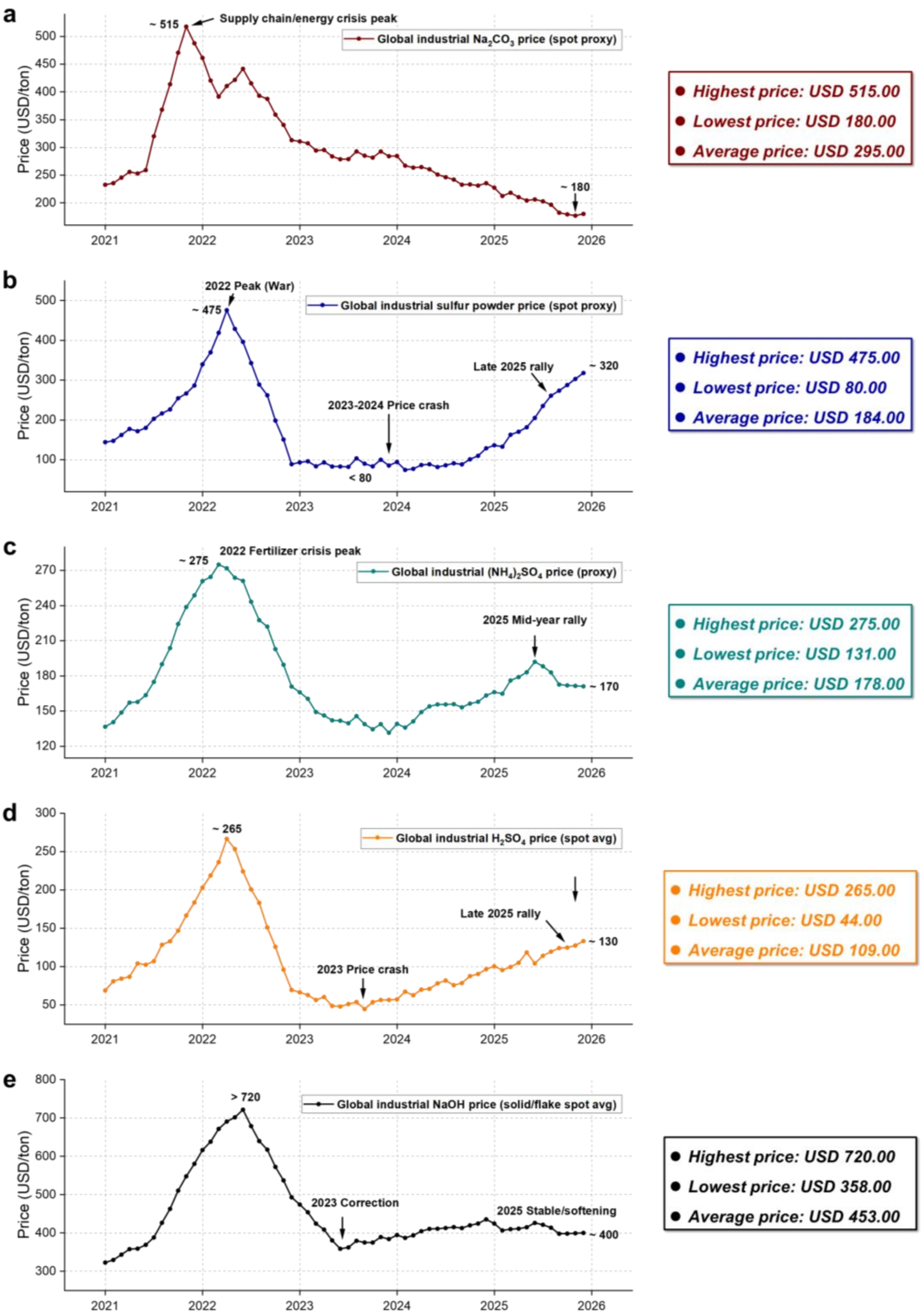

Supplementary Fig. 35 | The global price trend of the relevant products in the past five years. a, Na_2CO_3. **b**, Sulfur powder. **c**, $(NH_4)_2SO_4$. **d**, H_2SO_4. **e**, NaOH.

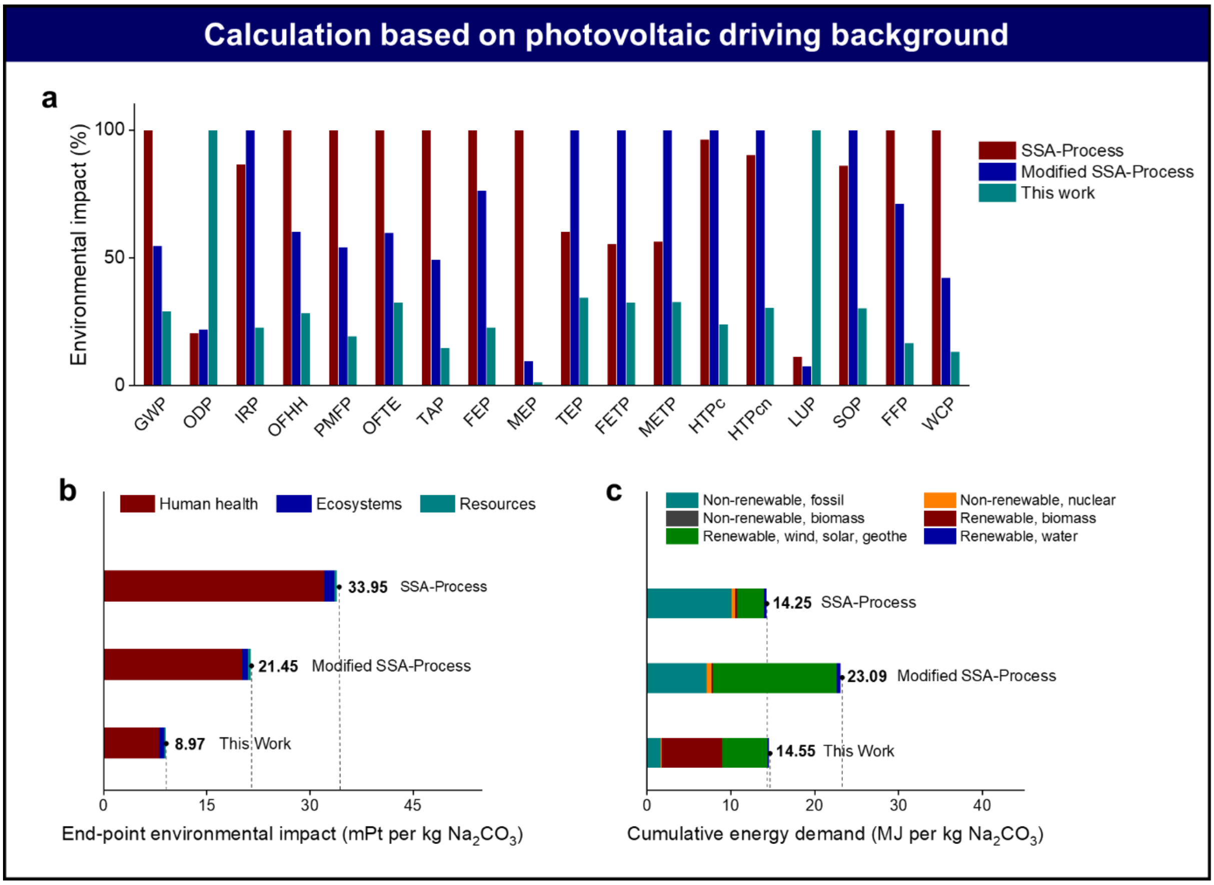

Supplementary Fig. 36 | Comparative assessment of environmental impacts and CED under the PV-driven scenario. a, Comparison of mid-point environmental impact categories for the SSA-Process, modified SSA-Process, and the proposed thermochemical route (this work). **b**, Comparison of end-point environmental impact results (measured in mPt). **c**, Comparison of CED results (measured in MJ).

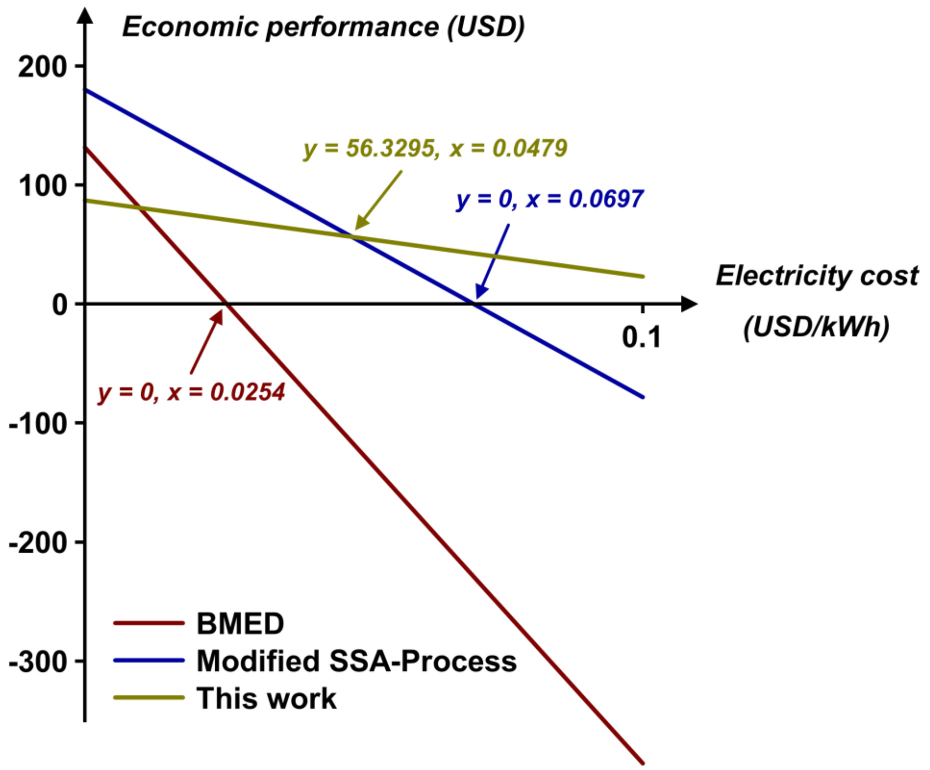

Supplementary Fig. 37 | Sensitivity analysis of economic performance to electricity price fluctuations. The linear relationship between net profit (USD) and electricity cost (USD/kWh) is compared for the BMED process (red), the modified SSA-Process (blue), and the proposed thermochemical route (this work, olive). To align with the comprehensive LCC methodology, the net profit (y) is mathematically defined as a function of the electricity price (x) using the equation: $y = \textbf{Total Revenue} - (\boldsymbol{E_{consumption}} \times x + \textbf{Other OPEX})/0.85$. In this model, $E_{consumption}$ is the electricity demand, Other OPEX encompasses other variable material costs, and the 0.85 denominator integrates the 15% fixed cost allowance (which includes annualized CAPEX).

The arrows indicate the break-even points where net profit equals zero. These occur at $x = 0.0254$ USD/kWh for the BMED process and $x = 0.0697$ USD/kWh for the modified SSA-Process. A critical crossover threshold is identified at $x = 0.0479$ USD/kWh ($y = 56.33$ USD). Beyond this specific electricity price, the proposed route becomes definitively superior to the modified SSA-Process in terms of economic viability. The significantly flatter slope of the proposed route visually demonstrates its robust resilience against rising energy costs compared to the highly electricity-intensive BMED and modified SSA-Processes.

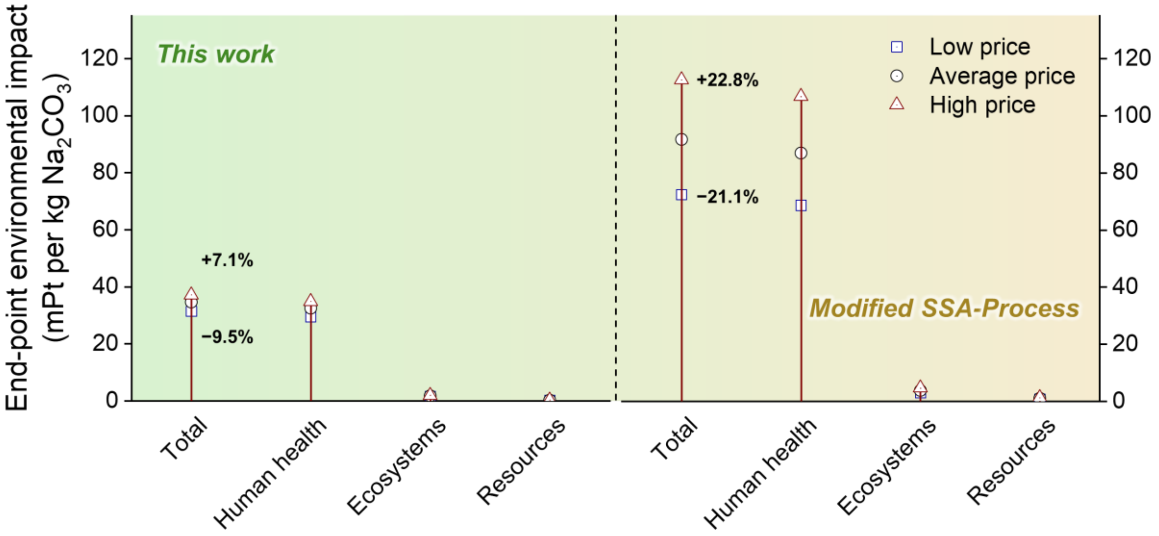

This work

Damage category	Low price	Average price	High price
Total	31.39	34.69	37.17
Human health	29.45348147	32.55384782	34.87912279
Ecosystems	1.660802689	1.835624023	1.966740027
Resources	0.272228208	0.300883806	0.322375509

Note: The distribution coefficients of Na_2CO_3 products under the lowest, average and highest prices are 76%, 84%, and 90% respectively.

Modified SSA-Process

Damage category	Low price	Average price	High price
Total	72.41	91.71	112.63
Human health	68.64450725	86.94970921	106.7803446
Ecosystems	2.969154746	3.760929346	4.618685161
Resources	0.791411555	1.002454636	1.231084641

Note: The distribution coefficients of Na_2CO_3 products under the lowest, average and highest prices are 45%, 57%, and 70%respectively.

Supplementary Fig. 38 | Impact of Na_2CO_3 price volatility on end-point environmental performance. Comparison of endpoint environmental impacts between the proposed thermochemical route (this work) and the modified SSA-Process under three price scenarios: low (blue squares), average (black circles), and high (red triangles). The percentages indicate the deviation from the baseline (average price) scenario.

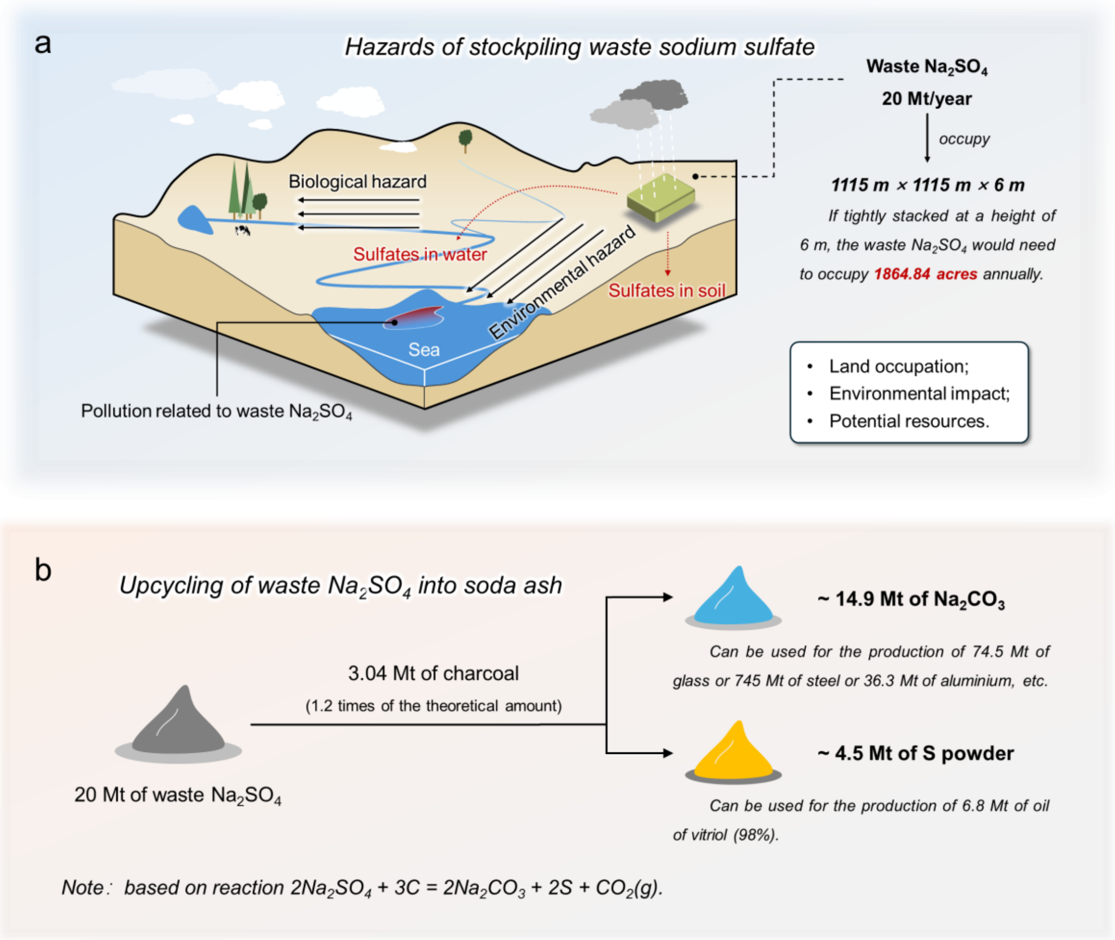

Supplementary Fig. 39 | Environmental implications and upcycling potential of waste Na_2SO_4. a, Schematic illustration of the environmental hazards associated with stockpiling waste Na_2SO_4, including land occupation (estimated at ~1,865 acres annually based on 20 Mt) and potential soil and water contamination. **b**, The projected material flow for the global valorization of 20 Mt of waste Na_2SO_4 using the proposed thermochemical route, theoretically yielding approximately 14.9 Mt of dense Na_2CO_3 and 4.5 Mt of S_x powder for downstream industrial applications.

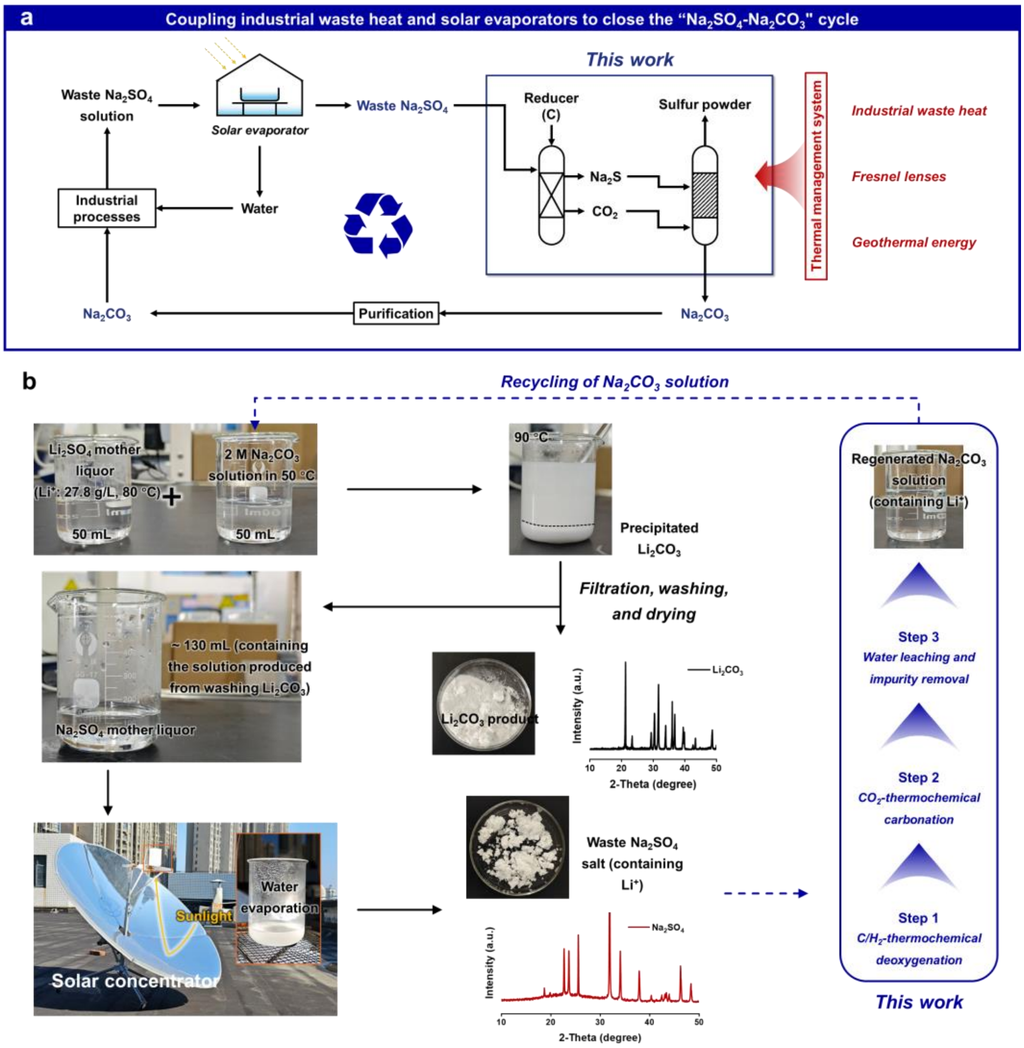

Supplementary Fig. 40 | Strategies for closing the "Na_2SO_4-Na_2CO_3" loop via multi-energy integration. a, Schematic illustration of the synergistic coupling of industrial waste heat and solar evaporators to achieve a sustainable Na_2SO_4-Na_2CO_3 cycle. The system integrates solar thermal energy (e.g., Fresnel lenses) and geothermal energy to optimize thermal management. **b**, Practical demonstration of this cycle applied to lithium extraction. The process involves the precipitation of Li_2CO_3 followed by the concentration of the Na_2SO_4 mother liquor using a solar concentrator. The recovered Na_2SO_4 solid is then processed via the proposed thermochemical route to regenerate the Na_2CO_3 solution for reuse.

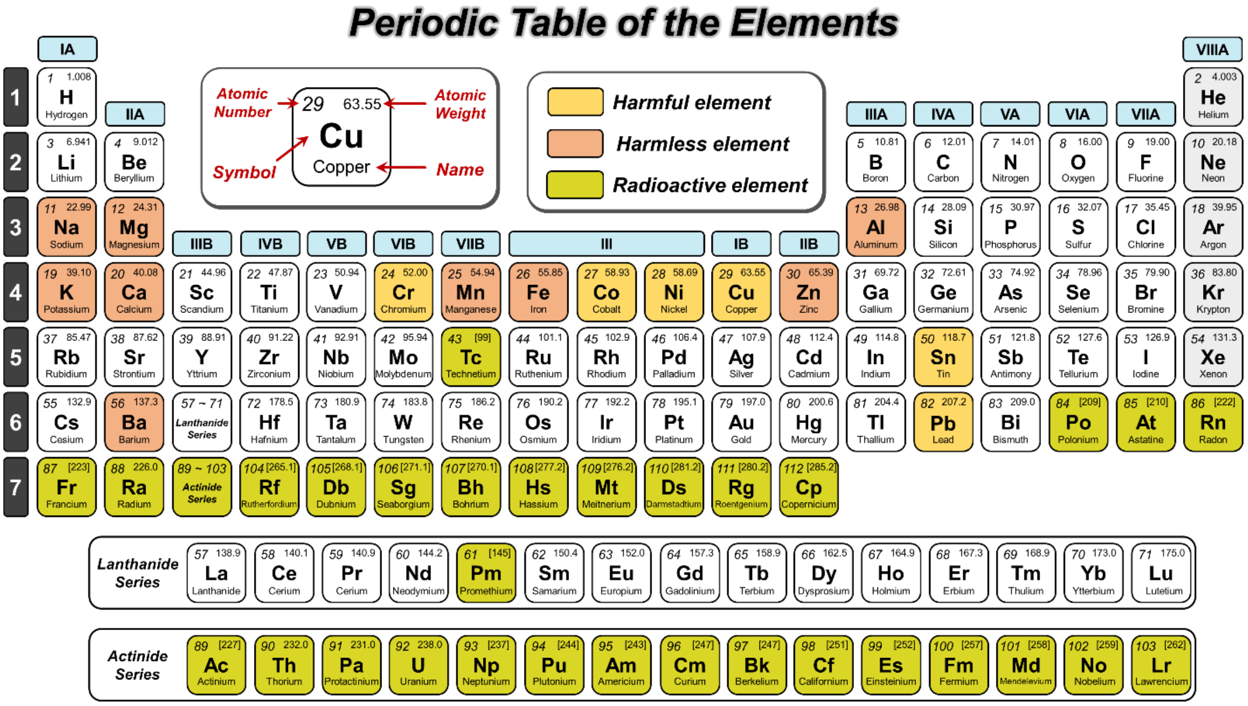

Supplementary Fig. 41 | Overview of typical waste sulfates (e.g., Na_2SO_4, $CaSO_4$, $FeSO_4$) identified as potential renewable resources. Beyond Na_2SO_4, there are diverse sulfate byproducts in many industrial processes, such as $FeSO_4$ from TiO_2 manufacturing, $CaSO_4$ from phosphate extraction, and $NiSO_4$ from spent LIBs. The thermochemical route proposed herein can serve as a versatile "soda regeneration hub" coupled with a pre-treatment strategy we term "alkaline sodiation." Taking the ubiquitous $FeSO_4$ as an example: direct carbothermal reduction typically releases hazardous SO_2 gas. However, by introducing Na-based alkalis (Na_2CO_3 or NaOH), complex sulfates can be converted into valuable metal oxides (e.g., Fe_xO_y for metallurgical feedstocks) and Na_2SO_4. This intermediate Na_2SO_4 is subsequently processed via our technology to regenerate the soda ash reagents required for the initial conversion and to recover S_x. This synergistic integration allows for the closed-loop valorization of a wide spectrum of sulfate wastes, avoiding SO_2 emissions while recovering critical metals and sulfur resources.

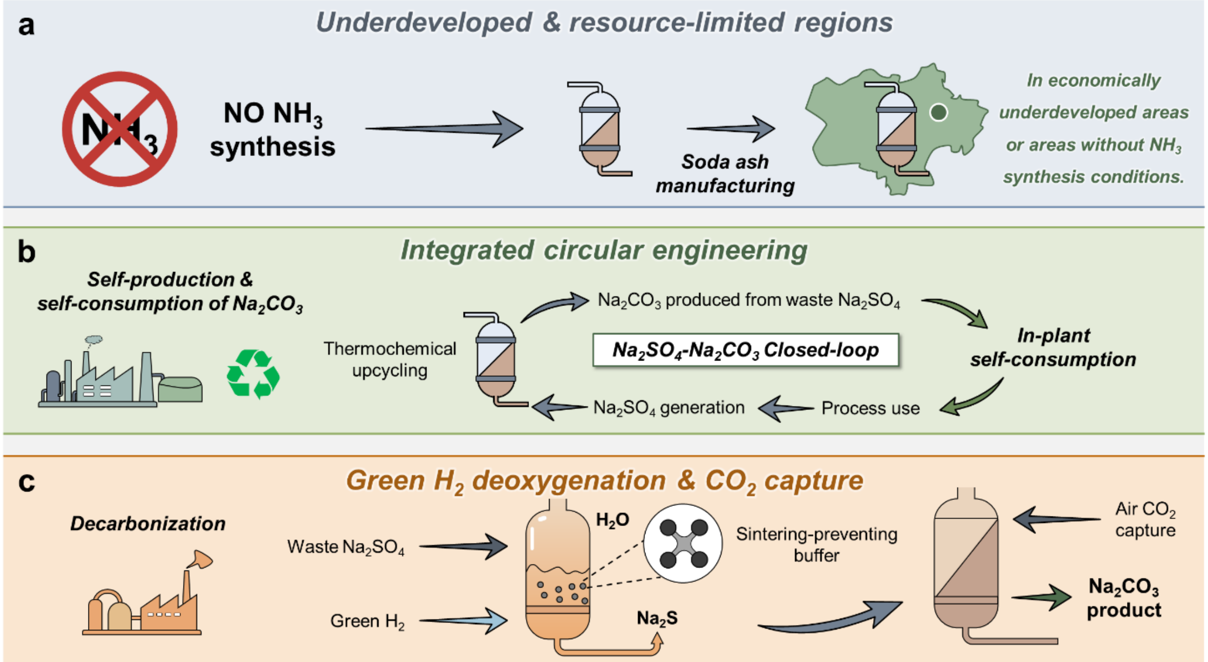

Supplementary Fig. 42 | Future perspectives on industrial deployment and low-carbon evolution of the proposed technology. a, Deployment feasibility for economically underdeveloped regions or areas lacking synthetic NH_3 infrastructure, enabling decentralized, NH_3-free soda ash manufacturing. **b**, Strategy for integrated circular engineering to establish an on-site Na_2SO_4-Na_2CO_3 closed-loop, realizing internal self-sufficiency. **c**, Advanced decarbonization pathway coupling green H_2-based deoxygenation (utilizing sintering-preventing buffers in a fluidized bed) with direct air CO_2 capture.

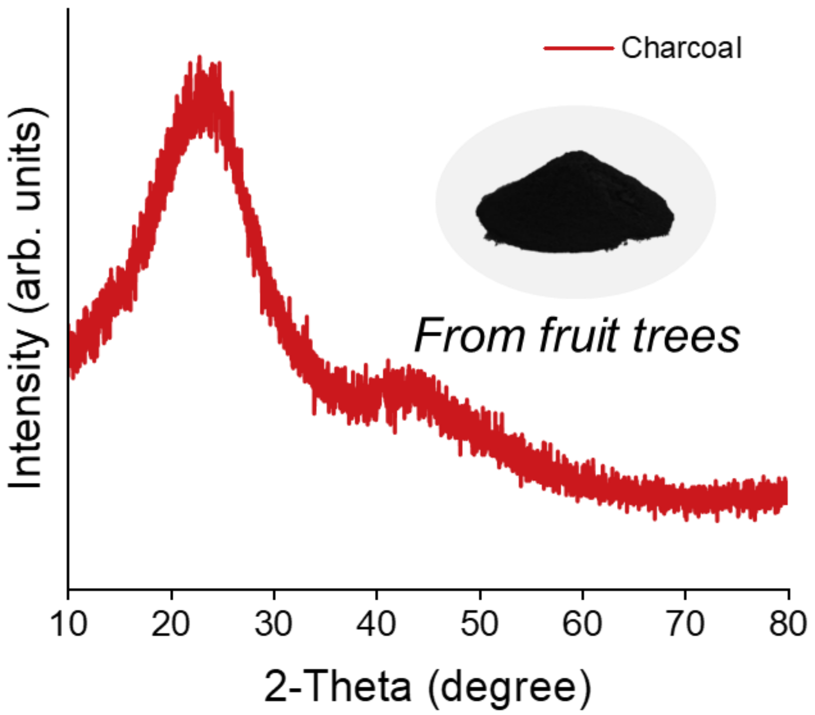

Supplementary Fig. 43 | XRD pattern of charcoal derived from the carbonization of cherry wood. The diffractogram exhibits characteristic broad diffraction peaks (humps), confirming the amorphous structure of the carbon material used in the reduction process.

Supplementary References

1. Gao, W., Fang, Q., Yan, H., Wei, X. & Wu, K. Recovery of Acid and Base from Sodium Sulfate Containing Lithium Carbonate Using Bipolar Membrane Electrodialysis. *Membranes* **11**, 152 (2021).
2. Lee, D., Ho Yun, T., Gi Min, J., Byun, Y. & Yim, C. Regeneration of sodium bicarbonate from industrial Na-based desulfurization waste using ammonium hydroxide. *J. Ind. Eng. Chem.* **122**, 500-510 (2023).
3. Zhang, T. et al. Breaking the Performance Limit of Pure Metals for N_2 Electroreduction. *Angew. Chem., Int. Ed.* **64**, e202514028 (2025).
4. Geng, Z. et al. Achieving a Record-High Yield Rate of 120.9 $\mu g\text{-}NH_3 \cdot mg_{cat}^{-1} \cdot h^{-1}$ for N_2 Electrochemical Reduction over Ru Single-Atom Catalysts. *Adv. Mater.* **30**, 1803498 (2018).
5. Das, A., Barman, S. & Dey, A. Electrocatalytic Reduction of Nitrogen to Ammonia Using a Trinuclear Nickel Thiolate Complex. *J. Am. Chem. Soc.* **147**, 32341-32346 (2025).
6. Cai, X. et al. Membrane electrode assembly design for lithium-mediated electrochemical nitrogen reduction. *Energy Environ. Sci.* **16**, 3063-3073 (2023).
7. Xi, B., Liu, J., Yang, B. & Guan, X. Modulating the alkalinity of molten chloride salt with proton sources for ammonia synthesis. *Catal. Sci. Technol.* **15**, 4406-4418 (2025).
8. Lazouski, N., Schiffer, Z.J., Williams, K. & Manthiram, K. Understanding Continuous Lithium-Mediated Electrochemical Nitrogen Reduction. *Joule* **3**, 1127-1139 (2019).
9. Fu, Y. et al. Copper–palladium hydride interfaces promote electrochemical ammonia synthesis. *Nat. Synth.* (2025). https://doi.org:10.1038/s44160-025-00941-1
10. Huang, Y. et al. Pulsed electroreduction of low-concentration nitrate to ammonia. *Nat. Commun.* **14**, 7368 (2023).
11. Chen, F.-Y. et al. Electrochemical nitrate reduction to ammonia with cation shuttling in a solid electrolyte reactor. *Nat. Catal.* **7**, 1032-1043 (2024).
12. Dang, Y. et al. Balancing active hydrogen and NO_2^- for interfacial tandem nitrate electrocatalysis to ammonia. *Chem. Eng. J.* **520**, 166370 (2025).

13. Liu, W. et al. Efficient ammonia synthesis from the air using tandem non-thermal plasma and electrocatalysis at ambient conditions. *Nat. Commun.* **15**, 3524 (2024).

14. Li, P. et al. Pulsed Nitrate-to-Ammonia Electroreduction Facilitated by Tandem Catalysis of Nitrite Intermediates. *J. Am. Chem. Soc.* **145**, 6471-6479 (2023).

15. Hu, B. et al. Electronic Modulation of the Interaction between Fe Single Atoms and $WO_{2.72-x}$ for Photocatalytic N_2 Reduction. *ACS Catal.* **12**, 11860-11869 (2022).

16. Yu, X. et al. Doping induced diatomic active sites over MoO_{3-x} for efficient and stable photocatalytic N_2 reduction to ammonia. *Chem. Eng. J.* **504**, 158816 (2025).

17. Ren, X. et al. New Lewis acidic ionic liquid coupled carbon shell coated defective WO_3 for efficient photocatalytic N_2 reduction. *Appl. Catal. B: Environ. Energy* **361**, 124579 (2025).

18. Zhang, J. et al. Local microenvironment regulation of $MoS_{2-x}/ZnIn_2S_{4-x}$ heterojunction for enhancing photocatalytic NO_3^- reduction. *Chem. Eng. J.* **496**, 153713 (2024).

19. Hiramatsu, W. et al. Surface Oxygen Vacancies on Copper-Doped Titanium Dioxide for Photocatalytic Nitrate-to-Ammonia Reduction. *J. Am. Chem. Soc.* **147**, 1968-1979 (2025).

20. Guan, X. Without that crystalline touch. *Nat. Catal.* **7**, 961-962 (2024).

21. Zeng, Z., Wang, C. & Fu, L. Liquid metal catalyst for ammonia synthesis at low pressure. *Sci. China Mater.* **68**, 681-682 (2025).

22. European Commission. Large Volume Inorganic Chemicals–Solids and Others Industry. (European Commission, Brussels, 2007).

23. Piccinno, F., Hischier, R., Seeger, S. & Som, C. From laboratory to industrial scale: a scale-up framework for chemical processes in life cycle assessment studies. *J. Cleaner Prod.* **135**, 1085-1097 (2016).

24. Li, B., Asselin, E. & Li, Z. New Process for Na_2CO_3 Production from Na_2SO_4 Based on Modeling the Na_2SO_4–$(NH_4)_2SO_4$–MEA–MEG–H_2O System. *ACS Omega* **9**, 1265-1277 (2024).

25. Abeynaike, A. & Barbenel, Y. Energy carrier exports from New Zealand to Japan – A

comparative life cycle assessment of hydrogen and ammonia. *Int. J. Hydrogen Energy* **107**, 270-278 (2025).

26. Wu, Y. et al. Soda Ash Production with Low Energy Consumption Using Proton Cycled Membrane Electrolysis. *Ind. Eng. Chem. Res.* **58**, 3450-3458 (2019).

27. Zhang, Y., Bakshi, B.R. & Demessie, E.S. Life Cycle Assessment of an Ionic Liquid versus Molecular Solvents and Their Applications. *Environ. Sci. Technol.* **42**, 1724-1730 (2008).

28. Zhu, M. et al. High-value conversion of waste Na_2SO_4 by a bipolar membrane electrodialysis metathesis system. *Resour., Conserv. Recycl.* **186**, 106556 (2022).

29. Wood Mackenzie. China claims 70-90% cost advantage in power plant carbon capture as European projects face costs approaching US$300 per tonne. https://www.woodmac.com/press-releases/china-claims-70-90-cost-advantage-in-power-plant-carbon-capture-as-european-projects-face-costs-approaching-us$300-per-tonne/ (2025) (accessed 15 February 2026).

30. Donkers, P.A.J., Linnow, K., Pel, L., Steiger, M. & Adan, O.C.G. $Na_2SO_4\cdot 10H_2O$ dehydration in view of thermal storage. *Chem. Eng. Sci.* **134**, 360-366 (2015).

31. Hackett, B. The Essentials of Continuous Evaporation. https://publications.aiche.org/cep/2018/may/essentials-continuous-evaporation. (AIChE, 2018) (accessed 15 February 2026).

32. Finkbeiner, M., Inaba, A., Tan, R., Christiansen, K. & Klüppel, H.-J. The New International Standards for Life Cycle Assessment: ISO 14040 and ISO 14044. *Int. J. Life Cycle Assess.* **11**, 80-85 (2006).

33. Wernet, G. et al. The ecoinvent database version 3 (part I): overview and methodology. *Int. J. Life Cycle Assess.* **21**, 1218-1230 (2016).

34. Ferreira, A.G.M. & Lobo, L.Q. The low-pressure phase diagram of sulfur. *J. Chem. Thermodyn.* **43**, 95-104 (2011).
