# Supplementary material for: Upcycling of waste sodium sulfate to sodium carbonate and sulfur
Source: Nat Commun. 2026 Apr 24;17:5503. doi: 10.1038/s41467-026-72286-y (PMC13287641; doi:10.1038/s41467-026-72286-y)
Supplement: Supplementary file 2 — Description of Additional Supplementary File [file 41467_2026_72286_MOESM2_ESM.pdf]

Description of Additional Supplementary Files

Supplementary Data 1: Summary of uncertainty analysis for this work and modified SSA-Process.
