# Supplementary material for: Upcycling of waste sodium sulfate to sodium carbonate and sulfur
Source: Nat Commun. 2026 Apr 24;17:5503. doi: 10.1038/s41467-026-72286-y (PMC13287641; doi:10.1038/s41467-026-72286-y)
Supplement: Supplementary file 5 — Transparent Peer Review File [file 41467_2026_72286_MOESM5_ESM.pdf]

Peer Review File

Upcycling of waste sodium sulfate to sodium carbonate and sulfur

Corresponding Author: Professor Huayi Yin

This file contains all reviewer reports in order by version, followed by all author rebuttals in order by version.

Version 0:

Reviewer comments:

Reviewer #1

(Remarks to the Author)
Manuscript#: NCOMMS-25-65907
Corresponding Author: Huayi Yin
Title: Upcycling of Waste Sodium Sulfate to Sodium Carbonate and SulfurManuscript

General comments:
This study presents a two-step thermochemical process for the conversion of sodium sulfate to sodium carbonate and elemental sulfur. The experimental method is rigorous, the data are of high quality, and the characterization is reasonable . However, the overall significance and novelty of the work are substantially hampered by a problematic framing of the research and an incomplete comparison with state-of-the-art industrial practices.

1. Validity
The data presented are technically sound and support the authors' claims regarding the chemical pathway (Na_2SO_4 → Na_2S → Na_2CO_3 + S_x). The experimental design is appropriate, and the conclusions drawn from the XRD, SEM, TG-MS, and gas analysis are valid. The life-cycle assessment methodology appears standard.

2. Significance
The potential significance of the work is currently overstated. The authors position their process as a novel solution to a major waste problem. However, this positioning is based on two key mischaracterizations:

2.1 Na_2SO_4 is not primarily a "waste" but a valuable commodity chemical intentionally produced for detergents, glass, and the pulp industry. The actual challenge lies in valorizing dilute or contaminated streams of sulfate, which is not the focus here.

2.2 The portrayal of $(NH_4)_2SO_4$ from the SSA-Process as a "secondary ammonia waste" is inaccurate. It is a high-value fertilizer. The true environmental burden of the SSA-Process stems from its reliance on the Haber-Bosch process for ammonia synthesis.

2.3 Furthermore, the authors completely overlook recent critical advances in separation technology of Na_2SO_4 and $(NH_4)_2SO_4$ for the SSA-Process (e.g., MEA anti-solvent crystallization, e.g., Li et al., ACS Omega 9, 1265–1277, 2024). The emergence of photovoltaic-driven electrochemical ammonia synthesis, which operates under mild conditions and is already being piloted in China, promises to decarbonize the SSA-Process's largest energy sink, against which the authors' high-temperature process must be compared.

2.4 The transformation from Na_2SO_4 to Na_2S via carbothermal reduction is well-known (e.g., historically used in the Leblanc process). The subsequent reaction of Na_2S with CO_2 to form Na_2CO_3 + S_x, while interesting, does not represent a conceptual breakthrough. The authors should emphasize what is truly new—e.g., the integration of the two steps, the avoidance of SO_x emissions, or the application to impure streams—rather than implying fundamental discovery of the core chemistry.

2.5 High-Temperature Process Lacks Energy Innovation. The proposed process requires temperatures ≥750°C, which is

highly energy-intensive. The authors do not address: The source of low-carbon process heat required for decarbonization. How the energy efficiency compares to electrochemical route for Na_2SO_4 valorization. The authors must provide a realistic energy-balance analysis and discuss the scalability of their process in a renewables-powered industrial context.

3. Data and Methodology**
The experimental data are of high quality and well-presented. The supplementary information is comprehensive. The LCA is methodologically standard but is applied to an outdated benchmark (a conventional SSA-Process), limiting the usefulness of its conclusions.
The XRD and SEM data are supportive but do not sufficiently prove the absence of intermediates or side products. In situ XRD or XAS during the Na_2S + CO_2 step would strengthen the mechanism.

4. Analytical Approach
The analytical approach is appropriate and comprehensive for validating the chemical process. The LCA approach is standard but would benefit from a scenario analysis that includes a modern benchmark (green NH_3 + advanced separation). A comparison against a future, decarbonized SSA-Process utilizing green PV-NH_3 is absent. The authors must justify the significant energy demand of their own high-temperature process (750-800 °C) against this competing decarbonization pathway. A revised life-cycle assessment should include a scenario where the SSA-Process uses green ammonia to provide a fair and contemporary comparison.

5. Suggested Improvements
To elevate the manuscript to a level suitable for a high-impact journal, major revisions in framing and analysis are required:
5.1 Recast the introduction and discussion to focus on valorizing low-value or impure sulfate streams (e.g., from flue-gas desulfurization) rather than treating Na_2SO_4 as a generic "waste."
5.2 Discuss existing solutions for the SSA-Process's challenges, notably MEA antisolvent crystallization for efficient salt separation.
Perform a Fair Comparative LCA: The most critical revision. The LCA must include a comparison against a modernized SSA-Process benchmark that utilizes: (a) green ammonia from PV-electrochemical synthesis, and (b) advanced MEA separation technology. This is essential to robustly claim any environmental advantage.
Justify Energy Demand: Discuss the high energy requirement (≥750°C) of the proposed process and how it would be powered sustainably in a future industrial context to achieve a true carbon advantage.

6. Clarity and Context
The manuscript is clearly written and well-structured. However, the context provided is incomplete and outdated, as detailed above. The literature review should be expanded to include modern developments in sulfate valorization and green ammonia production.

7. References
References are adequate for the experimental work but are lacking in the sections discussing industrial context and competing technologies. Key literature on advanced separation techniques and electrochemical NH_3 synthesis should be incorporated.

8. Your Expertise
My expertise lies in industrial chemistry and process engineering. I am confident in assessing the experimental work and LCA. My comments on the agricultural market for ammonium sulfate are based on general scientific knowledge.

Reviewer #2

(Remarks to the Author)
The manuscript presents an innovative thermochemical process for upcycling waste Na2SO4 into Na2CO3 and elemental sulfur through carbothermal reduction and CO2 carbonation. The proposed process demonstrates environmental advantages compared to the conventional SSA process. However, several aspects are insufficiently described, particularly within the LCA section, which requires substantial revision. The analysis also needs more detail for reproducibility. The following very critical issues should be addressed:

[Detailed Comments]

1. The applied Na2SO4:C molar ratio of 1:4 is consistent with the overall stoichiometry shown in the main text. However, no evidence is provided regarding unreacted carbon or overall carbon balance. This information is essential to validate the completeness of the reduction and the reliability of the proposed stoichiometry.

2. The Introduction and Methods sections indicate that both life cycle assessment (LCA) and life cycle cost (LCC) analyses were performed. However, no LCC results are provided anywhere in the manuscript or supplementary information. Why were the LCC results omitted, and what methodology was used to analyze them? This omission represents a major gap that raises serious concerns about the completeness of the study. The LCC results, or at minimum a clear methodological description and cost breakdown, must be included to substantiate the authors’ claim of conducting both LCA and LCC analyses.

3. Please complement the LCA breakdown by process step so readers can distinguish the contributions of dehydration, reduction, carbonation, and purification steps. Without this, identifying the dominant contributors remains difficult.

4. The LCA mainly reports total impact scores (mPt) and cumulative energy demand (CED), but the contribution of each product (Na2CO3 and Sx) to the impact categories is not explained. It should be clarified whether the observed environmental benefits originate primarily from Na2CO3 production or from the allocation to Sx as a byproduct.

5. Table S13 indicates that Monte Carlo simulations were performed, but the specific procedure is under described. Readers cannot reproduce the analysis without sufficient detail on how the simulations were conducted. Moreover, some impact categories exhibit extremely high coefficients of variation (water consumption: 4746%; human non-carcinogenic toxicity: 738%). Such high uncertainty could affect the reliability of the results and requires thorough discussion.

6. The study applies economic allocation to distribute environmental burdens among co-products, but no sensitivity analysis is provided. Given the substantial volatility of product prices in recent years, the allocation results presented here cannot be considered reliable. The authors should re-evaluate the LCA outcomes by incorporating the recent variations in product price or, at minimum, perform a quantitative sensitivity analysis to verify the robustness of their conclusions.

7. To enhance the reproducibility of the thermodynamic analysis, it would be helpful to include a more detailed description of the input parameters and model settings used in the FactSage and HSC calculations. While general assumptions (e.g., activities = 1, standard pressure) and reaction equations are provided, clarifying other relevant settings or input data would improve transparency.

8. Minor issues include typographical and formatting errors: in line 88, “tetrahedral structure of SO4 2- is highly sTab.” should read “stable”; in line 135, “like H2 may serve ass viable options” should read “as”; and in Supplementary Table S4, the column labeled “Total 1 (frac)” should be corrected to “100 (%)” for consistency.

Reviewer #3

(Remarks to the Author)
In this study, the authors propose a novel process for producing Na2CO3 from Na2SO4. Considering the growing issue of large-scale Na2SO4 discharge, the topic and approach are timely and of potential interest. However, the main weakness of this work lies in the insufficient discussion of the engineering aspects of the proposed process. Although the method may offer certain mechanical or operational advantages compared with conventional processes, its practical feasibility remains unclear. Overall, the manuscript would benefit from a more comprehensive discussion of the engineering considerations, including scale-up strategies, process limitations, and future research directions.

Specific Comments
1. To validate the feasibility of the proposed Na2CO3 production process, a techno-economic analysis should be performed. For example, although the manuscript mentions some drawbacks of bipolar membrane electrodialysis (BMED), BMED could still exhibit better economic viability when considering the cost of NaOH. Therefore, the authors should include a comparative TEA to quantitatively assess the economic feasibility of the proposed process relative to conventional methods.
2. What is the production yield of Na2CO3 from Na2SO4 in the proposed process? Additionally, while the authors claim that SO_x emissions are inhibited, incomplete reactions could potentially lead to the formation of sulfur oxides. Please provide further discussion on the productivity, reaction completeness, and possible side reactions.
3. In Figure 4, it is unclear how the authors controlled or mitigated the formation of volatile S_x species and residual carbon on the Na2CO3 product. Please clarify the control strategy and its influence on product quality.
4. Although the manuscript focuses primarily on mechanistic studies, the engineering aspects of the process are not sufficiently addressed. The authors should expand the discussion to include process limitations, purity control, and potential scale-up strategies, as well as future research directions necessary to advance this process toward industrial application.

Version 1:

Reviewer comments:

Reviewer #1

(Remarks to the Author)
Re: Review of revised Nature Communications manuscript NCOMMS-25-65907A entitled “Upcycling of waste sodium sulfate to sodium carbonate and sulfur”.

I have carefully examined the authors’responses to the reviewers’comments, including my own, as well as the corresponding revisions made to the manuscript.

I am pleased to confirm that the authors have addressed all the concerns I raised in my previous report comprehensively and thoughtfully. Their revisions include:

Detailed clarification of the reaction mechanisms and pathways, which significantly strengthens the scientific rigor of the work.

A more thorough and quantitative comparison between the proposed process (this study) and existing industrial(SSA-process) or reported methods (New SSA-Process). This comparative analysis has been substantially enhanced with relevant data and discussion, providing a clear and convincing context for the novelty and advantages of their approach.

Strengthened evidence and discussion supporting the claimed high credibility of the results. The authors have added necessary experimental details, data validation, and more nuanced interpretation where appropriate.

The revised manuscript now presents a more complete, robust, and well-justified study. The technical improvements are clear, and the narrative has been polished to better highlight the significance and potential impact of this waste upcycling strategy. The work demonstrates a sound methodological approach, and the conclusions are well-supported by the presented data.

Overall, the revision process has markedly improved the quality of the manuscript. The current version is coherent, technically solid, and presents a significant advancement in the field of chemical upcycling and resource recovery. The level of scientific exposition and experimental substantiation now meets the high standards expected for publication in a leading journal.

Reviewer #2

(Remarks to the Author)
The manuscript presents an innovative thermochemical process for upcycling waste Na2SO4 into Na2CO3 and elemental sulfur via carbothermal reduction followed by CO_2 carbonation. The authors have made substantial efforts to address the concerns raised in the previous review, and the revised manuscript shows improvement in clarity, methodological transparency, and robustness of the analyses.

The major issues identified in the first round have been satisfactorily addressed. However, one remaining issue concerns the economic assessment described as “LCC,” which requires substantial clarification for methodological transparency and reproducibility.

[Detailed Comments]
1. Major comment (LCC / economic analysis): The revised manuscript includes cost-related results with breakdowns and electricity price sensitivity. However, the calculation framework is not sufficiently described in either the main text or the Supplementary Information (e.g., system boundary, included cost components, and cost aggregation procedure/equations), making it difficult to assess and reproduce the analysis. In addition, the assessment appears limited to OPEX, raising concerns about whether the term “LCC” is appropriate without a clear definition of scope. Please either provide a clear and reproducible description of the LCC methodology (scope/system boundary, cost items, and calculation procedure) or revise the terminology to accurately reflect an operating-cost-based techno-economic assessment.

Reviewer #3

(Remarks to the Author)
All concerns have been addressed, and the manuscript is suitable for publication as is.

Version 2:

Reviewer comments:

Reviewer #2

(Remarks to the Author)
The authors have made substantial revisions in response to the concerns raised during the previous review round. The economic assessment framework has been significantly clarified, with a clearly defined system boundary, explicit cost components, and reproducible calculation procedures presented in the Supplementary Information. The scope and definition of the life cycle cost (LCC) assessment are now clearly articulated and consistently implemented, and the sensitivity analysis aligns with the revised methodology.

Overall, the revisions adequately address the prior concerns, and the manuscript is now suitable for publication in Nature Communications.

Open Access This Peer Review File is licensed under a Creative Commons Attribution 4.0 International License, which permits use, sharing, adaptation, distribution and reproduction in any medium or format, as long as you give appropriate credit to the original author(s) and the source, provide a link to the Creative Commons license, and indicate if changes were made.
In cases where reviewers are anonymous, credit should be given to 'Anonymous Referee' and the source.
The images or other third party material in this Peer Review File are included in the article's Creative Commons license, unless indicated otherwise in a credit line to the material. If material is not included in the article's Creative Commons license and your intended use is not permitted by statutory regulation or exceeds the permitted use, you will need to obtain permission directly from the copyright holder.
To view a copy of this license, visit https://creativecommons.org/licenses/by/4.0/

Response to referees

Journal: Nature Communications

Manuscript ID: NCOMMS-25-65907

Title: Upcycling of waste sodium sulfate to sodium carbonate and sulfur

Author(s): Hongya Wang, Shiyu Wang, Fengyin Zhou, Muya Cai, Jingjing Zhao, Danfeng Wang, Zhan Shi, Xiang Chen, Dihua Wang, Huayi Yin*

We are grateful to all the reviewers for their comments and suggestions. We have revised the manuscript, taking into full account the reviewers' comments and recommendations. Below are our point-to-point responses in the same order as the reviewers' comments. Replies are marked in blue, revisions are marked in purple.

Reviewer #1

Comments:

This study presents a two-step thermochemical process for the conversion of sodium sulfate to sodium carbonate and elemental sulfur. The experimental method is rigorous, the data are of high quality, and the characterization is reasonable. However, the overall significance and novelty of the work are substantially hampered by a problematic framing of the research and an incomplete comparison with state-of-the-art industrial practices.

Reply: We sincerely thank you for recognizing the rigor of our experimental method and the high quality of our data, and we deeply appreciate your insightful critique regarding the framing of our research. We fully agree with you that a thorough benchmarking against state-of-the-art industrial practices is essential to substantiate the novelty and practical significance of our work.

In direct response to your suggestion, we have significantly expanded our discussion to include a comprehensive comparison with cutting-edge technologies, specifically analyzing our approach against PV-driven green NH_3 synthesis and advanced MEA-MEG extraction systems for high-purity $(NH_4)_2SO_4$ production. These revisions, which have been incorporated into the **Revised Manuscript** and **Revised Supplementary Information**, clearly delineate the comparative advantages of our technology, and we are grateful for your guidance in prompting these crucial improvements that have firmly strengthened the positioning of our manuscript.

1. Validity

The data presented are technically sound and support the authors' claims regarding the chemical pathway (Na_2SO_4 → Na_2S → Na_2CO_3 + S_x). The experimental design is appropriate, and the conclusions drawn from the XRD, SEM, TG-MS, and gas analysis are valid. The life-cycle assessment methodology appears standard.

Reply: We are deeply grateful for your time and positive assessment. We are truly honored by your recognition of the technical soundness and methodological validity of our work.

2. Significance

The potential significance of the work is currently overstated. The authors position their process as a novel solution to a major waste problem. However, this positioning is based on two key mischaracterizations:

2.1 Na_2SO_4 is not primarily a "waste" but a valuable commodity chemical intentionally produced for detergents, glass, and the pulp industry. The actual challenge lies in valorizing dilute or contaminated streams of sulfate, which is not the focus here.

Reply: We appreciate your insightful comments regarding the market status of Na_2SO_4. We fully agree with your assessment that Na_2SO_4 has traditionally been a valuable commodity in the desiccant, detergent, and glass industries. However, we would like to clarify why we focus on it as a "waste" or surplus issue in our specific context. The term "waste" in this paper does not mean it is not useful. It means that it is a byproduct and cannot be fully digested by industry.

1. **Too much supply, less demand:** While Na_2SO_4 is a commodity, industries like Li battery making and chemical processing are now producing huge amounts of it as a byproduct (over 20 million tons/year) [*Resources, Conservation and Recycling*, **2022**, 186: 106556] (**Fig. R1-1a**). This is much more than the world needs (about 9 – 12 million tons/year). At the same time, detergent makers are switching to liquid soaps, which do not use Na_2SO_4, so the demand is shrinking. This creates a massive surplus that piles up as waste.
2. **Closing the loop inside the factory:** Our goal is to turn this "extra" salt back into something the factory actually needs (**Fig. R1-1b**). For example, a lithium extraction plant produces Na_2SO_4 byproduct but has to buy Na_2CO_3 to operate. Our method allows them to recycle their waste back into Na_2CO_3 right on-site. This saves money, reduces waste accumulation, and also reduces Li

losses [*Ind. Eng. Chem. Res.*, **2024**, 63(11): 4918-4933].

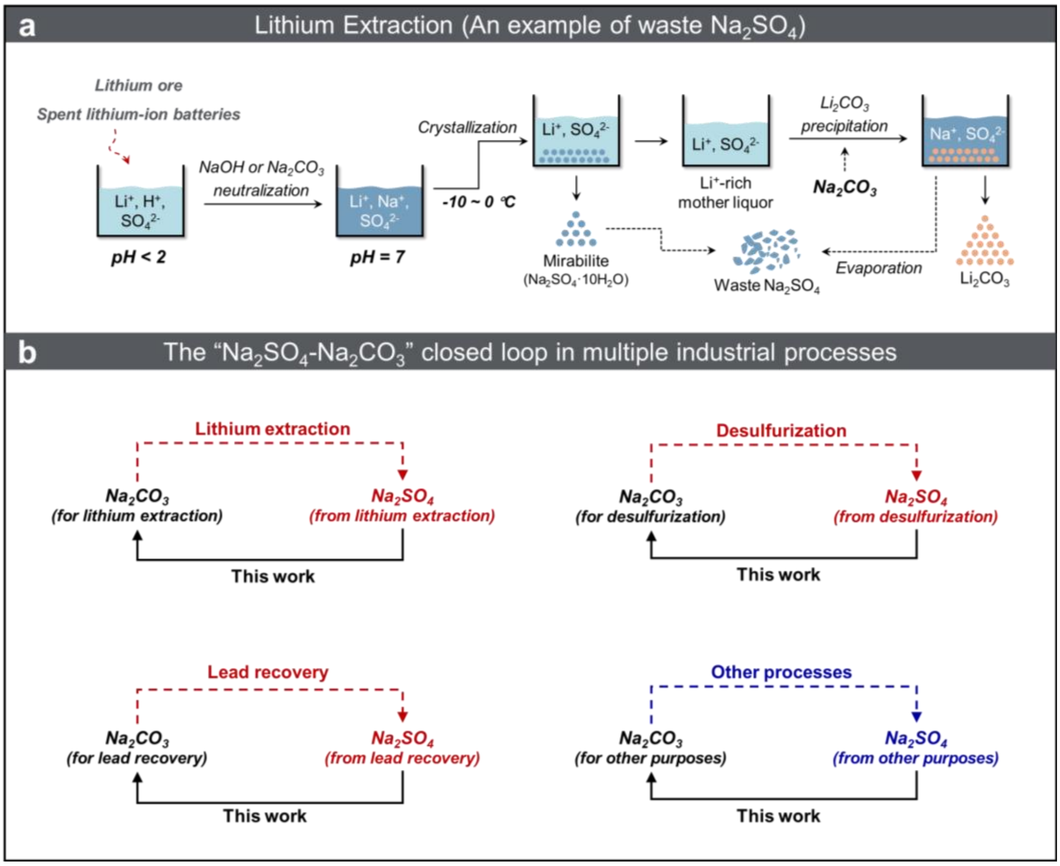

Fig. R1-1 | a, Discharge of waste Na_2SO_4 stream during lithium extraction process. **b**, The "Na_2SO_4- Na_2CO_3" closed loop in multiple industrial processes.

3. Handling dirty or watery waste:

- **The Na_2SO_4 solution dissolved with other salts:** You mentioned contaminated streams. We tested our method with Na_2SO_4 mixed with common salt (NaCl), and it works very well (***Scale-up production, LCA, and economic analysis*** in **Manuscript**). A big advantage is that our process does not need NH_3, making it easier to use than traditional methods (SSA-Process).

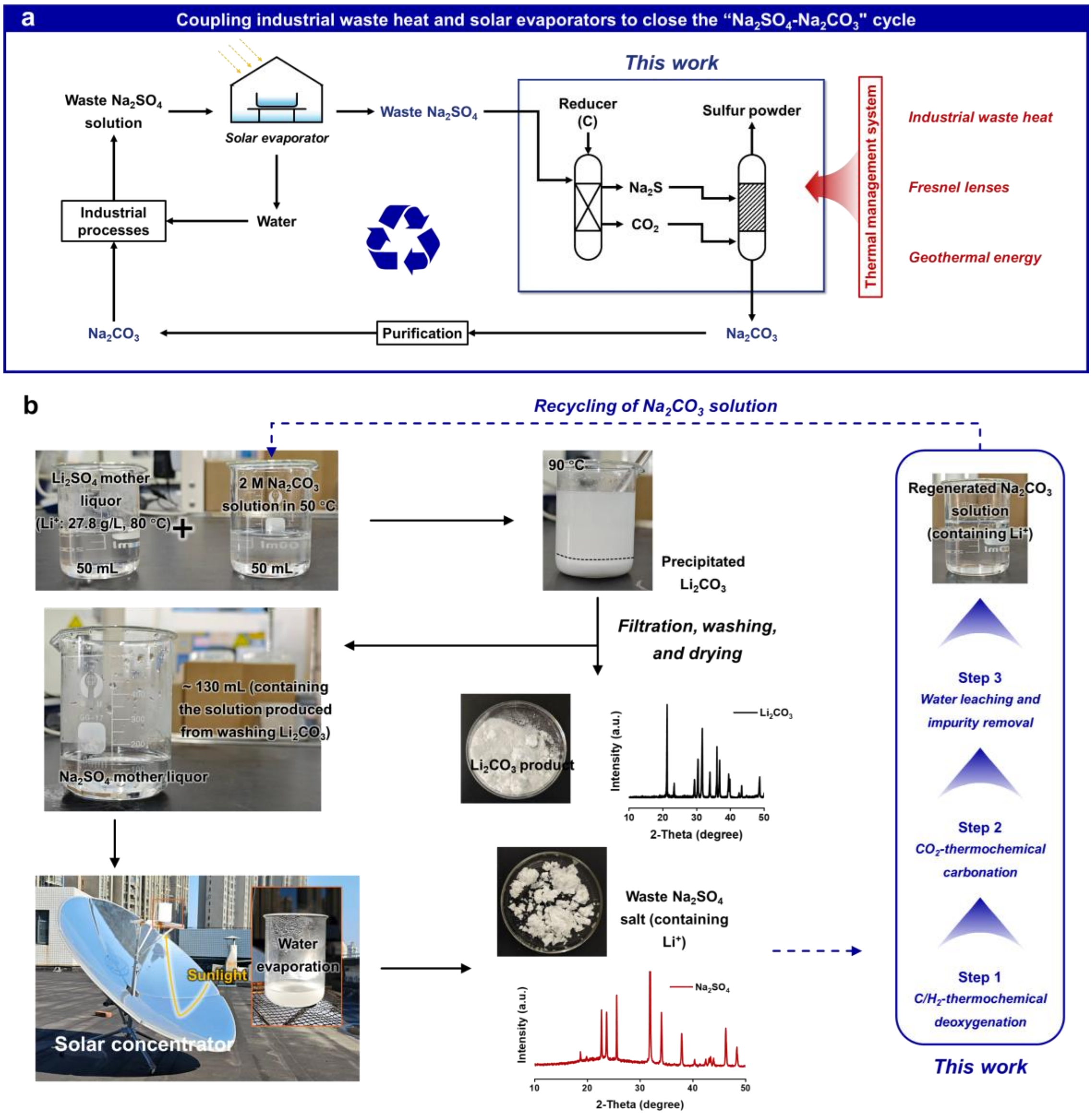

Fig. R1-2 | Strategies for closing the "Na_2SO_4-Na_2CO_3" loop via multi-energy integration. a, Schematic illustration of the synergistic coupling of industrial waste heat and solar evaporators to achieve a sustainable Na_2SO_4-Na_2CO_3 cycle. The system integrates solar thermal energy (e.g., Fresnel lenses) and geothermal energy to optimize thermal management. **b**, Practical demonstration of this cycle applied to lithium extraction. The process involves the precipitation of Li_2CO_3 followed by the concentration of the Na_2SO_4 mother liquor using a solar concentrator. The recovered Na_2SO_4 solid is then processed via the proposed thermochemical route (Steps 1-3) to regenerate the Na_2CO_3 solution for reuse.

- **Dilute Na_2SO_4 solution:** For the solution with a low Na_2SO_4 centration, we suggest using solar evaporation (using the sun to dry it, like making salt from seawater) to remove the water first,

which has been successfully implemented in seawater desalination (**Fig. R1-2**) [*Nat. Water*, **2024**, 2(2): 115-126]. Once it is dried, our method can process it efficiently.

We have updated the manuscript and added new diagrams to explain these points clearly. Thank you again for helping us improve the context of our work.

Revise:

The Revised Introduction

Several strategies have been adopted to valorize waste Na_2SO_4 into industrial feedstocks, aiming to establish a sustainable "valuable product-to-Na_2SO_4-to-valuable product" resource loop. Existing methods primarily focus on converting SO_4^{2-} into H_2SO_4 or reusable sulfates[5, 14-16]. For instance, bipolar membrane electrodialysis (BMED) offers a pathway to regenerate NaOH and H_2SO_4 (or sulfate) solutions[5, 17-19]. However, to fully realize its techno-economic potential for large-scale applications, optimization is still required regarding membrane costs (~50% of capital expenditure)[20, 21], fouling induced by impurities in waste Na_2SO_4[22, 23], and product concentrations (< 1 M)[24, 25]. Alternatively, the SSA-Process produces Na_2CO_3, a critical raw material with world production projected to exceed 80 million tons by 2030 (**Supplementary Figs. 2a-b** and **Table 2**)[26, 27], simultaneously yielding ammonium sulfate [$(NH_4)_2SO_4$][6, 16, 28, 29]. While successfully demonstrated at the pilot scale[30], this process currently relies on ammonia (NH_3) to drive carbonation (**Supplementary Figs. 2c-d**)[29]. Although emerging electrochemical NH_3 synthesis offers a potential sustainable pathway (**Supplementary Fig. 3**), further enhancements in efficiency are anticipated to facilitate its large-scale deployment (**Supplementary Table 3**)[31]. Recent advances in anti-solvent crystallization have notably improved Na_2SO_4 utilization and byproduct purity, underscoring the evolving potential of the SSA-Process[16]. Nevertheless, the development of a complementary NH_3-free paradigm is essential for ensuring comprehensive industrial viability and enabling Na_2CO_3 production in regions lacking NH_3 infrastructure[32, 33]. Consequently, an NH_3-free strategy that reconverts waste Na_2SO_4 into Na_2CO_3 and an easy-to-digest byproduct is urgently needed, offering a transformative solution to close both the sodium and sulfur resource loops.

The Revised Results

Beyond process-level metrics, this work has broader implications for waste management and circular resource use. We estimate that unmanaged stockpiling of global waste Na_2SO_4 would squander ~1,864 acres of land annually (**Supplementary Fig. 39**). To demonstrate circularity, we tested a

closed-loop application in Li_2CO_3 extraction by coupling a solar concentrator for dehydration with carbonate reagent regeneration (**Supplementary Fig. 40**). Furthermore, we developed a general "alkaline sodiation" strategy to extend this protocol to other complex sulfate wastes, effectively preventing hazardous SO_x emissions (**Supplementary Fig. 41**). Looking forward, we envision three application scenarios: (i) decentralized Na_2SO_4 upcycling in regions lacking NH_3 infrastructure, (ii) integrated on-site production-consumption loops within industrial parks, and (iii) a carbon-negative pathway coupling green H_2 deoxygenation with direct air CO_2 capture (**Supplementary Fig. 42**).

The Revised Supplementary Information

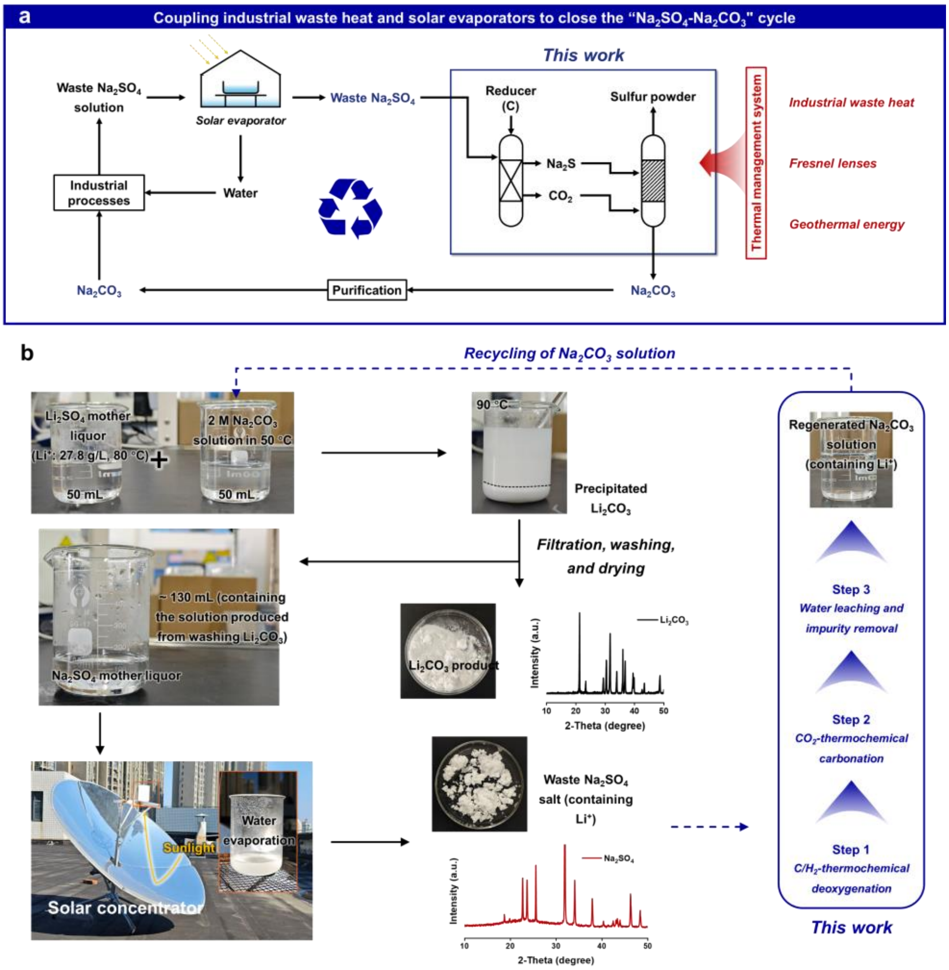

Supplementary Fig. 40 | Strategies for closing the "Na_2SO_4-Na_2CO_3" loop via multi-energy integration. a, Schematic illustration of the synergistic coupling of industrial waste heat and solar evaporators to achieve a sustainable Na_2SO_4-Na_2CO_3 cycle. The system integrates solar thermal energy (e.g., Fresnel lenses) and geothermal energy to optimize thermal management. **b**, Practical demonstration of this cycle applied to lithium extraction. The process involves the precipitation of Li_2CO_3 followed by the concentration of the Na_2SO_4 mother liquor using a solar concentrator. The

recovered Na_2SO_4 solid is then processed via the proposed thermochemical route (Steps 1-3) to regenerate the Na_2CO_3 solution for reuse.

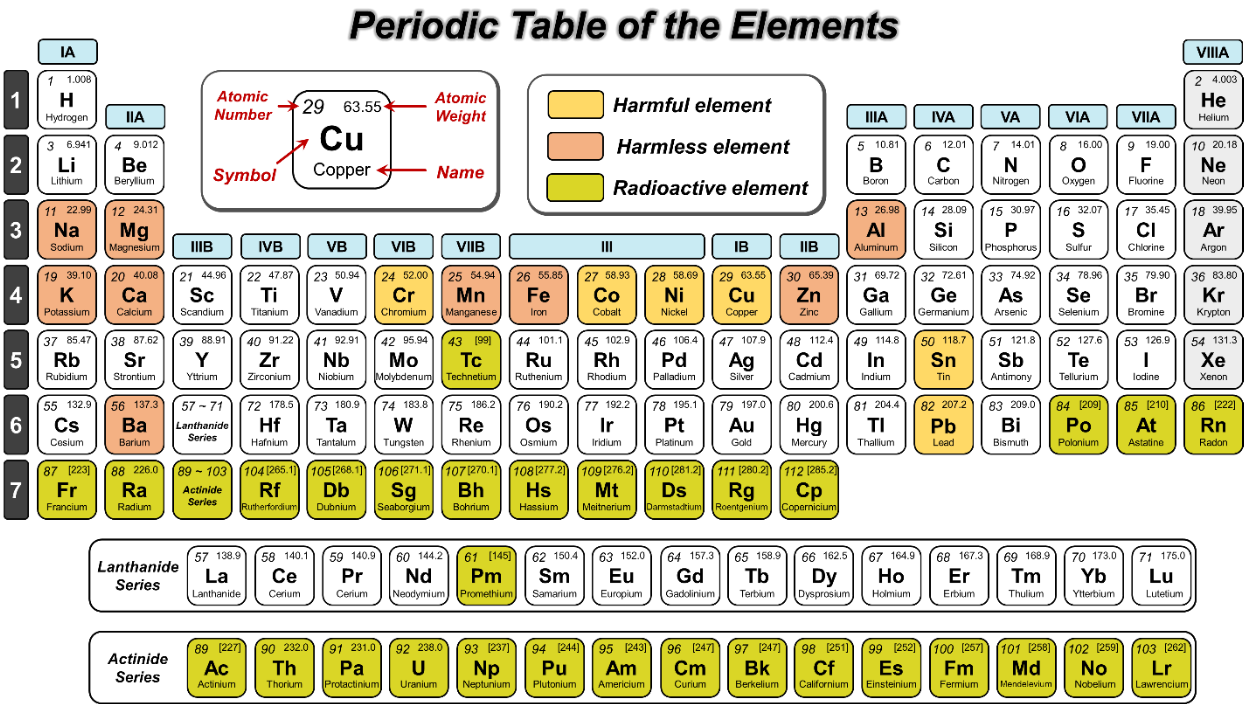

Supplementary Fig. 41 | Overview of typical waste sulfates (e.g., Na_2SO_4, $CaSO_4$, $FeSO_4$) identified as potential renewable resources.

Beyond Na_2SO_4, there are diverse sulfate byproducts in many industrial processes, such as $FeSO_4$ from TiO_2 manufacturing, $CaSO_4$ from phosphate extraction, and $NiSO_4$ from spent LIBs. The thermochemical route proposed herein can serve as a versatile "soda regeneration hub" coupled with a pre-treatment strategy we term "alkaline sodiation." Taking the ubiquitous $FeSO_4$ as an example: direct carbothermal reduction typically releases hazardous SO_2 gas. However, by introducing Na-based alkalis (Na_2CO_3 or NaOH), complex sulfates can be converted into valuable metal oxides (e.g., Fe_xO_y for metallurgical feedstocks) and Na_2SO_4. This intermediate Na_2SO_4 is subsequently processed via our technology to regenerate the soda ash reagents required for the initial conversion and to recover S_x. This synergistic integration allows for the closed-loop valorization of a wide spectrum of sulfate wastes, avoiding SO_2 emissions while recovering critical metals and sulfur resources.

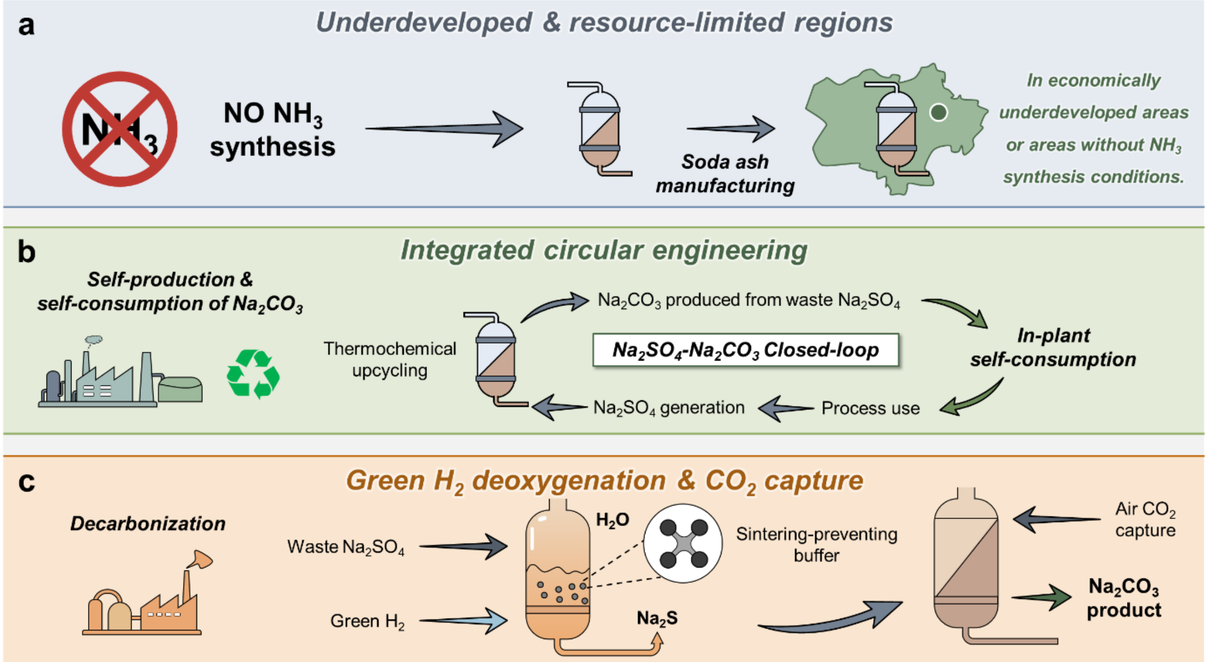

Supplementary Fig. 42 | Future perspectives on industrial deployment and low-carbon evolution of the proposed technology. a, Deployment feasibility for economically underdeveloped regions or areas lacking synthetic NH_3 infrastructure, enabling decentralized, NH_3-free soda ash manufacturing. **b**, Strategy for integrated circular engineering to establish an on-site Na_2SO_4-Na_2CO_3 closed-loop, realizing internal self-sufficiency. **c**, Advanced decarbonization pathway coupling green H_2-based deoxygenation (utilizing sintering-preventing buffers in a fluidized bed) with direct air CO_2 capture.

2.2 The portrayal of $(NH_4)_2SO_4$ from the SSA-Process as a "secondary ammonia waste" is inaccurate. It is a high-value fertilizer. The true environmental burden of the SSA-Process stems from its reliance on the Haber-Bosch process for ammonia synthesis.

Reply: Thank you for your insightful comments. We appreciate the opportunity to clarify the portrayal of $(NH_4)_2SO_4$ in the context of the SSA-Process. We fully agree with your observation that $(NH_4)_2SO_4$ is a high-value fertilizer and not "secondary ammonia waste," and we regret any confusion caused by the initial wording in our **Manuscript**.

We reaffirm our agreement with your view on the value of $(NH_4)_2SO_4$. However, we maintain that eliminating the use of NH_3 entirely—and thus removing the upstream reliance on the Haber-Bosch process—is critical for decoupling Na_2CO_3 production from high CO_2 emissions and NH_3 supply constraints. We have revised the **Revised Manuscript** to correct the "secondary waste" terminology, redirecting the discussion toward the issues of NH_3 dependence, storage instability, and potential contamination risks.

Revise:

The Revised Abstract

Waste sodium sulfate (Na_2SO_4) is a common industrial byproduct that poses environmental risks and resource loss if improperly managed. Here, we report a thermochemical upcycling method to convert waste Na_2SO_4 into value-added sodium carbonate (Na_2CO_3) and sulfur (S_x). In this process, Na_2SO_4 is first reduced to sodium sulfide (Na_2S) at 750 °C using charcoal. Subsequently, the generated Na_2S is oxidized by CO_2 via carbonation at 300 °C to produce Na_2CO_3 and S_x. Temperature modulation shifts thermodynamic equilibrium to drive the conversion of SO_4^{2-} to S_x, achieving a carbonate yield of 95.35% with purity exceeding 99.53%. Life cycle assessment (LCA) indicates that this anhydrous route reduces the global warming potential by > 0.43 kg CO_2-eq per kg Na_2CO_3 compared with the conventional sodium sulfate-based ammonia-soda process (SSA-Process). By eliminating water-intensive steps and ammonia usage, our method lowers the end-point environmental impact to 34.69 mPt per kg Na_2CO_3 (vs. 48.81 mPt for conventional routes). Overall, this work provides a sustainable strategy for reclaiming waste salts and closing the sodium and sulfur cycles.

The Revised Introduction

Alternatively, the SSA-Process produces Na_2CO_3, a critical raw material with world production

projected to exceed 80 million tons by 2030 (**Supplementary Figs. 2a-b** and **Table 2**)[26, 27], simultaneously yielding ammonium sulfate [$(NH_4)_2SO_4$][6, 16, 28, 29]. While successfully demonstrated at the pilot scale[30], this process currently relies on ammonia (NH_3) to drive carbonation (**Supplementary Figs. 2c-d**)[29]. Although emerging electrochemical NH_3 synthesis offers a potential sustainable pathway (**Supplementary Fig. 3**), further enhancements in efficiency are anticipated to facilitate its large-scale deployment (**Supplementary Table 3**)[31]. Recent advances in anti-solvent crystallization have notably improved Na_2SO_4 utilization and byproduct purity, underscoring the evolving potential of the SSA-Process[16]. Nevertheless, the development of a complementary NH_3-free paradigm is essential for ensuring comprehensive industrial viability and enabling Na_2CO_3 production in regions lacking NH_3 infrastructure[32, 33]. Consequently, an NH_3-free strategy that reconverts waste Na_2SO_4 into Na_2CO_3 and an easy-to-digest byproduct is urgently needed, offering a transformative solution to close both the sodium and sulfur resource loops.

The Revised Results

As previously discussed, current methods for disposing and recycling waste Na_2SO_4 involve the direct production of other low-usage products (**Equation R1**), or the utilization of bipolar membranes or NH_3 to produce acids, bases, and Na_2CO_3 (**Figs. 1a-c**). However, these approaches often result in substantial environmental burdens or rely heavily on energy-intensive NH_3 production. BMED generates NaOH and H_2SO_4 by dissociating water and coupling Na^+ and SO_4^{2-} (**Equation R2**). While the SSA-Process produces the more desirable Na_2CO_3, implementation is constrained by its dependence on NH_3 (**Equation R3**). In contrast, the proposed two-step thermochemical upcycling process converts Na_2SO_4 into Na_2CO_3 and S_x under NH_3-free conditions (**Fig. 1d**). This method first involves the deoxygenation of Na_2SO_4 followed by CO_2 carbonation, thereby achieving NH_3-free Na_2CO_3 production and providing a cleaner and more controllable route for sulfur transformation.

The Revised Supplementary Information

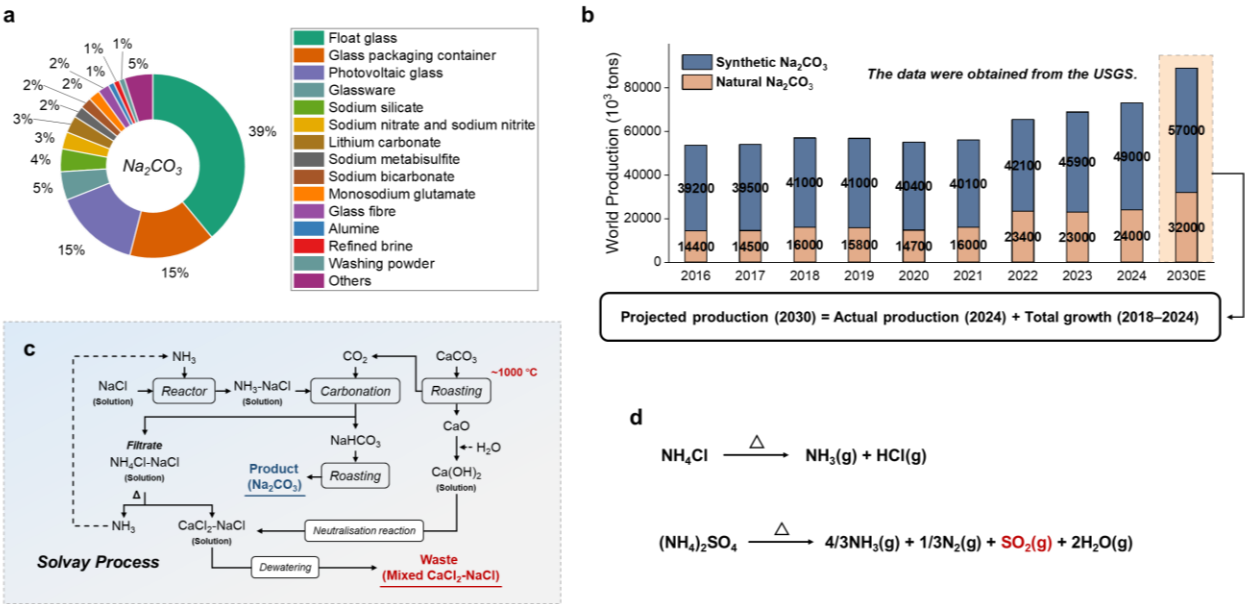

Supplementary Fig. 2 | Market dynamics and conventional production pathways of Na_2CO_3. a, Downstream consumption structure of Na_2CO_3 in China (2023). **b**, World production trends for Na_2CO_3 from 2016 to 2030. Data are sourced from the U.S. Geological Survey (USGS). **c**, Schematic flowchart of the conventional Solvay process for Na_2CO_3 production. **d**, Reaction schemes comparing the thermal decomposition of NH_4Cl and $(NH_4)_2SO_4$.

Supplementary Fig. 2a illustrates that the float glass sector dominated consumption in China in 2023, accounting for 39% of the market, followed by glass packaging containers and photovoltaic glass. Propelled by the global transition toward renewable photovoltaic energy and the rapid expansion of the electric vehicle market, the demand for exhibits a sustained upward trajectory (**Supplementary Fig. 2b**). Currently, industrial production relies predominantly on the Solvay process, a method constrained by high energy intensity (2.8 GJ per ton of Na_2CO_3) and substantial waste generation (**Supplementary Fig. 2c**)[26]. Consequently, the SSA-Process encounters thermodynamic and environmental challenges analogous to those inherent in this conventional route.

2.3 Furthermore, the authors completely overlook recent critical advances in separation technology of Na_2SO_4 and $(NH_4)_2SO_4$ for the SSA-Process (e.g., MEA anti-solvent crystallization, e.g., Li et al., ACS Omega 9, 1265–1277, 2024). The emergence of photovoltaic-driven electrochemical ammonia synthesis, which operates under mild conditions and is already being piloted in China, promises to decarbonize the SSA-Process's largest energy sink, against which the authors' high-temperature process must be compared.

Reply: We sincerely thank you for bringing these recent research advances to our attention. We fully agree that this field is evolving rapidly, and comparing our work with these cutting-edge technologies is crucial for properly contextualizing our contribution.

1. Regarding MEA-MEG anti-solvent crystallization: We have carefully studied the work by Li et al. on the MEA-MEG-H_2O system for the efficient separation of Na_2SO_4 and $(NH_4)_2SO_4$ (*ACS Omega*, **2024**, 9(1): 1265-1277). We acknowledge this as a significant advancement for the traditional SSA-Process, and we have updated our **Manuscript** to highlight its contribution to separation technology.

2. Regarding PV-driven electrochemical NH_3 synthesis: We fully recognize the potential of PV-driven electrochemical synthesis to decarbonize the traditional Haber-Bosch route. Accordingly, we have incorporated the latest technical parameters and production efficiencies of green NH_3 into our **Revised Supplementary Information** to assess current progress (**Fig. R1-3**). We note that in the near term, the Haber-Bosch process likely remains the dominant industrial route. We emphasize that our NH_3-free process for converting waste Na_2SO_4 is intended not as a competitor to the SSA-Process, but as a complement. Our goal is to achieve waste disposal and clean S transfer via an NH_3-free pathway. In addition, our technology is specifically designed for factories and regions that lack NH_3 synthesis infrastructure or supply. Finally, our process is not inherently dependent on fossil fuels; it can be powered by electrification or concentrated solar thermal energy, aligning with the same global decarbonization goals as PV-driven NH_3 synthesis.

To address your comments, we have summarized the advances in MEA-MEG solvent extraction and PV-driven green NH_3 synthesis in the **Revised Introduction** to provide a balanced perspective. Furthermore, we propose that our technology will serve as an effective parallel solution for NH_3-free Na_2CO_3 production, particularly in regions unsuitable for the conventional SSA-Process (***outlook***).

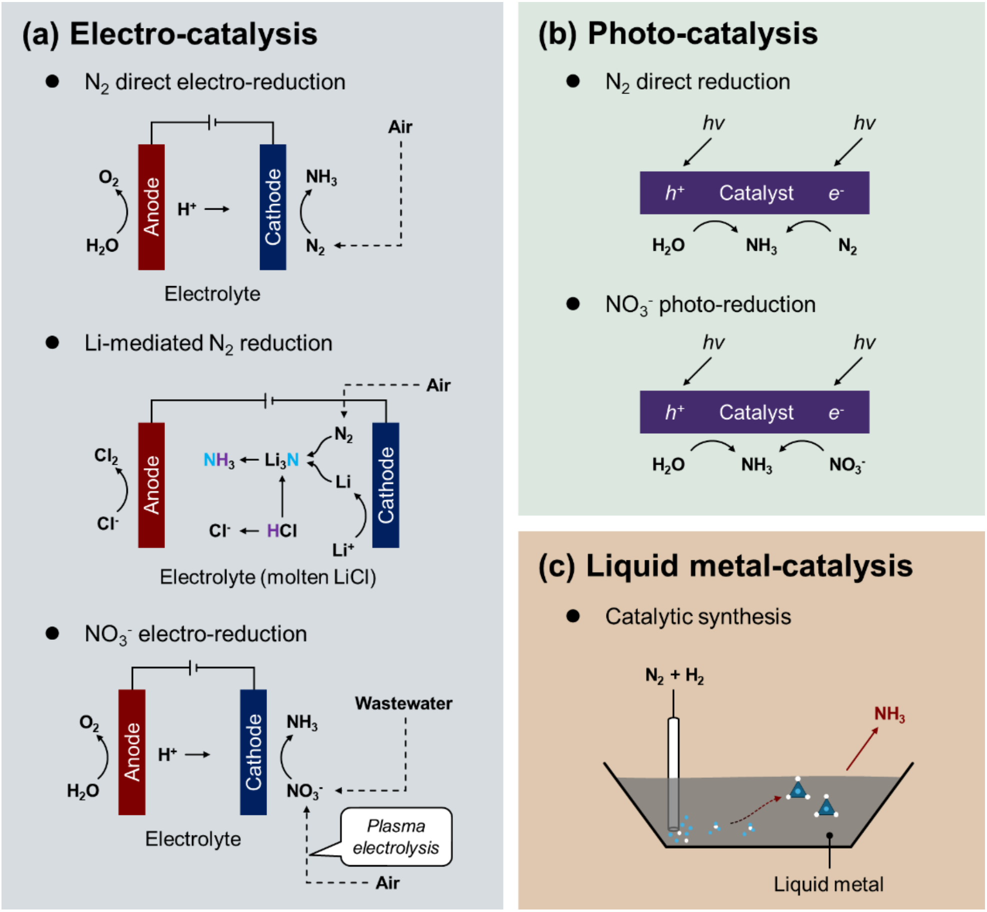

Fig. R1-3. Schematic representation of emerging alternative NH_3 synthesis pathways. a, Electro-catalytic strategies, encompassing direct nitrogen reduction (N_2 direct electro-reduction), lithium-mediated nitrogen reduction (Li-mediated N_2 reduction), and nitrate electro-reduction (NO_3^- electro-reduction). **b**, Photo-catalytic approaches utilizing light energy (hν) to drive direct N_2 reduction and NO_3^- photo-reduction over semiconductor catalysts. **c**, Liquid metal-catalyzed synthesis, where liquid metals facilitate the reaction between gaseous N_2 and H_2.

Revise:

The Revised Introduction

Although emerging electrochemical NH_3 synthesis offers a potential sustainable pathway (**Supplementary Fig. 3**), further enhancements in efficiency are anticipated to facilitate its large-scale deployment (**Supplementary Table 3**)[31].

The Revised Supplementary Information

Supplementary Table 3 | Summary of state-of-the-art developments in green NH_3 synthesis.

		Catalyst	Electrolyte	Production rate	Current efficiency	Ref.
**Electro-**	N_2 direct	Fe HF	0.1 M Na_2SO_4	27.1 μg·h^{-1}·cm^{-2}	3.5%	Zhang et

**catalysis**	electro-reduction					al. (2025)[24]
		Ru SAs/N-C	0.05 M H_2SO_4	120.9 $\mu g \cdot h^{-1} \cdot mg_{cat}^{-1}$	29.6%	Geng et al. (2018)[25]
		Trinuclear nickel thiolate complex	PhOH-0.1 M $^{n}Bu_4NClO_4$	/	83%	Das et al. (2025)[26]
	Li-mediated N_2 reduction	Lithium-deposited stainless-steel cloth	Lithium-doped polyethylene oxide	< 2.55 $mmol \cdot h^{-1} \cdot cm_{geo}^{-2}$	< 10.6%	Cai et al. (2023)[27]
		Molten salt-based NH_3 synthesis system (Ni rod cathode)	Molten LiCl-KCl	3.24 $\mu mol \cdot h^{-1} \cdot g_{molten\ salt}^{-1}$	/	Xi et al. (2025)[28]
		Cu-Li/Li_3N	$LiBF_4$ THF EfOH	< 34.2 $\mu mol \cdot h^{-1} \cdot cm^{-2}$	< 20.4%	Lazouski et al. (2019)[29]
	NO_3^- electro-reduction	Cu-Pd	1 M KNO_3 + 1M KOH	19.9 $mmol \cdot h^{-1} \cdot cm^{-2}$	86.8%	Fu et al. (2025)[30]
		$RuIn_3$/C	0.1 M KOH (10 mM NO_3^-) + 0.1 M KOH	2.7 $mmol \cdot h^{-1} \cdot mg_{Ru}^{-1}$	97.6%	Huang et al. (2023)[31]
		Ru-CuNW	0.258 M NaOH (2,000 ppm NO_3^-) + Nafion-117 + 0.258 M NaOH	/	> 90%	Chen et al. (2024)[32]
		Ni@Cu_{2-x}Se	1 M KOH (0.1 M NO_3^-) + 1 M KOH	17.2 $mg \cdot h^{-1} \cdot cm^{-2}$	91.6%	Dang et al. (2025)[33]
		$Ni(OH)_x$/Cu	1 M KOH (0.1 M NO_3^-)	3 $mmol \cdot h^{-1} \cdot cm^{-2}$	92%	Liu et al. (2024)[34]
		Cu SAGs	0.1 M PBS, pH = 7.0, and 20 mM NO_3^-	/	97%	Li et al. (2023)[35]
**Photo-catalysis**	N_2 direct reduction	Fe-SA/$WO_{2.72-x}$	Ultrapure water	186.5 $\mu mol \cdot h^{-1} \cdot cm^{-2}$	/	Hu et al. (2022)[36]
		Fe doped MoO_{3-x} nanowires	N_2-saturated solution	742.92 $\mu mol \cdot g^{-1} \cdot h^{-1}$	/	Yu et al. (2025)[37]
		Carbon shell	Ultrapure	52.6	/	Ren et al.

		coated defective WO_3	water (2 %methanol)	$\mu mol \cdot g^{-1} \cdot h^{-1}$		(2025)[38]
	NO_3^- photo-reduction	$MoS_{2-x}/ZnIn_2S_{4-x}$ heterojunction	KNO_3 solution (30 $mg \cdot L^{-1}$)	185.59 $\mu mol \cdot g^{-1} \cdot h^{-1}$	/	Zhang et al. (2024)[39]
		Cu-TiO_2	0.01 M KNO_3	~0.49 $\mu g \cdot g^{-1} \cdot h^{-1}$	/	Hiramatsu et al. (2024)[40]
**Liquid metal-catalysis**	Liquid metal-catalysis synthesis	Cu-Ga	/	3,400 $\mu mol \cdot g^{-1} \cdot h^{-1}$	/	Guan et al. (2024)[41]
		$CuGa_2$	/	10,000 $\mu mol \cdot g^{-1} \cdot h^{-1}$	/	Zeng et al. (2025)[42]

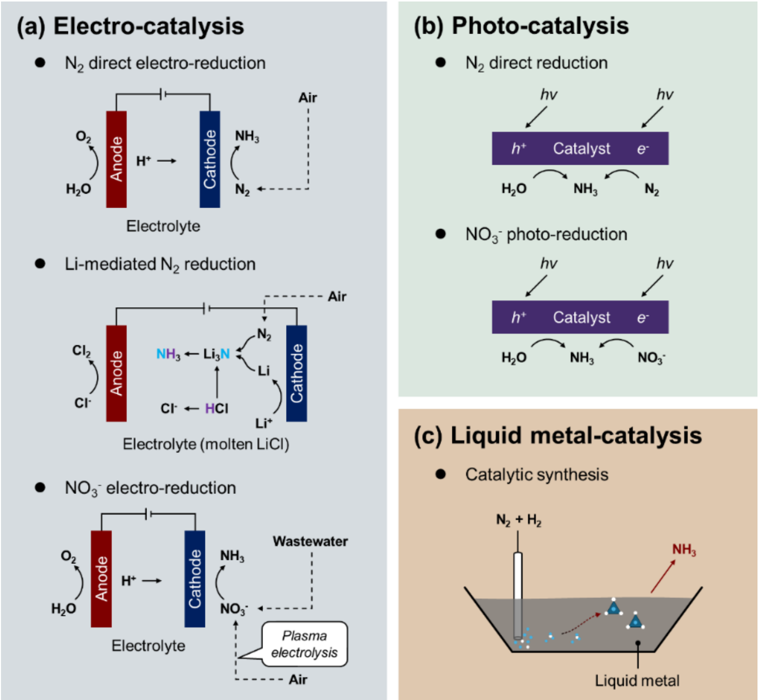

Supplementary Fig. 3 | Schematic representation of emerging alternative NH_3 synthesis pathways. a, Electro-catalytic strategies, encompassing direct nitrogen reduction (N_2 direct electro-reduction), lithium-mediated nitrogen reduction (Li-mediated N_2 reduction), and nitrate electro-reduction (NO_3^- electro-reduction). **b**, Photo-catalytic approaches utilizing light energy (hν) to drive direct N_2 reduction and NO_3^- photo-reduction over semiconductor catalysts. **c**, Liquid metal-catalyzed synthesis, where liquid metals facilitate the reaction between gaseous N_2 and H_2.

2.4 The transformation from Na_2SO_4 to Na_2S via carbothermal reduction is well-known (e.g., historically used in the Leblanc process). The subsequent reaction of Na_2S with CO_2 to form Na_2CO_3 + S_x, while interesting, does not represent a conceptual breakthrough. The authors should emphasize what is truly new—e.g., the integration of the two steps, the avoidance of SO_x emissions, or the application to impure streams—rather than implying fundamental discovery of the core chemistry.

Reply: We sincerely appreciate this constructive feedback. We fully agree with your assessment that the carbothermal reduction of Na_2SO_4 is historically established (e.g., the Leblanc process) and that the core chemical equations do not, in isolation, represent a fundamental discovery of new reactivity. In review of the historic developments of soda production, the core chemistry is the reaction between Na_2S and CO_2, which is highly sensitive to the operating temperature. We discovered this phenomenon and verified the temperature-controllable reaction by both thermodynamic and experimental analysis.

Therefore, as you rightly suggested, the true innovation of our work lies in process integration and environmental engineering, specifically regarding the clean transfer pathway of sulfur. We have clarified these distinctions as follows:

1. **Solving the historical "Leblanc waste" problem:** The historical Leblanc process generated massive pollution in the form of CaS waste, which released toxic H_2S. In distinct contrast, our approach couples the reduction step with a novel controlled carbonation step that recovers sulfur as S_x. This represents a critical divergence from historical methods, effectively turning a potential pollutant into a storable resource without generating SO_x emissions.
2. **Feedstock flexibility and impurity tolerance:** Unlike historical methods requiring pure feedstocks, our process is specifically engineered to valorize both pure and impure mixed-salt waste streams (e.g., from battery recycling or flue-gas desulfurization). Crucially, we demonstrate robust conversion efficiency even in the presence of contaminants like NaCl, enabling the continuous reuse of the regenerated product for secondary SO_2 removal (**Fig. R1-4**, i.e., **Fig. 5c** in the **Manuscript**).

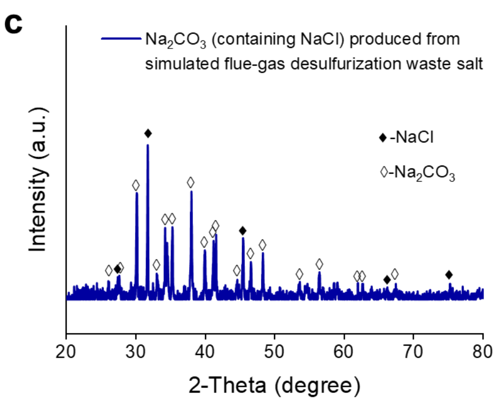

Fig. R1-4 | XRD pattern of the product synthesized from simulated flue-gas desulfurization waste salt, demonstrating the tolerance of this work to NaCl impurities.

3. **Closed-loop integration:** The novelty also lies in the design of a continuous closed-loop system that enables the *in-situ* regeneration of Na_2CO_3 from sulfate streams, specifically for reuse in Li extraction or recovery (**Fig. R1-5b**). This significantly reduces the industrial footprint by minimizing external reagent procurement.

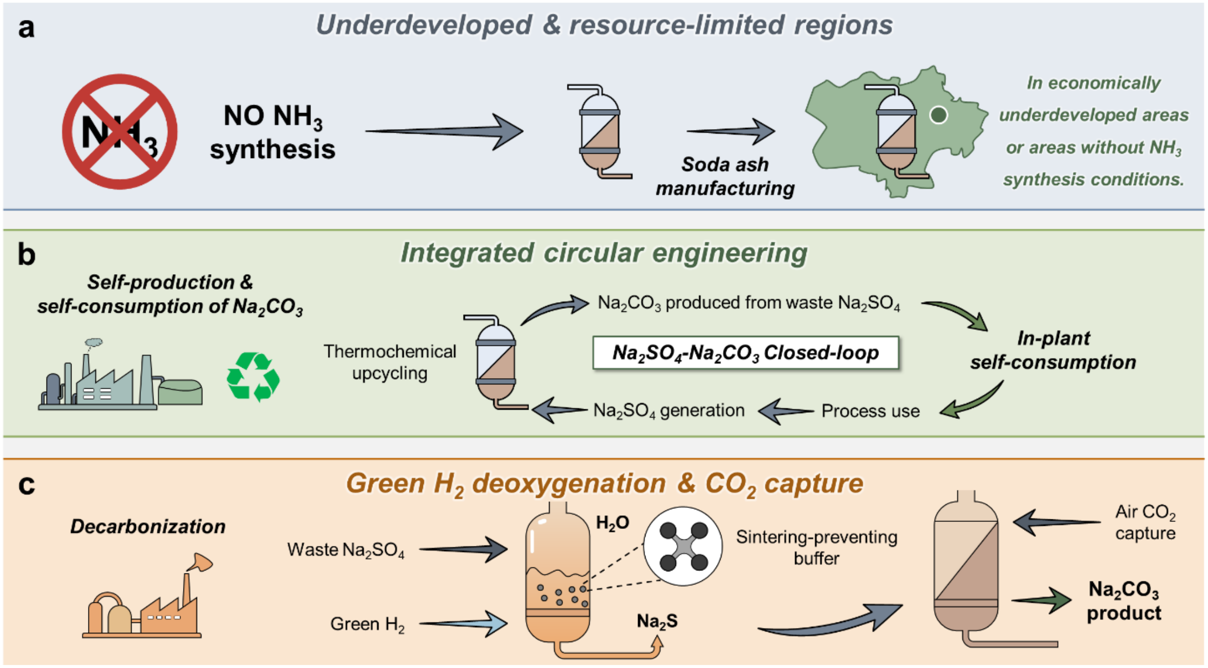

Fig. R1-5 | Future perspectives on industrial deployment and low-carbon evolution of the proposed technology. a, Deployment feasibility for economically underdeveloped regions or areas lacking synthetic NH_3 infrastructure, enabling decentralized, NH_3-free soda ash manufacturing. **b**, Strategy for integrated circular engineering to establish an on-site Na_2SO_4-Na_2CO_3 closed-loop, realizing internal self-sufficiency. **c**, Advanced decarbonization pathway coupling green H_2-based deoxygenation (utilizing sintering-preventing buffers in a fluidized bed) with direct air CO_2 capture.

In accordance with your suggestion, we have comprehensively revised the **Manuscript** to explicitly frame this work as a “sustainable process intensification” strategy. We now emphasize that the integration of these two steps (the clean fixation of S_x and the upcycling of industrial waste) constitutes the primary contribution of this study. Furthermore, we have expanded the discussion to propose a broader strategy for recovering other waste sulfates by converting them into Na_2SO_4 for subsequent sulfur recovery (**Fig. R1-5**).

2.5 High-Temperature Process Lacks Energy Innovation. The proposed process requires temperatures ≥750°C, which is highly energy-intensive. The authors do not address: The source of low-carbon process heat required for decarbonization. How the energy efficiency compares to electrochemical route for Na_2SO_4 valorization. The authors must provide a realistic energy-balance analysis and discuss the scalability of their process in a renewables-powered industrial context.

Reply: Thank you for your valuable feedback. We fully acknowledge the concerns regarding the high energy demand of the proposed process, especially with the requirement for temperatures ≥750 °C, which indeed presents significant energy challenges. As you rightly pointed out, one critical aspect that requires further attention is the source of low-carbon process heat necessary for the reduction step (which we understand should refer to $Na_2SO_4 \rightarrow Na_2S$, rather than decarbonization). In response to your comments, we have expanded the discussion on potential sources of process heat. One possibility is the integration of renewable energy, such as concentrated solar power (CSP) or Fresnel lenses, which can provide the required high temperatures in a sustainable manner. Additionally, waste heat recovery systems from other industrial processes, such as coupling the waste Na_2SO_4 generated from lithium extraction with the waste heat from spodumene roasting, could further enhance the overall energy efficiency of the system (**Fig. R1-2**).

Regarding the energy efficiency comparison with the electrochemical route for Na_2SO_4 valorization (producing NaOH and H_2SO_4), we acknowledge that electrochemical processes are typically energy-efficient due to their operation at temperatures below 100 °C. However, it is crucial to note that current electrolysis technologies generally require high-purity feedstocks and have not been widely validated for the mixed-purity Na_2SO_4 waste streams common in industry.

While the electrochemical route is indeed a promising alternative, we believe our thermochemical method offers distinct advantages in terms of robustness, particularly regarding scalability and the capacity to valorize complex, impure waste streams.

To substantiate this, we have conducted a preliminary energy-balance analysis (**Fig. R1-6**), which is now included in the **Revised Supplementary Information**. This analysis highlights a unique advantage of our approach:

Adaptability to fluctuating energy: This work—specifically the energy-intensive dehydration and evaporation stages—demonstrates high compatibility with intermittent or “unstable” thermal energy

sources (e.g., solar thermal or industrial waste heat). Unlike electrochemical processes, which require a strictly stable electricity supply to maintain efficiency and protect membrane components, our thermal-driven approach is inherently more resilient to energy fluctuations.

We should point out that the energy-consumption calculation is based on lab-scale experiments, which does have some deviation from the large-scale applications. In the current stage, we mainly focus on the novel chemistry and the design of the closed loop. We believe these additions provide a more realistic and comprehensive assessment of the process's energy balance and scalability. We hope these clarifications satisfactorily address your comments, and we thank you again for your thoughtful feedback.

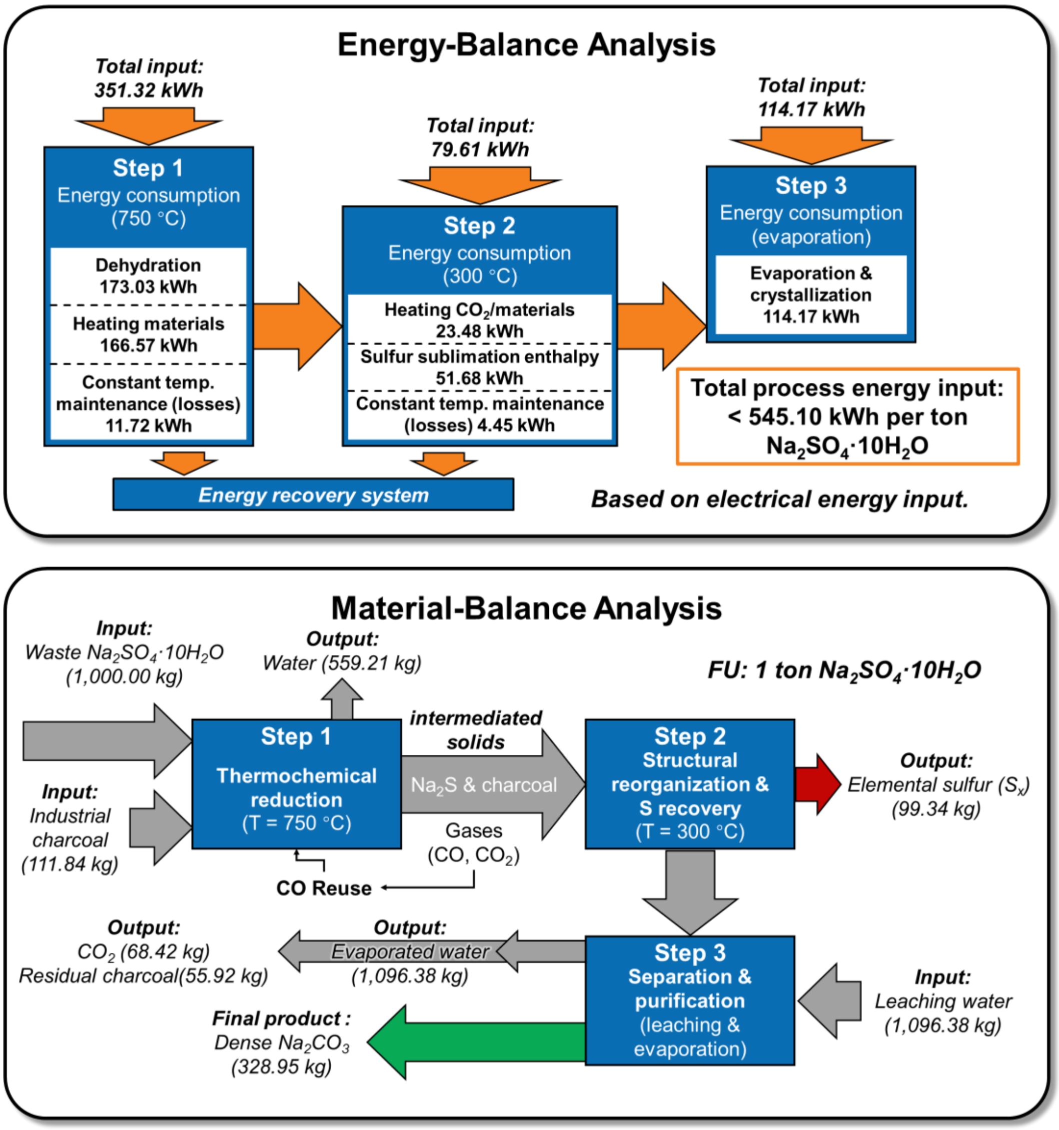

Fig. R1-6 | Energy- and material-balance analysis for thermochemical upcycling of 1,000 kg waste $Na_2SO_4 \cdot 10H_2O$. The energy-balance analysis reveals that the total process energy input is <

545.10 kWh per ton of $Na_2SO_4 \cdot 10H_2O$. Notably, the dehydration (**Step 1**, 173.03 kWh) and evaporation (**Step 3**, 114.17 kWh) processes collectively account for approximately 52.7% of the total energy consumption. As these steps are driven primarily by thermal demand, they offer a significant avenue for decarbonization by coupling with intermittent renewable sources, such as solar thermal energy or industrial waste heat, instead of relying solely on grid electricity.

Revise:

The Revised Results

Consequently, the total end-point environmental impact of this work (34.69 mPt per kg Na_2CO_3) is significantly lower than that of competitive routes (**Fig. 5e** and **Supplementary Table 9**). Although the cumulative energy demand (CED) appears marginally higher than the SSA-Process (**Fig. 5f** and **Supplementary Table 10**), contribution analysis clarifies a critical distinction: a substantial fraction of this demand is derived from renewable biomass (charcoal) rather than fossil fuels (**Supplementary Fig. 33**). Furthermore, energy-balance analysis further indicates that 52.7% of the total energy is associated with dehydration and evaporation, processes that are highly adaptable to intermittent clean thermal sources (e.g., solar thermal energy or industrial waste heat) rather than grid electricity (**Supplementary Fig. 34**). Under a projected PV-driven scenario, our technology maintains the lowest environmental footprint while achieving a competitive CED (**Supplementary Fig. 36**). We should point out that the energy-consumption calculation is based on lab-scale experiments, which may have some deviation from large-scale applications. Overall, our technology demonstrates exceptional potential across multiple sustainability metrics (**Fig. 5g** and **Supplementary Note 3**), with the observed gap in energy-saving potential likely reflecting the current maturity of industrial NH_3 synthesis infrastructure compared to emerging thermochemical routes.

The Revised Supplementary Information

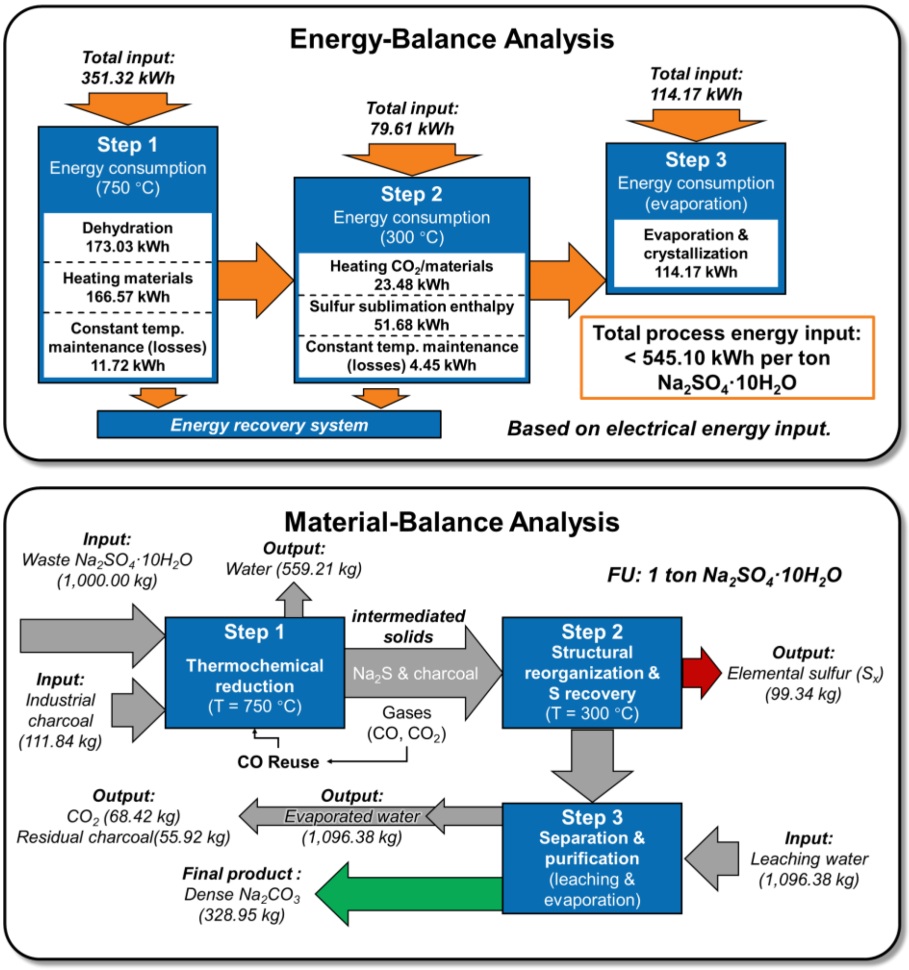

Supplementary Fig. 34 | Energy- and material-balance analysis for thermochemical upcycling of 1,000 kg waste $Na_2SO_4 \cdot 10H_2O$. The energy-balance analysis reveals that the total process energy input is < 545.10 kWh per ton of $Na_2SO_4 \cdot 10H_2O$. Notably, the dehydration (**Step 1**, 173.03 kWh) and evaporation (**Step 3**, 114.17 kWh) processes collectively account for approximately 52.7% of the total energy consumption. As these steps are driven primarily by thermal demand, they offer a significant avenue for decarbonization by coupling with intermittent renewable sources, such as solar thermal energy or industrial waste heat, instead of relying solely on grid electricity.

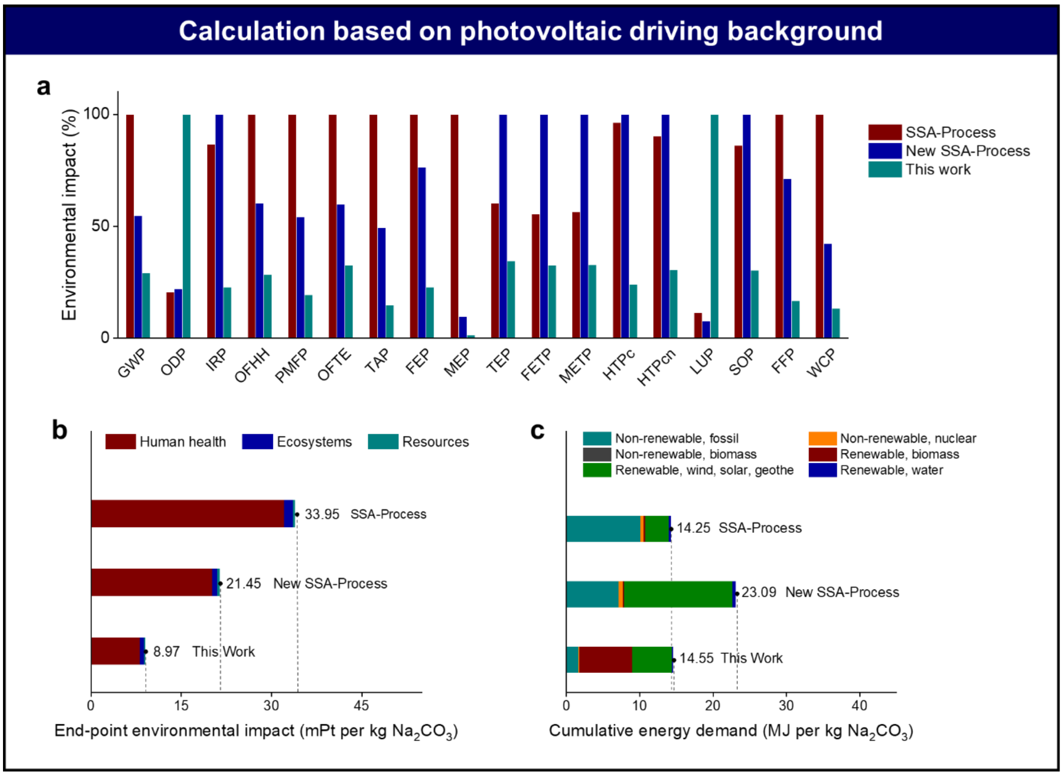

Supplementary Fig. 36 | Comparative assessment of environmental impacts and CED under the PV-driven scenario. a, Comparison of mid-point environmental impact categories for the SSA-Process, New SSA-Process, and the proposed thermochemical route (this work). **b**, Comparison of end-point environmental impact results (measured in mPt). **c**, Comparison of CED results (measured in MJ).

3. Data and Methodology

The experimental data are of high quality and well-presented. The supplementary information is comprehensive. The LCA is methodologically standard but is applied to an outdated benchmark (a conventional SSA-Process), limiting the usefulness of its conclusions.

Reply: We sincerely thank you for your positive evaluation of our data quality, experimental rigor, and the comprehensiveness of our supplementary information. We also deeply value your constructive critique regarding the benchmarks used in our LCA. While we initially selected the conventional SSA-Process as the primary baseline because it remains the dominant industrial route currently in implementation, we fully agree with your view that LCA studies must be forward-looking to effectively guide future technology deployment.

To address your concerns and enhance the long-term applicability of our conclusions, we have significantly expanded the scope of our LCA:

1. Integration of advanced benchmarks: We comprehensively evaluated the environmental impacts and CED of emerging technologies, specifically focusing on PV-driven green NH_3 synthesis and MEA-MEG solvent extraction for high-purity $(NH_4)_2SO_4$. under the current energy background, deploying these advanced technologies actually results in higher CO_2 emissions compared to the traditional SSA process. This arises because conventional Haber-Bosch ammonia production remains less carbon-intensive than electrolytic synthesis powered by the current fossil-dominated electricity mix. However, the scenario shifts dramatically with the future proliferation of photovoltaics. In a PV-dominated future, the New SSA-Process (i.e., green NH_3 and MEA-MEG anti-solvent crystallization) is projected to reduce CO_2 emissions by approximately 0.54 kg per kg of Na_2CO_3, demonstrating immense decarbonization potential (**Table R1-1, Global warming**).

Table. R1-1 | Mid-point environmental impact assessment results of the Na_2CO_3 production process. Values in parentheses represent the photovoltaic (PV)-driven scenario.

Impact category	Unit	SSA-Process	New SSA-Process	This work
Global warming	kg CO_2 eq	1.758042084 (1.189936703)	3.335624607 (0.649737929)	1.329170016 (0.346158546)
Stratospheric ozone depletion	kg CFC11 eq	2.32902E-07 (1.56829E-07)	5.28168E-07 (1.68509E-07)	8.96357E-07 (7.64725E-07)

Ionizing radiation	kBq Co-60 eq	0.049750078 (0.024942653)	0.146108834 (0.028824375)	0.04947613 (0.006551024)
Ozone formation, Human health	kg NO_x eq	0.003507984 (0.001951449)	0.008535849 (0.001176866)	0.00324818 (0.000554856)
Fine particulate matter formation	kg PM2.5 eq	0.00248644 (0.001644234)	0.004874339 (0.000892563)	0.00177733 (0.000320034)
Ozone formation, Terrestrial ecosystems	kg NO_x eq	0.003624663 (0.002067919)	0.008598301 (0.001238335)	0.003365817 (0.000672132)
Terrestrial acidification	kg SO_2 eq	0.005798459 (0.003908322)	0.010859342 (0.001923159)	0.003849499 (0.000578933)
Freshwater eutrophication	kg P eq	0.000403585 (0.000322918)	0.000627947 (0.000246571)	0.000213107 (7.3527E-05)
Marine eutrophication	kg N eq	0.000885644 (0.000880121)	0.000110359 (8.4246E-05)	2.24994E-05 (1.29422E-05)
Terrestrial ecotoxicity	kg 1,4-DCB	4.469192213 (5.469660885)	4.360302483 (9.090314903)	1.38992887 (3.121072801)
Freshwater ecotoxicity	kg 1,4-DCB	0.045499119 (0.057739294)	0.046421938 (0.104290998)	0.012664453 (0.033844032)
Marine ecotoxicity	kg 1,4-DCB	0.060142768 (0.075031623)	0.06276473 (0.133156209)	0.017671811 (0.043434487)
Human carcinogenic toxicity	kg 1,4-DCB	0.065114516 (0.052332345)	0.114810628 (0.054379127)	0.035127132 (0.013009721)
Human non-carcinogenic toxicity	kg 1,4-DCB	1.093827993 (0.999403096)	1.555439581 (1.109017864)	0.502556473 (0.339169956)
Land use	m^2a crop eq	0.024402616 (0.018582822)	0.040037012 (0.012522209)	0.174129293 (0.164059112)
Mineral resource scarcity	kg Cu eq	0.003280938 (0.00367873)	0.002393923 (0.004274601)	0.000603257 (0.001291569)
Fossil resource scarcity	kg oil eq	0.326838693 (0.219024176)	0.665764832 (0.156039725)	0.222939757 (0.036384744)
Water consumption	m^3	0.024067092 (0.020252414)	0.026575589 (0.008540566)	0.009263805 (0.002663141)

2. Inclusion of BMED: To provide a comprehensive evaluation of waste Na_2SO_4 valorization technologies, we have broadened our study to include an LCC of bipolar membrane electrodialysis (BMED), which produces H_2SO_4 and NaOH solution from waste streams (**Table. R1-2**).

In conclusion, thank you for your critical suggestions on our research, which have greatly improved the quality of our **Revised Manuscript**.

Table. R1-2 | Summary of life cycle costs (LCC) for BMED (yielding H_2SO_4 and NaOH), the SSA-Process, the New SSA-Process, and this work. The assessment is based on a functional unit of processing 1,000 kg of waste $Na_2SO_4 \cdot 10H_2O$. Calculations utilize average market prices over the past five years (see **Supplementary Fig. 35** for historical price fluctuations of all products). Primary values are calculated based on coal-fired electricity generation, while values in parentheses represent a future PV-driven scenario.

Parameter	BMED	New SSA-Process	This work
**1. Material & Energy Costs (USD)**			
Feedstock			
Waste $Na_2SO_4 \cdot 10H_2O$	0.00[a]	0.00[a]	0.00[a]
Energy			
Electricity	439.90[b] (189.16)[b]	219.47[b] (94.37)[b]	54.51[b] (23.44)[b]
Chemical Reagents			
Charcoal (Reactant)	—	—	22.37[c]
NH_3	—	—	—
CO_2	—	4.78[d]	—
Process water/solvent	13.53[e]	18.00[f]	1.64[e]
**Total Costs (USD)**	**453.43 (202.69)**	**242.25 (117.15)**	**78.52 (47.45)**
**2. Revenue Streams (USD)**			
Main Product			
H_2SO_4	34.17[g]	—	—
NaOH	113.25[h]	—	—
Na_2CO_3	—	97.04	97.04
Byproducts			
$(NH_4)_2SO_4$	—	109.79[i]	—
S_x	—	—	18.28
**Total Revenue (USD)**	**147.42**	**206.83**	**115.32**
**3. Economic Performance (USD)**			
**Gross Profit**	**−306.01 (−55.27)**	**−35.42 (+89.68)**	**+36.80 (+67.87)**
**Profit Margin (%)**	**−207.58% (−37.49%)**	**−17.13% (+43.36%)**	**+31.91% (+58.85%)**

a: Waste $Na_2SO_4 \cdot 10H_2O$ is assumed to be burden-free (0.00 USD) as it is a waste stream.

b: Baseline electricity cost is set at 0.10 USD·kWh^{-1}. A sensitivity scenario considers a reduced rate of 0.043 USD·kWh^{-1}, reflecting the global weighted average cost of utility-scale solar PV (Source: IRENA, https://www.irena.org/).

c: Charcoal (reductant) is priced at 400 USD·ton^{-1} based on prevailing market rates in China; future adoption of low-cost alternative carbon sources could further reduce expenses.

d: Industrial CO_2 is priced at 35 USD·ton^{-1}, reflecting capture costs in the Chinese region (Source: CCUS and Global Power Generation Analysis)[32].

e: Process water is valued at 1.50 USD·ton^{-1}.

f: Solvent costs are based on Chinese market prices: MEA at 1,000 USD·ton^{-1} and MEG at 500 USD·ton^{-1}. A makeup rate of 5% per cycle is assumed to account for operational losses.

g: Product revenue for NaOH and H_2SO_4 is calculated on a 100% pure substance basis (e.g., the output of NaOH is equivalent to 250 kg of pure NaOH).

h: Same as note **g**; H_2SO_4 production is also calculated on a 100% basis.

i: The market value of $(NH_4)_2SO_4$ is estimated at 150% of the standard fertilizer-grade price. This premium reflects the higher purity and economic value achieved through the MEA-MEG purification process compared to conventional fertilizer products.

Limitations: The economic analysis is restricted to the variable operating expenditures (OPEX) associated with material and energy inputs. Fixed costs, including capital expenditures (CAPEX) for plant construction, equipment depreciation, and labor costs, are excluded from this assessment.

The XRD and SEM data are supportive but do not sufficiently prove the absence of intermediates or side products. In situ XRD or XAS during the Na_2S + CO_2 step would strengthen the mechanism.

Reply: We sincerely appreciate this valuable suggestion. We fully agree that *in-situ* XRD or XAS techniques would provide the most direct evidence for the reaction mechanism of the Na_2S + CO_2 step. However, we would like to respectfully clarify the significant technical challenges associated with implementing *in-situ* XRD or XAS for this specific reaction ($Na_2S + CO_2 \rightarrow Na_2CO_3 + S_x$):

1. Hygroscopic challenge of the intermediate (Na_2S): Anhydrous Na_2S is highly hygroscopic and sensitive to air. Any sample transfer process carries the risk of forming the hydrated phase ($Na_2S{\cdot}5H_2O$, as evidenced in **Fig. 2c** of the **manuscript**). This hydration introduces impurities and experimental artifacts that would interfere with the interpretation of the true reaction results. It is important to note, however, that this hygroscopicity does not affect the practical viability of our developed Na_2SO_4-to- Na_2CO_3 conversion process. In our integrated system, the intermediate Na_2S undergoes immediate carbonation with CO_2 without any exposure to air, effectively bypassing the hydration issue entirely.

2. Interference from sulfur vapor: The reaction operates at 300 – 350 °C, requiring a specialized high-temperature *in situ* chamber. Crucially, the continuous generation of S_x vapor presents a severe obstacle. The sulfur vapor tends to condense and deposit on the observation windows of the *in-situ* cell (blocking signal acquisition) and accumulate in the cooler downstream gas lines, causing blockages.

3. Equipment safety: This deposition not only leads to inaccurate data collection but also poses a significant risk of permanent damage to delicate *in-situ* equipment. Consequently, we were unable to secure an external testing agency willing to execute this specific experiment due to the high risk of equipment contamination and damage from sulfur deposition.

Although performing *in-situ* XRD or XAS analysis for the Na_2S + CO_2 step was technically challenging, we directly addressed your concerns regarding reaction pathways and byproducts by establishing a custom gas collection apparatus to conduct detailed GC-MS analysis (**Fig. R1-7**). Crucially, our results confirm the complete absence of detectable SO_x species, aligning perfectly with our thermodynamic equilibrium calculations and validating the environmental cleanliness of the process. The gas phase is dominated by the CO_2 carrier gas and the CO reduction product. Semi-quantitative analysis revealed that Carbonyl Sulfide (COS) constitutes less than 2% of the gaseous

byproducts. We attribute this trace formation to the spontaneous recombination of CO and sulfur vapor, noting that such minor impurities can be readily removed via standard industrial separation techniques (e.g., condensation, b.p.: −50.4 °C) without impacting the overall process viability. We sincerely thank you for prompting this investigation, as it was crucial for fully elucidating the reaction chemistry. These detailed findings and discussions have now been incorporated into the **Revised Manuscript** and **Revised Supplementary Information**.

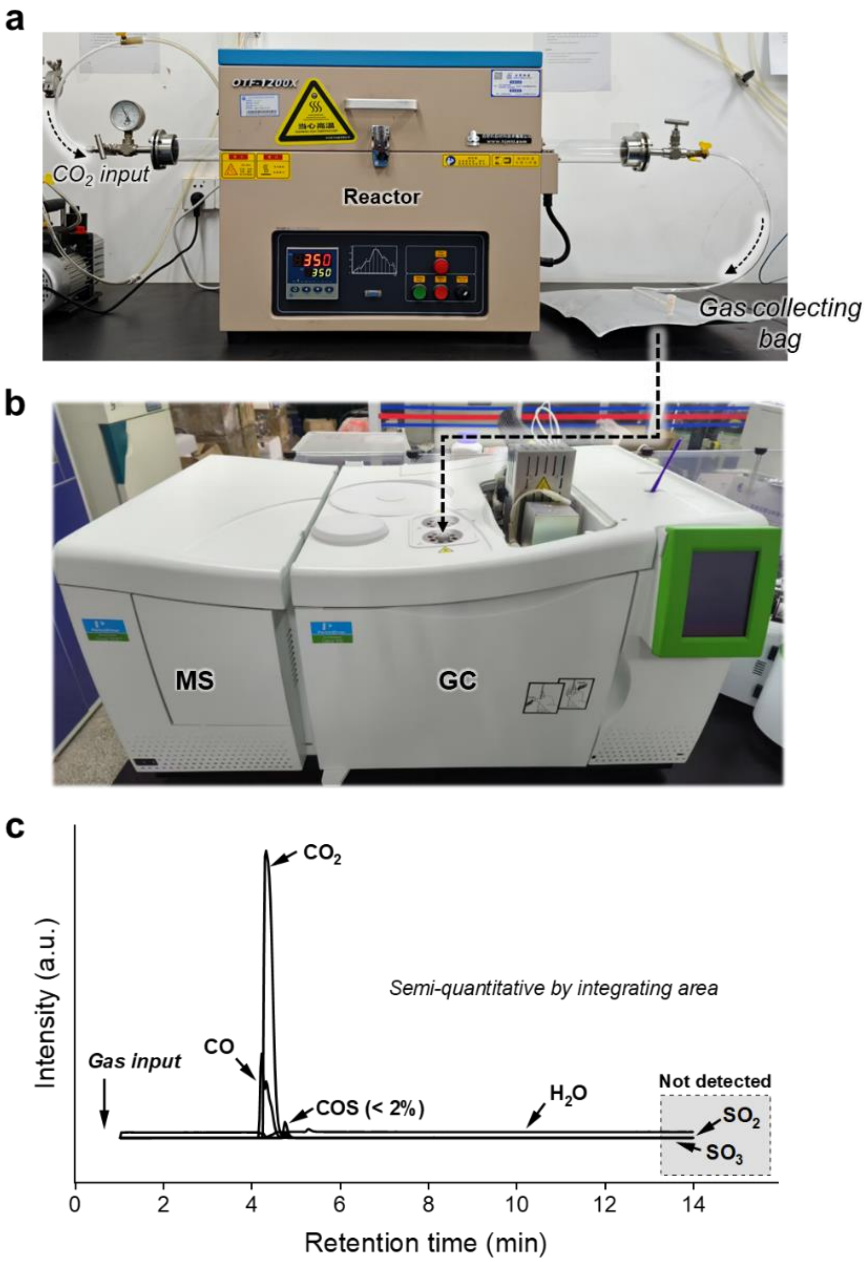

Fig. R1-7 | Investigation of potential side reactions between Na_2S and CO_2. a, Digital photograph of the experimental setup used for reaction gas collection. **b**, The GC-MS instrument. **c**, Total ion chromatogram (TIC) identifying the gaseous byproducts. Test conditions: The system was operated in

full scan mode to capture the complete spectrum of each data point during the chromatographic analysis. A 1 μL gas sample was injected into the chromatographic column with He (99.999%) as the carrier gas at a flow rate of 1.0 mL·min^{-1}. The temperature program involved holding at 40 °C for 2 min, followed by a ramp to 300 °C at a rate of 8 °C·min^{-1}. The mass selective detector's electron impact (EI) ion source temperature was set at 200 °C, and the mass spectra were analyzed in the range of 0 – 300 atomic mass units (amu).

Revise:

The Revised Results

Mechanistically, this transformation is driven by a strong acid-base neutralization reaction, where acidic CO_2 reacts readily with alkaline Na_2S within the thermodynamically favorable window of 200 – 400 °C. The C buffer plays a pivotal role here, preventing sintering and providing a high surface area for efficient gas-solid contact (**Fig. 4d**). To rigorously assess potential pollutant formation, the gas composition at 350 °C was analyzed by gas chromatography-mass spectrometry (GC-MS) (**Supplementary Fig. 26**). Crucially, the analysis confirmed the absence of detectable SO_x, validating the kinetic and thermodynamic inhibition of SO_x generation in this system. The product gas consisted almost exclusively of CO_2 (carrier gas) and CO (reduction byproduct), with only negligible traces of carbonyl sulfide (COS) observed. In summary, this NH_3-free thermochemical strategy achieves the clean, site-independent upcycling of waste Na_2SO_4 into high-value Na_2CO_3, successfully overcoming the scalability and pollution constraints inherent in conventional precipitation-based routes.

The Revised Supplementary Information

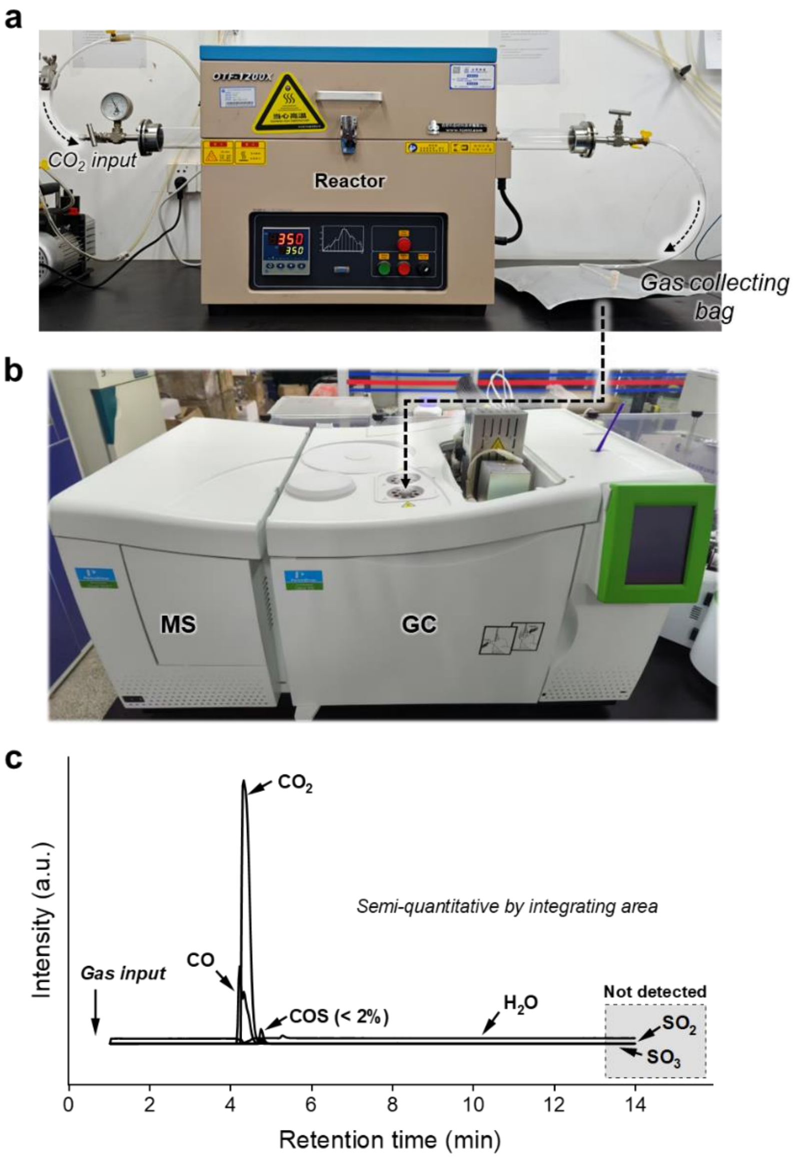

Supplementary Fig. 26 | Investigation of potential side reactions between Na_2S and CO_2. a, Digital photograph of the experimental setup used for reaction gas collection. **b**, The GC-MS instrument. **c**, Total ion chromatogram (TIC) identifying the gaseous byproducts. Test conditions: The system was operated in full scan mode to capture the complete spectrum of each data point during the chromatographic analysis. A 1 μL gas sample was injected into the chromatographic column with He (99.999%) as the carrier gas at a flow rate of 1.0 mL·min^{-1}. The temperature program involved holding at 40 °C for 2 min, followed by a ramp to 300 °C at a rate of 8 °C·min^{-1}. The mass selective detector's electron impact (EI) ion source temperature was set at 200 °C, and the mass spectra were analyzed in the range of 0 – 300 atomic mass units (amu).

4. Analytical Approach

The analytical approach is appropriate and comprehensive for validating the chemical process. The LCA approach is standard but would benefit from a scenario analysis that includes a modern benchmark (green NH_3 + advanced separation). A comparison against a future, decarbonized SSA-Process utilizing green PV-NH_3 is absent. The authors must justify the significant energy demand of their own high-temperature process (750-800 °C) against this competing decarbonization pathway. A revised life-cycle assessment should include a scenario where the SSA-Process uses green ammonia to provide a fair and contemporary comparison.

Reply: We sincerely appreciate your constructive criticism regarding the benchmarks used in our LCA. We fully agree that a fair comparison requires benchmarking the SSA-Process against models incorporating modern green NH_3 and MEA-MEG solvent extraction to provide rational guidance for future industrial deployment. Accordingly, we have revised the LCA in the **Revised Manuscript** and **Revised Supplementary Information** to include a comparative analysis with this future decarbonized SSA-Process (New SSA-Process) scenario. It is important to note that the LCA data for green NH_3 was adopted directly from established literature (*International Journal of Hydrogen Energy*, **2025**, 107: 270-278) to ensure data reliability and objectivity since modeling the complex green NH_3 synthesis process from scratch is outside our primary expertise. Furthermore, we highlight that our thermochemical process possesses the distinct potential to be driven by direct solar thermal energy, which bypasses the thermodynamic losses inherent in the solar-to-electricity-to-heat conversion chain. Our CED calculations indicate that, even under China's current grid structure, our technology maintains a lower energy consumption per ton of Na_2CO_3 compared to the New SSA-Process (20.21 MJ vs. 38.55 MJ, **Fig. R1-8**). We acknowledge that the SSA-Process currently exhibits the lowest total energy demand; however, a significant portion of the energy input in our proposed technology is derived from renewable biomass, offering a distinct sustainability advantage. Crucially, as the energy structure transitions toward a PV scenario, "This work" continues to demonstrate a lower energy demand compared to the New SSA-Process (**Fig. R1-9**). Nevertheless, we recognize that the New SSA-Process also holds substantial potential for future industrial application, as it becomes primarily driven by renewable electricity in such a landscape.

We believe these revisions robustly validate the viability and low-carbon potential of our

thermochemical conversion strategy within a decarbonized landscape.

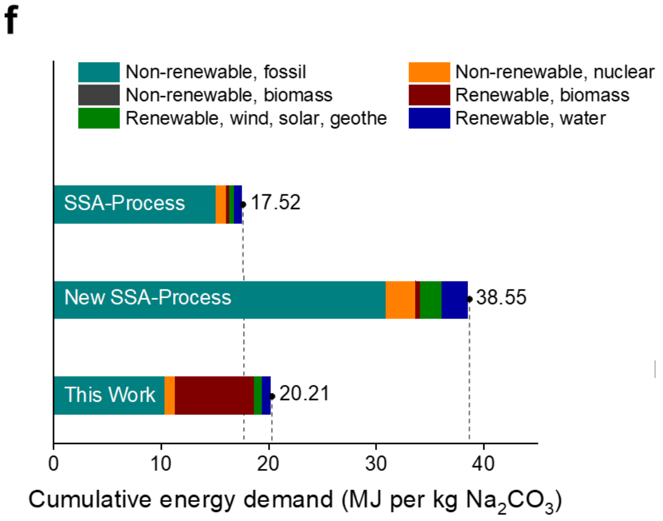

Fig. R1-8 | Cumulative energy demand under China's current grid structure.

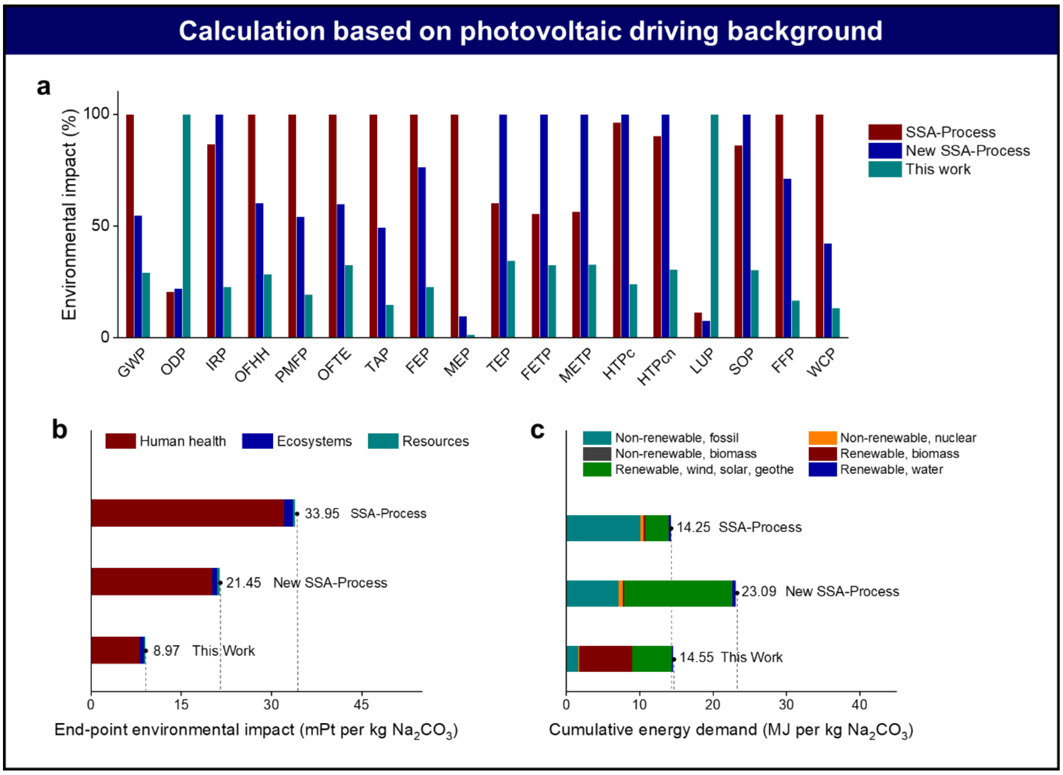

Fig. R1-9 | Comparative assessment of environmental impacts and CED under the PV-driven scenario. a, Comparison of mid-point environmental impact categories for the SSA-Process, New SSA-Process, and the proposed thermochemical route (this work). **b**, Comparison of end-point environmental impact results (measured in mPt). **c**, Comparison of CED results (measured in MJ).

5. Suggested Improvements

To elevate the manuscript to a level suitable for a high-impact journal, major revisions in framing and analysis are required:

5.1 Recast the introduction and discussion to focus on valorizing low-value or impure sulfate streams (e.g., from flue-gas desulfurization) rather than treating Na_2SO_4 as a generic "waste."

Reply: We sincerely appreciate your strategic guidance in refining the positioning of our work. While our initial classification of Na_2SO_4 as "waste" stemmed from the current global scenario where production significantly exceeds consumption, we apologize for any confusion caused by this terminology and have revised the **Introduction** to explicitly discuss the limitations of handling impure streams via conventional methods like BMED which suffers from membrane fouling or the SSA-Process which relies on NH_3. Our study addresses two critical objectives which are the management of surplus Na_2SO_4 and the valorization of impure sulfate streams. While our core investigation establishes the reaction pathways and scale-up parameters using pure Na_2SO_4, we have expanded the scope to include experiments containing NaCl as a common impurity in flue-gas desulfurization (**Fig. R1-4**). These results prove that the presence of NaCl does not inhibit the conversion to Na_2CO_3, offering a distinct advantage over BMED where Cl^- often compromises membrane integrity or product purity. To further validate the feasibility of processing impure waste from sectors like Li_2CO_3 extraction, we simulated a closed-loop cycle (Na_2CO_3-to-Na_2SO_4-to-Na_2CO_3) (**Fig. R1-2**) to demonstrate the seamless parallel integration of our technology with existing industries. Consequently, we now frame this technology not merely as waste (surplus and impure Na_2SO_4) treatment but as a resource upcycling strategy for local industrial reuse, and your feedback has significantly enhanced the logical framework and relevance of our paper. All of our revisions have been recorded in the **Revised Manuscript** and **Revised Supplementary Information** for the revisions.

5.2 Discuss existing solutions for the SSA-Process's challenges, notably MEA antisolvent crystallization for efficient salt separation.

Reply: We sincerely appreciate your suggestion. In the revised Introduction, we have incorporated a discussion regarding existing solutions for the SSA-Process, specifically addressing the MEA-MEG anti-solvent crystallization technology. We have highlighted its relevance to efficient salt separation to provide a more comprehensive overview of the current state-of-the-art.

Revise:

The Revised Introduction

Recent advances in anti-solvent crystallization have notably improved Na_2SO_4 utilization and byproduct purity, underscoring the evolving potential of the SSA-Process[16].

Perform a Fair Comparative LCA: The most critical revision. The LCA must include a comparison against a modernized SSA-Process benchmark that utilizes: (a) green ammonia from PV-electrochemical synthesis, and (b) advanced MEA separation technology. This is essential to robustly claim any environmental advantage.

Reply: We sincerely appreciate this critical suggestion. We fully agree that establishing a modernized benchmark is essential for a robust environmental assessment.

In response, we have conducted a comprehensive comparative LCA against a newly defined "New SSA-Process," which explicitly incorporates: (**a**) green NH_3 derived from PV-electrochemical synthesis, and (**b**) advanced MEA-MEG anti-solvent separation technology. Furthermore, we extended the analysis beyond total impact scores to include an LCA contribution analysis. This allows for a detailed breakdown of environmental hotspots within the advanced benchmark compared to our proposed method. These comprehensive results confirm that our thermochemical route maintains distinct environmental advantages, particularly regarding energy adaptability and biogenic carbon utilization, even when compared against this modernized, high-standard benchmark. The detailed data and contribution breakdowns are presented in the **Revised Manuscript** and **Revised Supplementary Information**.

Revise:

The Revised Results

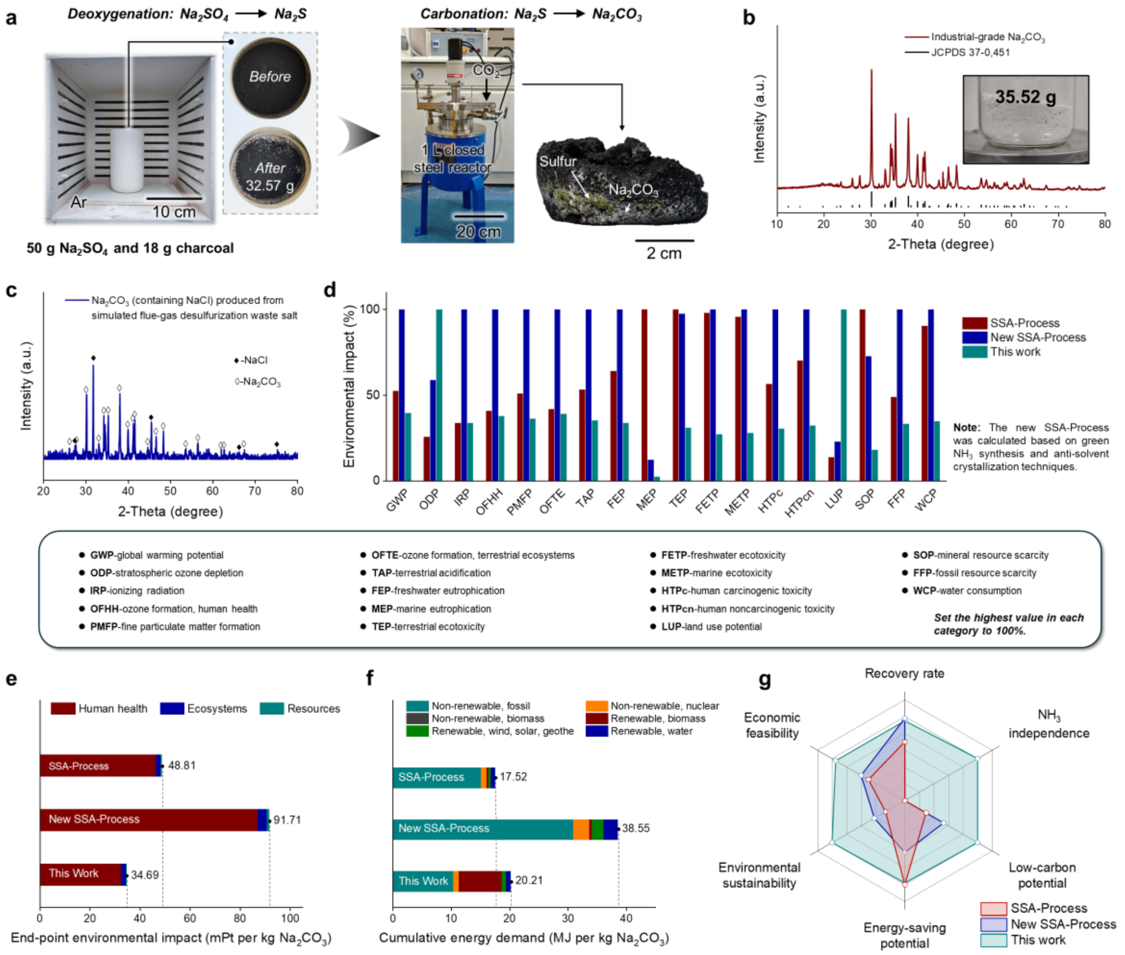

Fig. 5 | Scale-up production, LCA, and economic analysis. a, Workflow of the upscaled production using 50 g of waste Na_2SO_4 and 18 g of C, illustrating the transition from deoxygenation to carbonation. **b**, XRD pattern of the purified Na_2CO_3 obtained from the scale-up experiment. The inset photograph displays the final collected white powder (35.52 g). **c**, XRD pattern of the product synthesized from simulated flue-gas desulfurization waste salt, demonstrating the tolerance of this work to NaCl impurities. **d-f**, Comparative LCA results among the SSA-Process, New SSA-Process, and the method developed in this work: **d**, mid-point environmental impact categories; **e,** end-point environmental impact aggregated into human health, ecosystems, and resources; **f**, cumulative energy demand (CED). **g**, Radar chart comparing multidimensional performance metrics, including economic feasibility, recovery rate, NH_3 independence, low-carbon potential, energy-saving potential, and environmental

sustainability.

The Revised Supplementary Information

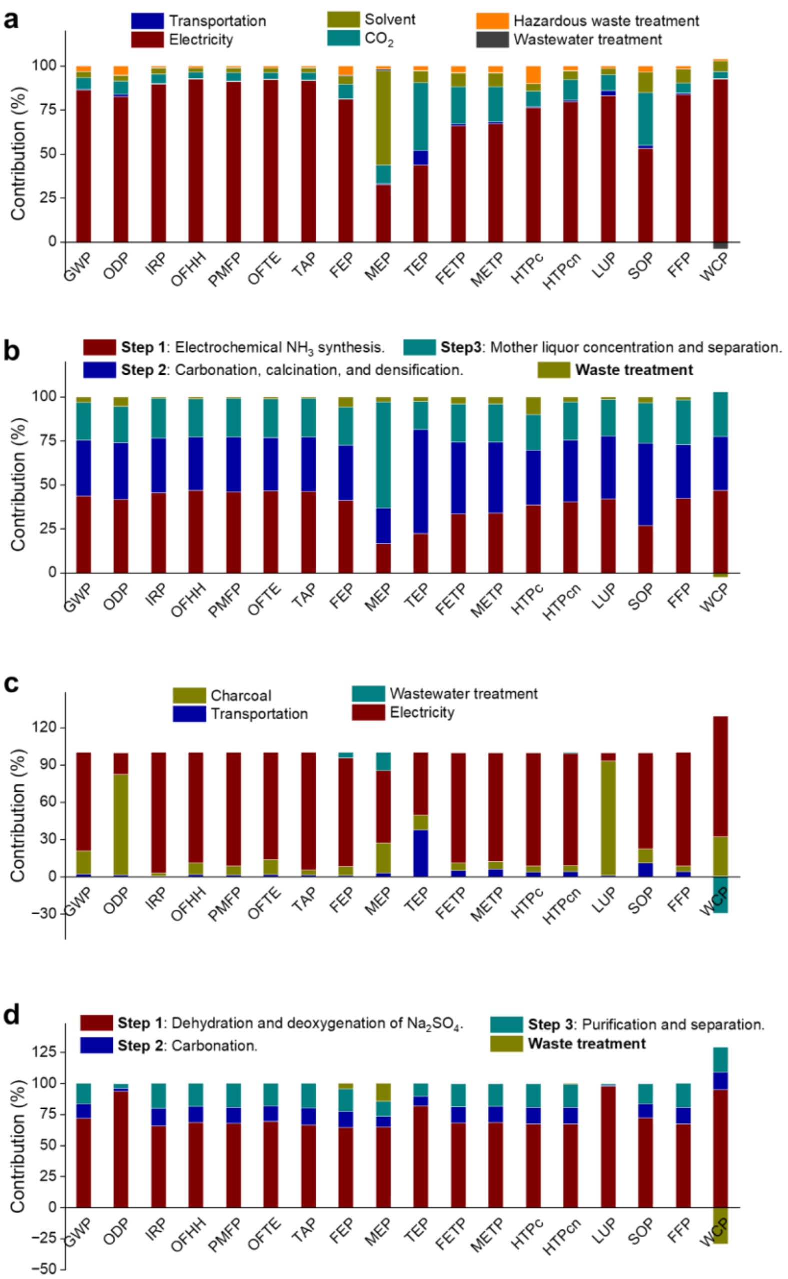

Supplementary Fig. 31 | Contribution analysis of mid-point environmental impact categories. a-b, Contribution analysis for the New SSA-Process, broken down by specific inventory inputs (**a**) and unit processes/production steps (**b**). **c-d**, Contribution analysis for the proposed thermochemical route (this work), broken down by specific inventory inputs (**c**) and unit processes/production steps (**d**).

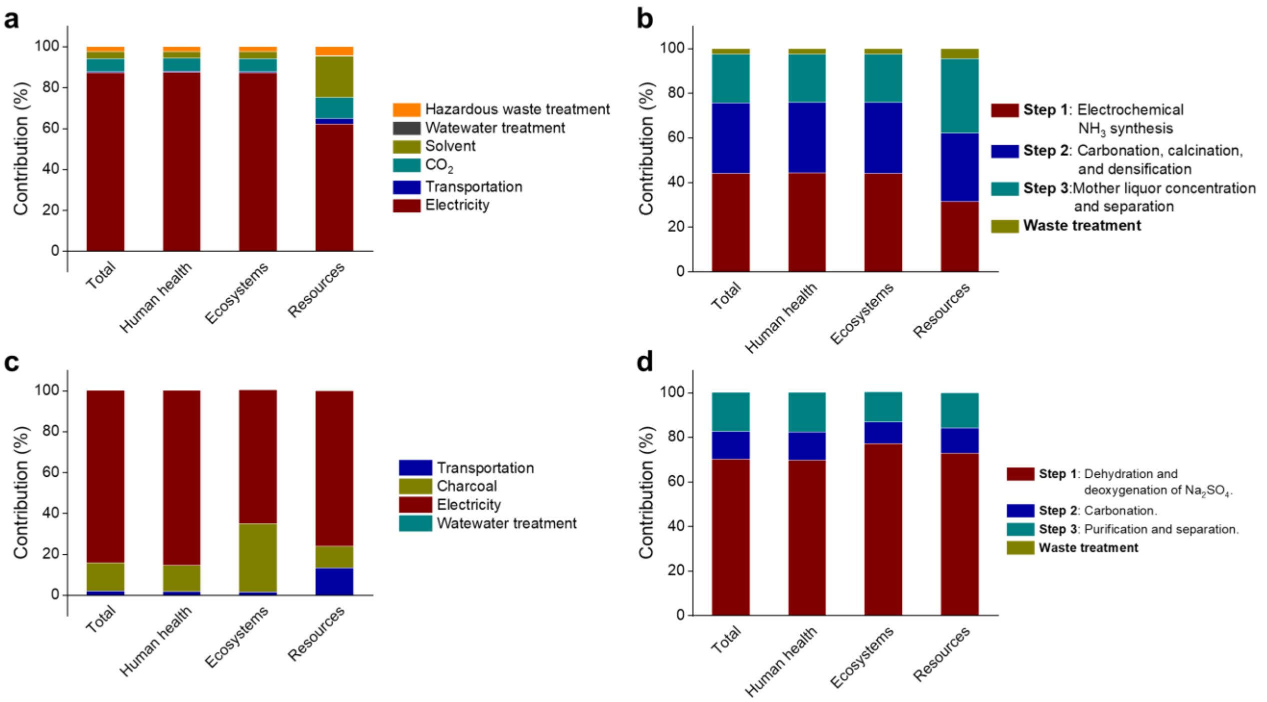

Supplementary Fig. 32 | Contribution analysis of end-point environmental impact categories. a-b, Contribution analysis for the New SSA-Process, broken down by specific inventory inputs (**a**) and unit processes/production steps (**b**). **c-d**, Contribution analysis for the proposed thermochemical route (this work), broken down by specific inventory inputs (**c**) and unit processes/production steps (**d**).

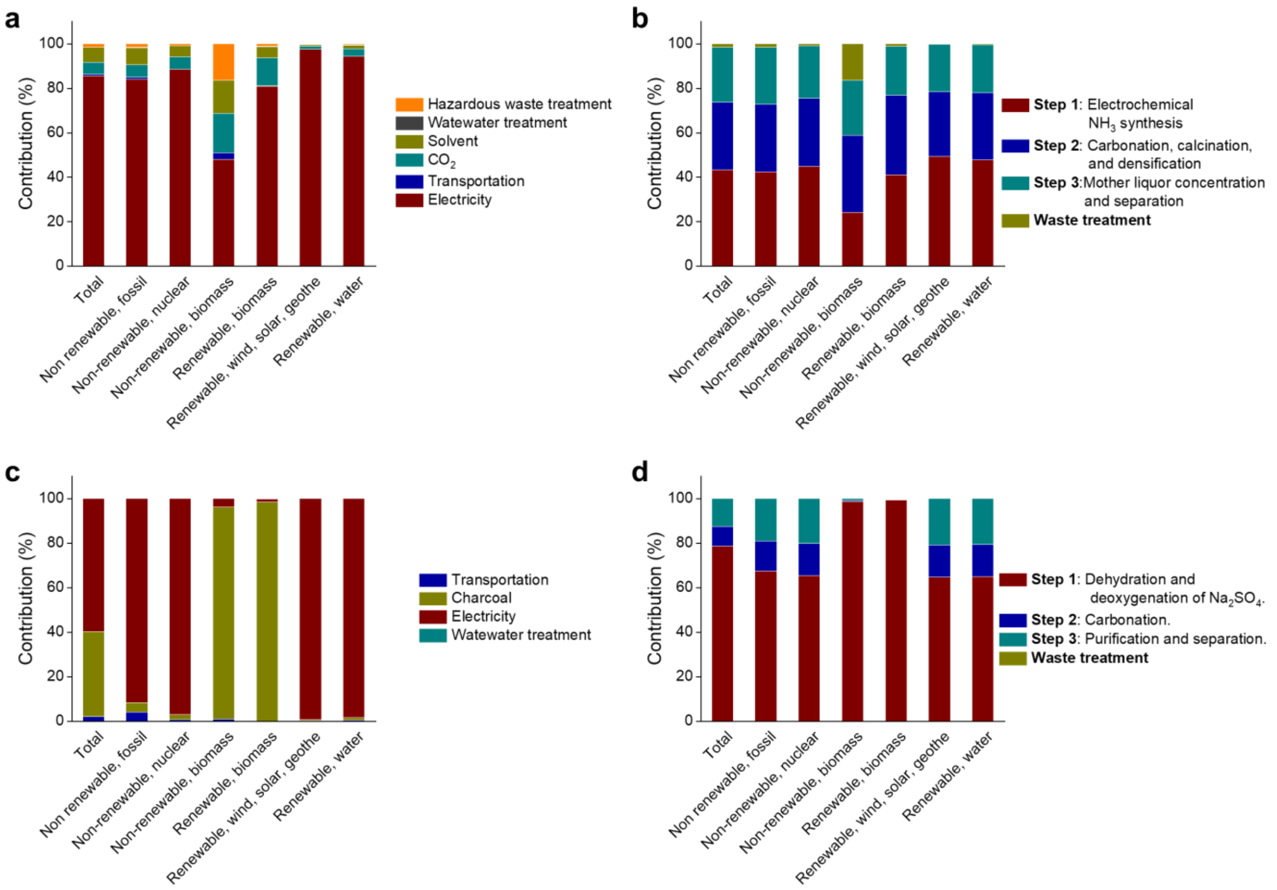

Supplementary Fig. 33 | Contribution analysis of CED. a-b, Contribution analysis for the New SSA-Process, broken down by specific inventory inputs (**a**) and unit processes/production steps (**b**). **c-d**, Contribution analysis for the proposed thermochemical route (this work), broken down by specific inventory inputs (**c**) and unit processes/production steps (**d**).

Justify Energy Demand: Discuss the high energy requirement (≥750 °C) of the proposed process and how it would be powered sustainably in a future industrial context to achieve a true carbon advantage.

Reply: We acknowledge the critical concern regarding the energy intensity of our process operating at or above 750 °C which undeniably implies a significant energy demand. However, we maintain that this demand can be sustainably met through direct industrial electrification utilizing renewable sources like wind and PV, industrial waste heat recovery, or via concentrated solar thermal integration, thereby facilitating efficient resource cycling within a future low-carbon landscape as illustrated in **Fig. R1-2**. Our reactor design is compatible with resistive electric heating and solar concentrators which effectively decouples the high-temperature requirement from carbon emissions by directly leveraging renewable electricity and solar heat. Crucially, while the operating temperature is substantial, our process eliminates the need for NH_3 infrastructure and bypasses the energy-intensive multi-step conversion chain inherent to green NH_3 synthesis (solar energy → electricity → green H_2 → green NH_3 → carbonation). In contrast, our approach follows a thermodynamically more direct pathway (solar or electrical energy → thermal energy → carbonation) (**Fig. R1-2**). Consequently, we have expanded the future outlook **Section** (in ***Scale-up production, LCA, and economic analysis***) and incorporated comparative LCA data in the **Revised Manuscript** and **Revised Supplementary Information** to quantitatively demonstrate the superior carbon reduction potential of this electrified thermal route.

6. Clarity and Context

The manuscript is clearly written and well-structured. However, the context provided is incomplete and outdated, as detailed above. The literature review should be expanded to include modern developments in sulfate valorization and green ammonia production.

Reply: We sincerely appreciate your positive feedback regarding the clarity and structure of our manuscript while fully acknowledging your valid critique that the initial context was incomplete. In strict accordance with your suggestions, we have comprehensively expanded the literature review in the revised **Introduction** to incorporate the most recent advancements in the field, specifically detailing the progress in MEA-MEG anti-solvent crystallization for sulfate valorization and the

emergence of PV-driven green NH_3 production. These updates provide a rigorous contemporary framework that allows us to better articulate the distinct position of our NH_3-free thermochemical route as a critical complementary strategy for processing impure waste streams where even modernized conventional processes face limitations. We appreciate your input and believe these revisions significantly improve the **Revised Manuscript**.

7. References

References are adequate for the experimental work but are lacking in the sections discussing industrial context and competing technologies. Key literature on advanced separation techniques and electrochemical NH_3 synthesis should be incorporated.

Reply: We sincerely appreciate your valuable comments. In response, we have comprehensively incorporated key literature regarding advanced separation technologies, specifically the MEA-MEG anti-solvent crystallization developed by Li et al. (*ACS Omega*, **2024**, 9(1): 1265-1277), as well as critical studies on green NH_3 synthesis into the **Revised Manuscript** (*Nature Reviews Chemistry*, **2025**, 9: 285-286) in accordance with your suggestions. Furthermore, to reinforce the breadth of the discussion, we have summarized recent literature concerning electrochemical NH_3 synthesis (**Supplementary Fig. 3** and **Table 3**). We believe these additions are essential for establishing the industrial context and accurately positioning our approach amidst a range of competing technologies, and we thank you once again for identifying these gaps which has allowed us to significantly improve the quality of our review.

Revise:

The Revised References

1. Vignes, A. Extractive metallurgy 2: metallurgical reaction processes. (John Wiley & Sons, 2013).
2. Caputo, H.E., Straub, J.E. & Grinstaff, M.W. Design, synthesis, and biomedical applications of synthetic sulphated polysaccharides. *Chem. Soc. Rev.* **48**, 2338-2365 (2019).
13. Fang, J. et al. Green recycling and regeneration of $LiNi_{0.5}Co_{0.2}Mn_{0.3}O_2$ from spent Lithium-ion batteries assisted by sodium sulfate electrolysis. *Chem. Eng. J.* **440**, 135880 (2022).
16. Li, B., Asselin, E. & Li, Z. New Process for Na_2CO_3 Production from Na_2SO_4 Based on Modeling the Na_2SO_4–$(NH_4)_2SO_4$–MEA–MEG–H_2O System. *ACS Omega* **9**, 1265-1277

(2024).
22. Cui, Z. et al. Durable Janus membrane with on-demand mode switching fabricated by femtosecond laser. *Nat. Commun.* **15**, 1443 (2024).
23. Zhu, J. et al. Bipolar membranes electrodialysis of lithium sulfate solutions from hydrometallurgical recycling of spent lithium-ion batteries. *Sep. Purif. Technol.* **354**, 128715 (2025).
31. Zhang, H., Lu, Z. & Wen, Z. Advancing lithium-mediated ammonia synthesis. *Nat. Rev. Chem.* **9**, 285-286 (2025).
40. Chen, L.-M. et al. Mechanism of Sodium Carbonate-Assisted Carbothermic Reduction of Titanomagnetite Concentrate. *Metall. Mater. Trans. B* **53**, 2272-2292 (2022).
42. Rumble, J. CRC handbook of chemistry and physics, Vol. 102. (CRC press Boca Raton, FL, 2017).
45. Abeynaike, A. & Barbenel, Y. Energy carrier exports from New Zealand to Japan – A comparative life cycle assessment of hydrogen and ammonia. *Int. J. Hydrogen Energy* **107**, 270-278 (2025).
46. International Renewable Energy Agency. Power generation costs. https://www.irena.org/.

8. Your Expertise

My expertise lies in industrial chemistry and process engineering. I am confident in assessing the experimental work and LCA. My comments on the agricultural market for ammonium sulfate are based on general scientific knowledge.

Reply: We would like to express our sincere gratitude for your rigorous assessment and constructive feedback throughout this review process. Your profound expertise in industrial chemistry and process engineering was clearly reflected in the depth of your technical comments, which have been instrumental in significantly elevating the rigor of our experimental validation and LCA. Moreover, we found your perspective on the agricultural market for $(NH_4)_2SO_4$ to be exceptionally insightful and accurate, serving as a critical guide for us to re-evaluate the strategic positioning of our technology within the broader industrial and environmental context. We are deeply grateful for the time and professional effort you have dedicated to helping us refine and improve our **Revised Manuscript**.

Reviewer #2

Comments:

The manuscript presents an innovative thermochemical process for upcycling waste Na_2SO_4 into Na_2CO_3 and elemental sulfur through carbothermal reduction and CO_2 carbonation. The proposed process demonstrates environmental advantages compared to the conventional SSA process. However, several aspects are insufficiently described, particularly within the LCA section, which requires substantial revision. The analysis also needs more detail for reproducibility. The following very critical issues should be addressed:

Reply: We sincerely appreciate the time and effort you have dedicated to reviewing our manuscript. We are particularly grateful for your encouraging comments regarding the innovation and environmental potential of our thermochemical upcycling process. We also highly value your constructive feedback regarding the LCA and experimental reproducibility. To address your concerns, we have performed a comprehensive revision of the manuscript, as detailed below:

1. **Updated benchmarks:** We have introduced an analysis of a "New SSA-Process" which incorporates green NH_3 synthesis and advanced separation systems. We conducted a comprehensive evaluation comparing this work against both the traditional SSA-Process and the New SSA-Process. Furthermore, we have added a radar chart in the **Revised Manuscript** (**Fig. R2-1**) to compare multidimensional performance metrics, including economic feasibility, recovery rate, NH_3 independence, low-carbon potential, energy-saving potential, and environmental sustainability.

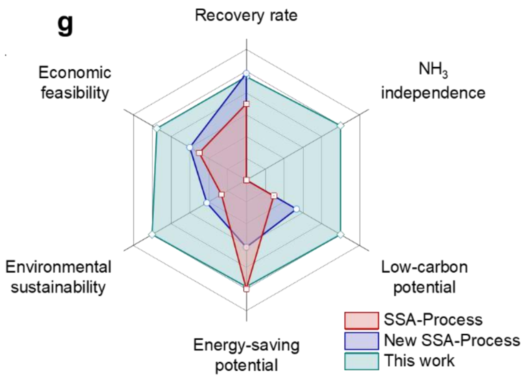

Fig. R2-1 | Radar chart comparing multidimensional performance metrics, including economic feasibility, recovery rate, NH_3 independence, low-carbon potential, energy-saving potential, and environmental sustainability.

2. **Economic feasibility analysis:** We have incorporated a life cycle cost (LCC) evaluation of relevant competing technologies into the **Revised Manuscript**. This analysis assesses technical feasibility under both current grid and future photovoltaic (PV) scenarios, thereby validating the environmental and economic advantages of our method within a broader context.
3. **Reproducibility and industrial application:** To further validate experimental reproducibility and industrial applicability, we expanded our testing beyond NaCl-containing desulfurization waste salts. We conducted additional validation using waste Na_2SO_4 containing Li^+ impurities and constructed a "Na_2CO_3-to-Na_2SO_4-to-Na_2CO_3" closed-loop system to simulate the Li_2CO_3 extraction industry (**Fig. R2-2**).

We believe these extensive revisions effectively address your concerns and significantly enhance the relevance and readability of the manuscript for a broader audience. Detailed responses to your specific comments are provided in the point-by-point response below.

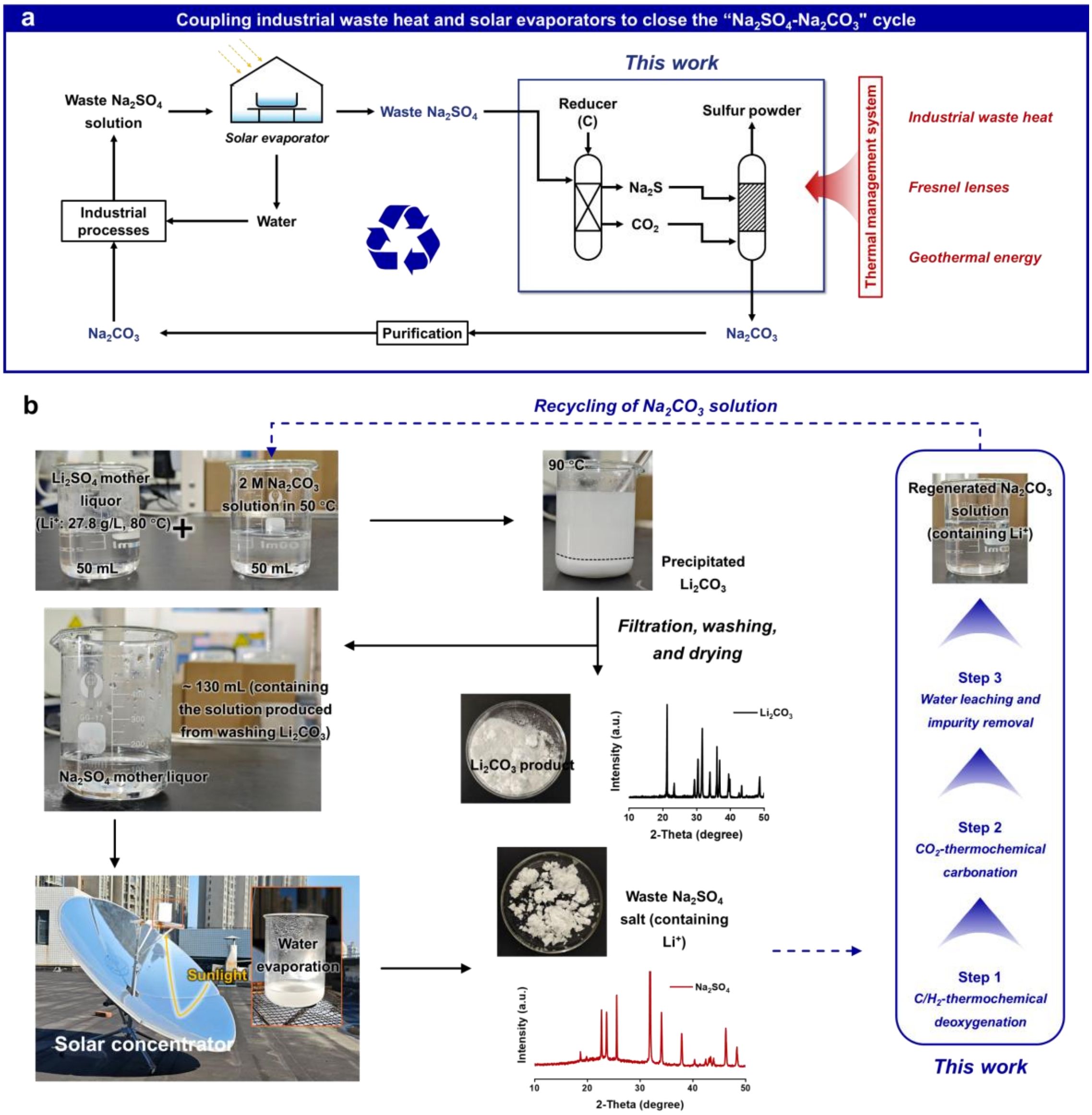

Fig. R2-2 | Strategies for closing the "Na_2SO_4-Na_2CO_3" loop via multi-energy integration. a, Schematic illustration of the synergistic coupling of industrial waste heat and solar evaporators to achieve a sustainable Na_2SO_4-Na_2CO_3 cycle. The system integrates solar thermal energy (e.g., Fresnel lenses) and geothermal energy to optimize thermal management. **b**, Practical demonstration of this cycle applied to lithium extraction. The process involves the precipitation of Li_2CO_3 followed by the concentration of the Na_2SO_4 mother liquor using a solar concentrator. The recovered Na_2SO_4 solid is then processed via the proposed thermochemical route (Steps 1-3) to regenerate the Na_2CO_3 solution for reuse.

1. The applied Na_2SO_4:C molar ratio of 1:4 is consistent with the overall stoichiometry shown in the main text. However, no evidence is provided regarding unreacted carbon or overall carbon balance. This information is essential to validate the completeness of the reduction and the reliability of the proposed stoichiometry.

Reply: We appreciate your valuable suggestion. We fully agree with your assessment that verifying the actual carbon consumption is essential for determining the content of unreacted carbon and validating the overall mass balance.

To address this, we conducted additional measurements of unreacted carbon at experimental temperatures ranging from 750 and 850 °C. We found that the actual carbon consumption ranges from 142% to 148% of the theoretical value, as shown in **Fig. R2-3**. This confirms that the applied Na_2SO_4:C molar ratio of 1:4 provides sufficient carbon for the deoxygenation of sodium sulfate. The observed deviation from theoretical stoichiometry arises because the reduction of Na_2SO_4 generates CO in addition to CO_2. Therefore, the use of excess carbon (double the theoretical amount based on the reaction $Na_2SO_4 + 2C = Na_2S + 2CO_2$) is scientifically justified. The residual carbon serves as a physical carrier for the Na_2S product, effectively preventing sintering and maintaining the necessary surface area for the subsequent reaction between Na_2S and CO_2.

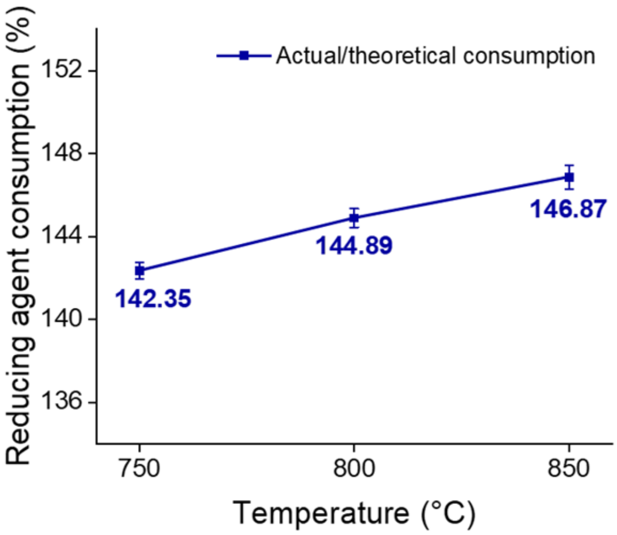

Fig. R2-3 | Temperature-dependent consumption of the reducing agent (C) during the carbothermal deoxygenation of Na_2SO_4.

Regarding the implications for the LCA, we clarify that our calculation model assumes a closed-loop system in which all generated CO is recovered and fully oxidized to CO_2 prior to its utilization in

carbonation or discharge (**Fig. R2-4**). This CO recovery strategy is readily implementable in industrial settings, ensuring maximum utilization of reducing agents (C and CO) while eliminating safety risks associated with CO emissions.

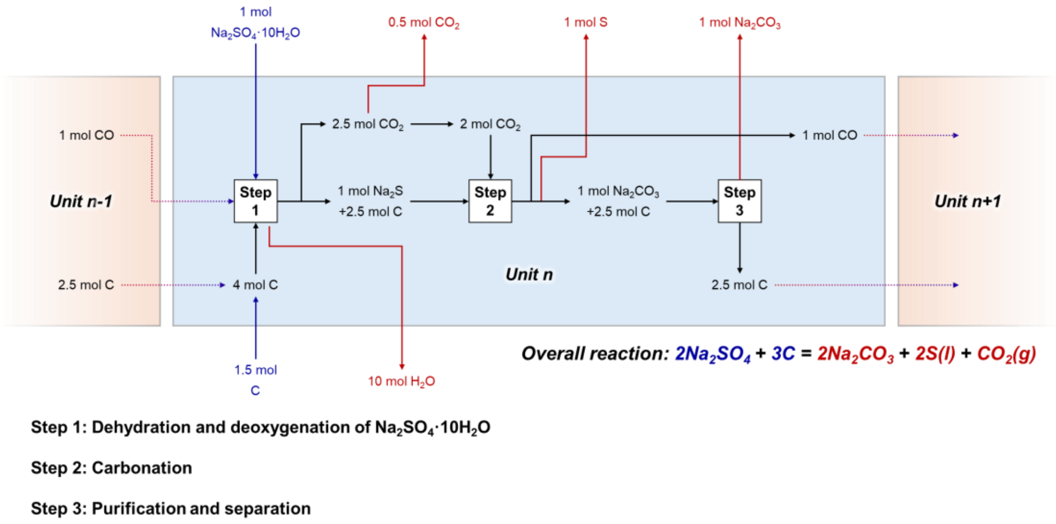

Fig. R2-4 | System boundary and process flow for the thermochemical conversion of waste Na_2SO_4 to Na_2CO_3 (this study). In the *n*-th cycle, both CO and C are assumed to undergo complete oxidation to CO_2 to maximize reductant utilization efficiency. Consequently, the carbon consumption in Unit *n* is calculated based on the stoichiometry of the overall reaction depicted in the figure.

To further clarify the carbon balance, we have explicitly distinguished between “utilizable” CO_2 (CO_2 1, Recycled flow to **Step 2**.) and “non-utilizable” CO_2 (CO_2 2, Direct emission to air.) in the inventory (**Table R2-1**). Importantly, the CO_2 emitted from the carbothermal reduction of charcoal is classified as biogenic carbon. This classification highlights a key environmental advantage of our process, as biogenic emissions are considered carbon-neutral in carbon footprint calculations.

We believe that these additional data and discussions satisfactorily address your query regarding carbon balance and significantly enhance the validity of our **Revised Manuscript** for a broader readership.

Table R2-1 | Life cycle inventory (LCI) for Na_2CO_3 production via thermochemical upcycling of waste Na_2SO_4 designed with a continuous recycling loop. Functional unit: production of 1 kg of dense Na_2CO_3. The detailed process flow is shown in **Supplementary Fig. 29**.

Inputs/outputs	Unit	Quantity	Comments
**Step 1: Dehydration and deoxygenation of Na_2SO_4**			
Inputs			

Waste $Na_2SO_4 \cdot 10H_2O$	kg	3.040	Calculated based on stoichiometry.
Transportation	tkm	0.304	Assumed transport distance of 100 km.
Electricity 1	kWh	0.526	Dehydration energy consumption, see **Supplementary Note 1** for details.
Charcoal	kg	0.453	Excess carbon source based on experimental data.
CO	kg	0.264	Recycled flow from **Step 2**.
Electricity 2	kWh	0.542	Heating energy consumption (750 °C, 4h), see **Supplementary Note 1** for details.
Outputs			
Na_2S	kg	0.736	Intermediate product for **Step 2**.
CO_2 1, biogenic	kg	0.830	Recycled flow to **Step 2**.
CO_2 2, biogenic	kg	0.208	Direct emission to air.
Charcoal (unreacted)	kg	0.283	Excess charcoal passed to **Step 2**.
Water	kg	1.700	Recovered via condensation.
**Step 2: Carbonation**			
Inputs			
Na_2S	kg	0.736	From **Step 1**.
CO_2	kg	0.830	From **Step 1**.
Charcoal	kg	0.283	Unreacted residue from Step 1 (inert in **Step 2**).
Electricity 3	kWh	0.242	Heating energy consumption (300 °C, 4h), see **Supplementary Note 1** for details.
Outputs			
Mixture (Na_2CO_3 + charcoal)	kg	1.283	Solid product mixture.
S	kg	0.302	Byproduct.
CO	kg	0.264	Recycled flow to **Step 1**.
**Step 3: Purification and separation**			
Inputs			
Mixture (Na_2CO_3 +	kg	1.283	From **Step 2**.

charcoal)			
Water (process)	kg	3.333	Solvent (based on solubility 0.45 kg/L with 1.5× safety factor).
Electricity 4	kWh	0.345	Energy for densification (0.9 GJ/t-Na_2CO_3)[22].
Outputs			
Na_2CO_3 (dense)	kg	1.000	Final product.
Charcoal	kg	0.283	Recycled flow to **Step 1**.
Water	kg	3.333	Recovered via evaporation/condensation.
**Overall**			
Inputs			
Waste $Na_2SO_4·10H_2O$	kg	3.040	Zero burden assumption.
Transportation	tkm	0.304	Uncertainty distribution: 1.50, 1.10, 1.00, 1.00, 2.00, 2.00; GSD: 2.90.
Charcoal	kg	0.170	Uncertainty distribution: 1.20, 1.10, 1.00, 1.00, 2.00, 1.05; GSD: 2.06.
Electricity (1-3)	kWh	1.311	Uncertainty distribution: 1.20, 1.10, 1.00, 1.00, 2.00, 1.05; GSD: 2.06.
Electricity 4	kWh	0.346	Uncertainty distribution: 1.20, 1.05, 1.50, 1.05, 1.05, 1.05; GSD: 1.58.
Outputs			
Na_2CO_3 (dense)	kg	1.000	Product (economic allocation: 84%).
S	kg	0.302	Byproduct (economic allocation: 16%).
CO_2 2, biogenic	kg	0.208	Uncertainty distribution: 1.20, 1.10, 1.00, 1.00, 2.00, 1.05; GSD: 2.06.
Water (Wastewater treatment)	kg	1.700	Net emissions; Uncertainty distribution: 1.20, 1.10, 1.00, 1.00, 2.00, 1.05; GSD: 2.06.

Note: Within the uncertainty distribution parameters, U_1, U_2, U_3, U_4, U_5, and U_b represent reliability, completeness, temporal correlation, geographical correlation, technological correlation, and the basic uncertainty factor, respectively.

Revise:

The Revised Results

C effectively reduced Na_2SO_4 to Na_2S without releasing SO_x, and the resulting Na_2S was porous, which is advantageous for the second step of the thermochemical reaction (i.e., the formation of Na_2CO_3). The CO_2/CO ratio in the effluent gas decreased with increasing temperature, indicating that the Boudouard reaction ($C + CO_2 \rightarrow 2CO$) becomes more prominent at higher temperatures and thus a lower carbon-utilization efficiency. Consistently, C consumption exceeded theoretical stoichiometry (142.35% – 146.87%) as temperature rose (**Supplementary Fig. 12**). Additionally, the feasibility of using hydrogen (H_2) as a reducing agent was confirmed above 750 °C (**Supplementary Fig. 13**). As green H_2 costs decline, this presents a viable, ultra-clean alternative for the valorization of waste Na_2SO_4.

The Revised Supplementary Information

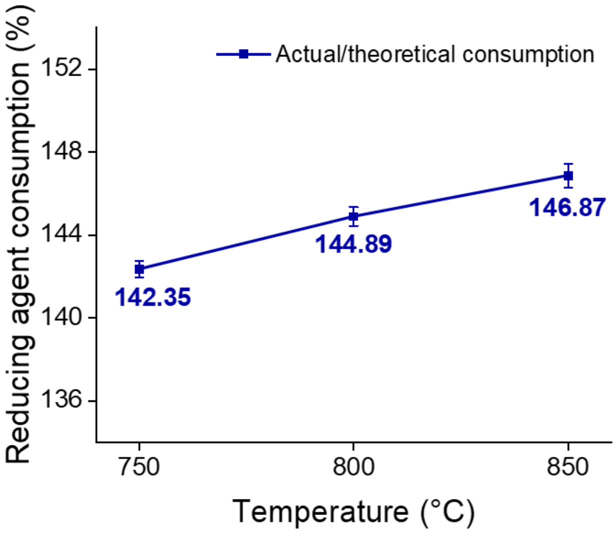

Supplementary Fig. 12 | Temperature-dependent consumption of the reducing agent (C) during the carbothermal deoxygenation of Na_2SO_4.

Supplementary Table 5 | Life cycle inventory (LCI) for Na_2CO_3 production via thermochemical upcycling of waste Na_2SO_4 designed with a continuous recycling loop. Functional unit: production of 1 kg of dense Na_2CO_3. The detailed process flow is shown in **Supplementary Fig. 29**.

Inputs/outputs	Unit	Quantity	Comments
**Step 1: Dehydration and deoxygenation of Na_2SO_4**			
Inputs			

Waste $Na_2SO_4·10H_2O$	kg	3.040	Calculated based on stoichiometry.
Transportation	tkm	0.304	Assumed transport distance of 100 km.
Electricity 1	kWh	0.526	Dehydration energy consumption, see **Supplementary Note 1** for details.
Charcoal	kg	0.453	Excess carbon source based on experimental data.
CO	kg	0.264	Recycled flow from **Step 2**.
Electricity 2	kWh	0.542	Heating energy consumption (750 °C, 4h), see **Supplementary Note 1** for details.
Outputs			
Na_2S	kg	0.736	Intermediate product for **Step 2**.
CO_2 1, biogenic	kg	0.830	Recycled flow to **Step 2**.
CO_2 2, biogenic	kg	0.208	Direct emission to air.
Charcoal (unreacted)	kg	0.283	Excess charcoal passed to **Step 2**.
Water	kg	1.700	Recovered via condensation.
**Step 2: Carbonation**			
Inputs			
Na_2S	kg	0.736	From **Step 1**.
CO_2	kg	0.830	From **Step 1**.
Charcoal	kg	0.283	Unreacted residue from Step 1 (inert in **Step 2**).
Electricity 3	kWh	0.242	Heating energy consumption (300 °C, 4h), see **Supplementary Note 1** for details.
Outputs			
Mixture (Na_2CO_3 + charcoal)	kg	1.283	Solid product mixture.
S	kg	0.302	Byproduct.
CO	kg	0.264	Recycled flow to **Step 1**.
**Step 3: Purification and separation**			
Inputs			
Mixture (Na_2CO_3 +	kg	1.283	From **Step 2**.

charcoal)			
Water (process)	kg	3.333	Solvent (based on solubility 0.45 kg/L with 1.5× safety factor).
Electricity 4	kWh	0.345	Energy for densification (0.9 GJ/t-Na_2CO_3)[22].
Outputs			
Na_2CO_3 (dense)	kg	1.000	Final product.
Charcoal	kg	0.283	Recycled flow to **Step 1**.
Water	kg	3.333	Recovered via evaporation/condensation.
**Overall**			
Inputs			
Waste $Na_2SO_4 \cdot 10H_2O$	kg	3.040	Zero burden assumption.
Transportation	tkm	0.304	Uncertainty distribution: 1.50, 1.10, 1.00, 1.00, 2.00, 2.00; GSD: 2.90.
Charcoal	kg	0.170	Uncertainty distribution: 1.20, 1.10, 1.00, 1.00, 2.00, 1.05; GSD: 2.06.
Electricity (1-3)	kWh	1.311	Uncertainty distribution: 1.20, 1.10, 1.00, 1.00, 2.00, 1.05; GSD: 2.06.
Electricity 4	kWh	0.346	Uncertainty distribution: 1.20, 1.05, 1.50, 1.05, 1.05, 1.05; GSD: 1.58.
Outputs			
Na_2CO_3 (dense)	kg	1.000	Product (economic allocation: 84%).
S	kg	0.302	Byproduct (economic allocation: 16%).
CO_2 2, biogenic	kg	0.208	Uncertainty distribution: 1.20, 1.10, 1.00, 1.00, 2.00, 1.05; GSD: 2.06.
Water (Wastewater treatment)	kg	1.700	Net emissions; Uncertainty distribution: 1.20, 1.10, 1.00, 1.00, 2.00, 1.05; GSD: 2.06.

Note: Within the uncertainty distribution parameters, U_1, U_2, U_3, U_4, U_5, and U_b represent reliability, completeness, temporal correlation, geographical correlation, technological correlation, and the basic uncertainty factor, respectively.

2. The Introduction and Methods sections indicate that both life cycle assessment (LCA) and life cycle cost (LCC) analyses were performed. However, no LCC results are provided anywhere in the manuscript or supplementary information. Why were the LCC results omitted, and what methodology was used to analyze them? This omission represents a major gap that raises serious concerns about the completeness of the study. The LCC results, or at minimum a clear methodological description and cost breakdown, must be included to substantiate the authors' claim of conducting both LCA and LCC analyses.

Reply: We sincerely appreciate your rigorous review and fully accept your criticism regarding the omission of the LCC results. We acknowledge that while our initial manuscript prioritized the mechanistic investigation and environmental impact assessment to maintain a focused narrative on the chemical innovation, the exclusion of the detailed economic data was a significant oversight that diminished the comprehensive scope of the study.

We agree that the economic viability is as critical as the environmental benefits. In response, we have now fully integrated the LCC analysis into the **Revised Manuscript** and **Revised Supplementary Information** to substantiate our claims.

1. Inclusion of LCC results: We have added a detailed economic assessment in the revised **Table 2** (**Table R2-2**) and **Supplementary Table 13**. This analysis explicitly compares the production costs of "This work" against the New SSA-Process and the BMED under both current grid and future photovoltaic energy structures.

Table R2-2 | Summary of economic analysis (fundamental unit: 1 ton of waste $Na_2SO_4 \cdot 10H_2O$).

Parameter	BMED	New SSA-Process	This work
**1. Costs (USD)**			
Electricity[a]	439.90 (189.16)	219.47 (94.37)	54.51 (23.44)
Other Materials (Reagents & Water)	13.53	22.78	24.01
Total Production Cost	453.43 (202.69)	242.25 (117.15)	78.52 (47.45)
**2. Revenue (USD)**			
Total Revenue	147.42	206.83	115.32
(Main Products)	NaOH, H_2SO_4	Na_2CO_3	Na_2CO_3

(Byproducts)	—	$(NH_4)_2SO_4$	S_x
**3. Profitability**			
Gross Profit (USD)	−306.01 (−55.27)	−35.42 (+89.68)	+36.80 (+67.87)
Profit Margin (%)	−207.58% (−37.49%)	−17.13% (+43.36%)	+31.91% (+58.85%)

Note: Values in parentheses (**a**) represent the scenario using renewable energy (PV) electricity costs (USD 0.043 per kWh[47]) instead of the standard grid electricity cost (USD 0.10 per kWh), For more detailed information, please refer to **Supplementary Table 13**.

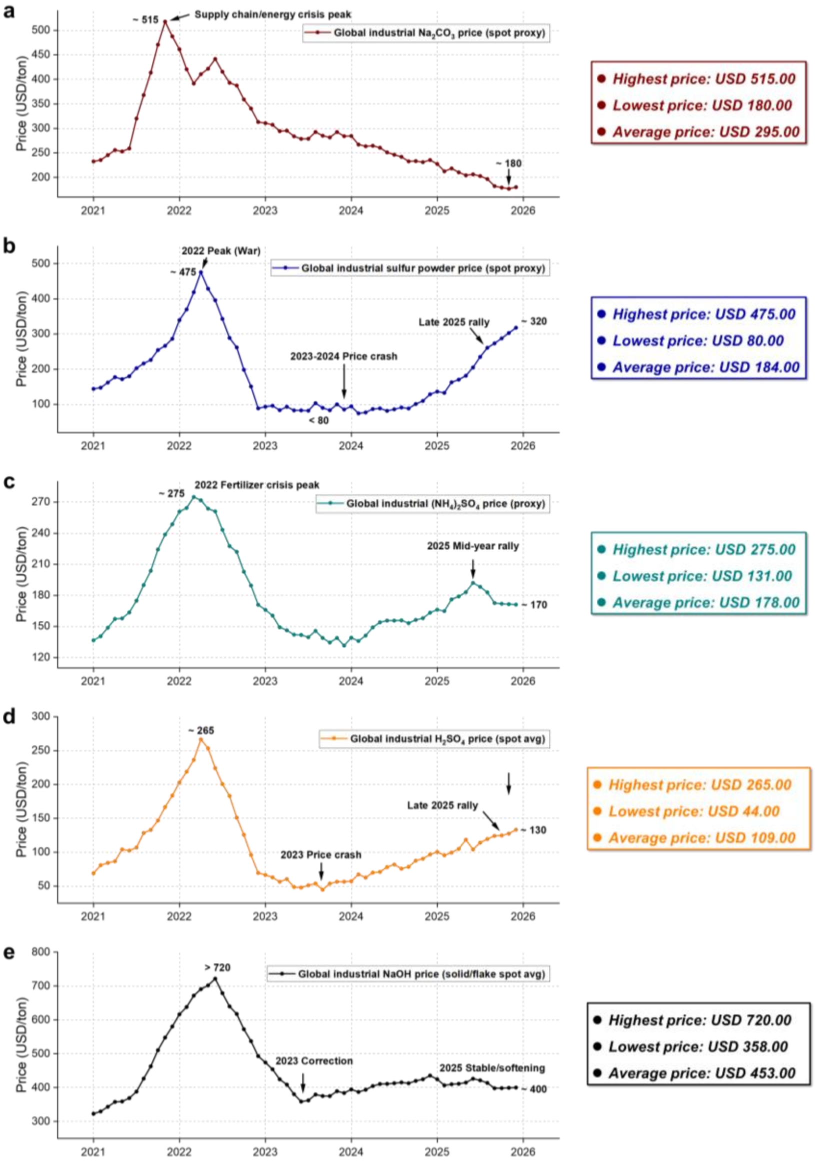

Fig. R2-5 | The global price trend of the relevant products in the past five years. **a**, Na_2CO_3. **b**, Sulfur powder. **c**, $(NH_4)_2SO_4$. **d**, H_2SO_4. **e**, NaOH.

2. Methodology Clarification: The revised LCC analysis is established based on a functional unit of recycling 1 ton of $Na_2SO_4{\cdot}10H_2O$. The model focuses on the differential values of electricity consumption, raw material inputs, and generated products. Capital expenditure regarding plant construction was excluded due to the complexity of normalizing construction costs across varying facility scales. To ensure accuracy, we aggregated 5-year historical market price data for key commodities (sodium carbonate, sulfur, sodium hydroxide, sulfuric acid, and ammonium sulfate) and utilized their average values for the assessment (**Fig. R2-5**).

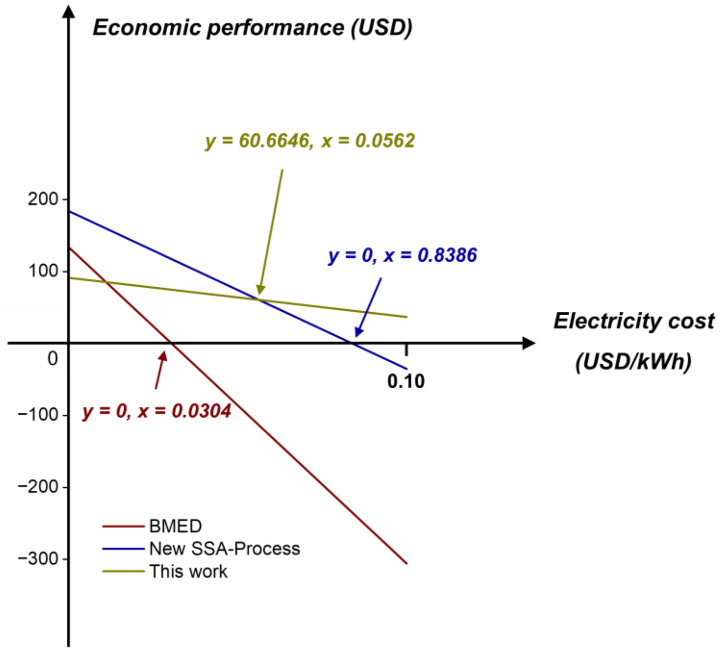

Fig. R2-6 | Sensitivity analysis of economic performance to electricity price fluctuations. The linear relationship between gross profit (USD) and electricity cost (USD/kWh) is compared for the BMED process (red), new SSA-Process (blue), and the proposed thermochemical route (this work, olive). The arrows indicate the break-even points (where profit equals zero) and the crossover threshold ($x \approx 0.056$ USD/kWh), beyond which the proposed route becomes economically superior to the new SSA-Process. The significantly flatter slope of the proposed route demonstrates its higher insensitivity against rising energy costs compared to the electricity-intensive BMED and new SSA-Processes.

3. Sensitivity analysis: Furthermore, we constructed a sensitivity analysis curve correlating profitability with electricity costs (**Fig. R2-6**). The results indicate that “This work” demonstrates superior economic robustness, exhibiting lower sensitivity to electricity price fluctuations compared to competing methods. In contrast, while the other technologies (New SSA-Process/BMED) show high

profit potential in future low-cost energy scenarios, they remain highly sensitive to electricity costs, presenting higher risks under current grid conditions.

We believe that the inclusion of this data fills the identified gap and provides the holistic assessment necessary to validate the proposed technology.

Revise:

The Revised Results

The economic evaluation was broadened to include BMED as a comparative benchmark via a detailed LCC analysis (**Supplementary Tables 12-13**). The proposed route demonstrates superior profitability, yielding +36.80 USD per ton of waste $Na_2SO_4·10H_2O$ (rising to +67.87 USD under PV-driven scenarios), representing a profit margin of 31.91% (58.85%) (**Table 2**). Crucially, sensitivity analysis reveals high economic robustness; our route becomes the most cost-effective option when electricity costs exceed $0.056 per kWh and shows minimal sensitivity to electricity price increases (**Supplementary Fig. 37**). Additionally, our process exhibits higher market stability because soda ash contributes 84% of total product revenue (**Supplementary Fig. 38**), whereas the New SSA-Process remains vulnerable to supply-demand fluctuations in the $(NH_4)_2SO_4$ market.

Table 2 | Summary of economic analysis (fundamental unit: 1 ton of waste $Na_2SO_4·10H_2O$).

Parameter	BMED	New SSA-Process	This work
**1. Costs (USD)**			
Electricity[a]	439.90 (189.16)	219.47 (94.37)	54.51 (23.44)
Other Materials (Reagents & Water)	13.53	22.78	24.01
Total Production Cost	453.43 (202.69)	242.25 (117.15)	78.52 (47.45)
**2. Revenue (USD)**			
Total Revenue	147.42	206.83	115.32
(Main Products)	NaOH, H_2SO_4	Na_2CO_3	Na_2CO_3
(Byproducts)	—	$(NH_4)_2SO_4$	S_x
**3. Profitability**			
Gross Profit (USD)	−306.01 (−55.27)	−35.42 (+89.68)	+36.80 (+67.87)

Profit Margin (%)	−207.58% (−37.49%)	−17.13% (+43.36%)	+31.91% (+58.85%)

Note: Values in parentheses (**a**) represent the scenario using renewable energy (PV) electricity costs (USD 0.043 per kWh[47]) instead of the standard grid electricity cost (USD 0.10 per kWh), For more detailed information, please refer to **Supplementary Table 13**.

The Revised Supplementary Information

Supplementary Table 13 | Summary of life cycle costs (LCC) for BMED (yielding H_2SO_4 and NaOH), the SSA-Process, the New SSA-Process, and this work. The assessment is based on a functional unit of processing 1,000 kg of waste $Na_2SO_4 \cdot 10H_2O$. Calculations utilize average market prices over the past five years (see **Supplementary Fig. 35** for historical price fluctuations of all products). Primary values are calculated based on coal-fired electricity generation, while values in parentheses represent a future PV-driven scenario.

**Parameter**	**BMED**	**New SSA-Process**	**This work**
**1. Material & Energy Costs (USD)**			
Feedstock			
Waste $Na_2SO_4 \cdot 10H_2O$	0.00[a]	0.00[a]	0.00[a]
Energy			
Electricity	439.90[b] (189.16)[b]	219.47[b] (94.37)[b]	54.51[b] (23.44)[b]
Chemical Reagents			
Charcoal (Reactant)	—	—	22.37[c]
NH_3	—	—	—
CO_2	—	4.78[d]	—
Process water/solvent	13.53[e]	18.00[f]	1.64[e]
**Total Costs (USD)**	**453.43 (202.69)**	**242.25 (117.15)**	**78.52 (47.45)**
**2. Revenue Streams (USD)**			
Main Product			
H_2SO_4	34.17[g]	—	—
NaOH	113.25[h]	—	—
Na_2CO_3	—	97.04	97.04

Byproducts			
$(NH_4)_2SO_4$	—	109.79[i]	—
S_x	—	—	18.28
**Total Revenue (USD)**	**147.42**	**206.83**	**115.32**
	**3. Economic Performance (USD)**		
**Gross Profit**	**−306.01 (−55.27)**	**−35.42 (+89.68)**	**+36.80 (+67.87)**
**Profit Margin (%)**	**−207.58% (−37.49%)**	**−17.13% (+43.36%)**	**+31.91% (+58.85%)**

a: Waste $Na_2SO_4 \cdot 10H_2O$ is assumed to be burden-free (0.00 USD) as it is a waste stream.

b: Baseline electricity cost is set at 0.10 $USD \cdot kWh^{-1}$. A sensitivity scenario considers a reduced rate of 0.043 $USD \cdot kWh^{-1}$, reflecting the global weighted average cost of utility-scale solar PV (Source: IRENA, https://www.irena.org/).

c: Charcoal (reductant) is priced at 400 $USD \cdot ton^{-1}$ based on prevailing market rates in China; future adoption of low-cost alternative carbon sources could further reduce expenses.

d: Industrial CO_2 is priced at 35 $USD \cdot ton^{-1}$, reflecting capture costs in the Chinese region (Source: CCUS and Global Power Generation Analysis)[29].

e: Process water is valued at 1.50 $USD \cdot ton^{-1}$.

f: Solvent costs are based on Chinese market prices: MEA at 1,000 $USD \cdot ton^{-1}$ and MEG at 500 $USD \cdot ton^{-1}$. A makeup rate of 5% per cycle is assumed to account for operational losses.

g: Product revenue for NaOH and H_2SO_4 is calculated on a 100% pure substance basis (e.g., the output of NaOH is equivalent to 250 kg of pure NaOH).

h: Same as note **g**; H_2SO_4 production is also calculated on a 100% basis.

i: The market value of $(NH_4)_2SO_4$ is estimated at 150% of the standard fertilizer-grade price. This premium reflects the higher purity and economic value achieved through the MEA-MEG purification process compared to conventional fertilizer products.

Limitations: The economic analysis is restricted to the variable operating expenditures (OPEX) associated with material and energy inputs. Fixed costs, including capital expenditures (CAPEX) for plant construction, equipment depreciation, and labor costs, are excluded from this assessment.

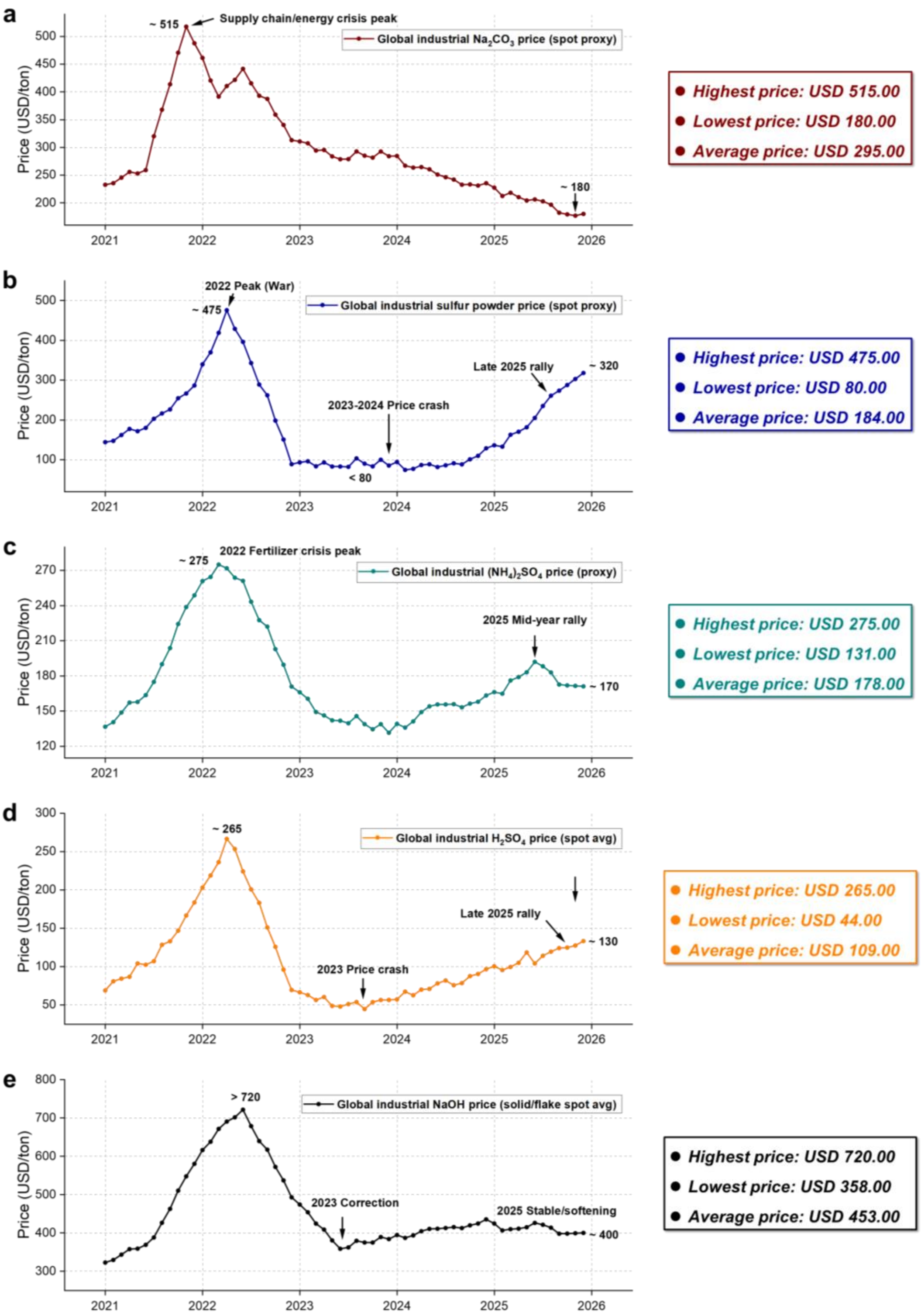

Supplementary Fig. 35 | The global price trend of the relevant products in the past five years. a, Na_2CO_3. **b**, Sulfur powder. **c**, $(NH_4)_2SO_4$. **d**, H_2SO_4. **e**, NaOH.

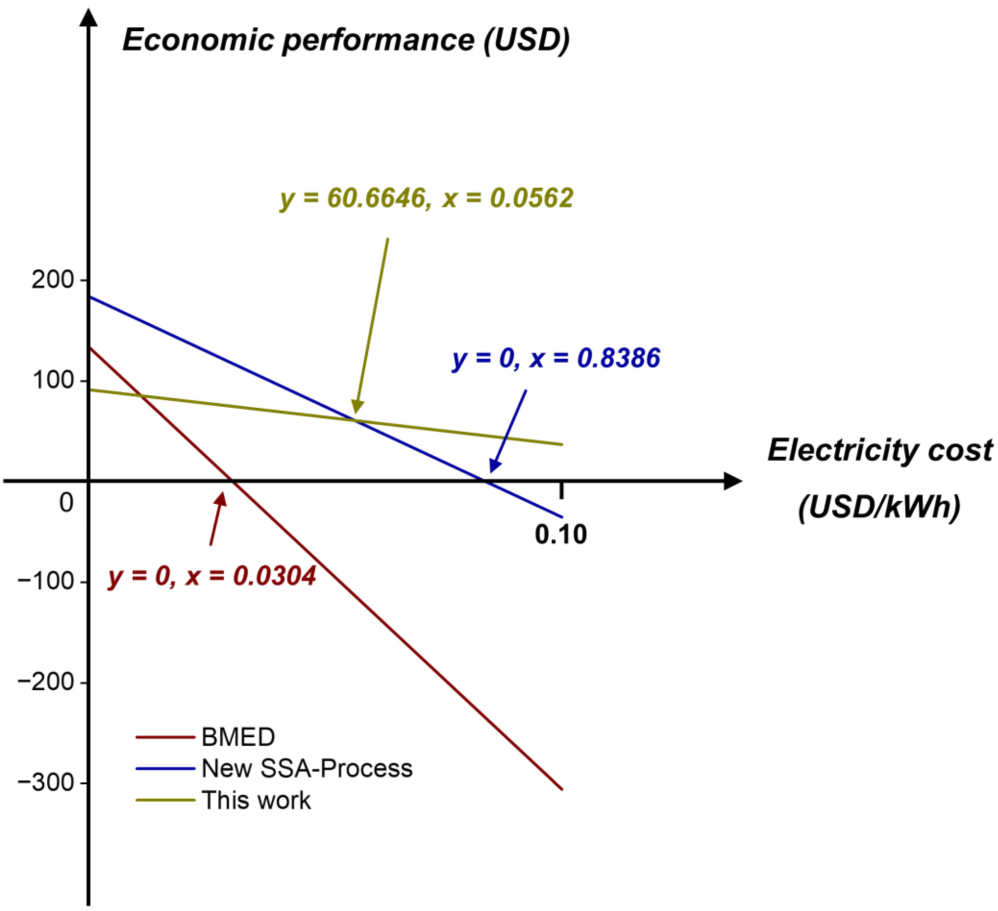

Supplementary Fig. 37 | Sensitivity analysis of economic performance to electricity price fluctuations. The linear relationship between gross profit (USD) and electricity cost (USD/kWh) is compared for the BMED process (red), new SSA-Process (blue), and the proposed thermochemical route (this work, olive). The arrows indicate the break-even points (where profit equals zero) and the crossover threshold (x ≈ 0.056 USD/kWh), beyond which the proposed route becomes economically superior to the new SSA-Process. The significantly flatter slope of the proposed route demonstrates its higher insensitivity against rising energy costs compared to the electricity-intensive BMED and new SSA-Processes.

3. Please complement the LCA breakdown by process step so readers can distinguish the contributions of dehydration, reduction, carbonation, and purification steps. Without this, identifying the dominant contributors remains difficult.

Reply: We sincerely appreciate your suggestion to provide a granular breakdown of the LCA results. We agree that distinguishing the contributions of individual process steps is crucial for identifying environmental hotspots.

To address this, we have supplemented the **Revised Supplementary Information** with a detailed contribution analysis for both the SSA-Process and This work (**Supplementary Figs. 31-33**). This assessment encompasses mid-point environmental impacts, end-point environmental impacts, and CED. The breakdown confirms that Step 1 is the dominant contributor to both environmental impact and energy demand.

However, we emphasize that the significant energy load in Step 1 is primarily driven by the dehydration process. As illustrated in **Fig. R2-2**, this specific operation possesses immense potential for optimization. In future industrial deployments, the dehydration step can be readily coupled with solar thermal energy technologies. This integration would substantially mitigate the energy footprint and further enhance the sustainability profile of the process.

Revise:

The Revised Supplementary Information

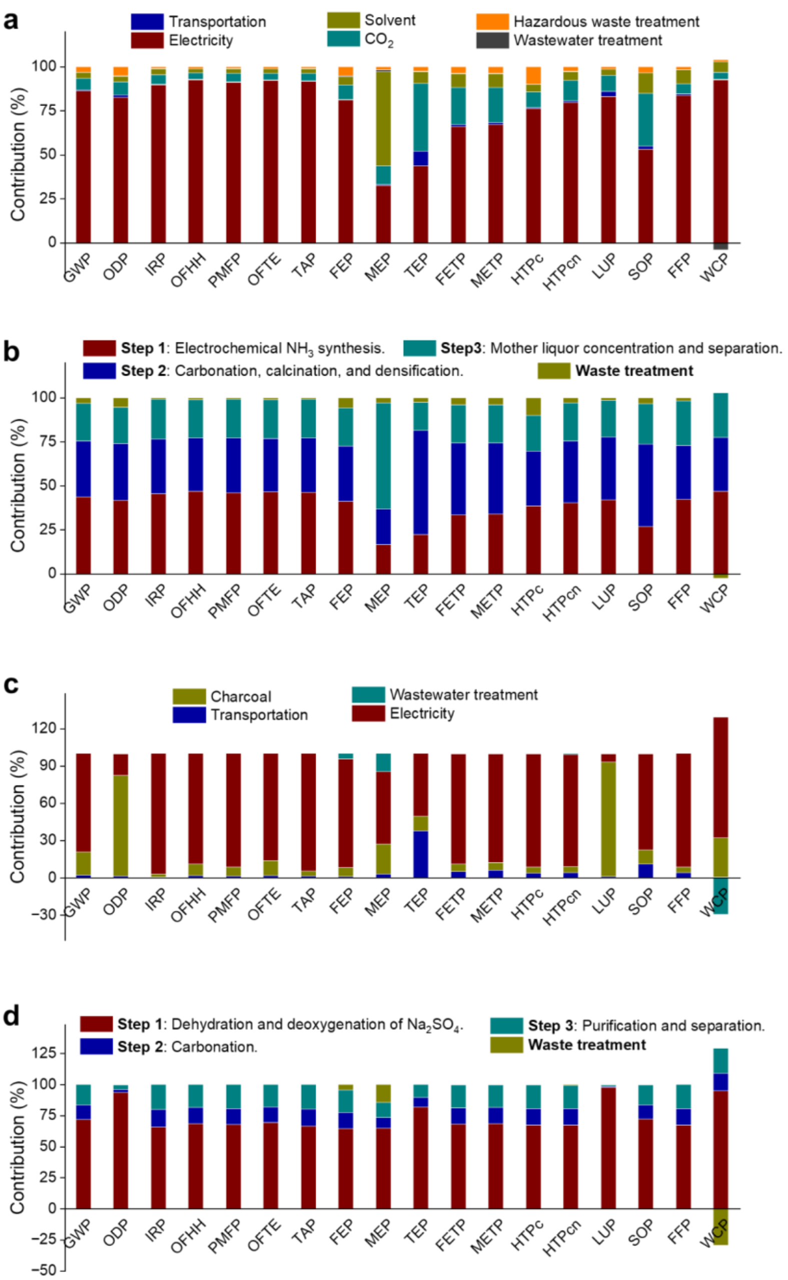

Supplementary Fig. 31 | Contribution analysis of mid-point environmental impact categories. a-b, Contribution analysis for the New SSA-Process, broken down by specific inventory inputs (**a**) and unit processes/production steps (**b**). **c-d**, Contribution analysis for the proposed thermochemical route (this work), broken down by specific inventory inputs (**c**) and unit processes/production steps (**d**).

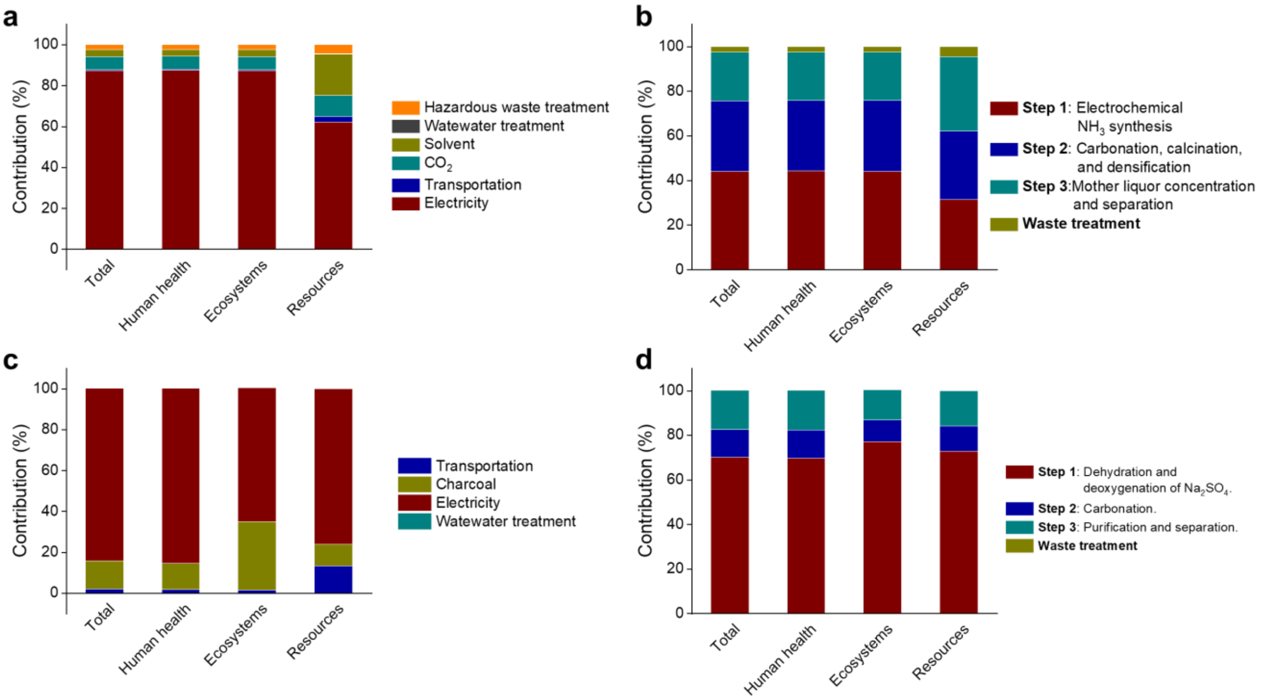

Supplementary Fig. 32 | Contribution analysis of end-point environmental impact categories. a-b, Contribution analysis for the New SSA-Process, broken down by specific inventory inputs (**a**) and unit processes/production steps (**b**). **c-d**, Contribution analysis for the proposed thermochemical route (this work), broken down by specific inventory inputs (**c**) and unit processes/production steps (**d**).

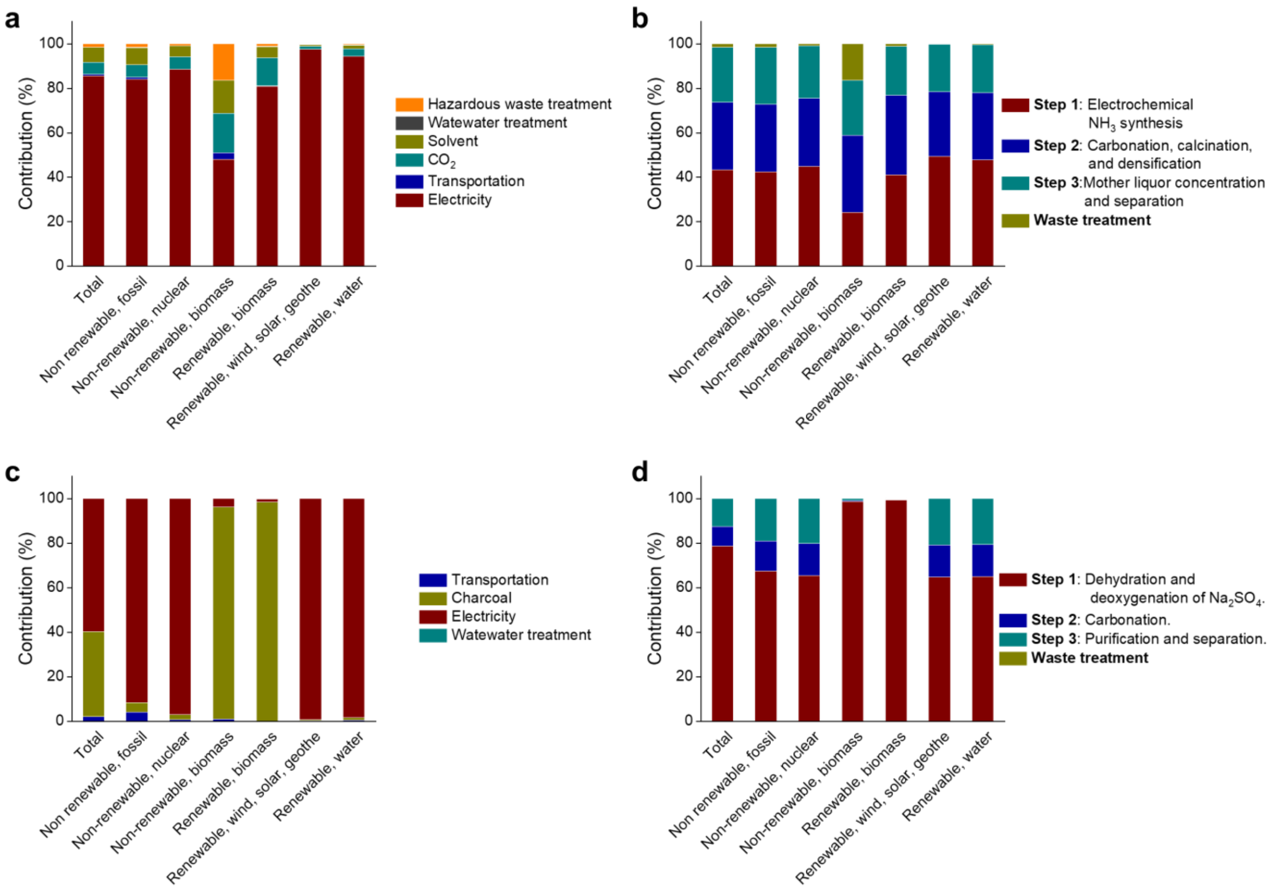

Supplementary Fig. 33 | Contribution analysis of CED. a-b, Contribution analysis for the New SSA-Process, broken down by specific inventory inputs (**a**) and unit processes/production steps (**b**). **c-d**, Contribution analysis for the proposed thermochemical route (this work), broken down by specific inventory inputs (**c**) and unit processes/production steps (**d**).

4. The LCA mainly reports total impact scores (mPt) and cumulative energy demand (CED), but the contribution of each product (Na_2CO_3 and S_x) to the impact categories is not explained. It should be clarified whether the observed environmental benefits originate primarily from Na_2CO_3 production or from the allocation to S_x as a byproduct.

Reply: We sincerely appreciate your question. We have clarified in the **Revised Manuscript** that the functional unit is defined as the production of 1 kg of dense Na_2CO_3. The reported end-point environmental impact scores and CED are based on this functional unit and represent the burdens specifically attributed to Na_2CO_3 following allocation.

Given the co-production of Na_2CO_3 and sulfur (S_x), this study employs economic allocation to partition the end-point environmental burden of the system. This method effectively separates the environmental burden share corresponding to co-products from the Na_2CO_3 results to ensure comparability across different process routes under the same functional unit.

We further clarify the rationale for exclusively adopting economic allocation in the revised version. System expansion requires defining substituted products, substitution ratios, and marginal supply scenarios. Since the types and applications of co-products vary across different process routes, these assumptions would significantly influence the results and reduce comparability between routes. In contrast, economic allocation does not rely on the selection of substituted products or marginal assumptions. Instead, it partitions the total system burden based solely on the economic value and output quantity of the co-products. This approach maintains methodological consistency even when co-product profiles differ. Based on these considerations, we have standardized the use of economic allocation as the default method in the revised manuscript and removed results related to system expansion to avoid excessive sensitivity to substitution assumptions.

It is important to emphasize that economic allocation only determines the attribution of environmental burdens among products and does not introduce substitution credits as seen in system expansion. Therefore, the observed differences in environmental performance primarily reflect variations in the total energy consumption and emission levels of each route, as well as the burden share attributed to Na_2CO_3 under economic allocation, rather than being driven by substitution credits from S_x.

The economic allocation coefficients were calculated based on the average market prices and

output quantities of each product within the same region over a five-year period (**Fig. R2-5**). The specific allocation coefficients for Na_2CO_3 and S_x are provided in **Table R2-1** (i.e., **Revised Supplementary Table 5**) and **R2-3** (i.e., **Revised Supplementary Table 7**). Should readers require the impact results attributed to S_x, these can be calculated by multiplying the total system environmental burden by the corresponding allocation coefficient for S_x, using the same calculation basis as for Na_2CO_3. We have provided further clarification on the allocation method and reporting basis in the **Supplementary Information** to facilitate reproducibility.

Table R2-3 | LCI data for Na_2CO_3 production via the new SSA-Process integrating electrochemical NH_3 synthesis and anti-solvent crystallization[24]. Functional unit: production of 1 kg of dense Na_2CO_3. System boundary: Cradle-to-gate.

Inputs/outputs	Unit	Quantity	Comments
**Step 1: Electrochemical NH_3 synthesis** (data sourced from **Supplementary Ref. 25**[25])			
Inputs			
Air	kg	0.333	Calculated based on stoichiometry.
Water	kg	0.528	Calculated based on stoichiometry.
Electricity 1	kWh	0.043	Energy for N_2 production (0.584 MJ/kg-N_2).
Electricity 2	kWh	3.229	Energy for H_2 production (55 kWh/kg-H_2).
Electricity 3	kWh	0.104	Energy for NH_3 synthesis (1.165 MJ/kg-NH_3).
Outputs			
NH_3	kg	0.320	Intermediate product for **Step 2**.
H_2	kg	0.002	Loss (3.32%), emission to air.
O_2	kg	0.540	Byproduct emission to air.
**Step 2: Carbonation, calcination, and densification**			
Inputs			
Waste $Na_2SO_4 \cdot 10H_2O$	kg	3.040	Stoichiometric requirement.
Waste $Na_2SO_4 \cdot 10H_2O$ (excess)	kg	1.173	Excess feed based on equilibrium data[24].
Transportation	tkm	0.304	Assumed transport distance of 100 km.
NH_3	kg	0.320	From **Step 1**.
CO_2	kg	0.830	Stoichiometric requirement.
Electricity 4	kWh	0.578	Used for pumps, compressors, and agitation (based on data from similar chemical processes; increased due to the addition of solvent systems)
Water	kg	3.589	Solvent (1.5× safety factor based on solubility of

			Na_2SO_4).
Electricity 5	kWh	1.064	Energy for $NaHCO_3$ decomposition[26].
Electricity 6	kWh	0.250	Energy for densification (0.9 GJ/t-Na_2CO_3)[22].
Outputs			
Na_2CO_3 (dense)	kg	1.000	Final product.
CO_2 (recycled)	kg	0.415	Recycled flow to **Step 2**.
Water	kg	0.170	Recovered via condensation.
Mother liquor (Na^+-NH_4^+-SO_4^{2-})	kg	7.367	Intermediate stream to **Step 3**.
**Step 3: Mother liquor concentration and separation**			
Inputs			
Mother liquor (Na^+-NH_4^+-SO_4^{2-})	kg	7.367	From **Step 2**.
Electricity 7	kWh	1.036	Energy for initial concentration, see **Supplementary Note 1** for details.
Solvent (MEA:MEG = 1:1)	kg	1.456	Anti-solvent for crystallization.
Electricity 8	kWh	0.369	Energy for post-separation solvent recovery, see **Supplementary Note 1** for details.
Outputs			
$(NH_4)_2SO_4$	kg	1.250	Byproduct.
Solvent	kg	1.383	Recycled flow to **Step 3**.
Solvent (hazardous waste treatment)	kg	0.073	Loss (5%), treated as hazardous waste[27].
Water	kg	4.944	Recovered via condensation.
Waste $Na_2SO_4 \cdot 10H_2O$	kg	1.173	Recycled flow to **Step 2**.
**Overall**			
Inputs			
Air	kg	0.333	Assumed burden-free.
Electricity (1-3)	kWh	3.375	Uncertainty distribution: 1.20, 1.10, 1.00, 1.05, 1.05, 1.05; GSD: 1.25.
Waste $Na_2SO_4 \cdot 10H_2O$	kg	3.040	Assumed burden-free.
Transportation	tkm	0.304	Uncertainty distribution: 1.50, 1.10, 1.00, 1.00, 2.00, 2.00; GSD: 2.90.
CO_2	kg	0.415	Uncertainty distribution: 1.20, 1.10, 1.00, 1.00, 2.00, 1.05; GSD: 2.06.
Electricity (4, 7, 8)	kWh	1.983	Uncertainty distribution: 1.20, 1.10, 1.00, 1.00, 2.00,

			1.05; GSD: 2.06.
Electricity (5, 6)	kWh	1.314	Uncertainty distribution: 1.20, 1.05, 1.50, 1.05, 1.05, 1.05; GSD: 1.58.
Solvent	kg	0.073	Uncertainty distribution: 1.20, 1.10, 1.50, 1.05, 2.00, 1.05; GSD: 2.30.
Outputs			
Na_2CO_3 (dense)	kg	1.000	Product (economic allocation: 57%).
$(NH_4)_2SO_4$	kg	1.250	Byproduct (economic allocation: 43%).
Water (wastewater treatment)	kg	0.997	Net emissions; Uncertainty distribution: 1.20, 1.10, 1.00, 1.00, 2.00, 1.05; GSD: 2.06.
Solvent (hazardous waste treatment)	kg	0.073	Uncertainty distribution: 1.20, 1.10, 1.50, 1.05, 2.00, 1.05; GSD: 2.30.
H_2		0.002	Uncertainty distribution: 1.20, 1.10, 1.00, 1.05, 1.05, 1.05; GSD: 1.25.
O_2	kg	0.540	Uncertainty distribution: 1.20, 1.10, 1.00, 1.05, 1.05, 1.05; GSD: 1.25.

5. Table S13 indicates that Monte Carlo simulations were performed, but the specific procedure is under described. Readers cannot reproduce the analysis without sufficient detail on how the simulations were conducted. Moreover, some impact categories exhibit extremely high coefficients of variation (water consumption: 4746%; human non-carcinogenic toxicity: 738%). Such high uncertainty could affect the reliability of the results and requires thorough discussion.

Reply: We sincerely appreciate this critical comment. We fully agree that a reproducible uncertainty analysis requires a detailed description of the procedural steps and parameter settings.

To address this, we have supplemented and refined the Monte Carlo simulation procedure in the **Revised Supplementary Information**, ensuring that readers can reproduce the analysis using the same parameters. Specifically, the methodology is detailed as follows:

1. Uncertainty assignment and determination of geometric standard deviation (GSD): We first assigned uncertainty distributions to each foreground inventory input and output. These assignments were based on the data quality indicator (DQI) evaluation matrix (**Table R2-4**) and the basic uncertainty factors (U_b) (**Table R2-5**). For each parameter, we determined specific scores for reliability (U_1), completeness (U_2), temporal correlation (U_3), geographical correlation (U_4), and technological correlation (U_5), alongside the basic uncertainty (U_b). These were synthesized into a geometric standard deviation (GSD) to serve as the comprehensive uncertainty coefficient, as calculated by **Equation 1** (detailed data provided in **Supplementary Table 5**, **Table 7**, and **Note 2**).

$$GSD = \exp\sqrt{\sum_{i=1}^{5}[\ln(U_i)]^2 + [\ln(U_b)]^2} \quad \textbf{(1)}$$

Where:

U_1: Uncertainty factor for reliability;

U_2: Uncertainty factor for completeness;

U_3: Uncertainty factor for temporal correlation;

U_4: Uncertainty factor for geographical correlation;

U_5: Uncertainty factor for technological correlation;

U_b: Basic uncertainty factor.

Table R2-4 | Data quality index evaluation matrix.

Indicator	1	2	3	4	5
Reliability (U_1)	Verified data based on measurements (1.00)	Verified data partly based on assumptions or non-verified data based on measurements (1.05)	Non-verified data partly based on qualified estimates (1.10)	Qualified estimate (1.20)	Non-qualified estimate (1.50)
Completeness (U_2)	Representative data from all sites relevant for the market considered, over an adequate period to even out normal fluctuation (1.00)	Representative data from >50% of the sites relevant for the market considered, over an adequate period to even out normal fluctuations (1.02)	Representative data from only some sites (<<50%) relevant for the market considered or >50% of sites but from shorter periods (1.05)	Representative data from only one site relevant for the market considered or some sites but from shorter periods (1.10)	Representativeness unknown or data from a small number of sites and from shorter periods (1.20)
Temporal correlation (U_3)	Less than 3 years of difference to the time period of the dataset (1.00)	Less than 6 years of difference to the time period of the dataset (1.03)	Less than 10 years of difference to the time period of the dataset (1.10)	Less than 15 years of difference to the time period of the dataset (1.20)	Age of data unknown or more than 15 years of difference to the time period of the dataset (1.50)
Geographical correlation (U_4)	Data from area under study (1.00)	Average data from larger area in which area under study is included (1.01)	Data from area with similar production conditions (1.02)	Data from area with slightly similar production conditions (1.05)	Data from unknown or distinctly different area (1.10)
Further technological correlation	Data from enterprises, processes and	Data from processes and materials	Data from processes and materials	Data on related	Data on related processes on laboratory scale

(U_5)	materials under study (1.00)	under study but from different enterprises (1.05)	under study but from different technologies (1.20)	processes or materials (1.50)	or from different technologies (2.00)

Table R2-5 | The basic uncertainty coefficients of input and output data (U_b).

Type	Input/output	Combustion emissions	Process emissions	Agricultural emissions
Demand input	Thermal energy, electricity, semi-finished products, working materials, and waste treatment	1.05	1.05	1.05
	Transport service	2.00	2.00	2.00
	Base installation	3.00	3.00	3.00
Resource input	Primary energy carriers, metals, and salts	1.05	1.05	1.05
	Land use (occupation)	1.50	1.50	1.50
	Land use (renovation)	2.00	2.00	2.00
Emission to air	CO_2	1.05	1.05	
	SO_2	1.05		
	NMVOC	1.50		
	NO_X、N_2O	1.50		1.40
	CH_4、NH_3	1.50		1.20
	Hydrocarbon monomer series	1.50	2.00	
	PM > 10	1.50	1.50	
	PM_{10}	2.00	2.00	
	$PM_{2.5}$	3.00	3.00	
	Polycyclic aromatic hydrocarbon	3.00		
	CO and heavy metals	5.00		
	Other inorganic emissions		1.50	
	Radionuclide		3.00	
Emission to water	BOD, COD, DOC, TOC, and inorganic compounds		1.50	
	Hydrocarbon monomer series and polycyclic aromatic hydrocarbon		3.00	
	Heavy metals		5.00	1.80
	Pesticides			1.50
	Phosphates and nitrates			1.50
Emission to soil	Petroleum		1.50	
	Heavy metals		1.50	1.50
	Pesticides			1.20

2. Distribution assumption: We adopted a log-normal uncertainty propagation model. This assumes that all foreground parameters follow a normal distribution in the ln-space. This approach ensures that sampled values for non-negative variables (e.g., energy consumption, mass flow) remain within a rational, positive range. This is the standard distribution assumption widely used in LCA uncertainty analyses.

3. Monte Carlo Simulation Settings and Output Metrics: We performed 1,000 iterations for the simulation. In each iteration, all inputs/outputs with assigned uncertainties were randomly sampled from their corresponding log-normal distributions. These sampled values were then entered into the LCI and LCA calculation chains to derive impact results for that specific iteration. We have reported the statistical metrics of the result distributions in the Supplementary Information, including the mean, median, standard deviation, coefficient of variation (CV), and the 95% confidence interval (2.5% and 97.5% quantiles) (**Supplementary Table 13**). To avoid sensitivity to extreme outliers, our interpretation of the results primarily focuses on the median and the 95% confidence interval.

Regarding the high coefficients of variation: We acknowledge your observation regarding the extremely high coefficients of variation in categories such as water consumption and human non-carcinogenic toxicity. We have added a clarification in the **Revised Supplementary Information** (**LCA methodology section**) to address this:

The high uncertainty in these categories stems primarily from the intrinsic characteristics of the LCIA methodology itself, rather than solely from measurement errors in the foreground inventory. In the ReCiPe method, characterization models for toxicity, land use, and water-related categories are subject to significant spatial heterogeneity and model structural uncertainty, a fact that is extensively documented in methodological literature [*The International Journal of Life Cycle Assessment*, **2017**, 22(2): 138-147; *The International Journal of Life Cycle Assessment*, **2016**, 21(11): 1543-1553]. We have observed similar patterns in our previous studies, where uncertainty in these categories consistently exceeds that of more robust metrics like Global Warming Potential [*Chemical Engineering Journal*, **2025**, 521: 166261].

For these reasons, we explicitly state in the revised manuscript that our primary conclusions do not rely on these high-uncertainty categories. Instead, we prioritize indicators with lower relative uncertainty that are directly relevant to the study's objectives, such as GWP and CED. Results for high-uncertainty categories are retained as reference ranges and are discussed in the context of

methodological limitations and future refinements, without over-interpretation.

Revise:

The Revised Supplementary Information

Supplementary Table 11 | Summary of uncertainty analysis for this work and new SSA-Process.

This Work							
**Impact category**	**Unit**	**Mean**	**Median**	**Standard deviation**	**Coefficient of variation (%)**	**2.5%**	**97.5%**
Fine particulate matter formation	kg PM2.5 eq	0.001775242	0.001675732	0.000585171	32.96288557	0.000999314	0.003209271
Fossil resource scarcity	kg oil eq	0.217136861	0.191810081	0.107886514	49.68595075	0.084100102	0.504769712
Freshwater ecotoxicity	kg 1,4-DCB	0.012419887	0.010531719	0.007129811	57.4064079	0.005053438	0.032331537
Freshwater eutrophication	kg P eq	0.000210321	0.000158946	0.00018036	85.75458576	5.52261E-05	0.000703499
Global warming	kg CO_2 eq	1.337897883	1.274854212	0.405614566	30.31730385	0.731105195	2.375251942
Human carcinogenic toxicity	kg 1,4-DCB	0.036912655	0.02570145	0.057708483	156.3379381	0.008807081	0.12814879
Human non-carcinogenic toxicity	kg 1,4-DCB	0.451661668	0.393372471	2.947275646	652.5405757	-5.690848254	5.976202888
Ionizing radiation	kBq Co-	0.053978666	0.025857867	0.101873103	188.7284559	0.004534926	0.271087168

	60 eq						
Land use	m^2a crop eq	0.170814609	0.145883123	0.148106692	86.70610387	-0.045481178	0.552686507
Marine ecotoxicity	kg 1,4-DCB	0.017322018	0.014671479	0.009975376	57.58783826	0.007133119	0.045306481
Marine eutrophication	kg N eq	2.22692E-05	2.08856E-05	7.20437E-06	32.3513514	1.2239E-05	3.98152E-05
Mineral resource scarcity	kg Cu eq	0.000607266	0.000576179	0.000202124	33.28425735	0.000327826	0.001046772
Ozone formation, Human health	kg NO_x eq	0.003256041	0.003027216	0.001208961	37.12979694	0.001605646	0.006059043
Ozone formation, Terrestrial ecosystems	kg NO_x eq	0.003374283	0.003162158	0.001222598	36.23282227	0.001674937	0.006221795
Stratospheric ozone depletion	kg CFC11 eq	8.80367E-07	7.86469E-07	4.24548E-07	48.22396644	3.64926E-07	1.96051E-06
Terrestrial acidification	kg SO_2 eq	0.003872895	0.00363282	0.001344282	34.71000549	0.002072475	0.007258994
Terrestrial ecotoxicity	kg 1,4-DCB	1.38563237	1.326125341	0.445166225	32.12729689	0.75650495	2.5104583
Water consumption	m^3	-0.003595564	0.09662842	0.661072398	-18385.77612	-1.584121626	1.057865119

Ecosystems	Pt	0.001811097	0.002086166	0.002531978	139.8035881	-0.004115449	0.006434786
Human health	Pt	0.032494291	0.032238819	0.022795419	70.15207329	-0.011502376	0.078820239
Resources	Pt	0.000296234	0.000269581	0.000119654	40.39161861	0.000143675	0.000614415
Total environmental impact	Pt	0.034601622	0.034884064	0.0251 0421	72.55211867	-0.014682335	0.0847 4588
Cumulative energy demand	MJ	20.23682481	18.82049913	7.167000508	35.41563747	10.54408364	37.61921961
**New SSA-Process**							
**Impact category**	**Unit**	**Mean**	**Median**	**Standard deviation**	**coefficient of variation (%)**	**2.5%**	**97.5%**
Fine particulate matter formation	kg PM2.5 eq	0.004918427	0.004731927	0.001176463	23.91949605	0.003247617	0.007639706
Fossil resource scarcity	kg oil eq	0.678421318	0.606853318	0.303577223	44.74759481	0.315670187	1.480967044
Freshwater ecotoxicity	kg 1,4-DCB	0.045899945	0.039620837	0.033022624	71.94480111	0.023140822	0.098468291
Freshwater eutrophication	kg P eq	0.000632152	0.0004 8202	0.0005 3215	84.18070602	0.000184431	0.002053076
Global warming	kg CO_2 eq	3.345430325	3.310326947	0.756597477	22.61584918	2.212641925	4.871328077
Human carcinogenic toxicity	kg 1,4-	0.114901966	0.093877025	0.129523061	112.7248426	0.025912865	0.330569119

	DCB						
Human non-carcinogenic toxicity	kg 1,4-DCB	1.869951249	1.547200412	18.58259153	993.7473796	-37.37500358	38.66155389
Ionizing radiation	kBq Co-60 eq	0.144139291	0.075814071	0.226445701	157.1019944	0.014777129	0.776387749
Land use	m^2a crop eq	0.040565071	0.034365342	0.026925053	66.37496863	0.015556362	0.103946984
Marine ecotoxicity	kg 1,4-DCB	0.062162533	0.053404462	0.04634849	74.56017048	0.031144951	0.133516954
Marine eutrophication	kg N eq	0.000111322	0.000103906	3.7972E-05	34.11008524	5.97262E-05	0.0002044
Mineral resource scarcity	kg Cu eq	0.002350007	0.002215684	0.000728755	31.01074582	0.00141532	0.004229172
Ozone formation, Human health	kg NO_x eq	0.00839914	0.008138465	0.00233378	27.78594584	0.004769613	0.013896219
Ozone formation, Terrestrial ecosystems	kg NO_x eq	0.008461651	0.008202219	0.002336305	27.61051037	0.004825531	0.01399991
Stratospheric ozone depletion	kg CFC11 eq	5.2889E-07	5.07613E-07	1.37507E-07	25.9991165	3.17075E-07	8.71345E-07
Terrestrial acidification	kg SO_2 eq	0.010823994	0.010510319	0.002451235	22.6463083	0.007067824	0.016470101

Terrestrial ecotoxicity	kg 1,4-DCB	4.332731612	4.066796799	1.491117832	34.41519036	2.431444722	7.305749643
Water consumption	m^3	0.036418583	0.241425633	1.784204063	4899.158433	-4.041824725	2.82858879
Ecosystems	Pt	0.003893505	0.005101071	0.006991043	179.556559	-0.01353962	0.014426948
Human health	Pt	0.088119309	0.092552806	0.078476661	89.05728151	-0.075528739	0.240041248
Resources	Pt	0.001017127	0.000978284	0.000294333	28.93771742	0.000603606	0.001709501
Total environmental impact	Pt	0.093029941	0.097374843	0.083515827	89.77306233	-0.089644288	0.251009425
Cumulative energy demand	MJ	37.904388	35.34560815	13.26990462	35.00888768	20.57201679	71.50459025

Note: The Monte Carlo simulation reveals relatively high coefficients of variation (CV) for water consumption and human non-carcinogenic toxicity in both This work and the SSA-Process. This elevated uncertainty stems primarily from the intrinsic characteristics of the LCA methodology (i.e., ReCiPe) rather than solely from measurement errors in the foreground inventory. As extensively documented in methodological literature, characterization models for toxicity, land use, and water-related categories are subject to significant spatial heterogeneity and model structural uncertainty[28, 29]. This pattern is consistent with our previous observations, where uncertainty in these categories consistently exceeds that of more robust metrics such as GWP[30]. Consequently, our primary conclusions prioritize indicators with lower relative uncertainty and direct relevance to the study's objectives (e.g., GWP and Cumulative Energy Demand). Results for high-uncertainty categories are retained as reference ranges and are acknowledged in the discussion regarding methodological limitations and future refinements.

Supplementary Note 2 | Life cycle assessment (LCA) methodology.

LCA was employed to evaluate the environmental impacts and cumulative energy demand of current mainstream waste Na_2SO_4 recycling processes compared to the method proposed in this study. The analysis followed the ISO 14040 and 14044 standards and was conducted using SimaPro software[35]. The functional unit was defined as the production of dense soda ash (Na_2CO_3). The system boundary includes inputs (energy and materials) and outputs (wastewater, waste gases, and solid waste) associated with the Na_2CO_3 production process, while the manufacturing and maintenance of capital equipment were excluded.

The LCI for the Na_2CO_3 production process developed in this study is detailed in **Supplementary Table 5**. To evaluate the potential of coupling advanced technologies, we integrated green NH_3 synthesis and an MEA-MEG-H_2O anti-solvent crystallization system into the standard Solvay process (SSA-Process)[24, 25]; this integrated route was evaluated as the “New SSA-Process” (**Supplementary Table 7**). For comparison, the LCI for the compared soda ash dense production process is based on the global production modeling of the improved Solvay process (Soda Ash process, i.e., SSA-Process in this study) in the Ecoinvent database[36].

The geographic location for electricity and fuel was set to China. Upstream background data for materials were sourced from the Ecoinvent 3.5 database, specifically data representing the Chinese (China, CN) market. When data for the CN region was unavailable, global average (GLO) or other world (ROW) production data were used as proxies. The electricity data for China within the Ecoinvent database was adjusted to reflect recent grid composition: coal-fired power (63.1%), hydropower (15.4%), wind power (8.7%), solar power (4.9%), nuclear power (4.8%), and natural gas power (3.1%). Additionally, the transportation distance for the raw material ($Na_2SO_4 \cdot 10H_2O$) was assumed to be 100 km, modeled using a truck meeting EURO 6 emission standards (>32 t).

Given the generation of byproducts (sulfur and distilled water) in the proposed process, economic allocation was applied to distribute environmental burdens and credits. This method assigns impacts in a multi-product system proportional to the economic value of the outputs. The five-year average market prices for dense Na_2CO_3, S_x powder, and industrial water were utilized: \$295/t, \$184/t, and \$1.5/t, respectively. Based on the production quantities presented in **Supplementary Table 5**, Na_2CO_3 accounts for 84% of the total environmental burden of the production system.

The ReCiPe 2016 method was utilized to assess environmental impacts, covering both midpoint and endpoint categories. Mid-point categories included global warming potential (GWP), stratospheric

ozone depletion (ODP), ionizing radiation (IRP), ozone formation, human health (OFHH), fine particulate matter formation (PMFP), terrestrial ecosystems (OFTE), terrestrial acidification potential (TAP), freshwater eutrophication (FEP), marine eutrophication (MEP), terrestrial ecotoxicity (TEP), freshwater ecotoxicity (FETP), marine ecotoxicity (METP), human carcinogenic toxicity (HTPc), human non-carcinogenic toxicity (HTPcn), land use potential (LUP), mineral resource scarcity (SOP), fossil resource scarcity (FFP), and water consumption (WCP). End-point categories include human health, ecosystems, and resources. Mid-point characterization results and normalized environmental impacts for the Na_2CO_3 production process are provided in **Supplementary Table 8** and **Supplementary Fig. 32**, respectively (values in parentheses denote the scenario utilizing a PV-driven electricity background). End-point environmental impact categories and cumulative energy demand are presented in **Supplementary Tables 9-10**. **Supplementary Figs. 33-35** provide a contribution analysis of environmental impacts under different allocation methods.

To rigorously evaluate the propagation of uncertainty from both foreground inventories and background databases, a Monte Carlo simulation was performed with 1,000 iterations. Uncertainty distributions for inventory inputs and outputs were quantified using the Pedigree Matrix approach. The reliability (U_1), completeness (U_2), temporal correlation (U_3), geographical correlation (U_4), and technological correlation (U_5) were determined for each parameter along with a basic uncertainty factor (U_b). These were synthesized into a geometric standard deviation (GSD) representing the comprehensive uncertainty coefficient, as shown in **Equation 1** (data detailed in **Supplementary Tables 5** and **7**):

$$\mathrm{GSD} = \exp\sqrt{\sum_{i=1}^{5}[\ln(U_i)]^2 + [\ln(U_b)]^2} \quad \textbf{(1)}$$

Where:

U_1: Uncertainty factor for reliability;

U_2: Uncertainty factor for completeness;

U_3: Uncertainty factor for temporal correlation;

U_4: Uncertainty factor for geographical correlation;

U_5: Uncertainty factor for technological correlation;

U_b: Basic uncertainty factor.

Supplementary Table 11 summarizes the uncertainty analysis results for all environmental impact indicators, reporting the probabilistic mean, standard deviation, coefficient of variation, and the 95% confidence interval range. Finally, sensitivity analyses were performed. First, the environmental impact of the three processes under a PV-driven scenario was evaluated to quantify emission reduction potentials (**Supplementary Fig. 36**). Second, the robustness of the economic allocation method was verified by assessing the impact of varying soda ash prices (high, average, and low scenarios) (**Supplementary Fig. 38**). The results indicate that while price fluctuations influence the magnitude of environmental impacts, the relative ranking of the technological routes and the study's key conclusions remain unchanged, confirming the reliability of the economic allocation approach used.

6. The study applies economic allocation to distribute environmental burdens among co-products, but no sensitivity analysis is provided. Given the substantial volatility of product prices in recent years, the allocation results presented here cannot be considered reliable. The authors should re-evaluate the LCA outcomes by incorporating the recent variations in product price or, at minimum, perform a quantitative sensitivity analysis to verify the robustness of their conclusions.

Reply: We sincerely appreciate you pointing out the issue regarding the robustness of economic allocation. We agree with you that product price volatility influences allocation coefficients and the resulting burden partitioning.

To mitigate the impact of short-term price anomalies on our conclusions, we calculated the default allocation coefficients using average market prices from the same region over a five-year period (**Fig. R2-5**). Based on this dataset, we conducted a quantitative sensitivity analysis to address your concern.

1. Methodology: We recalculated the Na_2CO_3 allocation coefficients and corresponding LCA results under three distinct scenarios: low-price, average-price, and high-price (**Fig. R2-7**).

The low and high scenarios correspond to the minimum and maximum annual average prices observed over the past five years, respectively (**Fig. R2-5**).

The Average scenario utilizes the five-year mean.

Data sources and statistical methods are detailed in the Supplementary Information, with summarized price trends provided in **Fig. R2-5** (i.e., **Supplementary Fig. 35**).

2. Sensitivity results: The results indicate that the allocation coefficient for Na_2CO_3 varies between 76% and 90%, with a default value of 84% (**Fig. R2-7**).

This work: The corresponding total endpoint score (mPt) ranges from 31.39 to 37.17 (default: 34.69). This represents a variation of −9.5% to +7.1% relative to the default scenario.

New SSA-Process: The total endpoint score ranges from 72.41 to 112.63 (default: 91.71). This represents a variation of −21.1% to +22.8% relative to the default scenario.

We have added these detailed results to **Supplementary Fig. 35** in the **Revised Manuscript** and included corresponding discussions in both the main text and **Revised Supplementary Information** to verify the robustness of our findings as you suggested.

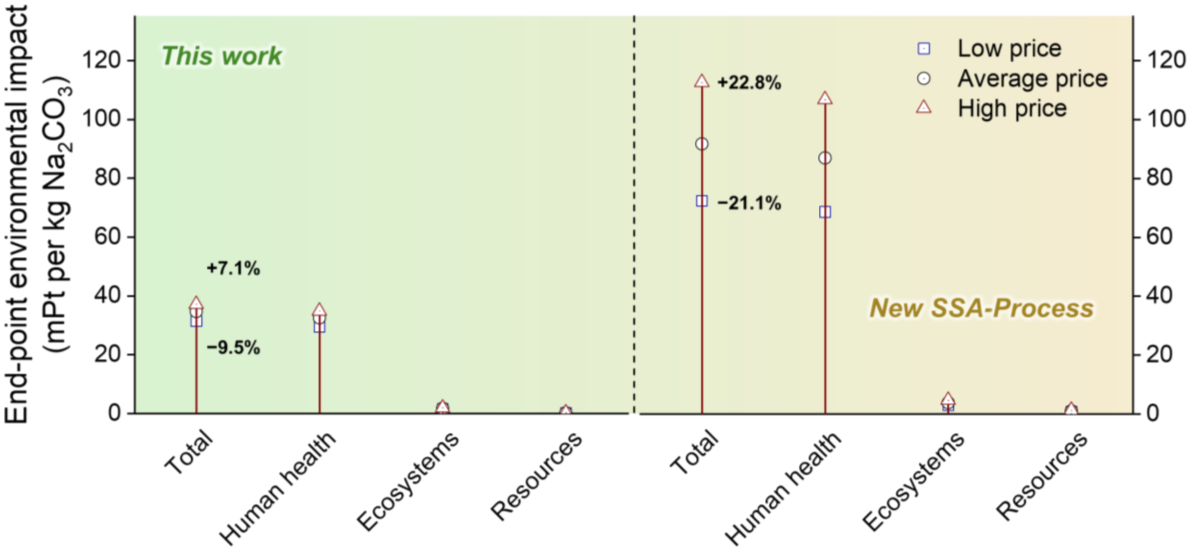

This work

Damage category	Low price	Average price	High price
Total	31.39	34.69	37.17
Human health	29.45348147	32.55384782	34.87912279
Ecosystems	1.660802689	1.835624023	1.966740027
Resources	0.272228208	0.300883806	0.322375509

Note: The distribution coefficients of Na_2CO_3 products under the lowest, average and highest prices are 76%, 84%, and 90% respectively.

New SSA-Process

Damage category	Low price	Average price	High price
Total	72.41	91.71	112.63
Human health	68.64450725	86.94970921	106.7803446
Ecosystems	2.969154746	3.760929346	4.618685161
Resources	0.791411555	1.002454636	1.231084641

Note: The distribution coefficients of Na_2CO_3 products under the lowest, average and highest prices are 45%, 57%, and 70%respectively.

Fig. R2-7 | Impact of Na_2CO_3 price volatility on end-point environmental performance. Comparison of endpoint environmental impacts between the proposed thermochemical route (this work) and the New SSA-Process under three price scenarios: low (blue squares), average (black circles), and high (red triangles). The percentages indicate the deviation from the baseline (average price) scenario.

Revise:

The Revised Results

The economic evaluation was broadened to include BMED as a comparative benchmark via a detailed LCC analysis (**Supplementary Tables 12-13**). The proposed route demonstrates superior profitability, yielding +36.80 USD per ton of waste $Na_2SO_4 \cdot 10H_2O$ (rising to +67.87 USD under PV-driven scenarios), representing a profit margin of 31.91% (58.85%) (**Table 2**). Crucially, sensitivity

analysis reveals high economic robustness; our route becomes the most cost-effective option when electricity costs exceed $0.056 per kWh and shows minimal sensitivity to electricity price increases (**Supplementary Fig. 37**). Additionally, our process exhibits higher market stability because soda ash contributes 84% of total product revenue (**Supplementary Fig. 38**), whereas the New SSA-Process remains vulnerable to supply-demand fluctuations in the $(NH_4)_2SO_4$ market.

The Revised Supplementary Information

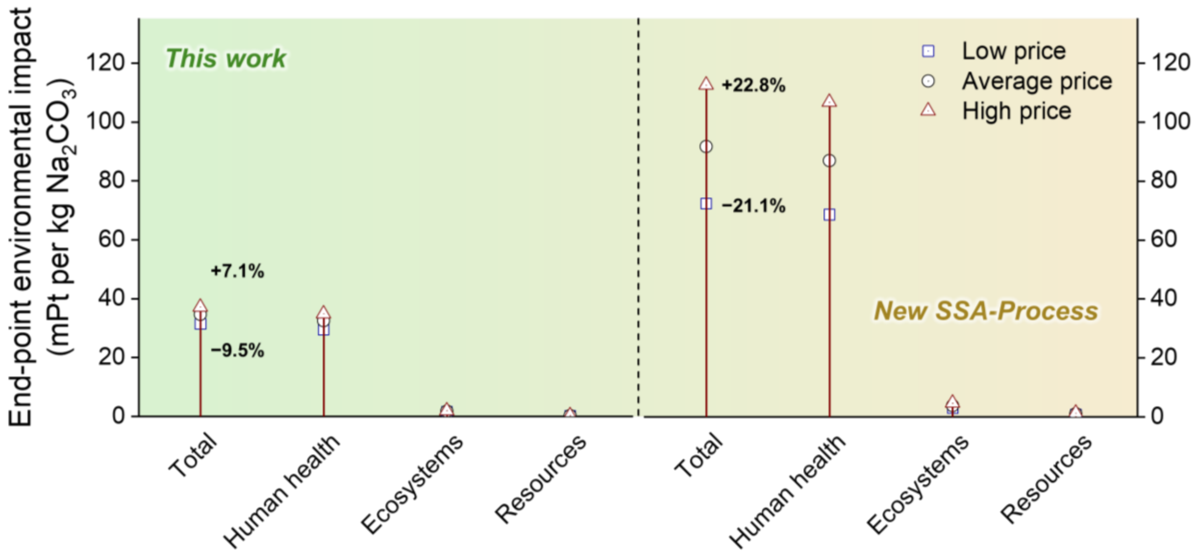

This work

Damage category	Low price	Average price	High price
Total	31.39	34.69	37.17
Human health	29.45348147	32.55384782	34.87912279
Ecosystems	1.660802689	1.835624023	1.966740027
Resources	0.272228208	0.300883806	0.322375509

Note: The distribution coefficients of Na_2CO_3 products under the lowest, average and highest prices are 76%, 84%, and 90% respectively.

New SSA-Process

Damage category	Low price	Average price	High price
Total	72.41	91.71	112.63
Human health	68.64450725	86.94970921	106.7803446
Ecosystems	2.969154746	3.760929346	4.618685161
Resources	0.791411555	1.002454636	1.231084641

Note: The distribution coefficients of Na_2CO_3 products under the lowest, average and highest prices are 45%, 57%, and 70%respectively.

Supplementary Fig. 38 | Impact of Na_2CO_3 price volatility on end-point environmental performance. Comparison of endpoint environmental impacts between the proposed thermochemical route (this work) and the New SSA-Process under three price scenarios: low (blue squares), average (black circles), and high (red triangles). The percentages indicate the deviation from the baseline (average price) scenario.

7. To enhance the reproducibility of the thermodynamic analysis, it would be helpful to include a more detailed description of the input parameters and model settings used in the FactSage and HSC calculations. While general assumptions (e.g., activities = 1, standard pressure) and reaction equations are provided, clarifying other relevant settings or input data would improve transparency.

Reply: Thank you for raising this important issue. We have carefully considered your suggestions and made corresponding improvements. Specifically, we have provided additional details regarding the input parameters. For the FactSage calculations, we have included the molar ratios of the input species as well as the concentration threshold values for all possible products (> 10^{-5} mol). This means that products with concentrations below this threshold are not considered in the reactions. For the HSC calculations, our inputs were based on standard pressure and an activity of 1, as we were unable to obtain CO_2 data for our closed reactor system under 5 atm conditions within the model. On the other hand, our experimental work in the continuous reactor process was conducted at 1 atm, which effectively ensures the reproducibility of the thermodynamic analysis. Additionally, we have provided more detailed settings used in the HSC calculations (e.g., “Show Transitions” and “Criss-Cobble”). Lastly, we appreciate your feedback on this matter, and all revisions made have been clearly marked in the revised manuscript and supplementary information.

Revise:

The Revised Results

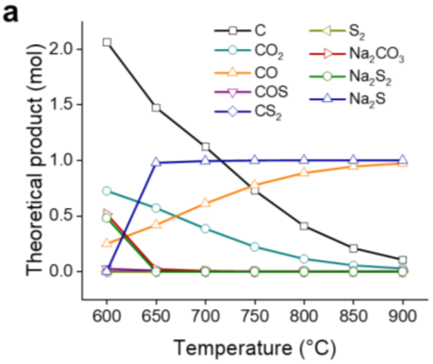

Fig. 2 | Thermochemical deoxygenation behavior of Na_2SO_4. a, Thermodynamic equilibrium analysis of the Na_2SO_4-C system (molar ratio: 1:4, reaction pressure: 1 atm, concentration cut-off: > 10^{-5} mol, and activity: 1) derived from FactSage 8.3 calculations, predicting product distribution and phase stability across the temperature range.

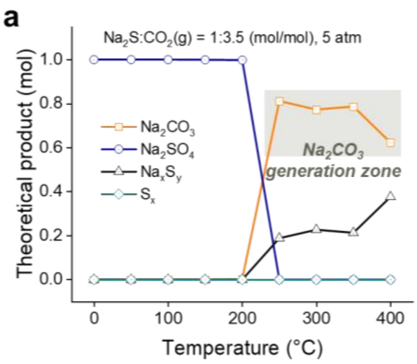

Fig. 3 | Thermochemical carbonation of Na_2S in a CO_2 atmosphere. a, Thermodynamic equilibrium analysis of the Na_2S-CO_2 system (molar ratio: 1:3.5, reaction pressure: 5 atm, concentration cut-off: > 10^{-5} mol, activity: 1), predicting the temperature window for Na_2CO_3 generation.

The Revised Supplementary Information

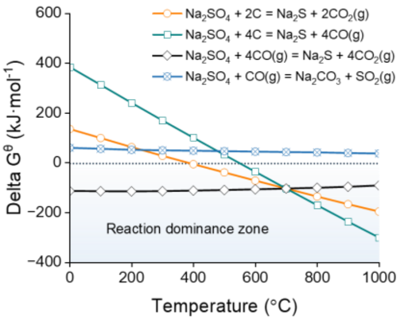

Supplementary Fig. 5 | Thermodynamic feasibility of carbothermal deoxygenation pathways for Na_2SO_4. The plot displays the standard Gibbs free energy (ΔG^{θ}) as a function of temperature for various reaction scenarios. Calculations were performed using HSC Chemistry 9.0 software under standard pressure (1 atm) with unit activity (activity: 1, settings: Show Transitions and Criss-Cobble).

Supplementary Fig. 5 elucidates the thermodynamic driving forces governing the deoxygenation of Na_2SO_4. As the temperature rises, the reduction of Na_2SO_4 to Na_2S via reaction with solid C or CO exhibits an increasingly negative ΔG^{θ}, indicating spontaneous reaction feasibility—particularly within the 600 – 800 °C window. In contrast, the hypothetical conversion of Na_2SO_4 to Na_2CO_3 (accompanied by SO_2 release) remains thermodynamically unfavorable ($\Delta G^{\theta} > 0$) across the entire investigated temperature range. Consequently, the carbothermal treatment of Na_2SO_4 selectively favors the formation of Na_2S over Na_2CO_3.

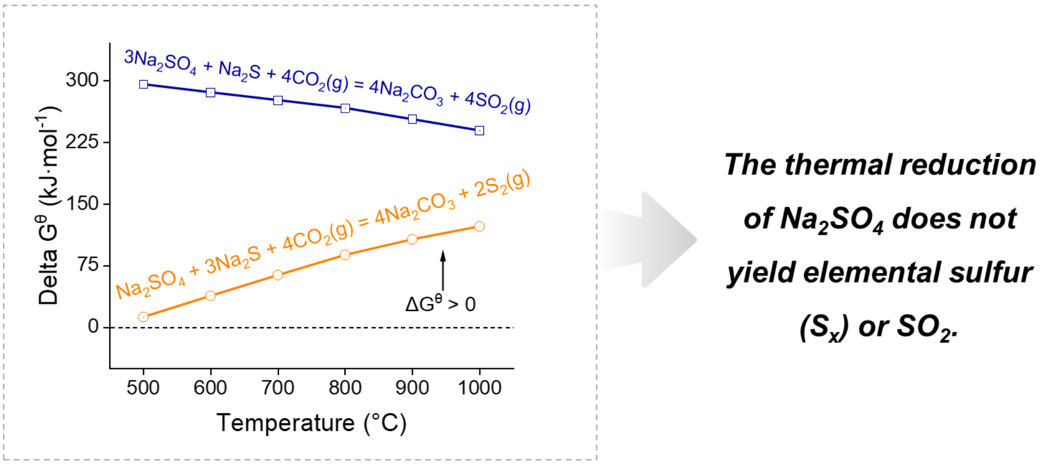

Supplementary Fig. 7 | Thermodynamic unfavourability of volatile sulfur species evolution during carbothermal reduction. The plot illustrates the standard Gibbs free energy (ΔG^{θ}) profiles for potential side reactions generating S_2(g) or SO_2(g) from the Na_2SO_4-Na_2S intermediate system. Calculations were performed using HSC Chemistry 9.0 software under standard pressure (1 atm) (activity: 1, settings: Show Transitions and Criss-Cobble).

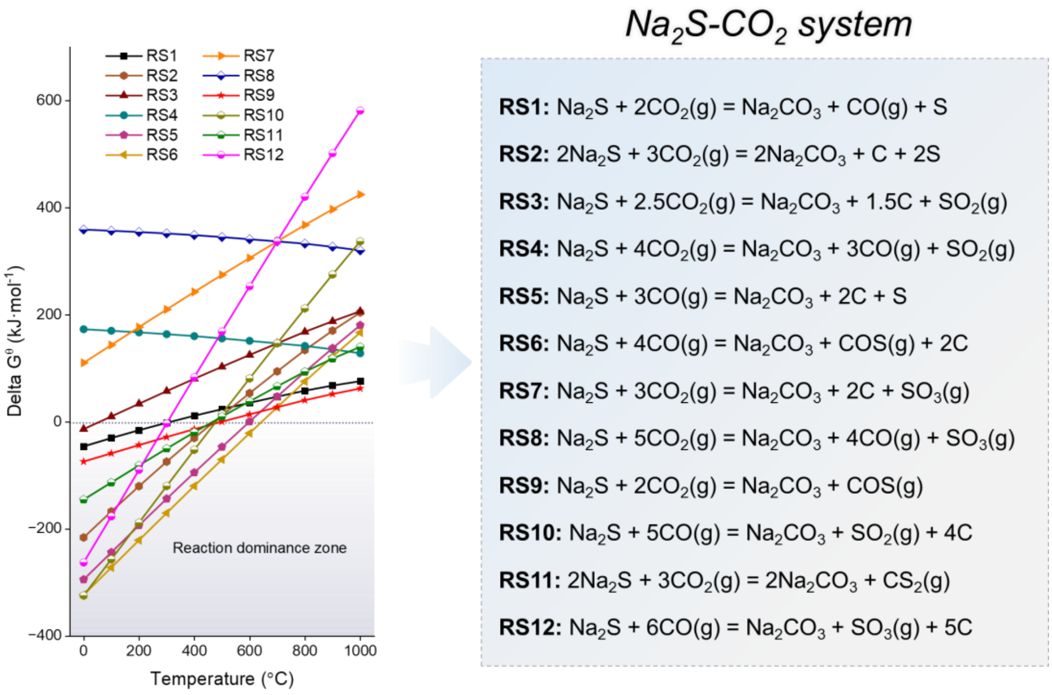

Supplementary Fig. 14 | Standard Gibbs free energy (ΔG^{θ}) profiles for the carbonation. The plot illustrates the temperature-dependent thermodynamic feasibility of the reaction in a pure CO_2 (1 atm). Thermodynamic calculations were performed using HSC Chemistry 9.0 software (activity: 1, settings: Show Transitions and Criss-Cobble).

8. Minor issues include typographical and formatting errors: in line 88, "tetrahedral structure of SO_4^{2-} is highly sTab." should read "stable"; in line 135, "like H_2 may serve ass viable options" should read "as"; and in Supplementary Table S4, the column labeled "Total 1 (frac)" should be corrected to "100 (%)" for consistency.

Reply: We sincerely apologize for the small errors that may have caused some confusion. Thank you for pointing these out. In response, we have thoroughly revised the manuscript to correct the issues you identified. We appreciate your attention to detail. Additionally, we have conducted a comprehensive review of the Revised Manuscript and Revised Supplementary Information to ensure that such issues do not occur again.

We sincerely appreciate you identifying these typographical and formatting errors. We apologize for the oversights, which resulted from unexpanded editing shortcuts and drafting abbreviations. We have explicitly corrected the errors ("stable" and "as"), and we have standardized the column labeling in **Supplementary Table 4** as suggested. Furthermore, we have conducted a comprehensive proofreading of the entire **Revised Manuscript** and **Revised Supplementary Information** to eliminate any remaining linguistic or formatting inconsistencies. We believe the presentation now fully aligns with the standards of your review.

Revise:

The Revised Results

Owing to the high thermodynamic stability and kinetic inertness of the SO_4^{2-} oxyanion (**Supplementary Fig. 4**), Na_2SO_4 is inherently resistant to decomposition and difficult to disrupt.

Additionally, the feasibility of using hydrogen (H_2) as a reducing agent was confirmed above 750 °C (**Supplementary Fig. 13**). As green H_2 costs decline, this presents a viable, ultra-clean alternative for the valorization of waste Na_2SO_4.

The Revised Supplementary Information

Supplementary Table 4 | Chemical composition of desulfurization waste determined by XRF. The data are adapted from **Supplementary Ref. 2**[2].

Components	Weight (%)		
C	1.29	Fe_2O_3	1.04

F	0.443	CuO	0.0108
**Na_2O**	**46.9**	ZnO	0.0236
MgO	0.051	SeO_2	0.0147
Al_2O_3	0.033	Br	0.0447
SiO_2	0.090	Rb_2O	0.0094
P_2O_5	0.0100	I	0.0696
**SO_3**	**39.3**	PbO	0.0818
**Cl**	**7.15**	Others	0.0384
K_2O	1.26	**Total**	**100**
CaO	2.14		

Reviewer #3

Comments:

In this study, the authors propose a novel process for producing Na_2CO_3 from Na_2SO_4. Considering the growing issue of large-scale Na_2SO_4 discharge, the topic and approach are timely and of potential interest. However, the main weakness of this work lies in the insufficient discussion of the engineering aspects of the proposed process. Although the method may offer certain mechanical or operational advantages compared with conventional processes, its practical feasibility remains unclear. Overall, the manuscript would benefit from a more comprehensive discussion of the engineering considerations, including scale-up strategies, process limitations, and future research directions.

Reply: We appreciate your recognition of the environmental and resource challenges posed by waste Na_2SO_4, as well as your sustained interest in this issue. As you correctly noted, our current study focuses on laboratory-scale experiments which have yet to undergo industrial validation. Existing literature confirms that the traditional SSA-Process relies heavily on NH_3 to facilitate carbonation; once NH_3 is removed, the degree of carbonation drops significantly [*Journal of Industrial and Engineering Chemistry*, **2023**, 122: 500-510]. Consequently, the primary objective of this work is to eliminate the dependency on NH_3 in the conversion of Na_2SO_4 to value-added Na_2CO_3.

We fully acknowledge your constructive comments regarding scale-up strategies, process limitations, and future research directions.

1. Regarding scale-up strategies: We anticipate that upgrading the corrosion resistance of reaction vessels will be critical to prevent product contamination and ensure purity. This is a standard engineering challenge that must be addressed during the transition to industrial-grade equipment.

2. Regarding process limitations: We recognize that the reliance on charcoal may present challenges related to supply chain stability and potential CO_2 emissions.

3. Regarding future directions: In response, we have proposed a transition toward green H_2 coupled with air CO_2 capture to achieve a cleaner, carbon-negative trajectory. We envision future research focusing on three primary scenarios (**Fig. R3-1**): (**i**) deploying this technology in regions lacking NH_3 synthesis infrastructure, (**ii**) establishing closed-loop self-production and self-consumption cycles within industrial parks, and (**iii**) integrating green H_2 and CO_2 capture to

specifically overcome the CO_2 emission and supply constraints associated with charcoal reduction.

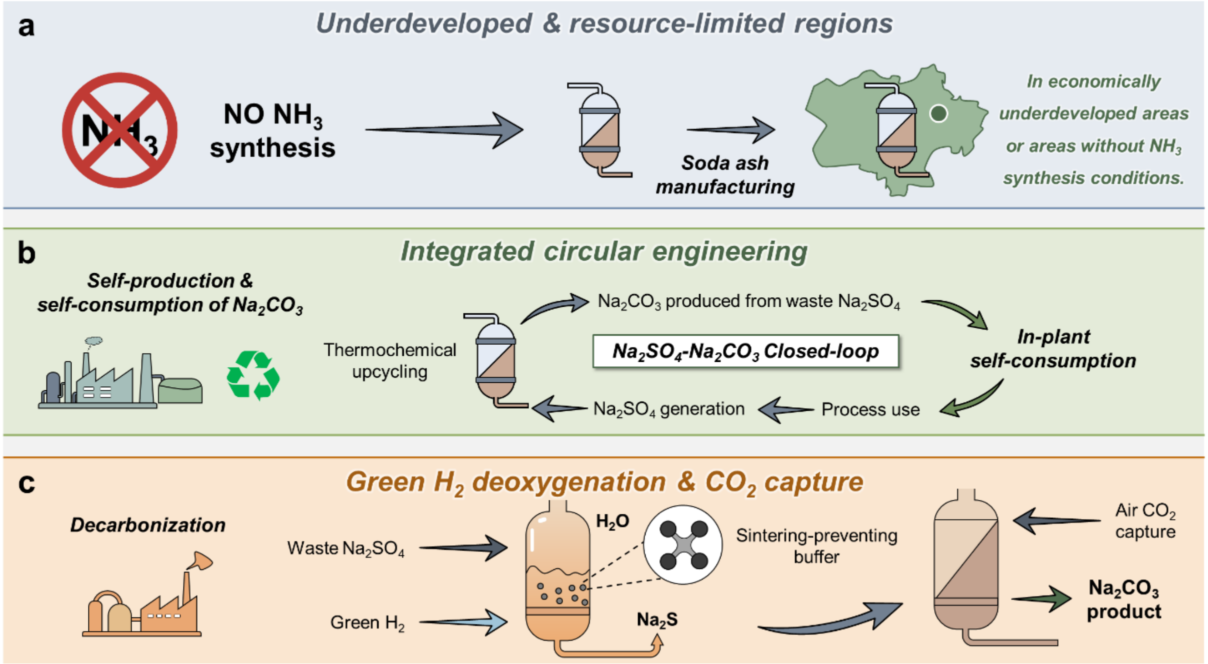

Fig. R3-1 | Future perspectives on industrial deployment and low-carbon evolution of the proposed technology. a, Deployment feasibility for economically underdeveloped regions or areas lacking synthetic NH_3 infrastructure, enabling decentralized, NH_3-free soda ash manufacturing. **b**, Strategy for integrated circular engineering to establish an on-site Na_2SO_4-Na_2CO_3 closed-loop, realizing internal self-sufficiency. **c**, Advanced decarbonization pathway coupling green H_2-based deoxygenation (utilizing sintering-preventing buffers in a fluidized bed) with direct air CO_2 capture.

We would greatly value any further insights or guidance you may have on these points. Finally, we have expanded the discussion in the **Revised Manuscript** and **Revised Supplementary Information** to address these aspects comprehensively and to clarify the issues you raised.

Revise:

The Revised Results

After purification yielded 35.52 g of white powder (**Supplementary Fig. 28**), XRD analysis confirmed the isolation of pure Na_2CO_3 with no detectable impurity peaks (**Fig. 5b**). The process achieved a yield of 95.35% relative to theoretical value (with further gains expected upon scale-up) and demonstrated a product purity exceeding 99.53%, containing only trace S and Si impurities originating from the glassware (**Table 1**). These minor impurities are likely mitigable by upgrading to industrial-scale equipment with superior corrosion resistance. Significantly, the presence of NaCl in

common mixed waste did not interfere with the thermochemical conversion of Na_2SO_4 (**Fig. 5c** and **Supplementary Table 4**).

Beyond process-level metrics, this work has broader implications for waste management and circular resource use. We estimate that unmanaged stockpiling of global waste Na_2SO_4 would squander ~1,864 acres of land annually (**Supplementary Fig. 39**). To demonstrate circularity, we tested a closed-loop application in Li_2CO_3 extraction by coupling a solar concentrator for dehydration with carbonate reagent regeneration (**Supplementary Fig. 40**). Furthermore, we developed a general "alkaline sodiation" strategy to extend this protocol to other complex sulfate wastes, effectively preventing hazardous SO_x emissions (**Supplementary Fig. 41**). Looking forward, we envision three application scenarios: (i) decentralized Na_2SO_4 upcycling in regions lacking NH_3 infrastructure, (ii) integrated on-site production-consumption loops within industrial parks, and (iii) a carbon-negative pathway coupling green H_2 deoxygenation with direct air CO_2 capture (**Supplementary Fig. 42**).

The Revised Supplementary Information

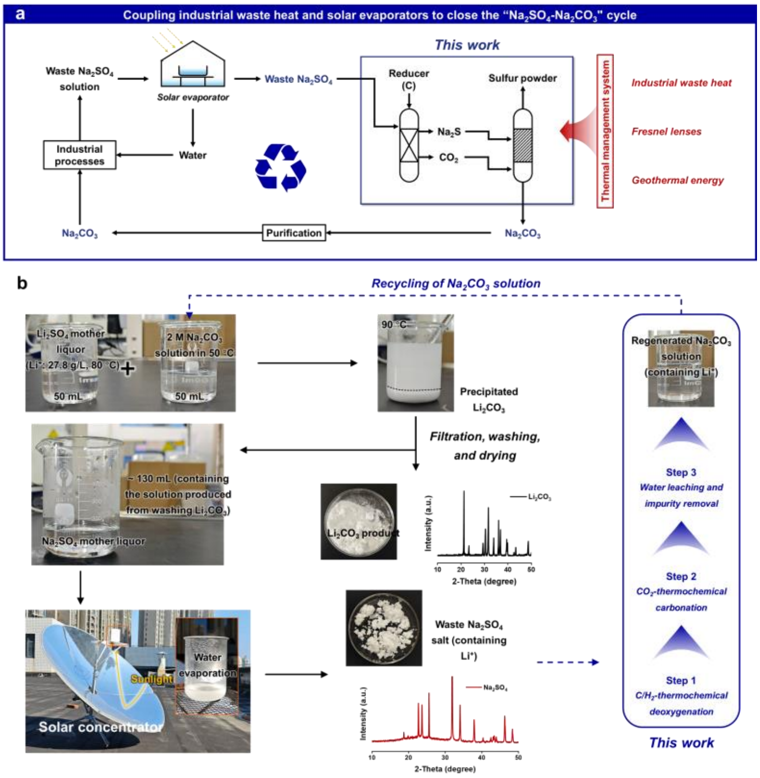

Supplementary Fig. 40 | Strategies for closing the "Na_2SO_4-Na_2CO_3" loop via multi-energy integration. a, Schematic illustration of the synergistic coupling of industrial waste heat and solar evaporators to achieve a sustainable Na_2SO_4-Na_2CO_3 cycle. The system integrates solar thermal energy (e.g., Fresnel lenses) and geothermal energy to optimize thermal management. **b**, Practical

demonstration of this cycle applied to lithium extraction. The process involves the precipitation of Li_2CO_3 followed by the concentration of the Na_2SO_4 mother liquor using a solar concentrator. The recovered Na_2SO_4 solid is then processed via the proposed thermochemical route (Steps 1-3) to regenerate the Na_2CO_3 solution for reuse.

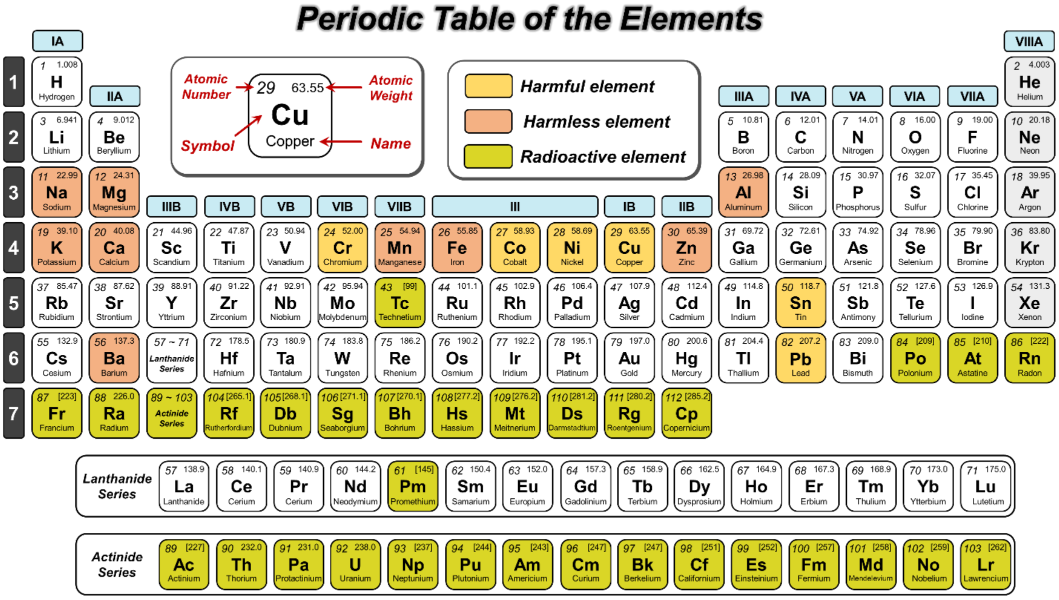

Supplementary Fig. 41 | Overview of typical waste sulfates (e.g., Na_2SO_4, $CaSO_4$, $FeSO_4$) identified as potential renewable resources.

Beyond Na_2SO_4, there are diverse sulfate byproducts in many industrial processes, such as $FeSO_4$ from TiO_2 manufacturing, $CaSO_4$ from phosphate extraction, and $NiSO_4$ from spent LIBs. The thermochemical route proposed herein can serve as a versatile "soda regeneration hub" coupled with a pre-treatment strategy we term "alkaline sodiation." Taking the ubiquitous $FeSO_4$ as an example: direct carbothermal reduction typically releases hazardous SO_2 gas. However, by introducing Na-based alkalis (Na_2CO_3 or NaOH), complex sulfates can be converted into valuable metal oxides (e.g., Fe_xO_y for metallurgical feedstocks) and Na_2SO_4. This intermediate Na_2SO_4 is subsequently processed via our technology to regenerate the soda ash reagents required for the initial conversion and to recover S_x. This synergistic integration allows for the closed-loop valorization of a wide spectrum of sulfate wastes, avoiding SO_2 emissions while recovering critical metals and sulfur resources.

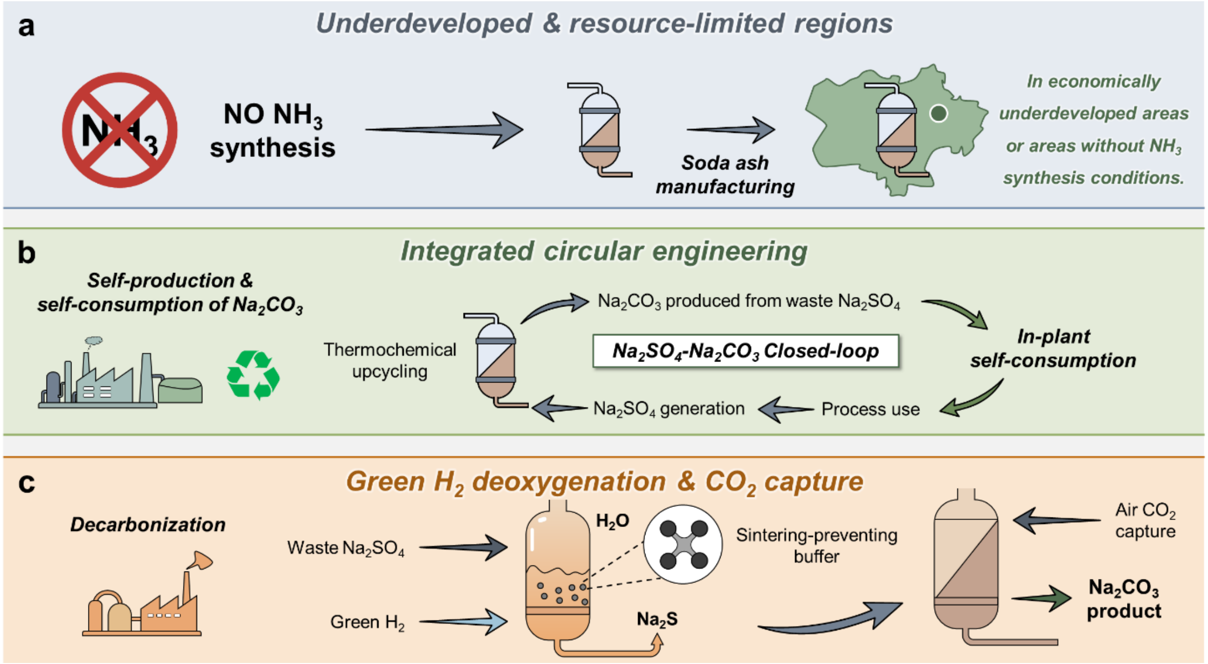

Supplementary Fig. 42 | Future perspectives on industrial deployment and low-carbon evolution of the proposed technology. a, Deployment feasibility for economically underdeveloped regions or areas lacking synthetic NH_3 infrastructure, enabling decentralized, NH_3-free soda ash manufacturing. **b**, Strategy for integrated circular engineering to establish an on-site Na_2SO_4-Na_2CO_3 closed-loop, realizing internal self-sufficiency. **c**, Advanced decarbonization pathway coupling green H_2-based deoxygenation (utilizing sintering-preventing buffers in a fluidized bed) with direct air CO_2 capture.

1. To validate the feasibility of the proposed Na_2CO_3 production process, a techno-economic analysis should be performed. For example, although the manuscript mentions some drawbacks of bipolar membrane electrodialysis (BMED), BMED could still exhibit better economic viability when considering the cost of NaOH. Therefore, the authors should include a comparative TEA to quantitatively assess the economic feasibility of the proposed process relative to conventional methods.

Reply: We sincerely appreciate your insightful suggestion regarding the necessity of a techno-economic analysis to validate the feasibility of our proposed process. We agree that a quantitative economic assessment is essential to benchmark our technology against BMED and the new SSA-Process. Although BMED has not yet achieved widespread industrial operation, we recognize its potential economic viability driven by the value of NaOH co-production.

To address this, we have incorporated a comprehensive life cycle cost (LCC) analysis in the **Revised Manuscript** and **Revised Supplementary Information** (**Table R3-1**). As a foundation for this assessment, we aggregated market price trends over the past five years for key commodities including Na_2CO_3, S_x, NaOH, and H_2SO_4 (**Fig. R3-2**).

Table R3-1 | Summary of life cycle costs (LCC) for BMED (yielding H_2SO_4 and NaOH), the SSA-Process, the New SSA-Process, and this work.

Parameter	BMED	New SSA-Process	This work
**1. Material & Energy Costs (USD)**			
Feedstock			
Waste $Na_2SO_4 \cdot 10H_2O$	0.00[a]	0.00[a]	0.00[a]
Energy			
Electricity	439.90[b] (189.16)[b]	219.47[b] (94.37)[b]	54.51[b] (23.44)[b]
Chemical Reagents			
Charcoal (Reactant)	—	—	22.37[c]
NH_3	—	—	—
CO_2	—	4.78[d]	—
Process water/solvent	13.53[e]	18.00[f]	1.64[e]
**Total Costs (USD)**	**453.43 (202.69)**	**242.25 (117.15)**	**78.52 (47.45)**
**2. Revenue Streams (USD)**			
Main Product			

H_2SO_4	34.17[g]	—	—
NaOH	113.25[h]	—	—
Na_2CO_3	—	97.04	97.04
Byproducts			
$(NH_4)_2SO_4$	—	109.79[i]	—
S_x	—	—	18.28
**Total Revenue (USD)**	**147.42**	**206.83**	**115.32**
	**3. Economic Performance (USD)**		
**Gross Profit**	**−306.01 (−55.27)**	**−35.42 (+89.68)**	**+36.80 (+67.87)**
**Profit Margin (%)**	**−207.58% (−37.49%)**	**−17.13% (+43.36%)**	**+31.91% (+58.85%)**

a: Waste $Na_2SO_4 \cdot 10H_2O$ is assumed to be burden-free (0.00 USD) as it is a waste stream.

b: Baseline electricity cost is set at 0.10 $USD \cdot kWh^{-1}$. A sensitivity scenario considers a reduced rate of 0.043 $USD \cdot kWh^{-1}$, reflecting the global weighted average cost of utility-scale solar PV (Source: IRENA, https://www.irena.org/).

c: Charcoal (reductant) is priced at 400 $USD \cdot ton^{-1}$ based on prevailing market rates in China; future adoption of low-cost alternative carbon sources could further reduce expenses.

d: Industrial CO_2 is priced at 35 $USD \cdot ton^{-1}$, reflecting capture costs in the Chinese region (Source: CCUS and Global Power Generation Analysis).

e: Process water is valued at 1.50 $USD \cdot ton^{-1}$.

f: Solvent costs are based on Chinese market prices: MEA at 1,000 $USD \cdot ton^{-1}$ and MEG at 500 $USD \cdot ton^{-1}$. A makeup rate of 5% per cycle is assumed to account for operational losses.

g: Product revenue for NaOH and H_2SO_4 is calculated on a 100% pure substance basis (e.g., the output of NaOH is equivalent to 250 kg of pure NaOH).

h: Same as note **g**; H_2SO_4 production is also calculated on a 100% basis.

i: The market value of $(NH_4)_2SO_4$ is estimated at 150% of the standard fertilizer-grade price. This premium reflects the higher purity and economic value achieved through the MEA-MEG purification process compared to conventional fertilizer products.

Limitations: The economic analysis is restricted to the variable operating expenditures (OPEX) associated with material and energy inputs. Fixed costs, including capital expenditures (CAPEX) for plant construction, equipment depreciation, and labor costs, are excluded from this assessment.

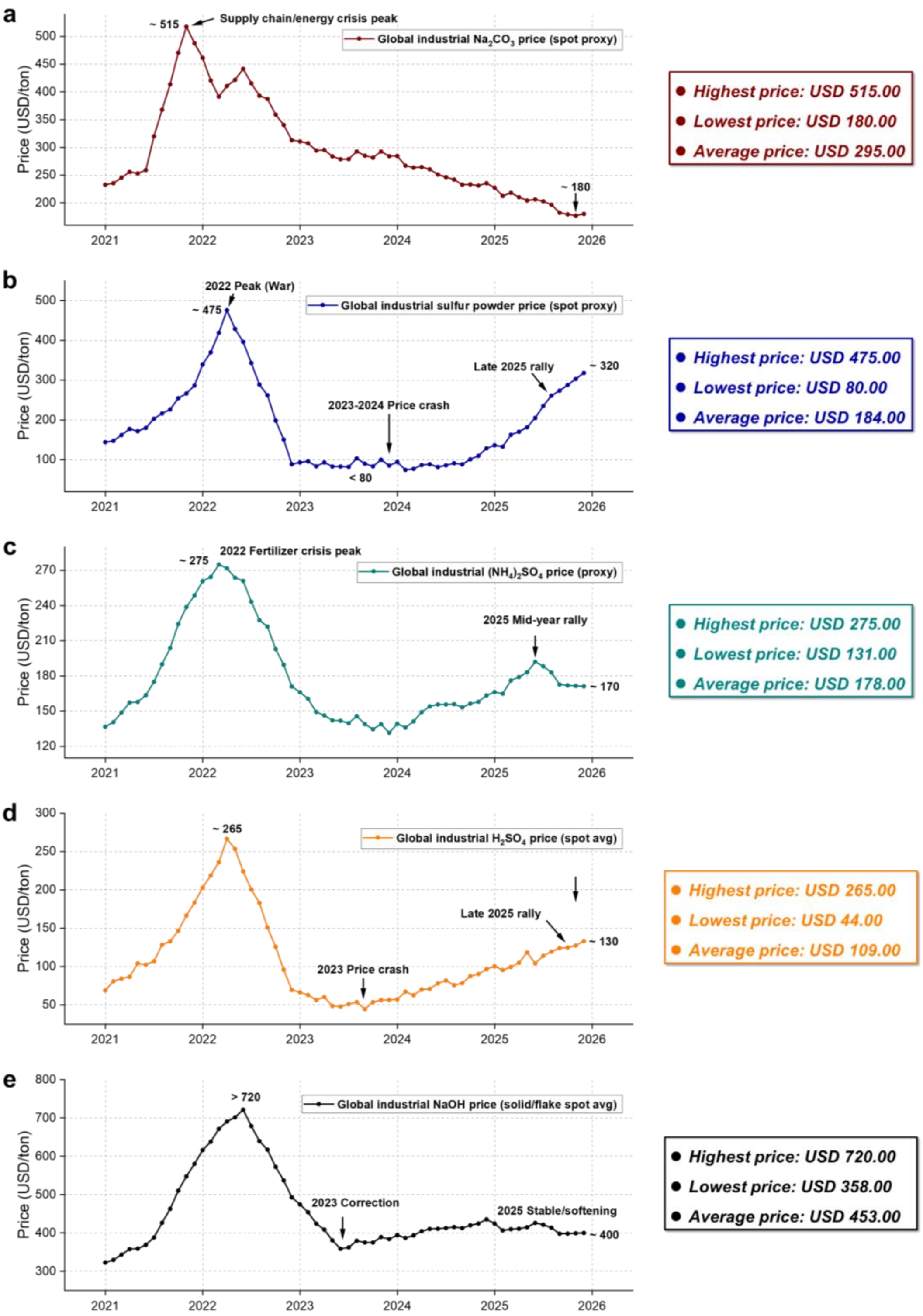

Fig.R3-2 | The global price trend of the relevant products in the past five years. a, Na_2CO_3. **b**, Sulfur powder. **c**, $(NH_4)_2SO_4$. **d**, H_2SO_4. **e**, NaOH.

Our LCC evaluation reveals that the primary economic bottleneck for BMED lies in the concentration of its acid and base products. The process typically yields dilute solutions which require energy-intensive concentration steps to facilitate cost-effective transportation. Without concentration,

the application scenarios for BMED products are severely restricted by logistical costs (**Table R3-1**). Furthermore, we compared the sensitivity of this work and competing technologies to electricity costs (**Fig. R3-3**). The results indicate that the competitive advantage of BMED is heavily dependent on future low-cost photovoltaic electricity. Consequently, we conclude that the optimal application scenario for BMED is within PV-driven clean manufacturing facilities. In such settings, BMED is best suited for on-site self-consumption where low-concentration reagents (approximately 1 M) are utilized directly to eliminate transportation requirements.

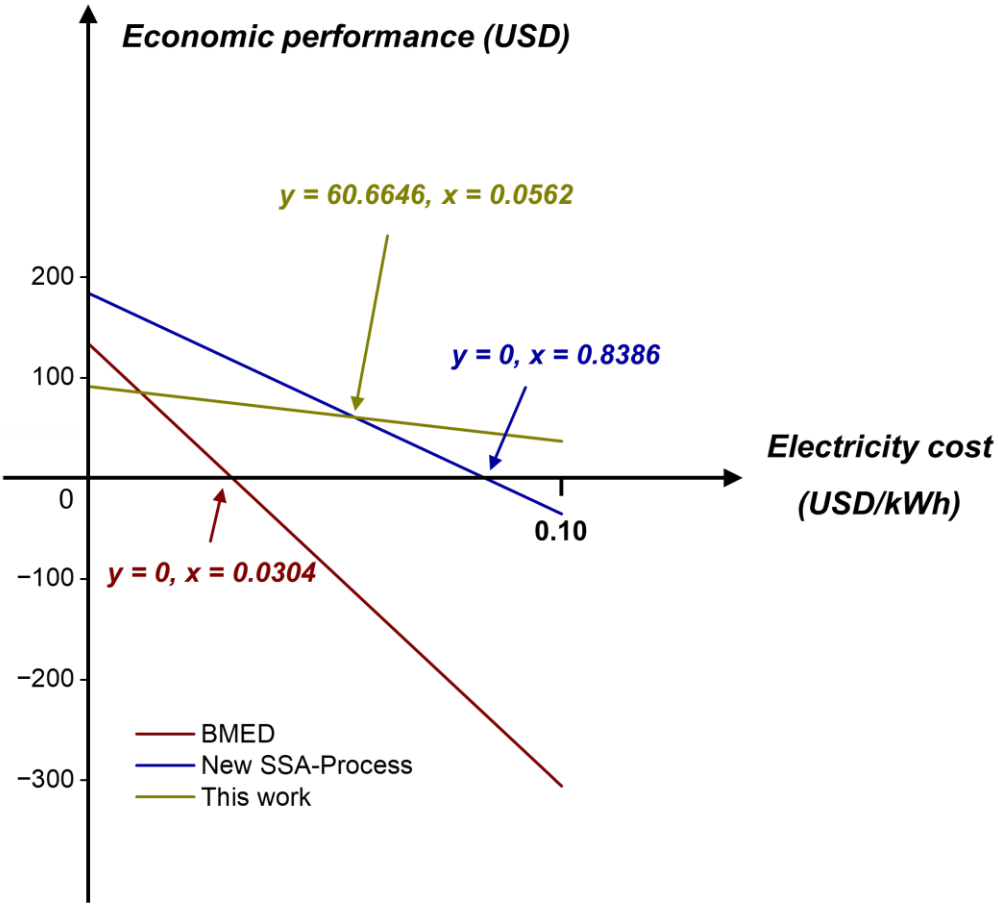

Fig.R3-3 | Sensitivity analysis of economic performance to electricity price fluctuations. The linear relationship between gross profit (USD) and electricity cost (USD/kWh) is compared for the BMED process (red), new SSA-Process (blue), and the proposed thermochemical route (this work, olive). The arrows indicate the break-even points (where profit equals zero) and the crossover threshold ($x \approx 0.056$ USD/kWh), beyond which the proposed route becomes economically superior to the new SSA-Process. The significantly flatter slope of the proposed route demonstrates its higher insensitivity against rising energy costs compared to the electricity-intensive BMED and new SSA-Processes.

Revise:

The Revised Results

The economic evaluation was broadened to include BMED as a comparative benchmark via a detailed LCC analysis (**Supplementary Tables 12-13**). The proposed route demonstrates superior profitability, yielding +36.80 USD per ton of waste $Na_2SO_4 \cdot 10H_2O$ (rising to +67.87 USD under PV-driven scenarios), representing a profit margin of 31.91% (58.85%) (**Table 2**). Crucially, sensitivity analysis reveals high economic robustness; our route becomes the most cost-effective option when electricity costs exceed $0.056 per kWh and shows minimal sensitivity to electricity price increases (**Supplementary Fig. 37**). Additionally, our process exhibits higher market stability because soda ash contributes 84% of total product revenue (**Supplementary Fig. 38**), whereas the New SSA-Process remains vulnerable to supply-demand fluctuations in the $(NH_4)_2SO_4$ market.

Table 2. Summary of economic analysis (fundamental unit: 1 ton of waste $Na_2SO_4 \cdot 10H_2O$).

Parameter	BMED	New SSA-Process	This work
**1. Costs (USD)**			
Electricity[a]	439.90 (189.16)	219.47 (94.37)	54.51 (23.44)
Other Materials (Reagents & Water)	13.53	22.78	24.01
Total Production Cost	453.43 (202.69)	242.25 (117.15)	78.52 (47.45)
**2. Revenue (USD)**			
Total Revenue	147.42	206.83	115.32
(Main Products)	NaOH, H_2SO_4	Na_2CO_3	Na_2CO_3
(Byproducts)	—	$(NH_4)_2SO_4$	S_x
**3. Profitability**			
Gross Profit (USD)	−306.01 (−55.27)	−35.42 (+89.68)	+36.80 (+67.87)
Profit Margin (%)	−207.58% (−37.49%)	−17.13% (+43.36%)	+31.91% (+58.85%)

Note: Values in parentheses (**a**) represent the scenario using renewable energy (PV) electricity costs (USD 0.043 per kWh) instead of the standard grid electricity cost (USD 0.10 per kWh), For more detailed information, please refer to **Supplementary Table 13**.

The Revised Supplementary Information

Supplementary Table 13 | Summary of life cycle costs (LCC) for BMED (yielding H_2SO_4 and NaOH), the SSA-Process, the New SSA-Process, and this work.

Parameter	BMED	New SSA-Process	This work
	**1. Material & Energy Costs (USD)**		
Feedstock			
Waste $Na_2SO_4 \cdot 10H_2O$	0.00[a]	0.00[a]	0.00[a]
Energy			
Electricity	439.90[b] (189.16)[b]	219.47[b] (94.37)[b]	54.51[b] (23.44)[b]
Chemical Reagents			
Charcoal (Reactant)	—	—	22.37[c]
NH_3	—	—	—
CO_2	—	4.78[d]	—
Process water/solvent	13.53[e]	18.00[f]	1.64[e]
**Total Costs (USD)**	**453.43 (202.69)**	**242.25 (117.15)**	**78.52 (47.45)**
	**2. Revenue Streams (USD)**		
Main Product			
H_2SO_4	34.17[g]	—	—
NaOH	113.25[h]	—	—
Na_2CO_3	—	97.04	97.04
Byproducts			
$(NH_4)_2SO_4$	—	109.79[i]	—
S_x	—	—	18.28
**Total Revenue (USD)**	**147.42**	**206.83**	**115.32**
	**3. Economic Performance (USD)**		
**Gross Profit**	**−306.01 (−55.27)**	**−35.42 (+89.68)**	**+36.80 (+67.87)**
**Profit Margin (%)**	**−207.58% (−37.49%)**	**−17.13% (+43.36%)**	**+31.91% (+58.85%)**

a: Waste $Na_2SO_4 \cdot 10H_2O$ is assumed to be burden-free (0.00 USD) as it is a waste stream.

b: Baseline electricity cost is set at 0.10 USD·kWh^{-1}. A sensitivity scenario considers a reduced rate of 0.043 USD·kWh^{-1}, reflecting the global weighted average cost of utility-scale solar PV (Source: IRENA, https://www.irena.org/).

c: Charcoal (reductant) is priced at 400 USD·ton^{-1} based on prevailing market rates in China; future adoption of low-cost alternative carbon sources could further reduce expenses.

d: Industrial CO_2 is priced at 35 USD·ton^{-1}, reflecting capture costs in the Chinese region (Source: CCUS and Global Power Generation Analysis).

e: Process water is valued at 1.50 USD·ton^{-1}.

f: Solvent costs are based on Chinese market prices: MEA at 1,000 USD·ton^{-1} and MEG at 500 USD·ton^{-1}. A makeup rate of 5% per cycle is assumed to account for operational losses.

g: Product revenue for NaOH and H_2SO_4 is calculated on a 100% pure substance basis (e.g., the output of NaOH is equivalent to 250 kg of pure NaOH).

h: Same as note **g**; H_2SO_4 production is also calculated on a 100% basis.

i: The market value of $(NH_4)_2SO_4$ is estimated at 150% of the standard fertilizer-grade price. This premium reflects the higher purity and economic value achieved through the MEA-MEG purification process compared to conventional fertilizer products.

Limitations: The economic analysis is restricted to the variable operating expenditures (OPEX) associated with material and energy inputs. Fixed costs, including capital expenditures (CAPEX) for plant construction, equipment depreciation, and labor costs, are excluded from this assessment.

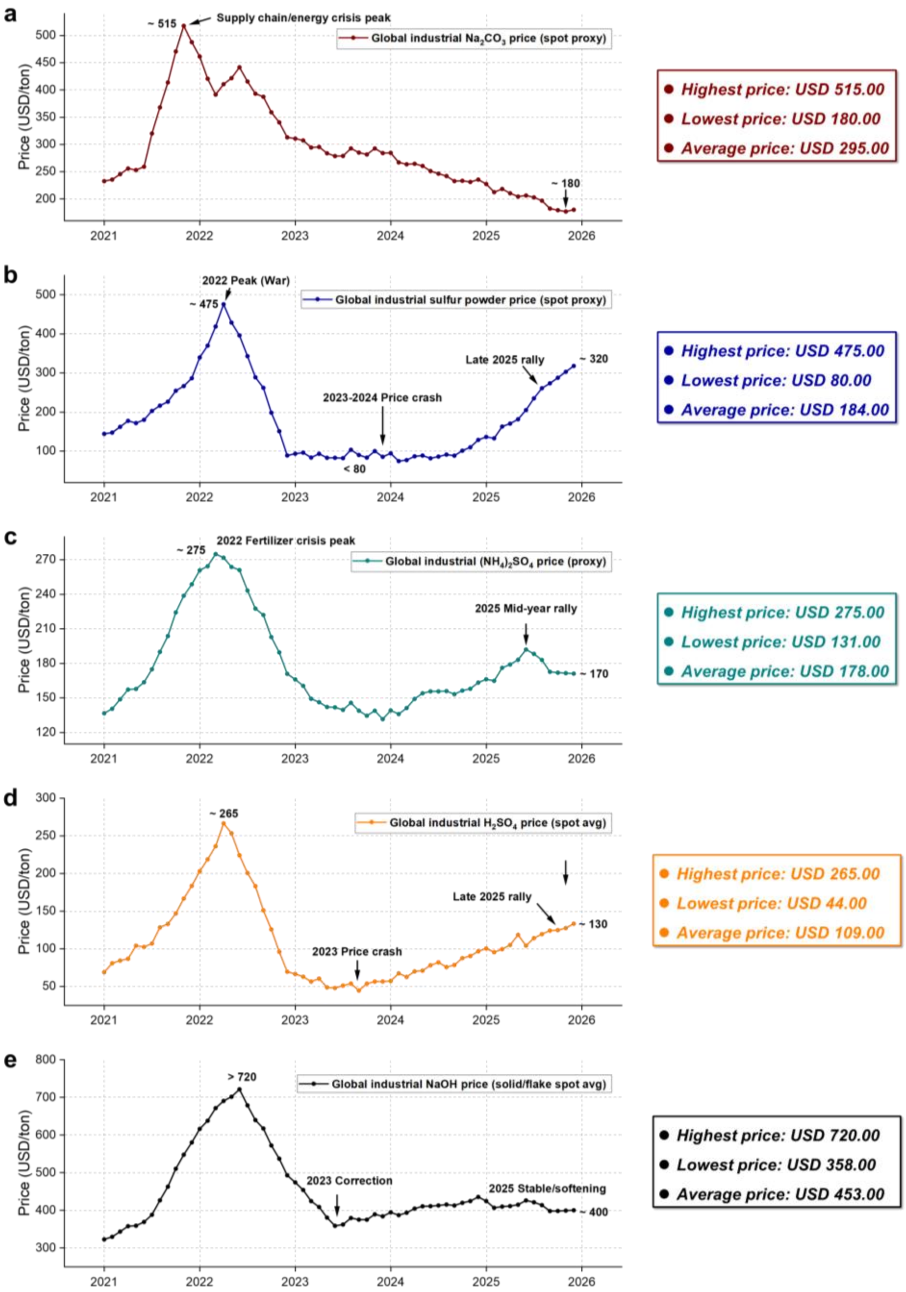

Supplementary Fig. 35 | The global price trend of the relevant products in the past five years. a, Na_2CO_3. **b**, Sulfur powder. **c**, $(NH_4)_2SO_4$. **d**, H_2SO_4. **e**, NaOH.

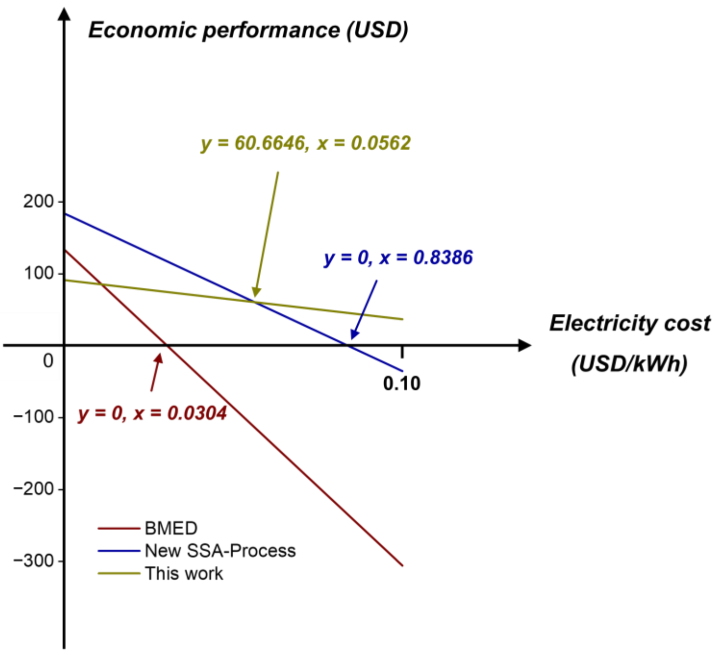

Supplementary Fig. 37 | Sensitivity analysis of economic performance to electricity price fluctuations. The linear relationship between gross profit (USD) and electricity cost (USD/kWh) is compared for the BMED process (red), new SSA-Process (blue), and the proposed thermochemical route (this work, olive). The arrows indicate the break-even points (where profit equals zero) and the crossover threshold (x ≈ 0.056 USD/kWh), beyond which the proposed route becomes economically superior to the new SSA-Process. The significantly flatter slope of the proposed route demonstrates its higher insensitivity against rising energy costs compared to the electricity-intensive BMED and new SSA-Processes.

2. What is the production yield of Na_2CO_3 from Na_2SO_4 in the proposed process? Additionally, while the authors claim that SO_x emissions are inhibited, incomplete reactions could potentially lead to the formation of sulfur oxides. Please provide further discussion on the productivity, reaction completeness, and possible side reactions.

Reply: We are deeply grateful for your positive evaluation of our work and appreciate your rigorous attention to detail regarding the production yield and possible side reactions.

Regarding the production yield, we sincerely apologize if <u>the recovery rate of 95.35% achieved in our 50 g scale-up experiment</u> was not sufficiently prominent in the original **Manuscript**. To address this, we have now explicitly highlighted this key metric in both the ***Abstract*** and ***Discussion*** sections to ensure immediate clarity for readers while noting that the minor mass loss (~5%) is primarily due to manageable mechanical factors like filtration of Na_2CO_3 and volatilization of Na_2S rather than reaction inefficiency.

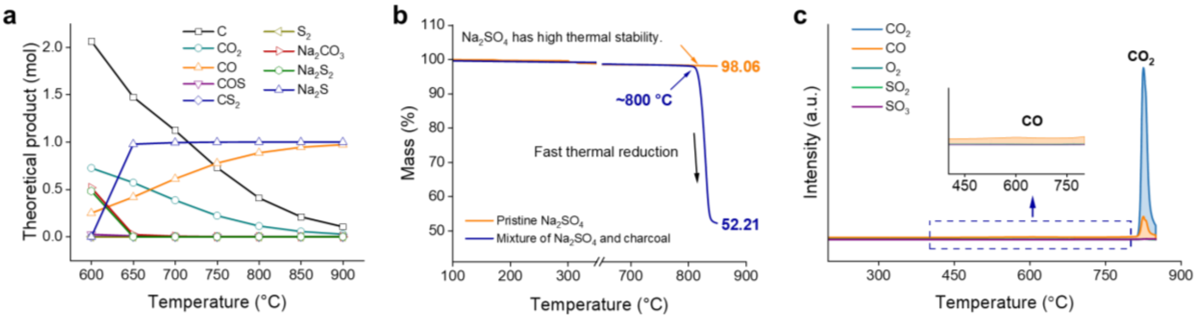

Fig. R3-4 | Thermochemical deoxygenation behavior of Na_2SO_4. a, Thermodynamic equilibrium analysis of the Na_2SO_4-C system (molar ratio: 1:4, reaction pressure: 1 atm, concentration cut-off: > 10^{-5} mol, and activity: 1) derived from FactSage 8.3 calculations, predicting product distribution and phase stability across the temperature range. **b**, Thermogravimetric (TG) profiles comparing the thermal decomposition behaviors of pristine Na_2SO_4 and the Na_2SO_4/C mixture (molar ratio: 1:4, heating rate: 10 $°C \cdot min^{-1}$, atmosphere: He). **c**, Mass spectrometry (MS) profiles monitoring the evolution of gaseous species from the Na_2SO_4/C mixture.

Second, regarding your valid concern about SO_x emissions, we have conducted a broader investigation. First, for the conversion of Na_2SO_4-to-Na_2S, we demonstrated in the original manuscript that SO_x is not generated (**Fig. R3-4**, corresponding to **Fig. 2** in **Manuscript**), as supported by chemical equilibrium data, thermodynamic calculations, and GC-MS results. To further explore potential side reactions between Na_2S and CO_2, we supplemented our study with a GC-MS analysis of the product

gas composition (**Fig. R3-5**). We initially explored *in-situ* XRD or XAS to analyze this process; however, technical constraints regarding high-temperature CO_2 atmospheres and, more critically, the complex interference caused by the inherent hygroscopicity of Na_2S rendered this unfeasible. Instead, we conducted rigorous GC-MS analysis to elucidate the gas-phase composition. Most importantly, the results confirmed that SO_2 and SO_3 are not generated in the reaction system, with levels remaining below detection limits (< 10 ppm). This validates the clean fixation of sulfur in our process. A minor byproduct, carbonyl sulfide (COS), was detected; however, semi-quantitative analysis indicated that the COS content is < 2%, likely originating from the spontaneous recombination of CO and S_x. In an industrial context, such trace impurities can be readily removed via standard separation techniques (e.g., low-temperature liquefaction separation).

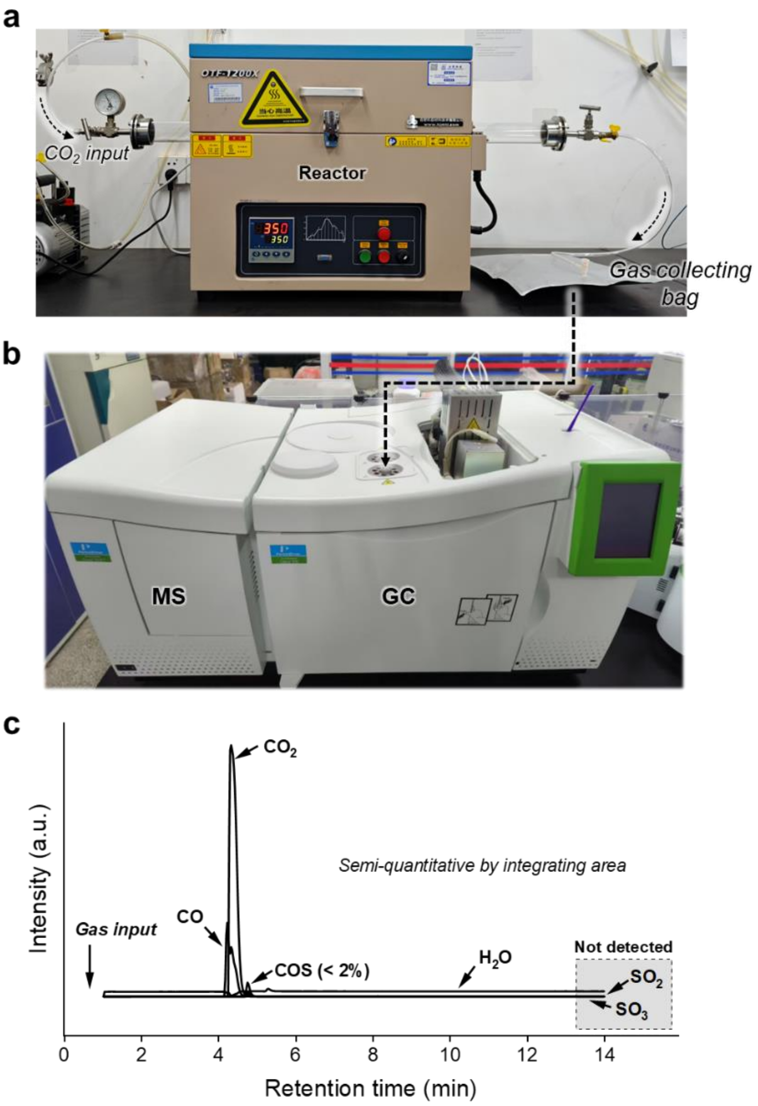

Fig. R3-5 | Investigation of potential side reactions between Na_2S and CO_2. a, Digital photograph

of the experimental setup used for reaction gas collection. **b**, The GC-MS instrument. **c**, Total ion chromatogram (TIC) identifying the gaseous byproducts. Test conditions: The system was operated in full scan mode to capture the complete spectrum of each data point during the chromatographic analysis. A 1 μL gas sample was injected into the chromatographic column with He (99.999%) as the carrier gas at a flow rate of 1.0 mL·min^{-1}. The temperature program involved holding at 40 °C for 2 min, followed by a ramp to 300 °C at a rate of 8 °C·min^{-1}. The mass selective detector's electron impact (EI) ion source temperature was set at 200 °C, and the mass spectra were analyzed in the range of 0 – 300 atomic mass units (amu).

In summary, the thermochemical conversion of Na_2SO_4 to Na_2CO_3 has been thoroughly validated, with no SO_x generation detected throughout the process. We have incorporated these findings into the **Revised Manuscript** and **Revised Supplementary Information** to address concerns regarding reaction completeness and side reactions, thereby ensuring that the article more accurately reflects the true experimental mechanisms and results.

Revise:

The Revised Abstract

Waste sodium sulfate (Na_2SO_4) is a common industrial byproduct that poses environmental risks and resource loss if improperly managed. Here, we report a thermochemical upcycling method to convert waste Na_2SO_4 into value-added sodium carbonate (Na_2CO_3) and sulfur (S_x). In this process, Na_2SO_4 is first reduced to sodium sulfide (Na_2S) at 750 °C using charcoal. Subsequently, the generated Na_2S is oxidized by CO_2 via carbonation at 300 °C to produce Na_2CO_3 and S_x. Temperature modulation shifts thermodynamic equilibrium to drive the conversion of SO_4^{2-} to S_x, achieving a carbonate yield of 95.35% with purity exceeding 99.53%. Life cycle assessment (LCA) indicates that this anhydrous route reduces the global warming potential by > 0.43 kg CO_2-eq per kg Na_2CO_3 compared with the conventional sodium sulfate-based ammonia-soda process (SSA-Process). By eliminating water-intensive steps and ammonia usage, our method lowers the end-point environmental impact to 34.69 mPt per kg Na_2CO_3 (vs. 48.81 mPt for conventional routes). Overall, this work provides a sustainable strategy for reclaiming waste salts and closing the sodium and sulfur cycles.

The Revised Results

To rigorously assess potential pollutant formation, the gas composition at 350 °C was analyzed by gas chromatography-mass spectrometry (GC-MS) (**Supplementary Fig. 26**). Crucially, the

analysis confirmed the absence of detectable SO_x, validating the kinetic and thermodynamic inhibition of SO_x generation in this system. The product gas consisted almost exclusively of CO_2 (carrier gas) and CO (reduction byproduct), with only negligible traces of carbonyl sulfide (COS) observed. In summary, this NH_3-free thermochemical strategy achieves the clean, site-independent upcycling of waste Na_2SO_4 into high-value Na_2CO_3, successfully overcoming the scalability and pollution constraints inherent in conventional precipitation-based routes.

Supplementary Information

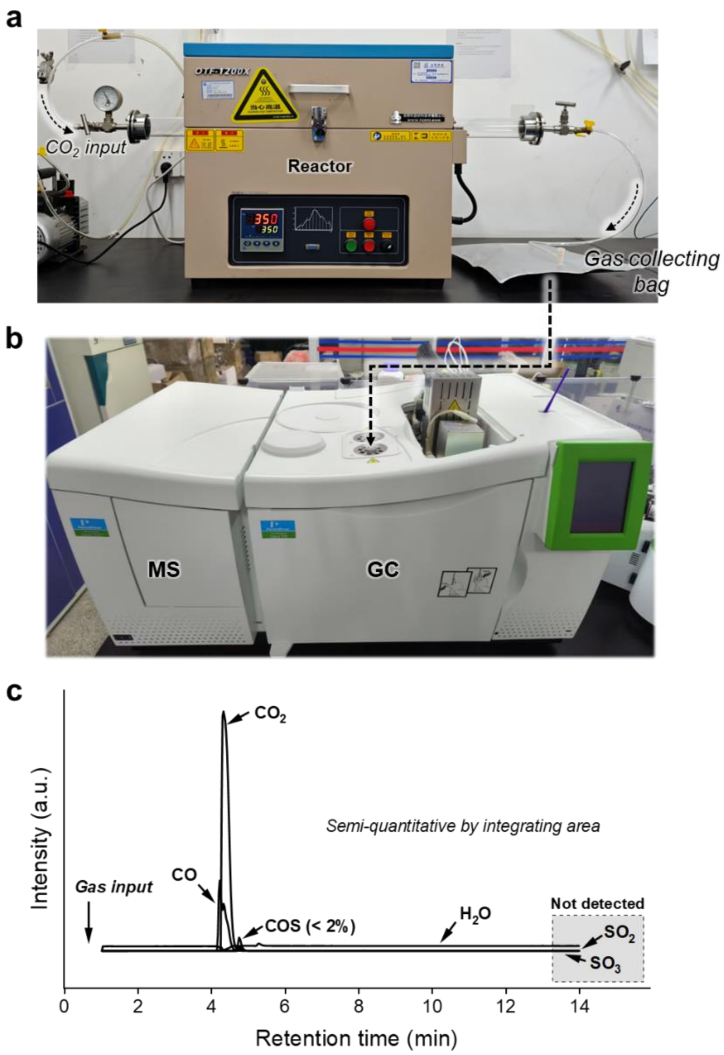

Supplementary Fig. 26 | Investigation of potential side reactions between Na_2S and CO_2. a, Digital photograph of the experimental setup used for reaction gas collection. **b**, The GC-MS instrument. **c**, Total ion chromatogram (TIC) identifying the gaseous byproducts. Test conditions: The system was operated in full scan mode to capture the complete spectrum of each data point during the chromatographic analysis. A 1 μL gas sample was injected into the chromatographic column with He (99.999%) as the carrier gas at a flow rate of 1.0 $mL\cdot min^{-1}$. The temperature program involved holding

at 40 °C for 2 min, followed by a ramp to 300 °C at a rate of 8 °C·min^{-1}. The mass selective detector's electron impact (EI) ion source temperature was set at 200 °C, and the mass spectra were analyzed in the range of 0 – 300 atomic mass units (amu).

The Revised Discussion

In this work, we demonstrate a two-step thermochemical route to upcycle waste Na_2SO_4 to Na_2CO_3 and S_x. The process leverages temperature modulation, in which carbon disrupts sulfate structures at 750 °C, followed by a shift to sulfide carbonation at 300 °C to achieve a Na_2CO_3 yield of 95.35%. Environmentally, this anhydrous route circumvents the water-intensive liquid-phase carbonation and eliminates NH_3 use required in conventional SSA-Process. Consequently, it reduces GWP by > 0.43 kg CO_2-eq per kg Na_2CO_3 while exhibiting superior economic stability against electricity price fluctuations (> $0.056 per kWh). Moreover, this technology demonstrates the lowest end-point environmental impact (34.69 mPt per kg Na_2CO_3) among the evaluated methods. On a broader scale, this approach enables decentralized deployment in resource-limited regions lacking NH_3 synthesis infrastructure and facilitates the integration of diverse sulfate waste recycling streams. Looking forward, the future integration of low-carbon deoxygenation and CO_2 capture could potentially transform this technology into a carbon-negative solution. This positions waste Na_2SO_4 as a critical vector for closing sodium and sulfur cycles and achieving deep decarbonization.

3. In Figure 4, it is unclear how the authors controlled or mitigated the formation of volatile S_x species and residual carbon on the Na_2CO_3 product. Please clarify the control strategy and its influence on product quality.

Reply: We sincerely appreciate your query regarding the control strategy for volatile sulfur species and residual carbon. We acknowledge that our original description of the separation mechanism may not have been sufficiently explicit, and we have revised the **Manuscript** to address this.

We clarify that product separation relies on the distinct physicochemical properties and phase behaviors of the reaction components:

- **Carbon behavior:** Elemental carbon remains solid and non-volatile under the reaction conditions.
- **Sulfur pathways:** Elemental sulfur follows two distinct pathways during the process: a significant portion volatilizes and is recovered via condensation, while the remaining fraction exists as a solid mixture with the residual carbon. We note that at a future industrial scale, the recovery of high-purity volatile sulfur can be further optimized by reducing the sulfur partial pressure and facilitating the rapid extraction of liquid sulfur.

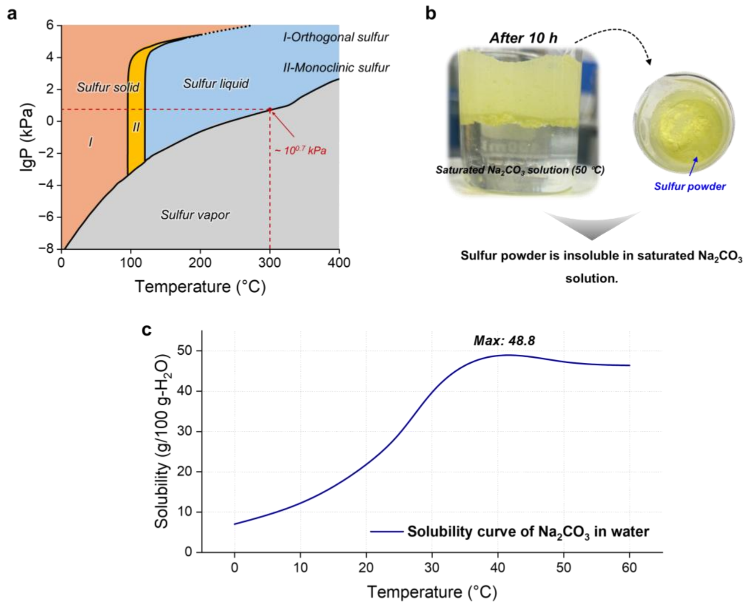

Fig. R3-6 | Physicochemical basis for the separation and recovery of Na_2CO_3 and S_x. a, Phase diagram showing the temperature-dependent saturation vapor pressure of sulfur. The rapid increase in vapor pressure at elevated temperatures facilitates sulfur recovery via sublimation/vaporization **b**, Solubility test demonstrating the chemical inertness and insolubility of S_x powder in a saturated

solution (50 °C) after a 10 h immersion. **c**, Temperature-dependent aqueous solubility profile of Na_2CO_3, indicating high solubility favorable for water leaching.

To ensure the purity of the Na_2CO_3 product, we employ a water leaching strategy that leverages solubility differences. Since Na_2CO_3 is highly soluble in water whereas elemental sulfur and carbon are insoluble (**Fig. R3-6**), these solid impurities (residual carbon and non-volatilized sulfur) are effectively removed via filtration (**Fig. R3-7**). This process ensures that the final carbonate product is free from residual carbon and sulfur contamination.

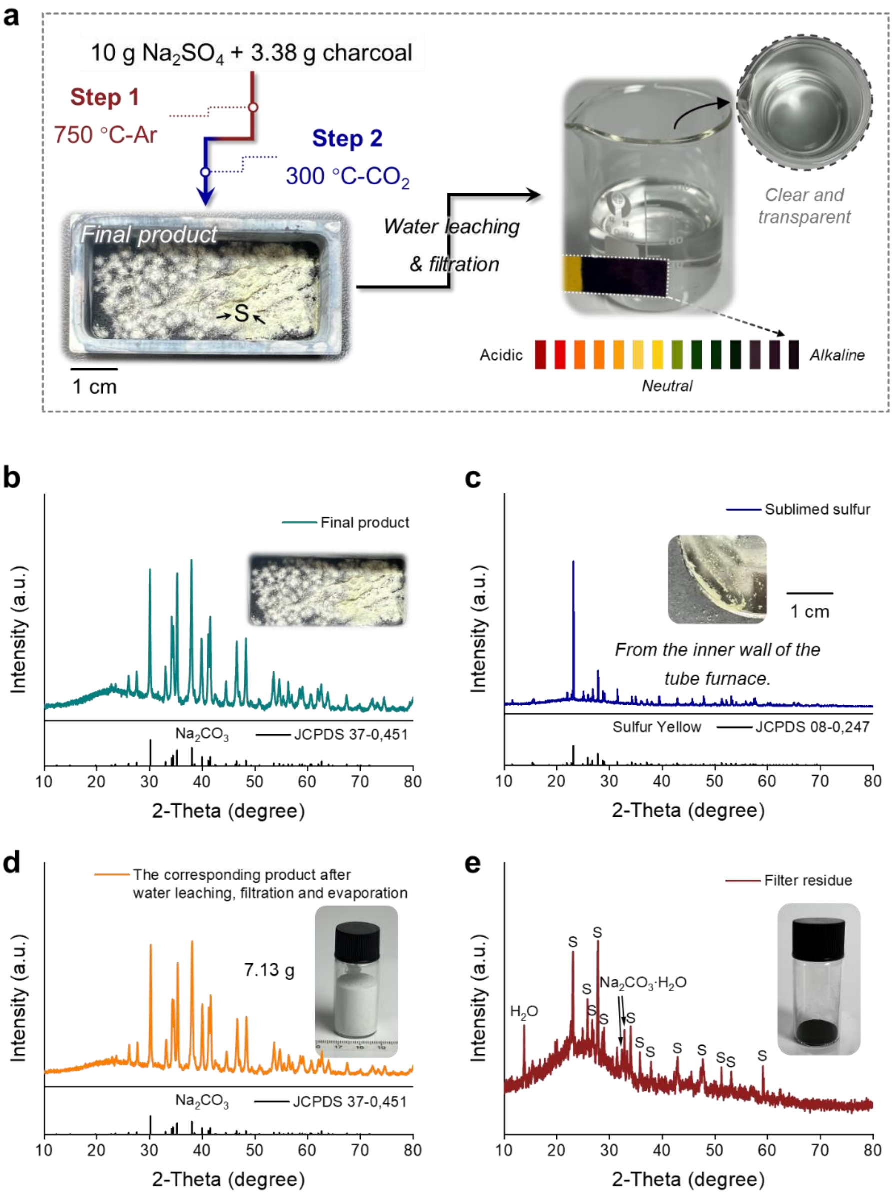

Fig. R3-7 | Conversion of Na_2SO_4 to Na_2CO_3 via a sequential gas-flow strategy. a, Schematic workflow and digital photographs illustrating the two-step process: carbothermal reduction under Ar (750 °C) followed by carbonation under CO_2 (300 °C). **b-e**, XRD patterns of the distinct product

fractions: the crude solid product after reaction (**b**), sublimed S_x collected from the reactor walls (**c**), purified Na_2CO_3 obtained after water leaching and evaporation (**d**), and the insoluble filter residue (**e**).

We have comprehensively illustrated this mass distribution and the separation workflow in the **Supplementary Fig. 19** (**Revised Supplementary Information**) to further clarify the impurity control mechanism

Revise:

The Revised Results

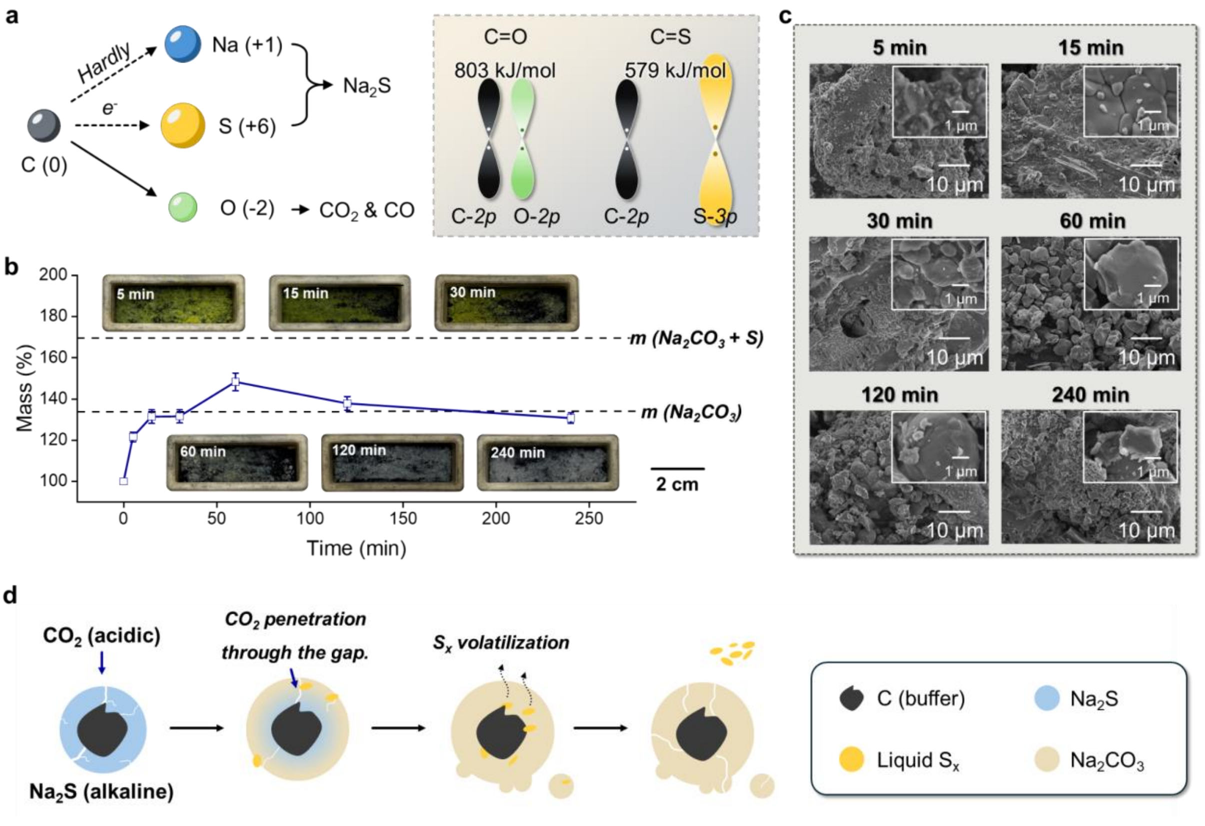

Fig. 4 | Transformation mechanism from Na_2SO_4 to Na_2CO_3 and S_x. a, Schematic of the selective reduction mechanism of Na_2SO_4 to Na_2S, driven by the bond dissociation energy difference between C=O and C=S bonds. **b**, Time-resolved mass variation profile during the carbonation of the Na_2S intermediate, with optical insets tracking the macroscopic color transition from yellow to white. **c**, SEM images revealing the morphological evolution of the solid products at different reaction stages (5-240 min). **d**, Proposed reaction model illustrating the CO_2 diffusion-controlled carbonation process and simultaneous S_x volatilization on the C buffer.

The Revised Supplementary Information

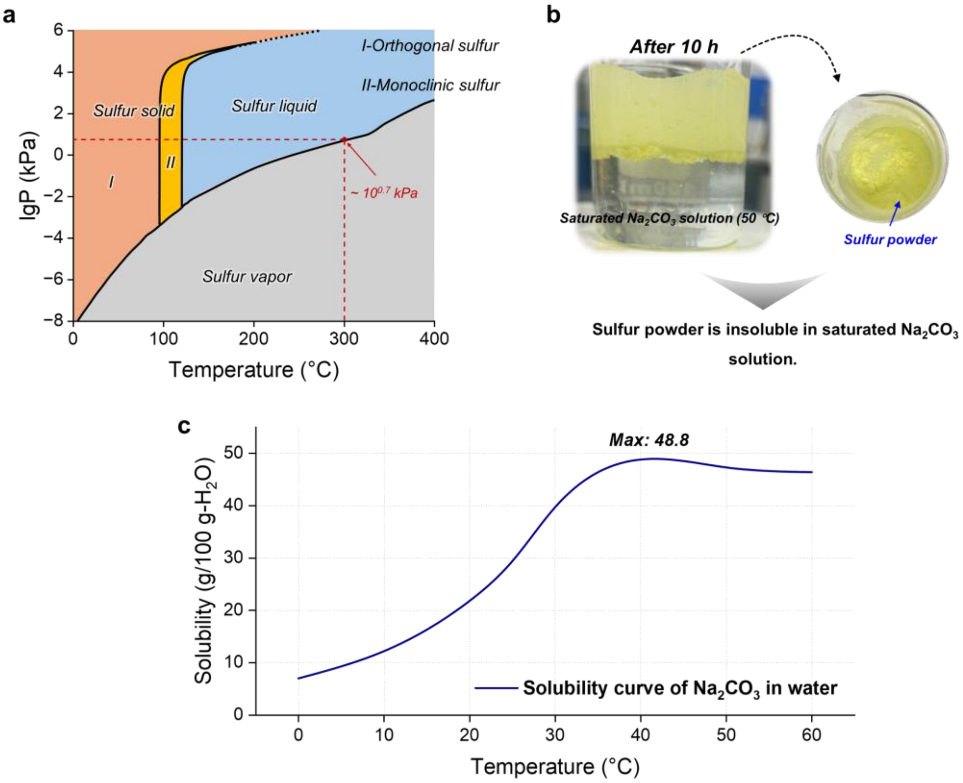

Supplementary Fig. 23 | Physicochemical basis for the separation and recovery of Na_2CO_3 and S_x. a, Phase diagram showing the temperature-dependent saturation vapor pressure of sulfur. The rapid increase in vapor pressure at elevated temperatures facilitates sulfur recovery via sublimation/vaporization **b**, Solubility test demonstrating the chemical inertness and insolubility of S_x powder in a saturated solution (50 °C) after a 10 h immersion. **c**, Temperature-dependent aqueous solubility profile of Na_2CO_3, indicating high solubility favorable for water leaching.

The effective separation of the reaction products relies on the distinct physicochemical properties of the components. As illustrated in **Supplementary Fig. 23a**, sulfur exhibits a significant rise in saturation vapor pressure with temperature (e.g., transitioning to the vapor phase above 300 °C at reduced pressures), which provides the thermodynamic driving force for its removal via sublimation or evaporation during the heating process. Conversely, the solid residue (Na_2CO_3) is recovered through water leaching. **Supplementary Fig. 23b** confirms that S_x remains insoluble and chemically stable in saturated Na_2CO_3 solutions, preventing cross-contamination during the leaching step. Furthermore, the high aqueous solubility of Na_2CO_3 (**Supplementary Fig. 23c**, reaching 48.8 g/100g water) ensures efficient recovery and crystallization of the final carbonate product.

4. Although the manuscript focuses primarily on mechanistic studies, the engineering aspects of the process are not sufficiently addressed. The authors should expand the discussion to include process limitations, purity control, and potential scale-up strategies, as well as future research directions necessary to advance this process toward industrial application.

Reply: We sincerely appreciate your constructive comment regarding the need to address the engineering aspects of the process. We agree that expanding the discussion to include process limitations, scale-up strategies, and future directions is essential for bridging the gap between our current mechanistic studies and industrial application.

In the **Revised Manuscript**, we have added a comprehensive discussion covering the following points:

- **Scale-up strategies and purity control**: We acknowledge that our current findings are based on laboratory-scale experiments. For industrial scale-up, ensuring the corrosion resistance of reaction vessels is critical to preventing product contamination. We view this as a standard engineering challenge that must be addressed through material selection to maintain the high purity observed in the lab.
- **Process limitations**: We recognize that the current reliance on charcoal may present challenges regarding supply chain stability and potential carbon emissions.
- **Future research directions**: To address these challenges and advance the technology, we have outlined three primary future scenarios (**Fig. R3-1**):
    i. *Decentralized deployment*: Implementing the technology in resource-limited regions that lack synthetic ammonia infrastructure.
    ii. *Industrial symbiosis*: Establishing closed-loop self-production and consumption cycles within industrial parks to minimize logistics.
    iii. *Green transition*: Coupling the process with green H_2 and air CO_2 capture. This strategy specifically aims to overcome the limitations of charcoal reduction, thereby evolving the process into a cleaner, carbon-negative solution.

We have incorporated these engineering perspectives into the **Revised Manuscript** and **Revised Supplementary Information** section to provide a more holistic view of the technology’ potential.

We sincerely appreciate the time and expertise you have dedicated to improving our manuscript.

Your constructive comments have significantly strengthened the quality and rigor of this work. We remain entirely open to further inquiries and would eagerly welcome any additional guidance or the opportunity to discuss new insights should you have them.

Revise:

The Revised Results

The primary objective of this technological development is the clean and low-carbon scale-up of converting waste Na_2SO_4 into value-added Na_2CO_3. Accordingly, an upscaled production process was carried out (**Fig. 5a**). EDS analysis characterized the intermediate powder as a mixture of Na_2CO_3 and S_x (**Supplementary Fig. 27**). After purification yielded 35.52 g of white powder (**Supplementary Fig. 28**), XRD analysis confirmed the isolation of pure Na_2CO_3 with no detectable impurity peaks (**Fig. 5b**). The process achieved a yield of 95.35% relative to theoretical value (with further gains expected upon scale-up) and demonstrated a product purity exceeding 99.53%, containing only trace S and Si impurities originating from the glassware (**Table 1**). These minor impurities are likely mitigable by upgrading to industrial-scale equipment with superior corrosion resistance. Significantly, the presence of NaCl in common mixed waste did not interfere with the thermochemical conversion of Na_2SO_4 (**Fig. 5c** and **Supplementary Table 4**).

The Revised Supplementary Information

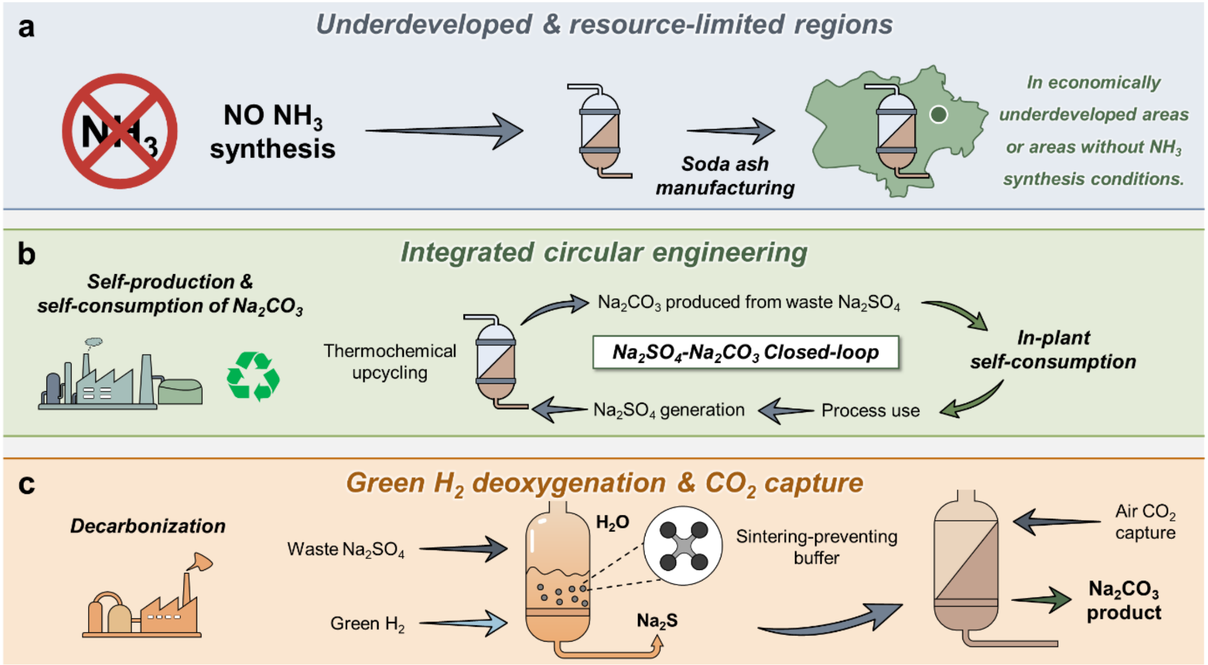

Supplementary Fig. 42 | Future perspectives on industrial deployment and low-carbon evolution of the proposed technology. a, Deployment feasibility for economically underdeveloped regions or areas lacking synthetic NH_3 infrastructure, enabling decentralized, NH_3-free soda ash manufacturing. **b**, Strategy for integrated circular engineering to establish an on-site Na_2SO_4-Na_2CO_3 closed-loop, realizing internal self-sufficiency. **c**, Advanced decarbonization pathway coupling green H_2-based deoxygenation (utilizing sintering-preventing buffers in a fluidized bed) with direct air CO_2 capture.

Response to referees

Journal: Nature Communications

Manuscript ID: NCOMMS-25-65907A

Title: Upcycling of waste sodium sulfate to sodium carbonate and sulfur

Author(s): Hongya Wang, Shiyu Wang, Fengyin Zhou, Muya Cai, Jingjing Zhao, Danfeng Wang, Zhan Shi, Xiang Chen, Dihua Wang, Huayi Yin*

We are grateful to all the reviewers for their comments and suggestions. We have revised the manuscript, taking into full account the reviewers' comments and recommendations. Below are our point-to-point responses in the same order as the reviewers' comments. Replies are marked in blue, revisions are marked in purple.

Reviewer #1

Comments:

Review of revised Nature Communications manuscript NCOMMS-25-65907A entitled "Upcycling of waste sodium sulfate to sodium carbonate and sulfur".

I have carefully examined the authors' responses to the reviewers' comments, including my own, as well as the corresponding revisions made to the manuscript.

I am pleased to confirm that the authors have addressed all the concerns I raised in my previous report comprehensively and thoughtfully. Their revisions include:

Detailed clarification of the reaction mechanisms and pathways, which significantly strengthens the scientific rigor of the work.

A more thorough and quantitative comparison between the proposed process (this study) and existing industrial (SSA-process) or reported methods (New SSA-Process). This comparative analysis has been substantially enhanced with relevant data and discussion, providing a clear and convincing context for the novelty and advantages of their approach.

Strengthened evidence and discussion supporting the claimed high credibility of the results. The authors have added necessary experimental details, data validation, and more nuanced interpretation where appropriate.

The revised manuscript now presents a more complete, robust, and well-justified study. The technical improvements are clear, and the narrative has been polished to better highlight the

significance and potential impact of this waste upcycling strategy. The work demonstrates a sound methodological approach, and the conclusions are well-supported by the presented data.

Overall, the revision process has markedly improved the quality of the manuscript. The current version is coherent, technically solid, and presents a significant advancement in the field of chemical upcycling and resource recovery. The level of scientific exposition and experimental substantiation now meets the high standards expected for publication in a leading journal.

Reply: My co-authors and I sincerely thank you for your positive assessment. We are deeply grateful for your time and insightful guidance throughout the review process. Your constructive feedback was instrumental in perfecting the manuscript and significantly elevated the quality of our work to meet the higher standards of the journal.

Reviewer #2

Comments:

The manuscript presents an innovative thermochemical process for upcycling waste Na_2SO_4 into Na_2CO_3 and elemental sulfur via carbothermal reduction followed by CO_2 carbonation. The authors have made substantial efforts to address the concerns raised in the previous review, and the revised manuscript shows improvement in clarity, methodological transparency, and robustness of the analyses.

The major issues identified in the first round have been satisfactorily addressed. However, one remaining issue concerns the economic assessment described as "LCC," which requires substantial clarification for methodological transparency and reproducibility.

[Detailed Comments]

1. Major comment (LCC / economic analysis): The revised manuscript includes cost-related results with breakdowns and electricity price sensitivity. However, the calculation framework is not sufficiently described in either the main text or the Supplementary Information (e.g., system boundary, included cost components, and cost aggregation procedure/equations), making it difficult to assess and reproduce the analysis. In addition, the assessment appears limited to OPEX, raising concerns about whether the term "LCC" is appropriate without a clear definition of scope. Please either provide a clear and reproducible description of the LCC methodology (scope/system boundary, cost items, and calculation procedure) or revise the terminology to accurately reflect an operating-cost-based techno-economic assessment.

Reply: First and foremost, we sincerely thank you for your comprehensive review. Your rigorous scrutiny in the previous rounds was instrumental in resolving the majority of issues and elevating the quality of this work to its current level. We deeply appreciate the time and effort you have dedicated to this process.

Regarding your concern about the appropriateness of the terminology "life cycle cost (LCC)," we fully agree that this point is highly valid. We have carefully considered your feedback and recognize the absolute necessity of clarifying the LCC methodology, specifically regarding the inclusion of cost components and the calculation procedures.

In both the previous and current versions, the economic assessment is strictly derived from the life cycle inventory (LCI) data (**Supplementary Tables 5**, **7**, and **12**), scaled to a functional unit of

1,000 kg of waste $Na_2SO_4 \cdot 10H_2O$. We apologize that our prior calculations focused solely on variable operating expenditures (Variable OPEX) and omitted capital-related costs (e.g., equipment depreciation, labor, and maintenance). We acknowledge that this omission rendered the term "LCC" technically inaccurate. To rectify this, we have expanded the cost model to include an estimation of fixed costs, which explicitly encompass annualized capital expenditures (CAPEX). Drawing on standard heuristics in the chemical process industry, we defined these comprehensive fixed costs to account for 15% of the total LCC, while variable OPEX accounts for the remaining 85%. Although a precise bottom-up CAPEX calculation is challenging given the current laboratory-scale data, this factorial estimation effectively bridges the gap between laboratory estimates and industrial reality, ensuring our calculations fully align with standard LCC definitions.

In this revision, we have comprehensive corrections throughout the section of LCC in **Revised Supplementary Information**. Specifically, we have added a detailed LCC calculation procedure in **Supplementary Note 2**. Furthermore, following the integration of fixed costs, we have updated the electricity price sensitivity analyses to better reflect real-world production scenarios.

We hope these revisions satisfactorily address your concerns and significantly enhance the credibility and transparency of the economic assessment.

Revise:

The Revised Results

The economic evaluation was broadened to include BMED as a comparative benchmark via a detailed LCC analysis (**Supplementary Note 2** and **Tables 12-13**). The proposed route demonstrates superior profitability, yielding a net profit of +22.94 USD per ton of waste $Na_2SO_4 \cdot 10H_2O$ (rising to +59.50 USD under PV-driven scenarios), representing a profit margin of 19.89% (51.59%) (**Table 2**). Crucially, sensitivity analysis reveals high economic robustness. Our route becomes the most cost-effective option when electricity costs exceed \$0.0479 per kWh and shows minimal sensitivity to electricity price increases (**Supplementary Fig. 37**). Additionally, our process exhibits higher market stability because soda ash contributes 84% of total product revenue (**Supplementary Fig. 38**), whereas the New SSA-Process remains vulnerable to supply-demand fluctuations in the $(NH_4)_2SO_4$ market.

Table 2 | Summary of economic analysis (fundamental unit: 1 ton of waste $Na_2SO_4·10H_2O$).

Parameter	BMED	New SSA-Process	This work
**1. Costs (USD)**			
Electricity	439.90 (189.16)	219.47 (94.37)	54.51 (23.44)
Other Materials (Reagents & Water)	13.53	22.78	24.01
Fixed Costs (*Sum of maintenance, labor, etc*)	80.02 (35.77)	42.75 (20.67)	13.86 (8.37)
Total LCC	533.45 (238.46)	285.00 (137.82)	92.38 (55.82)
**2. Revenue (USD)**			
Total Revenue	147.42	206.83	115.32
(Main Products)	NaOH, H_2SO_4	Na_2CO_3	Na_2CO_3
(Byproducts)	—	$(NH_4)_2SO_4$	S_x
**3. Profitability**			
Net Profit (USD)	−386.03 (−91.04)	−78.17 (+69.01)	+22.94 (+59.50)
Profit Margin (%)	−261.86% (−61.76%)	−37.79% (+33.36%)	+19.89% (+51.59%)

Note: Values in parentheses denote the scenario using renewable solar (PV) electricity (0.043 USD per kWh[46]) rather than standard grid electricity (0.10 USD per kWh). The total LCC encompasses variable operating expenditures (OPEX, including electricity and other materials) and fixed costs (estimated at 15% of the total LCC). For detailed calculation procedures and comprehensive data breakdowns, please refer to **Supplementary Note 2** and **Supplementary Table 13**.

The Revised Supplementary Information

Supplementary Table 13 | Summary of life cycle costs (LCC) for BMED (yielding H_2SO_4 and NaOH), the New SSA-Process, and this work. The assessment is based on a functional unit of processing 1,000 kg of waste $Na_2SO_4 \cdot 10H_2O$. Calculations utilize average market prices over the past five years (see **Supplementary Fig. 35** for historical price fluctuations of all products). Primary values are calculated based on coal-fired electricity generation, while values in parentheses represent a future PV-driven scenario.

Parameter	BMED	New SSA-Process	This work
**1. Variable Operating Expenditures (OPEX) (USD)**			
Feedstock			
Waste $Na_2SO_4 \cdot 10H_2O$	0.00[a]	0.00[a]	0.00[a]
Energy			
Electricity	439.90[b] (189.16)[b]	219.47[b] (94.37)[b]	54.51[b] (23.44)[b]
Chemical Reagents			
Charcoal (Reactant)	—	—	22.37[c]
NH_3	—	—	—
CO_2	—	4.78[d]	—
Process water/solvent	13.53[e]	18.00[f]	1.64[e]
**Total Variable OPEX (USD)**	**453.43 (202.69)**	**242.25 (117.15)**	**78.52 (47.45)**
**2. Fixed Costs (USD)**			
**Fixed Costs (Depreciation, Labor, etc.)[g]** *(Calculated as 15% of Total LCC)*	**80.02 (35.77)**	**42.75 (20.67)**	**13.86 (8.37)**
**3. Total LCC (USD)**			
(Sum of Variable OPEX + Fixed Costs)	**533.45 (238.46)**	**285.00 (137.82)**	**92.38 (55.82)**
**4. Revenue Streams (USD)**			
Main Product			
H_2SO_4	34.17[h]	—	—
NaOH	113.25[i]	—	—
Na_2CO_3	—	97.04	97.04
Byproducts			
$(NH_4)_2SO_4$	—	109.79[j]	—
S_x	—	—	18.28

**Total Revenue (USD)**	**147.42**	**206.83**	**115.32**
**5. Economic Performance (USD)**			
**Net Profit**	**−386.03**	**−78.17**	**+22.94**
(Total Revenue − Total LCC)	(−91.04)	**(+69.01)**	**(+59.50)**
**Profit Margin (%)**	**−261.86%**<br>**(−61.76%)**	**−37.79%**<br>**(+33.36%)**	**+19.89%**<br>**(+51.59%)**

a: Waste $Na_2SO_4{\cdot}10H_2O$ is assumed to be burden-free (0.00 USD) as it is a waste stream.

b: Baseline electricity cost is set at 0.10 USD·kWh^{-1}. A sensitivity scenario considers a reduced rate of 0.043 USD·kWh^{-1}, reflecting the global weighted average cost of utility-scale solar PV (Source: International Renewable Energy Agency (IRENA), https://www.irena.org/).

c: Charcoal (reductant) is priced at 400 USD·ton^{-1} based on prevailing market rates in China; future adoption of low-cost alternative carbon sources could further reduce expenses.

d: Industrial CO_2 is priced at 35 USD·ton^{-1}, reflecting capture costs in the Chinese region (Source: CCUS and Global Power Generation Analysis)[32].

e: Process water is valued at 1.50 USD·ton^{-1}.

f: Solvent costs are based on Chinese market prices: MEA at 1,000 USD·ton^{-1} and MEG at 500 USD·ton^{-1}. A makeup rate of 5% per cycle is assumed to account for operational losses.

g: Fixed costs account for components that cannot be directly priced at the laboratory scale, such as annualized capital expenditures (CAPEX) and labor. Given the absence of a detailed engineering design at this preliminary stage, fixed costs were estimated using standard heuristic factors widely applied in the chemical process industry. Specifically, fixed costs are assumed to constitute a typical 15% of the total LCC, with variable OPEX accounting for the remaining 85%. The integration of this estimation effectively bridges the gap between laboratory-scale data and industrial realities, providing a more realistic economic evaluation. The mathematical formulation utilized is: Fixed costs = Variable OPEX × (0.15/0.85).

h: Product revenue for NaOH and H_2SO_4 is calculated on a 100% pure substance basis (e.g., the output of NaOH is equivalent to 250 kg of pure NaOH).

i: Same as note **h**; H_2SO_4 production is also calculated on a 100% basis.

j: The market value of $(NH_4)_2SO_4$ is estimated at 150% of the standard fertilizer-grade price. This premium reflects the higher purity and economic value achieved through the MEA-MEG purification process compared to conventional fertilizer products.

Supplementary Note 2 | Life cycle assessment (LCA) and LCC assessment methodology.

The economic feasibility of the upcycling processes was evaluated using an LCC assessment framework. The system boundary was defined as “gate-to-gate,” encompassing all core unit operations from the intake of waste feedstocks to the generation of final marketable products. To ensure a standardized comparison across all evaluated methods (BMED, New-SSA-Process, and this work), the functional unit was established as the processing of 1,000 kg of waste $Na_2SO_4{\cdot}10H_2O$. The variable OPEX were directly derived from the mass and energy balances established in the LCI. This includes the costs of electricity, chemical reagents, and process water/solvent. Market prices were determined using a five-year average to mitigate short-term market volatility (**Supplementary Fig. 35**). The variable OPEX is calculated as:

$$\textbf{Variable OPEX} = \sum (m_i \times P_{m,\,i}) + \sum (E_i \times P_{e,\,i})$$

where m_i and E_j represent the mass of material inputs and energy consumption, respectively, and $P_{m,\,i}$ and $P_{e,\,i}$ denote their corresponding unit market prices.

To satisfy the comprehensive scope of an LCC assessment, the inclusion of fixed costs is imperative. Given the preliminary development stage of the proposed technology, a bottom-up engineering cost estimation is subject to significant uncertainty. Therefore, we adopted a standard factorial approach widely utilized in the chemical process industry. Fixed costs, which encompass annualized CAPEX, plant maintenance, and labor, were designated to constitute 15% of the total LCC, with variable OPEX accounting for the remaining 85%. Accordingly, the fixed costs are calculated as follows:

$$\textbf{Fixed costs} = \text{Variable OPEX} \times (0.15/0.85)$$

Consequently, the total LCC integrates both the variable OPEX and the fixed costs to represent the overall production cost per functional unit:

$$\textbf{Total LCC} = \text{Variable OPEX} + \text{Fixed costs} = \text{Variable OPEX}/0.85$$

Finally, the economic viability was quantified using net profit and profit margin. Total revenue was calculated by multiplying the yield of each product and byproduct by its respective industrial market grade price. The economic metrics were determined using the following equations:

$$\textbf{Net Profit} = \text{Total Revenue} - \text{Total LCC}$$

$$\textbf{Profit Margin (\%)} = \text{Net Profit}/\text{Total Revenue} \times 100\%$$

A positive net profit and profit margin indicate economic feasibility, whereas negative values signify an economic deficit under the modeled scenarios.

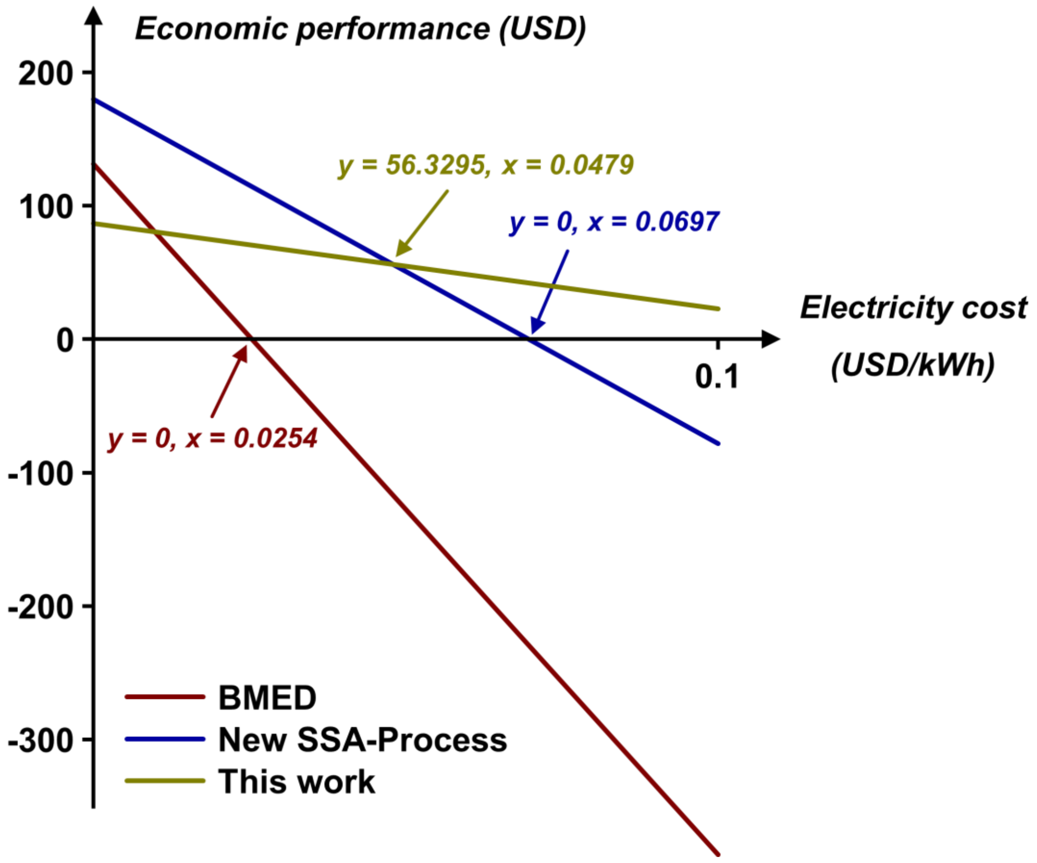

Supplementary Fig. 37 | Sensitivity analysis of economic performance to electricity price fluctuations. The linear relationship between net profit (USD) and electricity cost (USD/kWh) is compared for the BMED process (red), the New SSA-Process (blue), and the proposed thermochemical route (this work, olive). To align with the comprehensive LCC methodology, the net profit (y) is mathematically defined as a function of the electricity price (x) using the equation: y = **Total Revenue** − (**$E_{consumption}$** × x + **Other OPEX**)/0.85. In this model, $E_{consumption}$ is the electricity demand, Other OPEX encompasses other variable material costs, and the 0.85 denominator integrates the 15% fixed cost allowance (which includes annualized CAPEX).

The arrows indicate the break-even points where net profit equals zero. These occur at x = 0.0254 USD/kWh for the BMED process and x = 0.0697 USD/kWh for the New SSA-Process. A critical crossover threshold is identified at x = 0.0479 USD/kWh (y = 56.33 USD). Beyond this specific electricity price, the proposed route becomes definitively superior to the New SSA-Process in terms of economic viability. The significantly flatter slope of the proposed route visually demonstrates its

robust resilience against rising energy costs compared to the highly electricity-intensive BMED and New SSA-Processes.

Reviewer #3

Comments:

All concerns have been addressed, and the manuscript is suitable for publication as is.

Reply: We sincerely appreciate your positive assessment. We recognize that the manuscript's current quality owes much to your insightful review of the first version and your precise guidance on correcting errors. We are deeply grateful for the time and effort you have dedicated to improving our work.

Response to referees

Journal: Nature Communications

Manuscript ID: NCOMMS-25-65907B

Title: Upcycling of waste sodium sulfate to sodium carbonate and sulfur

Author(s): Hongya Wang, Shiyu Wang, Fengyin Zhou, Muya Cai, Jingjing Zhao, Danfeng Wang, Zhan Shi, Xiang Chen, Dihua Wang, Huayi Yin*

We are grateful to all the reviewers for their comments and suggestions. We have revised the manuscript, taking into full account the reviewers' comments and recommendations. Below are our point-to-point responses in the same order as the reviewers' comments. Replies are marked in blue, revisions are marked in purple.

Reviewer #2

Comments:

The authors have made substantial revisions in response to the concerns raised during the previous review round. The economic assessment framework has been significantly clarified, with a clearly defined system boundary, explicit cost components, and reproducible calculation procedures presented in the Supplementary Information. The scope and definition of the life cycle cost (LCC) assessment are now clearly articulated and consistently implemented, and the sensitivity analysis aligns with the revised methodology.

Overall, the revisions adequately address the prior concerns, and the manuscript is now suitable for publication in Nature Communications.

Reply: We are deeply thankful for your highly positive evaluation and recommendation for publication. We truly appreciate the time and expertise you dedicated to reviewing our work. Your insightful guidance on the economic assessment and LCC framework significantly elevated the quality and rigor of this study. Thank you once again for your immense support.
